# Supplementary material for: Stereospecific synthesis of silicon-stereogenic optically active silylboranes and general synthesis of chiral silyl Anions
Source: Nat Commun. 2023 Sep 9;14:5561. doi: 10.1038/s41467-023-41113-z (PMC10492825; doi:10.1038/s41467-023-41113-z)
Supplement: Supplementary file 1 — Supplementary Information [file 41467_2023_41113_MOESM1_ESM.pdf]

Supplementary Information for

Stereospecific Synthesis of Silicon-Stereogenic Optically Active Silylboranes and General Synthesis of Chiral Silyl Anions

Xihong Wang[a], Chi Feng[b], Julong Jiang[c], Satoshi Maeda[a,c], Koji Kubota*[a,b], Hajime Ito*[a,b]

[a]Institute for Chemical Reaction Design and Discovery (WPI-ICReDD), Hokkaido University, Sapporo, Hokkaido 060-8628, Japan.

[b]Division of Applied Chemistry, Graduate School of Engineering, Hokkaido University, Sapporo, Hokkaido 060-8628, Japan.

[c]Department of Chemistry, Faculty of Science, Hokkaido University Sapporo, Hokkaido 060-0815, Japan

Correspondence to: kbt@eng.hokudai.ac.jp (K.K.); hajito@eng.hokudai.ac.jp (H.I.)

TABLE OF CONTENTS

1. **General and Materials**
2. **Substrates Preparation**
3. **Typical Procedure of Platinum-Catalyzed Borylation of Chiral Hydrosilane**
4. **Characterization of Silylboranes**
5. **Typical Procedure of Si–Si Bond Forming Reaction**
6. **Characterization of Disilanes**
7. **Procedure of Protonation Study**
8. **Typical Procedure of Palladium-Catalyzed Silylation of Aryl Bromide**
9. **Typical Procedure of Palladium-Catalyzed Silylation of 1-(Bromomethyl)naphthalene**
10. **Typical Procedure of Copper-Catalyzed Silyl Conjugate Addition**
11. **Comparison with Other Catalytic Systems**
12. **$^{11}B\{^{1}H\}$ and $^{29}Si\{^{1}H\}$ NMR Experiments**
13. **Single Crystal Structure Analysis**
14. **DFT Calculations for the Stereospecific Reaction between Silylborane and MeLi followed by Protonation**
15. **Preliminary Study on Asymmetric Si–H Borylation**
16. **^{1}H, ^{13}C, ^{11}B and ^{29}Si NMR Spectra**
17. **HPLC Chromatograms**
18. **References**

1. General and Materials.

All reactions were performed in oven-dried glassware using conventional Schlenk techniques under a static pressure of nitrogen or argon. Materials were obtained from commercial suppliers and used as received unless otherwise noted. Dry solvents for the reactions were purchased from commercial suppliers, degassed via three freeze-pump-thaw cycles, and dried over molecular sieves (MS4A) before use. Cyclohexane purchased from Tokyo Chemical Industry Co. (TCI) was dried over CaH_2 and distilled before use. Tetrakis(triphenylphosphine)platinum(0) purchased from Tokyo Chemical Industry Co. (TCI) was used as received. A diethyl ether solution of methyllithium purchased from Kanto Chemical Co. was used as received. Silica Gel 60 N (40–100 μm, spherical, neutral) purchased from Kanto Chemical Co. was used as received. Preparative Thin layer chromatography (PTLC) was performed with 25 glass plates (20×20 cm) coated with Merck silica gel 60 F_{254} (layer thickness: 0.25 mm) purchased from Sigma-Aldrich Co. (1.05715.0001). NMR spectra were recorded on JEOL JNM-ECX400P, JNM-ECS400, and JNM-ECZ400 (^{1}H: 399.0, 400.5 MHz, ^{13}C: 100.3, 100.7 MHz, ^{11}B: 125.7 MHz, ^{29}Si: 77.8 MHz). Tetramethylsilane (δ = 0.00 for ^{1}H NMR and ^{29}Si NMR), $CDCl_3$ (δ = 77.0 for ^{13}C NMR) and THF-d_8 (δ = 1.72, 3.58 for ^{1}H NMR; δ = 67.0 for ^{13}C NMR) were employed as external standards, respectively. $BF_3 \cdot Et_2O$ was used as an external standard for ^{11}B NMR analysis. Multiplicity was reported as follows: s = singlet, brs = broad singlet, d = doublet, t = triplet, q = quartet, quint = quintet, sext = sextet, sept = septet, m = multiplet, dd = doublet of doublets, dt = doublet of triplets, td = triplet of doublets. Recycle preparative gel permeation chromatography (GPC) was conducted with a JAI LaboACE LC-5060 using $CHCl_3$ as eluent with JAIGEL-1HR and JAIGEL-2HR. Recycle preparative HPLC for resolution of racemic organosilicon compounds was also conducted with LC-9210NEXT using 100% hexane or 5% isopropanol/hexane as eluent with Daicel CHIRALCEL® OJ-3, 20 mm × 250 mmL or Daicel CHIRALPAK® ID-3, 20 mm × 250 mm. HPLC analyses with chiral stationary phase were carried out using a Hitachi Chromaster HPLC system [Daicel CHIRALPAK® IB N-3 (4.6 × 250 mm), Daicel CHIRALPAK® ID-3 (4.6 × 250 mm)], or D-2000 Elite HPLC System [Daicel CHIRALCEL® OJ-3 (4.6 × 250 mm), Daicel CHIRALCEL® OD-3 (4.6 × 250 mm), Daicel CHIRALCEL® OZ-3 (4.6 × 250 mm)]. The *es* value calculated according to the following formula:

$$\%es = \left(1 - \frac{Er_{\text{substrate}} - Er_{\text{product}}}{Er_{\text{substrate}}}\right) \times 100$$

where $Er_{\text{substrate}}$ is the ratio of the major enantiomer of the starting materials, and Er_{product} is the ratio of major enantiomer of the products.

Specific optical rotations were measured with HORIBA SEPA-300 and JASCO: P-2200. Single crystal X-ray structural analyses were carried out on a Rigaku XtaLAB PRO MM007 diffractometer using graphite monochromated Cu-Kα radiation. The structure was solved by direct methods and expanded using Fourier techniques. Non-hydrogen atoms were refined anisotropically. Hydrogen atoms were refined using the riding model. All calculations were performed using the Olex2 crystallographic software package except for refinement, which was performed using SHELXL-2013. High-resolution mass spectra were recorded at the Global Facility Center for Instrumental Analysis, Hokkaido University.

2. Substrates Preparation.

Preparation of (–)-(*R*)-[(1,1'-biphenyl)-4-yl](cyclohexyl)methylsilane [(–)-(*R*)-1a].

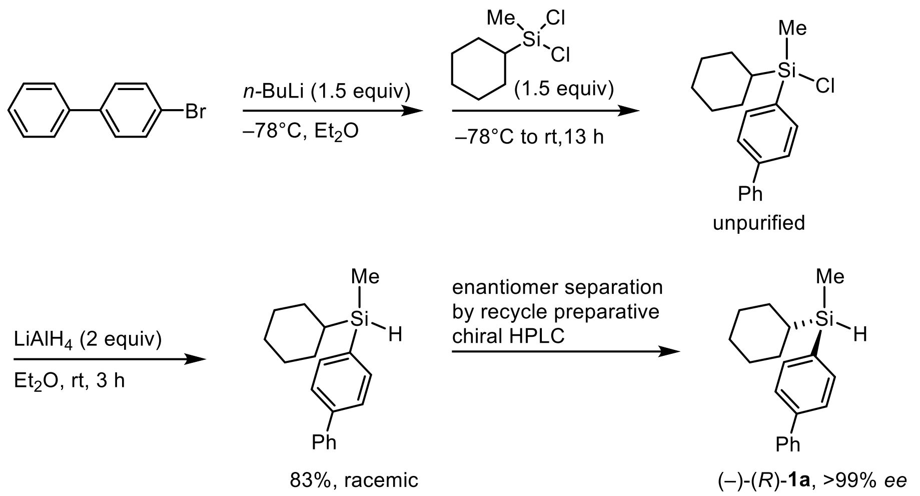

The reaction was performed according to the literature procedure.[1] *n*-BuLi (1.57 M in hexane, 4.5 mmol, 1.5 equiv) was slowly added by syringe to a solution of 4-bromobiphenyl (699.7 mg, 3.0 mmol, 1.0 equiv) in dry Et_2O (9.0 mL) at –78 °C under nitrogen. The mixture was warmed to room temperature. After stirring for 3 h, dichloro(cyclohexyl)methylsilane (874.4 mg, 4.4 mmol, 1.5 equiv) was slowly added by syringe at –78 °C. The mixture was then warmed to room temperature and stirred for 13 h. The solution was filtered with celite under an argon atmosphere with dry Et_2O as an eluent. Then $LiAlH_4$ (228.3 mg, 6.0 mmol, 2.0 equiv) in dry Et_2O (3.0 mL) was carefully added and stirred at room temperature for 3 h. The reaction mixture was quenched with 1.0 M aqueous HCl, and washed with sat. $NaHCO_3$, was then extracted with Et_2O three times and dried over $MgSO_4$. The organic layer was filtered and concentrated under reduced pressure. The crude mixture was purified by flash chromatography and GPC to afford the corresponding racemic product in 83% yield (702.1 mg, 2.5 mmol) as a white solid. Then (–)-(*R*)-[(1,1'-biphenyl)-4-yl](cyclohexyl)methylsilane [(–)-(*R*)-**1a**] (>99% *ee*) was isolated by recycle preparative HPLC with a chiral column (Daicel CHIRALCEL® OJ-3, 20 mm × 250 mm, hexane 100%, 5.0 mL/min); a single injection of 40 mg (±)-[1,1'-biphenyl]-4-yl(cyclohexyl)methylsilane yielded 16.6 mg of (–)-(*R*)-**1a**.

^{1}H NMR (399.0 MHz, $CDCl_3$, δ): 0.33 (d, J = 4.1 Hz, 3H), 0.89–1.02 (m, 1H), 1.11–1.31 (m, 5H), 1.63–1.82 (m, 5H), 4.23 (quint, J = 3.4 Hz, 1H), 7.34 (t, J = 7.4 Hz, 1H), 7.44 (t, J = 7.5 Hz, 2H), 7.56–7.63 (m, 6H). ^{13}C NMR (100.3 MHz, $CDCl_3$, δ): –7.7 (CH$_3$), 24.2 (CH), 26.8 (CH$_2$), 27.8 (CH$_2$), 27.9 (CH$_2$), 28.2 (CH$_2$), 126.4 (CH), 127.1 (CH), 127.4 (CH), 128.7 (CH), 134.6 (C), 135.2 (CH), 141.0 (C), 141.9 (C). ^{29}Si NMR (77.8 MHz, $CDCl_3$, δ): –9.4. EI (*m/z*): [M]$^+$ calcd for $C_{19}H_{24}Si$: 280.1647, found: 280.1653. $[\alpha]_D^{23}$ –9.3 (*c* 1.02 in $CHCl_3$, >99% *ee*). Daicel CHIRALCEL® OJ-3, hexane 100%, 0.5 mL/min, 40 °C, *R* isomer: t_R = 22.37 min; for the racemic compound: *R* isomer: t_R = 22.33 min, *S* isomer: t_S = 28.83 min.

Preparation of (+)-(*R*)-methyl(naphthalen-1-yl)phenylsilane [(+)-(*R*)-1b].

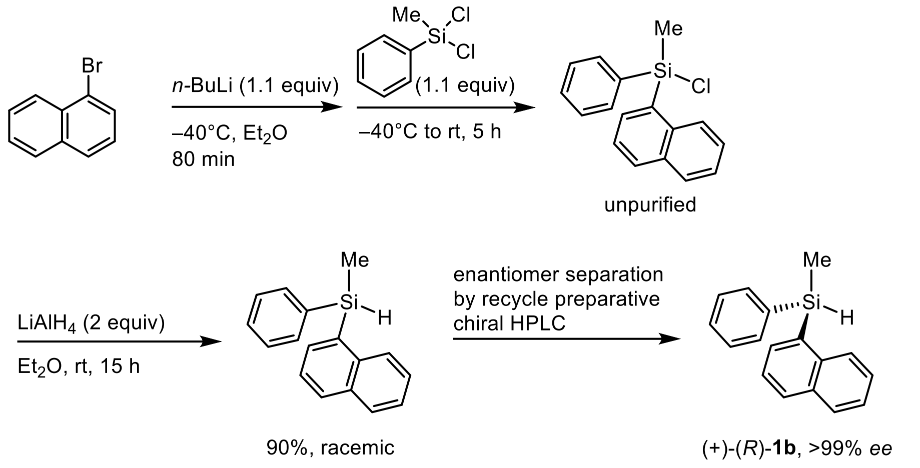

The reaction was performed according to the literature procedure.[2] *n*-BuLi (2.06 mL, 3.3 mmol, 1.1 equiv, 1.6 M in hexane) was slowly added by syringe to a solution of 1-bromonaphthalene (624.6 mg, 3.0 mmol, 1.0 equiv) in dry Et_2O (12.0 mL) at –40 °C under nitrogen. After stirring for 80 min, dichloro(methyl)phenylsilane (660.0 mg, 3.5 mmol, 1.2 equiv) was slowly added by syringe at –40 °C. The mixture was then warmed to room temperature and stirred for 5 h. The solution was filtered with celite under nitrogen with dry Et_2O as an eluent. Then $LiAlH_4$ (228.0 mg, 6.0 mmol, 2.0 equiv) in dry Et_2O (3.0 mL) was carefully added and stirred at room temperature for 15 h. The reaction mixture was quenched with 1.0 M aqueous HCl and washed with sat. $NaHCO_3$, was then extracted with Et_2O three times and dried over $MgSO_4$. The organic layer was filtered and concentrated under reduced pressure. The crude mixture was purified by flash chromatography to afford the corresponding racemic product in 90% yield (671.8 mg, 2.7 mmol) as a colorless solid. Then (+)-(*R*)-methyl(naphthalen-1-yl)phenylsilane [(+)-(*R*)-**1b**, >99% *ee*] was isolated by recycle preparative HPLC with a chiral column (Daicel CHIRALCEL® OJ-3, 20 mm × 250 mm, isopropanol/hexane = 5/95, 5.0 mL/min).

^{1}H NMR (399.0 MHz, $CDCl_3$, δ): 0.76 (d, *J* = 3.7 Hz, 3H), 5.35 (q, *J* = 4.0 Hz, 1H), 7.30–7.41 (m, 3H), 7.41–7.51 (m, 3H), 7.54–7.63 (m, 2H), 7.74 (d, *J* = 6.9 Hz, 1H), 7.83–7.89 (m, 1H), 7.91 (d, *J* = 8.2 Hz, 1H), 8.05 (d, *J* = 8.2 Hz, 1H). ^{13}C NMR (100.3 MHz, $CDCl_3$, δ): –4.5 (CH_3), 125.2 (*C*H), 125.6 (*C*H), 126.1 (*C*H), 127.9 (*C*H), 128.0 (*C*H), 128.9 (*C*H), 129.5 (*C*H), 130.5 (*C*H), 133.2 (*C*), 133.3 (*C*), 134.9 (*C*H), 135.2 (*C*H), 135.3 (*C*), 137.0 (*C*). ^{29}Si NMR (77.8 MHz, $CDCl_3$, δ): –20.0. EI (*m*/*z*): $[M]^+$ calcd for $C_{17}H_{16}Si$: 248.1021, found: 248.1014. $[\alpha]_D^{26}$ +37.5 (*c* 1.06 in $CHCl_3$, >99% *ee*). Daicel CHIRALCEL® OJ-3, IPA/hexane 5/95, 0.5 mL/min, 40 °C, *R* isomer: t_R = 11.79 min; for racemic compound: *R* isomer: t_R = 11.69 min, *S* isomer: t_S = 13.33 min.

Preparation of (+)-(*S*)-*tert*-butyl(methyl)phenylsilane [(+)-(*S*)-1c].

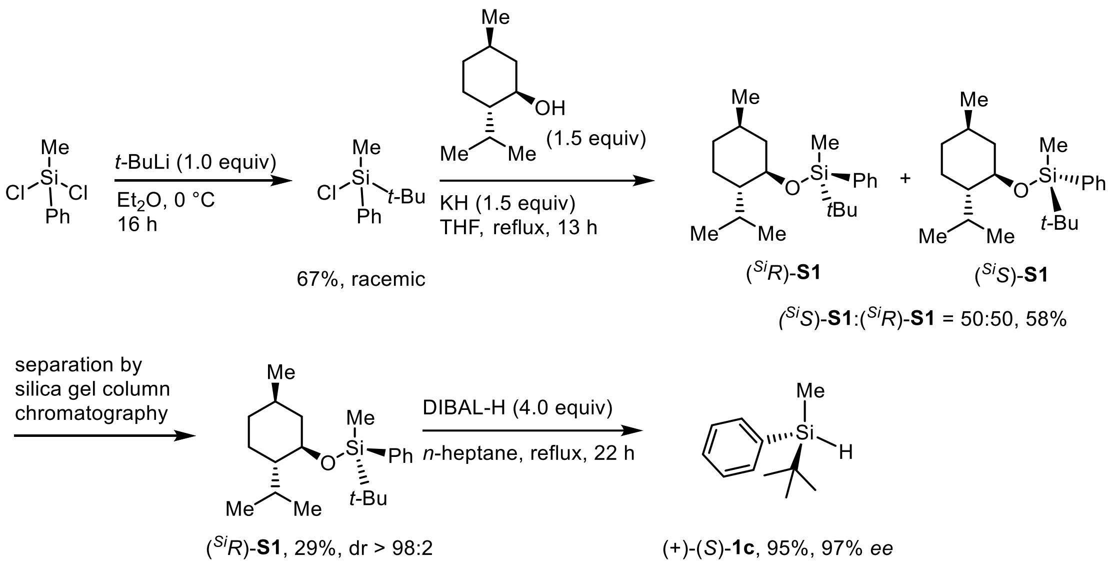

The reaction was performed according to the literature procedure with a slight modification.[3] *tert*-BuLi (12.5 mL, 1.6 M in Et_2O, 20.0 mmol, 1.0 equiv) was added to a solution of dichloro(methyl)phenylsilane (3.836 g, 20.1 mmol, 1.0 equiv) in dry Et_2O (12.0 mL) at 0 °C under nitrogen. After the solution was stirred 16 h at room temperature, lithium chloride was removed by filtration under nitrogen, and the crude product was distilled to give 67% of (±)-*tert*-butylchloro(methyl)phenylsilane (2.847 g, 13.4 mmol).

A solution of (–)-menthol (2.133 g, 13.6 mmol, 1.4 equiv, >99% *ee*) in dry THF (10.0 mL) was added to a suspension of potassium hydride (2.004 g, 15.0 mmol, 1.5 equiv, 30 % w/w) in dry THF (7.0 mL) at 0 °C under nitrogen. After refluxed for 3 h, a solution of (±)-*tert*-butylchloro(methyl)phenylsilane (2.195 g, 10.3 mmol, 1.0 equiv) in dry THF (17.0 mL) was added at room temperature. The reaction mixture was stirred at reflux for 13 h, then the mixture was quenched with the careful addition of H_2O followed by neutralization (pH = 7) with 1.0 M aqueous HCl. The organic layer was separated, and the aqueous phase was extracted with Et_2O. The combined organic layers were dried over $MgSO_4$ and concentrated under reduced pressure. The crude mixture was purified by flash chromatography on silica-gel with cyclohexane as eluent, affording a mixture of (^{Si}S)-**S1** and (^{Si}R)-**S1** (2.006 g, 6.03 mmol, 58%, dr = 50:50) as a colorless oil. After repeated flash chromatography with cyclohexane, (^{Si}R)-**S1** was isolated in 29% yield as a colorless oil (587.9 mg, 1.77 mmol, dr > 98:2). In the literature, (^{Si}S)-**S1** was separated for the synthesis of (–)-(*R*)-**1c**, whereas we used (^{Si}R)-**S1** for (+)-(*S*)-**1c**.[3] ^{1}H NMR (400.5 MHz, $CDCl_3$, δ): 0.40 (s, 3H), 0.75 (dd, *J* = 11.9, 6.9 Hz, 6H), 0.80–1.00 (m, 3H), 0.87 (s, 9H), 0.92 (d, *J* = 10.5 Hz, 3H), 1.18–1.32 (m, 2H), 1.54–1.63 (m, 2H), 1.68–1.77 (m, 1H), 2.30–2.44 (m, 1H), 3.48 (td, *J* = 10.3, 4.3 Hz, 1H), 7.28–7.39 (m, 3H), 7.52–7.60 (m, 2H).

DIBAL-H (7.2 mL, 7.2 mmol, 4.07 equiv, 1.0 M solution in hexane) was slowly added to a solution of (^{Si}R)-**S1** (587.9 mg, 1.77 mmol, 1.0 equiv) in dry *n*-heptane (9.6 mL) at 0 °C under nitrogen. The mixture was heated at reflux for 22 h before quenching with the careful addition of H_2O at 0 °C followed by neutralization (pH = 7) with 1.0 M aqueous HCl. The mixture was extracted with Et_2O. The combined organic layers were dried over $MgSO_4$ and concentrated under reduced pressure. Purification of the crude mixture via flash silica-gel column chromatography (100%

pentane) afforded colorless oil (+)-(*S*)-*tert*-butyl(methyl)phenylsilane [(+)-(*S*)-**1c**] (298.9 mg, 1.68 mmol, 95% yield, 97% *ee*).

^{1}H NMR (399.0 MHz, $CDCl_3$, δ): 0.33 (d, *J* = 4.1 Hz, 3H), 0.93 (s, 9H), 4.14 (q, *J* = 1.8 Hz, 1H), 7.31–7.41 (m, 3H), 7.51–7.56 (m, 2H). ^{13}C NMR (100.3 MHz, $CDCl_3$, δ): –8.5 (CH_3), 16.6 (*C*), 26.8 (CH_3), 127.6 (*C*H), 129.2 (*C*H), 135.1 (*C*H), 135.5 (*C*). ^{29}Si NMR (77.8 MHz, $CDCl_3$, δ): –1.4. EI (*m*/*z*): $[M]^+$ calcd for $C_{11}H_{18}Si$,178.1178; found,178.1178. $[\alpha]_D^{23}$ +11.9 (*c* 1.06 in $CHCl_3$, 97% *ee*). Daicel CHIRALCEL® OJ-3, MeOH 100%, 1.0 mL/min, 40 °C, *R* isomer: t_R = 4.69 min (minor), *S* isomer: t_S = 5.23 min (major); for the racemic compound: *R* isomer: t_R = 4.70 min, *S* isomer: t_S = 5.31 min.

Preparation of (–)-[3-(benzyloxy)propyl](cyclohexyl)methylsilane [(–)-1d].

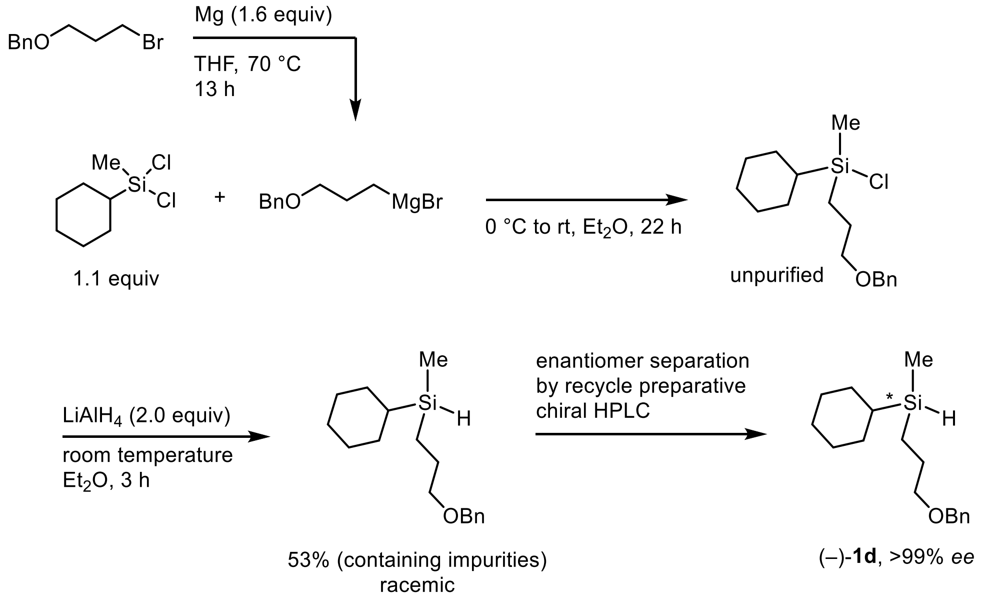

The reaction was performed according to the literature procedure.[4] In an oven-dried 50 mL two-necked round-bottomed flask equipped with a condenser were placed dry magnesium turnings (388.9 mg, 16.0 mmol, 1.6 equiv) under nitrogen. After the magnesium was further dried by a heat gun under nitrogen, a small amount of I_2 and dry THF (10.0 mL) were added to the flask. Then [(3-bromopropoxy)methyl]benzene (2.311 g, 10.1 mmol, 1.0 equiv) was slowly added to the reaction mixture at room temperature. After the mixture was stirred for 13 h at 70 °C, the heating was stopped, and the mixture was allowed to cool to room temperature. Then [3-(benzyloxy)propyl]magnesium bromide was added slowly to a solution of dichlo(cyclohexyl)methylsiane (2.207 g, 11.2 mmol, 1.1 equiv) in dry Et_2O (10.0 mL) at 0 °C. After the addition was complete, the reaction mixture was stirred for 22 h at room temperature. The solution was filtered with celite under nitrogen with dry Et_2O as an eluent. Then $LiAlH_4$ (760.2 mg, 20.0 mmol, 2.0 equiv) in dry Et_2O (10.0 mL) was carefully added to the mixture and stirred at room temperature for 3 h. The reaction mixture was quenched with 1.0 M aqueous HCl, and washed with sat. $NaHCO_3$, was then extracted with Et_2O three times and dried over $MgSO_4$. The organic layer was filtered and concentrated under reduced pressure. The crude product was purified by flash chromatography to afford the corresponding racemic product in 53% yield (1.467 g, 5.3 mmol, containing small amount of impurities as a colorless oil. Then (–)-[3-(benzyloxy)propyl](cyclohexyl)methylsilane [(–)-**1d**, >99% *ee*] was

isolated by recycle preparative HPLC with a chiral column (Daicel CHIRALPAK® ID-3, 20 mm × 250 mm, hexane 100%, 5.0 mL/min).

^{1}H NMR (399.0 MHz, $CDCl_3$, δ): –0.01 (d, *J* = 3.7 Hz, 3H), 0.50–0.67 (m, 2H), 0.67–0.77 (m, 1H), 1.06–1.31 (m, 5H), 1.60–1.76 (m, 7H), 3.44 (t, *J* = 6.9 Hz, 2H), 3.60 (sept, 1H), 4.50 (s, 2H), 7.25–7.31(m, 1H), 7.33 (d, *J* = 4.1 Hz, 4H). ^{13}C NMR (100.3 MHz, $CDCl_3$, δ): –8.2 (CH_3), 7.2 (CH_2), 23.4 (*C*H), 24.8 (CH_2), 26.8 (CH_2), 27.9 (CH_2), 28.0 (CH_2), 28.3 (CH_2), 72.8 (CH_2), 73.0 (CH_2), 127.5 (*C*H), 127.6 (*C*H), 128.3 (*C*H), 138.6 (*C*). ^{29}Si NMR (77.8 MHz, $CDCl_3$, δ): –5.6. EI (*m*/*z*): $[M-H]^+$ calcd for $C_{17}H_{27}OSi$: 275.1831, found: 275.1821. $[\alpha]_D^{26}$ –2.0 (*c* 1.02 in $CHCl_3$, >99% *ee*). Daicel CHIRALPAK® ID-3, hexane 100%, 0.5 mL/min, 40 °C, t_1 = 12.81 min; for racemic compound: t_1 = 12.87 min, t_2 = 15.21 min.

Preparation of (+)-benzyl(cyclohexyl)methylsilane [(+)-1e].

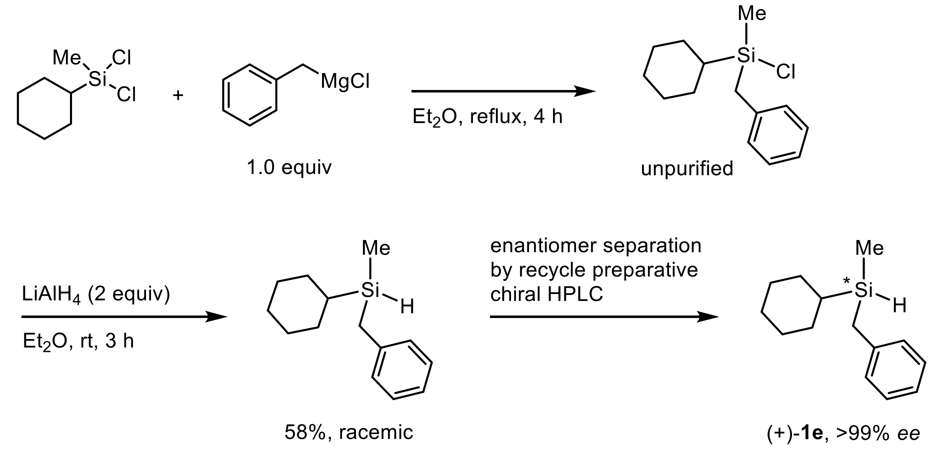

The reaction was performed according to the literature procedure.[5] Benzylmagnesium chloride (15 mL, 15.0 mmol, 1.0 equiv, 1.0 M in Et_2O) was slowly added by syringe to a solution of dichlo(cyclohexyl)methylsiane (2.976 g, 15.1 mmol, 1.0 equiv) in dry Et_2O (90.0 mL) at room temperature under nitrogen. The mixture was heated at reflux for 4 h and then cooled to room temperature. The solution was filtered with celite under nitrogen with dry Et_2O as an eluent. Then $LiAlH_4$ (1.154 g, 30.4 mmol, 2.0 equiv) in dry Et_2O (12.0 mL) was carefully added to the mixture and stirred at room temperature for 3 h. The reaction mixture was quenched with 1.0 M aqueous HCl, and washed with sat. $NaHCO_3$ was then extracted with Et_2O three times and dried over $MgSO_4$. The organic layer was filtered and concentrated under reduced pressure. The crude mixture was purified by flash chromatography to afford the corresponding racemic product in 58% yield (1.918 g, 8.8 mmol) as a colorless oil. Then (+)-benzyl(cyclohexyl)methylsilane [(+)-**1e**] (>99% *ee*) was isolated by recycle preparative HPLC (Daicel CHIRALCEL® OJ-3, 20 mm × 250 mm, hexane 100%, 5.0 mL/min).

^{1}H NMR (400.5 MHz, $CDCl_3$, δ): –0.03 (d, *J* = 3.7 Hz, 3H), 0.68–0.81 (m, 1H), 1.08–1.29 (m, 5H), 1.62–1.77 (m, 5H), 2.08 (dd, *J* = 13.8, 4.1 Hz, 1H), 2.20 (dd, *J* = 13.8, 2.8 Hz, 1H), 3.70 (sept, 1H), 7.00–7.12 (m, 3H), 7.17–7.26 (m, 2H). ^{13}C NMR (100.3 MHz, $CDCl_3$, δ): –8.5 (CH_3), 21.1 (CH_2), 23.1 (*C*H), 26.8 (CH_2), 27.8 (CH_2), 27.9 (CH_2), 28.2 (CH_2), 124.1 (*C*H), 128.2 (*C*H), 128.3 (*C*H), 140.3 (*C*). ^{29}Si NMR (77.8 MHz, $CDCl_3$, δ): –4.5. EI (*m*/*z*): $[M]^+$ calcd for $C_{14}H_{22}Si$: 218.1491, found: 218.1493. $[\alpha]_D^{23}$ +15.4 (*c* 0.75 in $CHCl_3$, >99% *ee*). Daicel CHIRALCEL® OJ-3, hexane 100%, 0.5 mL/min, 40 °C, t_1 = 8.67 min; for racemic compound: t_1 = 8.61 min, t_2 = 9.13 min.

Preparation of (–)-(*R*)-[4'-bromo-(1,1'-biphenyl)-4-yl](cyclohexyl)methylsilane [(–)-(*R*)-1f].

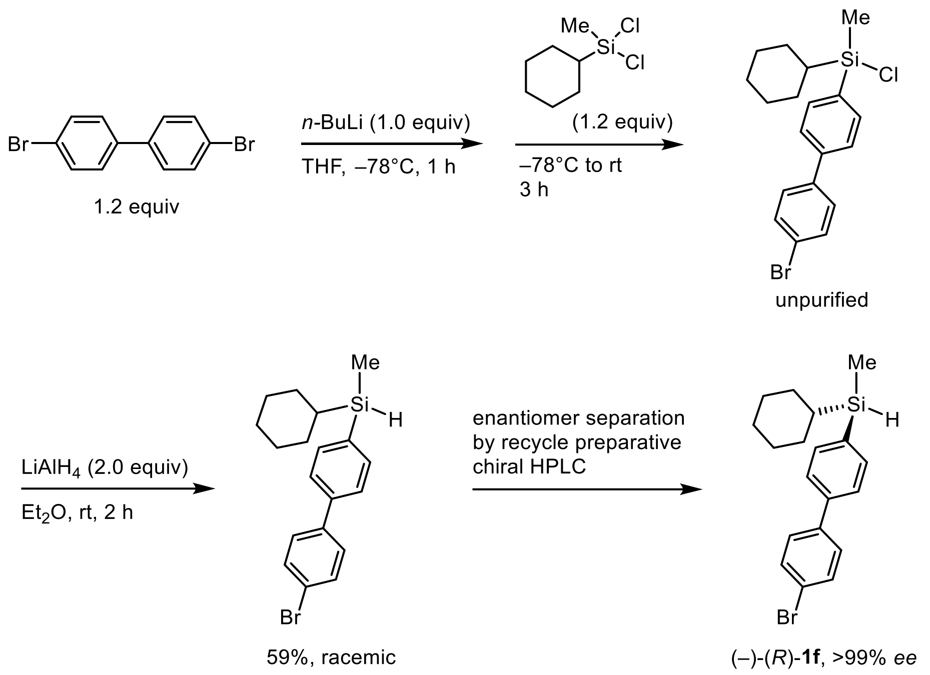

n-BuLi (1.27 mL, 2.0 mmol, 1.0 equiv, 1.57 M in hexane) was slowly added by syringe to a solution of 4,4'-dibromo-1,1'-biphenyl (748.8 mg, 2.4 mmol, 1.2 equiv) in dry THF (24.0 mL) at –78 °C under nitrogen. After stirring for 1.5 h, dichloro(cyclohexyl)methylsilane (472.6 mg, 2.4 mmol, 1.2 equiv) was slowly added by syringe at –78 °C. The mixture was then warmed to room temperature and stirred for 3 h. Then $LiAlH_4$ (152.2 mg, 4.0 mmol, 2.0 equiv) in dry Et_2O (2.0 mL) was carefully added to the mixture and stirred at room temperature for 2 h. The reaction mixture was quenched with 1.0 M aqueous HCl, and washed with sat. $NaHCO_3$, then extracted with Et_2O three times and dried over $MgSO_4$. The organic layer was filtered and concentrated under reduced pressure. The crude mixture was purified by flash chromatography and GPC to afford the corresponding racemic product in 59% yield (422.2 mg, 1.2 mmol) as a white solid. Then (–)-(*R*)-[4'-bromo-(1,1'-biphenyl)-4-yl](cyclohexyl)methylsilane [(–)-(*R*)-**1f**] (>99% *ee*) was isolated by recycle preparative HPLC with a chiral column (Daicel CHIRALCEL® OJ-3, 20 mm × 250 mm, hexane 100%, 5.0 mL/min).

^{1}H NMR (400.5 MHz, $CDCl_3$, δ): 0.33 (d, J = 3.7 Hz, 3H), 0.89–1.03 (m, 1H), 1.11–1.31 (m, 5H), 1.62–1.81 (m, 5H), 4.21 (quint, J = 3.4 Hz, 1H), 7.44–7.50 (m, 2H), 7.52–7.62 (m, 6H). ^{13}C NMR (99.5 MHz, $CDCl_3$, δ): –7.8 (CH_3), 24.2 (*C*H), 26.7 (CH_2), 27.80 (CH_2), 27.84 (CH_2), 28.1 (CH_2), 121.6 (*C*), 126.2 (*C*H), 128.7 (*C*H), 131.9 (*C*H), 135.2, (*C*), 135.3 (*C*H), 139.9 (*C*), 140.6 (*C*). ^{29}Si NMR (77.8 MHz, $CDCl_3$, δ): –9.3. EI (*m*/*z*): $[M]^+$ calcd for $C_{19}H_{23}BrSi$: 358.0752, found: 358.0752. $[\alpha]_D^{23}$ –8.4 (*c* 1.02 in $CHCl_3$, >99% *ee*). Daicel CHIRALCEL® OJ-3, hexane 100%, 0.5 mL/min, 40 °C, *R* isomer: t_R = 18.15 min; for the racemic compound: *R* isomer: t_R = 18.19 min, *S* isomer: t_S = 20.96 min.

Preparation of (+)-(*R*)-(Benzyloxy)(*tert*-butyl)(phenyl)silane [(+)-(*R*)-1g].

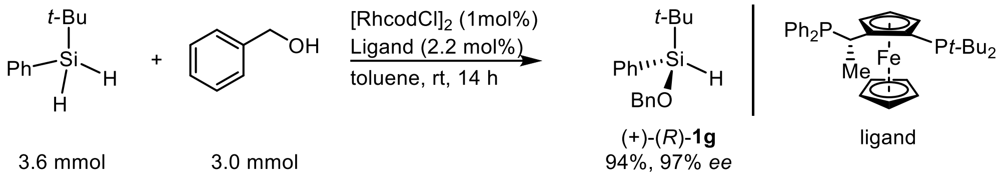

The reaction was performed according to the literature procedure.[6] $[Rh(cod)Cl]_2$ (14.8 mg, 0.03 mmol, 1 mol%) and ligand (35.8 mg, 0.066 mmol, 2.2 mol%) were added to a 50 mL flask in the glove box. After closing the flask with a rubber plug, the reaction flask was removed from the glove box, and then dry toluene (30 mL) was added to the flask via a syringe. Then *tert*-butyl(phenyl)silane (605.9 mg, 3.69 mmol. 1.2 equiv) and benzyl alcohol (326.4 mg, 3.02 mmol, 1.0 equiv) were added sequentially by syringes. After the reaction was stirred at room temperature for 12 h, the reaction mixture was filtered through a short pad of silica gel and evaporated under reduced pressure, then purified by silica-gel column chromatography with hexane eluent to afford (+)-(*R*)-(benzyloxy)(*tert*-butyl)(phenyl)silane in 94% yield (769.4 mg, 2.84 mmol, 97% *ee*) as a white solid. The enantiomeric excess was determined by chiral HPLC analysis. Corresponding racemic samples were obtained by carrying out the reactions with (±)-BINAP.

^{1}H NMR (400.0 MHz, $CDCl_3$, δ): 0.99 (d, J = 0.9 Hz, 9H), 4.76 (d, J = 0.9 Hz, 1H), 4.78 (d, J = 13.2 Hz, 1H), 4.81 (d, J = 13.2 Hz, 1H), 7.22–7.28 (m, 1H), 7.33 (d, J = 4.1 Hz, 4H), 7.35–7.45 (m, 3H), 7.56–7.62 (m, 2H). ^{13}C NMR (100.3 MHz, $CDCl_3$, δ): 18.1 (*C*), 25.6 (*C*H_3), 66.7 (*C*H_2), 126.2 (*C*H), 127.1 (*C*H), 127.8 (*C*H), 128.2 (*C*H), 130.0 (*C*H), 133.4 (*C*), 134.5 (*C*H), 140.5 (*C*). ^{29}Si NMR (77.8 MHz, $CDCl_3$, δ): 4.4. EI (*m*/*z*): $[M]^+$ calcd for $C_{17}H_{22}OSi$: 270.1440, found: 270.1434. $[\alpha]_D^{27}$ +53.1 (*c* 1.02 in $CHCl_3$, 97% *ee*). Daicel CHIRALCEL® OD-3 x2, hexane 100%, 0.5 mL/min, 40 °C, *S* isomer: t_S = 19.05 min (minor); *R* isomer: t_R = 19.49 min (major); for the racemic compound: *S* isomer: t_S = 18.91 min, *R* isomer: t_R = 19.39 min.

Preparation of (+)-(1*S*, 3*S*)-1-cyclopentyl-3-methyl-2,3-dihydro-1H-benzo[b]silole [(+)-(*S*)-1h].

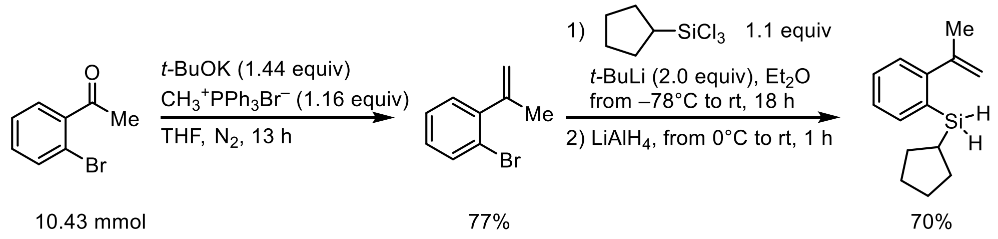

The reaction was performed according to the literature procedure.[7] Methyltriphenyl phosphonium bromide (4.306 g, 12.05 mmol, 1.16 equiv) in THF (25 mL) was treated with potassium *tert*-butoxide (1.690 g, 15.06 mmol, 1.44 equiv) under nitrogen. After stirring for 30 min at room temperature, a solution of 1-(2-bromophenyl)ethan-1-one (2.076 g, 10.43 mmol, 1.0 equiv) in THF (5.0 mL) was added dropwise to the above suspension, and the resulting mixture was stirred at room temperature. After the reaction was completed, the resulting solution was quenched with saturated NH_4Cl solution and extracted with Et_2O. The organic layer was washed with brine and dried over anhydrous $MgSO_4$, filtered, evaporated, and the residue was purified by silica gel chromatography column with hexane eluent to afford 1-bromo-2-(prop-1-en-2-yl)benzene in 77%

yield (1.591 g, 8.07 mmol) as a colorless oil.

To a solution of 1-bromo-2-(prop-1-en-2-yl)benzene (1.516 g, 7.69 mmol, 1.0 equiv.) derivative in ether (16 mL) was added *t*-BuLi (2.0 equiv., 1.7 M in pentane) dropwise at –78 °C under nitrogen. After stirred at –78 °C for 1.0 h, trichloro(cyclopentyl)silane (1.794 g, 9.10 mmol, 1.2 equiv.) in Et_2O (8.5 mL) was added dropwise to the above suspension and the reaction mixture was stirred for another 30 min at the same temperature. Then the reaction mixture was allowed to warm up to room temperature and stirred for 18 hours. $LiAlH_4$ (0.590 g, 15.56 mmol, 2.0 equiv) was added to the above suspension under nitrogen at 0 °C, and the reaction mixture was stirred at room temperature for another 1.0 h. The reaction was quenched with saturated NH_4Cl solution and was extracted with Et_2O. The organic layer was washed with brine and dried over anhydrous $MgSO_4$, filtered, evaporated, and the residue was purified by silica gel chromatography column with hexane eluent to afford cyclopentyl(2-(prop-1-en-2-yl)phenyl)silane in 70% yield (1.158 g, 7.01 mmol) as a colorless oil.

^{1}H NMR (391.8 MHz, $CDCl_3$, δ): 1.25–1.45 (m, 3H), 1.48–1.57 (m, 2H), 1.57–1.69 (m, 2H), 1.78–1.92 (m, 2H), 2.08 (t, *J* = 1.1 Hz, 3H), 4.23 (d, *J* = 3.1 Hz, 2H), 4.91–4.96 (m, 1H), 5.18 (quint, *J* = 1.6 Hz, 1H), 7.20 (d, *J* = 5.5 Hz, 1H), 7.22–7.28 (m, 1H), 7.36 (td, *J* = 7.6, 1.3 Hz, 1H), 7.60 (dd, *J* = 7.6, 1.3 Hz, 1H).

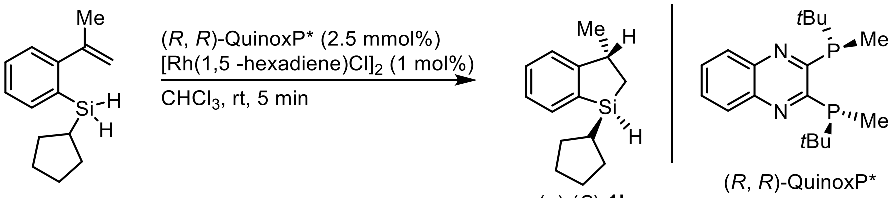

1.0 mmol

(+)-(*S*)-**1h**
90%, 96% *ee*, dr >20:1

The reaction was performed according to the literature procedure.[8] [Rh(1,5-hexadiene)Cl]$_2$ (4.4 mg, 0.010 mmol) and (*R*, *R*)-QuinoxP* (8.4 mg, 0.025 mmol) were charged to a 25 mL flask in the glove box. After closing the flask with a rubber plug, the reaction flask was removed from the glove box, and then dry $CHCl_3$ (2.5 mL) was added to the flask via a syringe. After stirring for 30 min at room temperature, 2.5 mL of the above suspension was added to a solution of cyclopentyl(2-(prop-1-en-2-yl)phenyl)silane (214.6, 0.99 mmol, 1.0 equiv) in dry $CHCl_3$ (2.5 mL) under nitrogen, and the resulting mixture was stirred at room temperature for 5 min. The reaction mixture was diluted with pentane and then filtered through a pad of silica gel with pentane as the eluent to remove the Rh catalyst. The residue was purified by silica gel chromatography column with hexane eluent to afford (+)-(1*S*, 3*S*)-1-cyclopentyl-3-methyl-2,3-dihydro-1H-benzo[b]silole in 90% yield (192.8 mg, 0.89 mmol) as a colorless oil.

^{1}H NMR (400.0 MHz, $CDCl_3$, δ): 0.85 (dq, *J* = 15.1, 2.7 Hz, 1H), 1.14–1.23 (m, 1H), 1.23–1.47 (m, 3H), 1.32 (d, *J* = 7.2 Hz, 3H), 1.47–1.66 (m, 4H), 1.79–1.94 (m, 2H), 3.35 (sext, *J* = 6.9 Hz, 1H), 4.60 (q, *J* = 2.9 Hz, 1H), 7.17–7.23 (m, 1H), 7.28 (d, *J* = 7.8 Hz, 1H), 7.32–7.38 (m, 1H), 7.58 (d, *J* = 7.3 Hz, 1H). ^{13}C NMR (100.3 MHz, $CDCl_3$, δ): 16.8 (CH_2), 22.8 (CH_3), 25.1 (*C*H), 26.87 (CH_2), 26.94 (CH_2), 28.7 (CH_2), 28.9 (CH_2), 39.2 (*C*H), 124.8 (*C*H), 125.7 (*C*H), 129.7 (*C*H), 133.1 (*C*H), 135.5 (*C*), 158.8 (*C*). ^{29}Si NMR (77.8 MHz, $CDCl_3$, δ): –1.34. EI (*m*/*z*): $[M]^+$ calcd for $C_{14}H_{20}Si$: 216.1334, found: 216.1335. $[\alpha]_D^{27}$ +24.8 (*c* 1.03 in $CHCl_3$, >96% *ee*). Daicel CHIRALCEL® OJ-3×2, hexane 100%, 0.5 mL/min, 20 °C, $t_{S,S}$ = 13.87 min (major); $t_{R,R}$ = 14.93 min(minor); for the racemic compound: $t_{S,S}$ = 13.97 min, $t_{R,R}$ = 15.09 min.

3. Typical Procedure of Platinum-Catalyzed Borylation of Chiral Hydrosilane.

(–)-(*R*)-[(1,1'-Biphenyl)-4-yl](cyclohexyl)methyl(4,4,5,5-tetramethyl-1,3,2-dioxaborolan-2-yl)silane [(–)-(*R*)-2a].

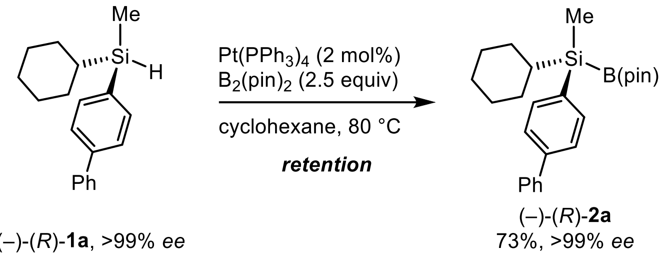

(–)-(*R*)-[(1,1'-Biphenyl)-4-yl](cyclohexyl)methylsilane [(–)-(*R*)-**1a**)] (84.2 mg, 0.301 mmol, 1.0 equiv), bis(pinacolato)diboron (190.5 mg, 0.75 mmol, 2.5 equiv) were placed in a vial with a screw cap containing a Teflon®-coated rubber septum under air. The vial was placed in a glove box, and then $Pt(PPh_3)_4$ (7.5 mg, 0.006 mmol, 2.0 mol %) was added to the vial in the glove box under an argon atmosphere. After closing the vial, the reaction vial was removed from the glove box, and then dry cyclohexane (0.3 mL) was added to the vial via a syringe. After being stirred at 80 °C for 23 h, the reaction mixture was analyzed by GC to check the completeness of the reaction. The mixture was directly filtered through celite with Et_2O as an eluent, and then the resultant solution was concentrated under reduced pressure. The crude product was purified by silica-gel column chromatography with hexane/Et_2O eluent (100:0 to 99:1) to give the corresponding product (–)-(*R*)-[(1,1'-biphenyl)-4-yl](cyclohexyl)methyl(4,4,5,5-tetramethyl-1,3,2-dioxaborolan-2-yl)silane [(–)-(*R*)-**2a**] in 73% yield (88.8 mg, 0.218 mmol) as a white solid.

^{1}H NMR (400.5 MHz, $CDCl_3$, δ): 0.33 (s, 3H), 0.87–1.00 (m, 1H), 1.10–1.37 (m, 5H), 1.28 (s, 12H), 1.61–1.81 (m, 5H), 7.30–7.37 (m, 1H), 7.43 (t, J = 7.8 Hz, 2H), 7.54–7.63 (m, 4H), 7.63–7.70 (m, 2H). ^{13}C NMR (100.3 MHz, $CDCl_3$, δ): –7.6 (CH$_3$), 25.0 (CH$_3$), 25.05 (CH$_3$), 25.15 (CH), 26.9 (CH$_2$), 28.1 (CH$_2$), 28.3 (CH$_2$), 83.3 (C), 126.3 (CH), 127.1 (CH), 127.2 (CH), 128.7 (CH), 135.4 (CH), 136.0 (C), 141.1 (C), 141.3 (C). ^{11}B NMR (125.7 MHz, $CDCl_3$, δ): 34.9. ^{29}Si NMR (77.8 MHz, $CDCl_3$, δ): There are no other signals except the peak of tetramethylsilane standard, probably because of the quadrupole effect of the boron atom. ESI (*m*/*z*): $[M+Na]^+$ calcd for $C_{25}H_{35}{}^{10}BO_2SiNa$: 428.2428, found: 428.2422. $[\alpha]_D^{25}$ –6.8 (*c* 1.03 in $CHCl_3$, >99% *ee*). Daicel CHIRALPAK® IB N-3, hexane 100%, 0.5 mL/min, 40 °C, *R* isomer: t_R = 13.54 min; for the racemic compound: *S* isomer: t_S = 10.39 min, *R* isomer: t_R = 13.50 min.

4. Characterization of Silylboranes.

(+)-(*R*)-Methyl(naphthalen-1-yl)phenyl(4,4,5,5-tetramethyl-1,3,2-dioxaborolan-2-yl)silane [(+)-(*R*)-2b].

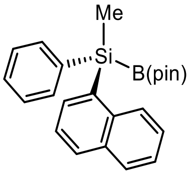

The reaction was conducted with (+)-(*R*)-**1b** (74.6 mg, 0.300 mmol, >99% *ee*) according to the typical procedure of the platinum-catalyzed borylation of (–)-(*R*)-**1a**. The product (+)-(*R*)-**2b** was obtained in 72% yield with >99% *ee* (81.3 mg, 0.217 mmol, viscous oil). Because we could not find HPLC conditions and chiral columns that could separate the racemic mixture of (±)-**2b**, from the results of (+)-(*R*)-**1b** (>99% *ee*) and (–)-(*S*)-**3b** (>99% *ee*), we deduce that the enantiomeric purity of (+)-(*R*)-**2b** is >99% *ee*.

^{1}H NMR (399.0 MHz, THF-d_8, δ): a. 0.68 (s, 3H), 1.229 (s, 6H), 1.233 (s, 6H), 7.22–7.32 (m, 3H), 7.32–7.43 (m, 3H), 7.57–7.63 (m, 2H), 7.65 (d, *J* = 6.9. Hz, 1H), 7.78–7.88 (m, 2H), 8.10 (d, *J* = 8.2 Hz, 1H). ^{13}C NMR (100.3 MHz, THF-d_8, δ): –3.7 (CH_3), 24.8 (CH_3), 24.9 (CH_3), 84.2 (*C*), 125.5 (*C*H), 125.7 (*C*H), 125.8 (*C*H), 128.1 (*C*H), 129.1 (*C*H), 129.2 (*C*H), 130.2 (*C*H), 134.1 (*C*), 135.3 (*C*H), 135.6 (*C*H), 137.6 (*C*), 137.9 (*C*). ^{11}B NMR (125.7 MHz, THF-d_8, δ): 35.1. ^{29}Si NMR (77.8 MHz, THF-d_8, δ): There are no other signals except the peak of tetramethylsilane standard, probably because of the quadrupole effect of the boron atom. ESI (m/z): $[M+Na]^+$ calcd for $C_{23}H_{27}{}^{10}BO_2SiNa$: 396.1802, found: 396.1806. $[\alpha]_D^{26}$ +5.6 (c 2.04 in cyclohexane, >99% *ee*).

(+)-(*S*)-*tert*-Butyl(methyl)phenyl(4,4,5,5-tetramethyl-1,3,2-dioxaborolan-2-yl)silane [(+)-(*S*)-2c].

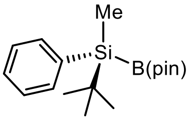

The reaction was conducted with (+)-(*S*)-**1c** (53.7 mg, 0.301 mmol, 97% *ee*) according to the typical procedure of the platinum-catalyzed borylation of (–)-(*R*)-**1a**. The product (+)-(*S*)-**2c** was obtained in 72% yield with 97% *ee* (66.2 mg, 0.218 mmol, colorless thick oil).

^{1}H NMR (399.0 MHz, $CDCl_3$, δ): 0.34 (s, 3H), 0.91 (s, 9H), 1.267 (s, 6H), 1.274 (s, 6H), 7.28–7.38 (m, 3H), 7.57–7.67 (m, 2H). ^{13}C NMR (100.3 MHz, $CDCl_3$, δ): –8.3 (CH_3), 17.1 (*C*), 25.0 (CH_3), 27.1 (CH_3), 83.3 (*C*), 127.3 (*C*H), 128.5 (*C*H), 135.4 (*C*H), 136.8 (*C*). ^{11}B NMR (125.7 MHz, $CDCl_3$, δ): 34.7. ^{29}Si NMR (77.8 MHz, $CDCl_3$, δ): There are no other signals except the peak of tetramethylsilane standard, probably because of the quadrupole effect of the boron atom. ESI (*m*/*z*): $[M+Na]^+$ calcd for $C_{17}H_{29}{}^{10}BO_2SiNa$: 326.1958, found: 326.1955. $[\alpha]_D^{23}$ +10.8 (*c* 1.02 in $CHCl_3$, 97% *ee*). Daicel CHIRALCEL® OD-3×2, hexane 100%, 0.5 mL/min, 40 °C, *R* isomer: t_R = 14.25 min (minor), *S* isomer: t_S = 15.04 min (major); for the racemic compound: *R* isomer: t_R = 14.27 min, *S* isomer: t_S = 15.09 min.

(+)-[3-(Benzyloxy)propyl](cyclohexyl)methyl(4,4,5,5-tetramethyl-1,3,2-dioxaborolan-2-yl)silane [(+)-2d].

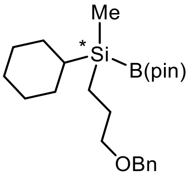

The reaction was conducted with (–)-**1d** (83.6 mg, 0.302 mmol, >99% *ee*) according to the typical procedure of the platinum-catalyzed borylation of (–)-(*R*)-**1a**. The product (+)-**2d** was obtained in 67% yield with >99% *ee* (81.5 mg, 0.203 mmol, colorless thick oil).

^{1}H NMR (399.0 MHz, $CDCl_3$, δ): –0.01 (s, 3H), 0.50–0.67 (m, 2H), 0.68–0.80 (m, 1H), 1.06–1.31 (m, 5H), 1.22 (s, 12 H), 1.60–1.76 (m, 7H), 3.43 (t, *J* = 7.1 Hz, 2H), 4.50 (s, 2H), 7.24–7.32 (m, 1H), 7.33 (d, *J* = 4.1 Hz, 4H). ^{13}C NMR (100.3 MHz, $CDCl_3$, δ): –8.0 (CH_3), 7.6 (CH_2), 24.1 (*C*H), 24.9 (CH_2), 25.0 (CH_3), 27.0 (CH_2), 28.1 (CH_2), 28.2 (CH_2), 28.5 (CH_2), 72.7 (CH_2), 73.5 (CH_2), 83.0 (*C*), 127.4 (*C*H), 127.6 (*C*H), 128.3 (*C*H), 138.7 (*C*). ^{11}B NMR (125.7 MHz, $CDCl_3$, δ): 34.9. ^{29}Si NMR (77.8 MHz, $CDCl_3$, δ): There are no other signals except the peak of tetramethylsilane standard, probably because of the quadrupole effect of the boron atom. ESI (*m*/*z*): $[M+Na]^+$ calcd for $C_{23}H_{39}{}^{10}BO_3SiNa$: 424.2690, found: 424.2685. $[\alpha]_D^{26}$ +3.4 (*c* 1.04 in $CHCl_3$, >99% *ee*). Daicel CHIRALCEL® OD-3, hexane 100%, 0.5 mL/min, 40 °C, t_1 = 14.25 min (minor); t_2 = 14.96 min (major), for the racemic compound: t_1= 14.19 min, t_2 = 15.17 min. The integral ratio of the racemic mixture of (±)-**2d** is 41:59, probably because the peak of a small number of impurities overlaps with one of the peaks of the racemate. But from the results of (–)-**1d** (>99% *ee*) and (–)-**3d** (>99% *ee*), we deduce that the enantiomeric purity of (+)-**2d** is >99% *ee*.

(–)-Benzyl(cyclohexyl)methyl(4,4,5,5-tetramethyl-1,3,2-dioxaborolan-2-yl)silane [(–)-2e].

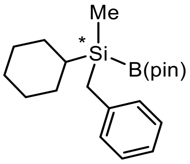

The reaction was conducted with (+)-**1e** (65.1 mg, 0.298 mmol, >99% *ee*) according to the typical procedure of the platinum-catalyzed borylation of (–)-(*R*)-**1a**. The product (–)-**2e** was obtained in 71% yield with >99% *ee* (73.0 mg, 0.212 mmol, colorless thick oil).

^{1}H NMR (399.0 MHz, $CDCl_3$, δ): –0.06 (s, 3H), 0.70–0.83 (m, 1H), 1.10–1.28 (m, 5H), 1.19 (s, 6H), 1.21 (s, 6H), 1.63–1.79 (m, 5H), 2.19 (d, *J* = 13.7 Hz, 1H), 2.24 (d, *J* = 13.7 Hz, 1H), 7.00–7.10 (m, 3H), 7.13–7.21 (m, 2H). ^{13}C NMR (100.3 MHz, $CDCl_3$, δ): –8.2 (CH_3), 21.6 (CH_2), 24.1 (*C*H), 25.00 (CH_3), 25.02 (CH_3), 27.0 (CH_2), 28.0 (CH_2), 28.1 (CH_2), 28.5 (CH_2), 83.2 (*C*), 123.7 (*C*H), 128.0 (*C*H), 128.3 (*C*H), 141.1 (*C*). ^{11}B NMR (125.7 MHz, $CDCl_3$, δ): 34.5. ^{29}Si NMR (77.8 MHz, $CDCl_3$, δ): There are no other signals except the peak of tetramethylsilane standard, probably because of the quadrupole effect of the boron atom. ESI (*m*/*z*): $[M]^+$ calcd for $C_{20}H_{33}{}^{10}BO_2Si$: 343.2379, found: 343.2378. $[\alpha]_D^{25}$ –0.95 (*c* 1.05 in $CHCl_3$, >99% *ee*). Daicel CHIRALPAK® IB N-3, hexane 100%, 0.5 mL/min, 40 °C, t_1 = 8.81 min; for the racemic compound: t_1= 8.71 min, t_2 = 9.12 min.

(–)-(*R*)-[4'-Bromo-(1,1'-biphenyl)-4-yl](cyclohexyl)methyl(4,4,5,5-tetramethyl-1,3,2-dioxaborolan-2-yl)silane [(–)-(*R*)-2f].

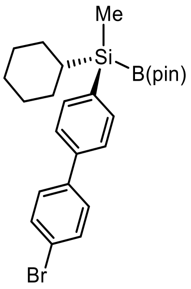

The reaction was conducted with (–)-(*R*)-**1f** (107.8 mg, 0.300 mmol, >99% *ee*) according to the typical procedure of platinum-catalyzed borylation of (–)-(*R*)-**1a**. The product (–)-(*R*)-**2f** was obtained in 72% yield with >99% *ee* (105 mg, 0.216 mmol, white solid).

^{1}H NMR (400.5 MHz, $CDCl_3$, δ): 0.33 (d, J = 1.8 Hz, 3H), 0.85–1.01 (m, 1H), 1.08–1.37 (m, 5H), 1.27 (s, 12H), 1.60–1.82 (m, 5H), 7.42–7.49 (m, 2H), 7.49–7.58 (m, 4H), 7.66 (d, J = 6.4 Hz, 2H). ^{13}C NMR (100.3 MHz, $CDCl_3$, δ): –7.6 (CH_3), 25.03 (CH_3), 25.05 (CH_3), 25.09 (*C*H), 26.9 (CH_2), 28.1 (CH_2), 28.3 (CH_2), 83.3 (*C*), 121.4 (*C*), 126.0 (*C*H), 128.7 (*C*H), 131.8 (*C*H), 135.5 (*C*H), 136.7 (*C*), 139.9 (*C*), 140.2 (*C*). ^{11}B NMR (125.7 MHz, $CDCl_3$, δ): 34.3. ^{29}Si NMR (77.8 MHz, $CDCl_3$, δ): There are no other signals except the peak of tetramethylsilane standard, probably because of the quadrupole effect of the boron atom. ESI (*m*/*z*): $[M+Na]^+$ calcd for $C_{25}H_{34}{}^{10}BBrO_2SiNa$: 506.1533, found: 506.1526. $[\alpha]_D^{25}$ –9.3 (*c* 1.02 in $CHCl_3$, >99% *ee*). Daicel CHIRALCEL® OD-3, hexane 100%, 0.5 mL/min, 40 °C, *R* isomer: t_R = 12.56 min; for the racemic compound: *S* isomer: t_S = 11.00 min, *R* isomer: t_R = 12.49 min.

(+)-(*R*)-(Benzyloxy)(tert-butyl)(phenyl)(4,4,5,5-tetramethyl-1,3,2-dioxaborolan-2-yl)silane [(+)-(*R*)-2g].

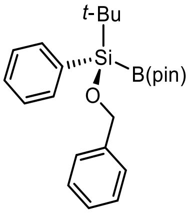

The reaction was conducted with (+)-(*R*)-**1g** (80.7 mg, 0.298 mmol, 97% *ee*) according to the typical procedure of platinum-catalyzed borylation of (–)-(*R*)-**1a**. 5 mol% of $Pt(PPh_3)_4$ catalyst was used.. The product (+)-(*R*)-**2g** was obtained in 34% yield with 95% *ee* (40.7 mg, 0.103 mmol, colorless thick oil).

^{1}H NMR (399.0 MHz, $CDCl_3$, δ): 0.98 (s, 9H), 1.25 (s, 6H), 1.26 (s, 6H), 4.81 (d, J = 13.2 Hz, 1H), 4.90 (d, J = 12.8 Hz, 1H), 7.20–7.27 (m, 1H), 7.29–7.42 (m, 7H), 7.62–7.70 (m, 2H). ^{13}C NMR (100.3 MHz, $CDCl_3$, δ):19.1 (*C*), 25.0 (CH_3), 25.1 (CH_3), 26.0 (CH_3), 66.2 (CH_2), 83.5 (*C*), 126.2 (*C*H), 126.7 (*C*H), 127.5 (*C*H), 128.0 (*C*H), 129.2 (*C*H), 134.6 (*C*H), 135.7 (*C*), 141.6 (*C*). ^{11}B NMR (125.7 MHz, $CDCl_3$, δ): 33.2. ^{29}Si NMR (77.8 MHz, $CDCl_3$, δ): There are no other signals except the peak of tetramethylsilane standard, probably because of the quadrupole effect of the boron atom. ESI (*m*/*z*): $[M+Na]^+$ calcd for $C_{23}H_{33}{}^{10}BO_3SiNa$:418.2221, found: 418.2221. $[\alpha]_D^{27}$ +16.3 (*c* 1.01 in $CHCl_3$, 95% *ee*). Daicel CHIRALCEL® OZ-3, hexane 100%, 0.5 mL/min, 40 °C, *R* isomer: t_R =

8.71 min (major), *S* isomer: t_S = 9.83 min (minor); for the racemic compound: *R* isomer: t_R = 8.68 min, *S* isomer: t_S = 9.59 min.

(–)-(1*S*, 3*S*)-1-Cyclopentyl-3-methyl-1-(4,4,5,5-tetramethyl-1,3,2-dioxaborolan-2-yl)-2,3-dihydro-1H-benzo[b]silole [(–)-(*S*)-2h]

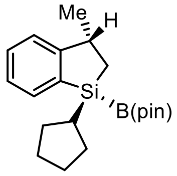

The reaction was conducted with (+)-(*S*)-**1h** (65.6 mg, 0.303 mmol, 96% *ee*) according to the typical procedure of platinum-catalyzed borylation of (–)-(*R*)-**1a**. The product (–)-(*S*)-**2h** was obtained in 71% yield with 96% *ee* (73.4 mg, 0.214 mmol, colorless thick oil).

^{1}H NMR (399.0 MHz, $CDCl_3$, δ): 0.86 (dd, *J* = 14.6, 6.4 Hz, 1H), 1.11–1.25 (m, 1H), 1.225 (s, 6H), 1.228 (s, 6H), 1.33 (d, *J* = 6.8 Hz, 3H), 1.25–1.42 (m, 3H), 1.42–1.63 (m, 4H), 1.75–1.88 (m, 2H), 3.33 (sext, *J* = 7.0 Hz, 1H), 7.17 (t, *J* = 7.1 Hz, 1H), 7.25 (d, *J* = 6.9 Hz, 1H), 7.28–7.33 (m, 1H), 7.60 (d, *J* = 7.3 Hz, 1H). ^{13}C NMR (100.3 MHz, $CDCl_3$, δ): 18.2 (CH_2), 23.9 (CH), 24.7 (CH_3), 24.9 (CH_3), 25.0 (CH_3), 26.9 (CH_2), 29.0 (CH_2), 29.2 (CH_2), 39.8 (CH), 83.3 (C), 124.3 (CH), 125.5 (CH), 129.0 (CH), 133.3 (CH), 137.9 (C), 158.5 (C). ^{11}B NMR (125.7 MHz, $CDCl_3$, δ): 34.1. ^{29}Si NMR (77.8 MHz, $CDCl_3$, δ): There are no other signals except the peak of tetramethylsilane standard, probably because of the quadrupole effect of the boron atom. ESI (*m*/*z*): $[M+Na]^+$ calcd for $C_{20}H_{31}{}^{10}BO_2SiNa$:364.2115, found: 364.2111. $[\alpha]_D^{28}$ –11.9 (*c* 1.01 in $CHCl_3$, 96% *ee*). Daicel CHIRALPAK® IB N-3, hexane 100%, 0.5 mL/min, 20 °C, $t_{R,R}$ = 8.86 min (minor), $t_{S,S}$ = 9.29 min (major); for the racemic compound: $t_{R,R}$ = 8.70 min, $t_{S,S}$ = 9.39 min.

5. Typical Procedure of Si–Si Bond Forming Reaction.

(+)-(*S*)-1-[(1,1'-Biphenyl)-4-yl]-1-cyclohexyl-1-methyl-2,2,2-triphenyldisilane [(+)-(*S*)-3a].

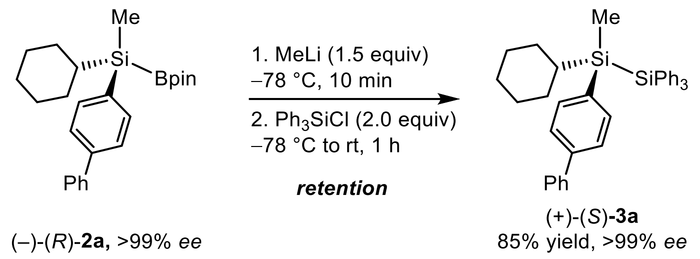

(–)-(*R*)-[(1,1'-Biphenyl)-4-yl](cyclohexyl)methyl(4,4,5,5-tetramethyl-1,3,2-dioxaborolan-2-yl)silane [(–)-(*R*)-**2a**] (40.6 mg, 0.100 mmol, 1.0 equiv) was placed in a vial with a screw cap containing a Teflon®-coated rubber septum. After the vial was connected to manifold with nitrogen and a vacuum line through a needle, it was evacuated and backfilled with nitrogen. This cycle was repeated three times. Dry THF (0.5 mL) was added to the vial through the rubber septum using a syringe, and the mixture was cooled to –78 °C. Then, MeLi (1.1 M in Et_2O, 136 μL, 0.150 mmol, 1.5 equiv) was added to the vial. After the mixture was stirred at –78 °C for 10 min, chlorotriphenylsilane (59.2 mg, 0.201 mmol, 2.0 equiv) was added dropwise to the vial at –78 °C. The mixture was allowed to warm to room temperature and stirred for 1 h. After that, the mixture was quenched by the addition of EtOH and filtered through a short silica-gel column with Et_2O as an eluent, then the resultant solution was concentrated under reduced pressure. The crude mixture was purified by silica-gel column chromatography with hexane/Et_2O eluent (100:0 to 99:1) and then further purified by GPC to give the corresponding product (+)-(*S*)-1-[(1,1'-biphenyl)-4-yl]-1-cyclohexyl-1-methyl-2,2,2-triphenyldisilane [(+)-(*S*)-**3a**] in 85% yield (45.6 mg, 0.085 mmol) with >99% *ee* as a white solid.

^{1}H NMR (400.5 MHz, $CDCl_3$, δ): 0.51 (s, 3H), 1.03–1.31 (m, 6H), 1.48–1.77 (m, 5H), 7.26–7.47 (m, 22H), 7.58 (d, J = 8.3 Hz, 2H). ^{13}C NMR (100.3 MHz, $CDCl_3$, δ): –7.0 (CH$_3$), 25.7 (CH), 26.7 (CH$_2$), 28.1 (CH$_2$), 28.2 (CH$_2$), 28.3 (CH$_2$), 28.9 (CH$_2$), 126.1 (CH), 127.0 (CH), 127.3 (CH), 127.8 (CH), 128.7 (CH), 129.0 (CH), 135.39 (CH), 135.42 (C), 135.6 (C), 136.2 (CH), 140.95 (C), 140.98 (C). ^{29}Si NMR (77.8 MHz, $CDCl_3$, δ): –22.4, –17.9. EI (*m*/*z*): $[M]^+$ calcd for $C_{37}H_{38}Si_2$: 538.2512, found: 538.2500. $[\alpha]_D^{23}$ +11.7 (*c* 1.07 in $CHCl_3$, >99% *ee*). Daicel CHIRALCEL® OD-3, hexane 100%, 0.5 mL/min, 40 °C, *S* isomer: t_S = 23.29 min; for the racemic compound: *R* isomer: t_R = 21.73 min, *S* isomer: t_S = 23.85 min.

6. Characterization of Disilanes.

(–)-(*S*)-1-Benzyl-1,1,2-trimethyl-2-(naphthalen-1-yl)-2-phenyldisilane [(–)-(*S*)-3b].

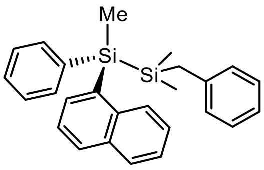

The reaction was conducted with (+)-(*R*)-**2b** (37.7 mg, 0.101 mmol, >99% *ee*) and benzylchlorodimethylsilane (37.4 mg, 0.202 mmol, 2.0 equiv) according to the typical procedure of Si–Si bond forming reaction for (+)-(*S*)-**3a**. In addition, the mixture was stirred at –78 °C for 30 min after MeLi was added to the vial, then benzylchlorodimethylsilane was added dropwise to the vial at –78 °C. The product (–)-(*S*)-**3b** was obtained in 51% yield with >99% *ee* (20.4 mg, 0.051 mmol, colorless viscous oil). The product was purified by silica-gel column chromatography with hexane/Et_2O eluent (100:0 to 99:1) followed by GPC and PTLC (hexane/ ethyl acetate 96:4). This product contains a small number of unremovable impurities.

^{1}H NMR (400.5 MHz, $CDCl_3$, δ): 0.13 (s, 3H), 0.16 (s, 3H), 0.69 (s, 3H), 2.17 (s, 2H), 6.82 (d, *J* = 7.3 Hz, 2H), 7.04 (t, *J* = 7.3 Hz, 1H), 7.13 (t, *J* = 7.3 Hz, 2H), 7.27–7.38 (m, 4H), 7.40–7.51 (m, 4H), 7.66–7.72 (m, 1H), 7.87 (t, *J* = 8.0 Hz, 2H), 7.92 (d, *J* = 8.2 Hz, 1H). ^{13}C NMR (100.3 MHz, $CDCl_3$, δ): –3.1 (CH_3), –3.0 (CH_3), 25.0 (*C*H), 124.0 (*C*H), 125.3 (*C*H), 125.5 (*C*H), 127.9 (*C*H), 128.0 (*C*H), 128.2 (*C*H), 128.7 (CH), 128.9 (*C*H), 129.0 (*C*H), 130.0 (*C*H), 133.4 (*C*), 134.8 (*C*H), 134.9 (*C*), 135.3 (*C*H), 137.1 (*C*), 137.8 (*C*), 139.7 (*C*). ^{29}Si NMR (77.8 MHz, $CDCl_3$, δ): –21.0, –16.3. EI (*m*/*z*): $[M]^+$ calcd for $C_{26}H_{28}Si_2$: 396.1730, found: 396.1723. $[\alpha]_D^{26}$ –26.5 (*c* 1.32 in $CHCl_3$, >99% *ee*). Daicel CHIRALCEL® OD-3, hexane 100%, 0.5 mL/min, 40 °C, *S* isomer: t_S = 31.15 min; for the racemic compound: *R* isomer: t_R = 25.53 min, *S* isomer: t_S = 30.67 min.

(+)-(*R*)-1-(*tert*-Butyl)-1-methyl-1,2,2,2-tetraphenyldisilane [(+)-(*R*)-3c].

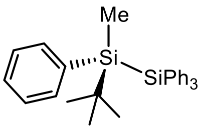

The reaction was conducted with (+)-(*S*)-**2c** (30.1 mg, 0.099 mmol, 97% *ee*) according to the typical procedure of Si–Si bond forming reaction for (+)-(*S*)-**3a**. The product (+)-(*R*)-**3c** was obtained in 80% yield with 97% *ee* (34.5 mg, 0.079 mmol, colorless viscous oil). The product was purified by silica-gel column chromatography with hexane/Et_2O eluent (100:0 to 99:1) followed by GPC and PTLC (hexane/ethyl acetate 96:4).

^{1}H NMR (400.5 MHz, $CDCl_3$, δ): 0.50 (s, 3H), 0.87 (s, 9H), 7.21 (t, *J* = 7.3 Hz, 2H), 7.25–7.39 (m, 12H), 7.43 (d, *J* = 7.2 Hz, 6H). ^{13}C NMR (100.3 MHz, $CDCl_3$, δ): –5.7 (CH_3), 19.5 (*C*), 28.5 (CH_3), 127.4 (*C*H), 127.8 (*C*H), 128.6 (*C*H), 128.9 (*C*H), 135.5 (*C*), 135.6 (*C*H), 136.4 (*C*H), 136.5 (*C*). ^{29}Si NMR (77.8 MHz, $CDCl_3$, δ): –21.9, –12.2. EI (*m*/*z*): $[M]^+$ calcd for $C_{29}H_{32}Si_2$: 436.2043, found: 436.2043. $[\alpha]_D^{23}$ +0.46 (*c* 2.18 in $CHCl_3$, 97% *ee*). Daicel CHIRALCEL® OD-3, hexane 100%, 0.5 mL/min, 40 °C, *S* isomer: t_S = 11.64 min (minor), *R* isomer: t_R = 13.04 min (major); for the racemic compound: *S* isomer: t_S = 10.40 min, *R* isomer: t_R = 11.52 min.

(–)-1-[3-(Benzyloxy)propyl]-1-cyclohexyl-1-methyl-2,2,2-triphenyldisilane [(–)-3d].

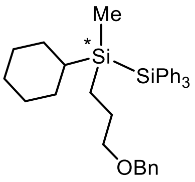

The reaction was conducted with (+)-**2d** (40.8 mg, 0.101 mmol, >99% *ee*) according to the typical procedure of Si–Si bond forming reaction for (+)-(*S*)-**3a**. The product (–)-**3d** was obtained in 72% yield with >99% *ee* (39.2 mg, 0.073 mmol, thick colorless oil). The product was purified by silica-gel column chromatography with hexane/Et_2O eluent (100:0 to 99:1) followed by GPC.

^{1}H NMR (399.0 MHz, $CDCl_3$, δ): 0.16 (s, 3H), 0.70–0.81 (m, 2H), 0.85–0.99 (m, 1H), 0.99–1.18 (m, 5H), 1.46–1.67 (m, 7H), 3.28 (t, *J* = 6.9 Hz, 2H), 4.38 (s, 2H), 7.25–7.29 (m, 3H), 7.30–7.39 (m, 11H), 7.44–7.49 (m, 6H). ^{13}C NMR (100.3 MHz, $CDCl_3$, δ): –6.1 (CH_3), 8.8 (CH_2), 24.8 (CH_2), 25.3 (*C*H), 26.8 (CH_2), 28.2 (CH_2), 28.3 (CH_2), 28.5 (CH_2), 28.8 (CH_2), 72.5 (CH_2), 73.2 (CH_2), 127.4 (*C*H), 127.6 (*C*H), 127.8 (*C*H), 128.3 (*C*H), 128.9 (*C*H), 135.9 (*C*), 136.1 (*C*H), 138.6 (*C*). ^{29}Si NMR (77.8 MHz, $CDCl_3$, δ): –20.8, –12.6. ESI (*m*/*z*): $[M+Na]^+$ calcd for $C_{35}H_{42}OSi_2Na$: 557.2666, found: 557.2657. $[\alpha]_D^{26}$ –3.5 (*c* 2.01 in $CHCl_3$, >99% *ee*). Daicel CHIRALPAK® IB N-3, hexane 100%, 0.5 mL/min, 40 °C, t_1 = 29.46 min (major), t_2 = 35.94 min (minor); for racemic compound: t_1 = 29.73 min, t_2 = 34.96 min.

(–)-1-Benzyl-1-cyclohexyl-1-methyl-2,2,2-triphenyldisilane [(–)-3e].

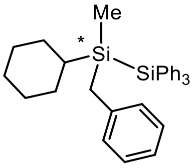

The reaction was conducted with (–)-**2e** (34.0 mg, 0.099 mmol, >99% *ee*) according to the typical procedure of Si–Si bond forming reaction for (+)-(*S*)-**3a**. The product (–)-**3e** was obtained in 78% yield with >99% *ee* (36.6 mg, 0.077 mmol, thick colorless oil). The product was purified by silica-gel column chromatography with hexane/Et_2O eluent (100:0 to 99:1).

^{1}H NMR (400.5 MHz, $CDCl_3$, δ): 0.13 (s, 3H), 0.80–0.94 (m, 1H), 0.96–1.20 (m, 5H), 1.53–1.73 (m, 5H), 2.28 (d, *J* = 14.0 Hz, 1H), 2.32, (d, *J* = 13.2 Hz, 1H), 6.63–6.72 (m, 2H), 6.98–7.13 (m, 3H), 7.25–7.40 (m, 15H). ^{13}C NMR (100.3 MHz, $CDCl_3$, δ): –5.6 (CH_3), 22.2 (CH_2), 25.2 (*C*H), 26.7 (CH_2), 28.1 (CH_2), 28.2 (CH_2), 28.5 (CH_2), 28.8 (CH_2), 124.0 (*C*H), 127.8 (*C*H), 128.1 (*C*H), 128.6 (*C*H), 128.9 (*C*H), 135.4 (*C*), 136.2 (*C*H), 139.9 (*C*). ^{29}Si NMR (77.8 MHz, $CDCl_3$, δ): –19.8, –12.1. EI (*m*/*z*): $[M]^+$ calcd for $C_{32}H_{36}Si_2$: 476.2356, found: 476.2350. $[\alpha]_D^{30}$ –2.5 (*c* 0.274 in $CHCl_3$, >99% *ee*). Daicel CHIRALCEL® OD-3×2, hexane 100%, 0.5 mL/min, 40 °C, t_2 = 40.88 min; for racemic compound: t_1 = 40.43 min, t_2 = 41.79 min.

(–)-1,2-Dibenzyl-1-cyclohexyl-1,2,2-trimethyldisilane [(–)-3e′].

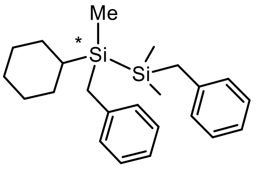

The reaction was conducted with (–)-**2e** (34.5 mg, 0.100 mmol, >99% *ee*) and benzylchlorodimethylsilane (37.1 mg, 0.201 mmol, 2.0 equiv) according to the typical procedure of Si–Si bond forming reaction for (+)-(*S*)-**3a**. The product (–)-**3e′** was obtained in 90% yield with >99% *ee* (33.0 mg, 0.090 mmol, colorless oil). The product was purified by silica-gel column chromatography with hexane/Et_2O eluent (100:0 to 99:1) followed by GPC.

^{1}H NMR (400.5 MHz, $CDCl_3$, δ): –0.064 (s, 3H), –0.062 (s, 3H), –0.04 (s, 3H), 0.70–0.82 (m, 1H), 1.05–1.29 (m, 5H), 1.58–1.80 (m, 5H), 1.98 (d, J = 14.0 Hz, 1H), 2.05 (d, J = 13.6 Hz, 1H), 2.13 (d, J = 13.2 Hz, 1H), 2.17 (d, J = 13.2 Hz, 1H), 6.90 (d, J = 6.9 Hz, 2H), 6.99 (d, J = 7.3 Hz, 2H), 7.02–7.09 (m, 2H), 7.15–7.24 (m, 4H). ^{13}C NMR (100.3 MHz, $CDCl_3$, δ): –7.2 (CH_3), –3.2 (CH_3), –3.1 (CH), 21.7 (CH_2), 24.9 (CH), 25.2 (CH_2), 26.9 (CH_2), 28.16 (CH_2), 28.18 (CH_2), 28.5 (CH_2), 28.8 (CH_2), 123.9 (CH), 124.0 (CH), 128.06 (CH), 128.13 (CH), 128.2 (CH), 128.3 (CH), 140.1 (C), 140.7 (C). ^{29}Si NMR (77.8 MHz, $CDCl_3$, δ): –17.2, –12.6. EI (*m*/*z*): $[M]^+$ calcd for $C_{23}H_{34}Si_2$: 366.2199, found: 366.2192. $[\alpha]_D^{23}$ –8.7 (*c* 1.03 in $CHCl_3$, >99% *ee*). Daicel CHIRALPAK® IB N-3, hexane 100%, 0.5 mL/min, 40 °C, t_1 = 14.13 min (minor), t_2 = 18.95 min (major); for racemic compound: t_1 = 13.33 min, t_2 = 18.32 min.

(+)-(*S*)-1-(Benzyloxy)-1-butyl-1,2,2,2-tetraphenyldisilane [(+)-(*S*)-3g].

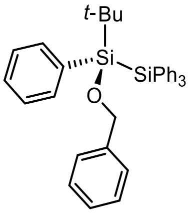

The reaction was conducted with (+)-(*R*)-**2g** (39.6 mg, 0.100 mmol, 95% *ee*) according to the typical procedure of Si–Si bond forming reaction for (+)-(*S*)-**3a**. The product (+)-(*S*)-**3g** was obtained in 83% yield with 95% *ee* (43.6 mg, 0.082 mmol, white solid). The product was purified by silica-gel column chromatography with hexane/Et_2O eluent (100:0 to 99:1) followed by GPC.

^{1}H NMR (400.5 MHz, $CDCl_3$, δ): 0.92 (s, 9H), 4.52 (d, J = 13.2 Hz, 1H), 4.65 (d, J = 13.2 Hz, 1H), 7.18–7.33(m, 13H), 7.23–7.42 (m, 6H), 7.48–7.54 (m, 6H).^{13}C NMR (100.3 MHz, $CDCl_3$, δ): 22.0 (C), 27.3 (CH_3), 125.9 (CH), 126.8 (CH), 127.6 (CH), 127.9 (CH), 126.2 (CH), 128.1 (CH), 129.17 (CH), 129.24 (CH), 134.6 (CH), 135.2 (C), 136.3 (C) 136.6 (CH), 141.0 (C). ^{29}Si NMR (77.8 MHz, $CDCl_3$, δ): –27.6, 4.6. FD (*m*/*z*): $[M]^+$ calcd for $C_{35}H_{36}OSi_2$: 528.2305, found: 528.2305. $[\alpha]_D^{26}$ +5.8 (*c* 1.03 in $CHCl_3$, 95% *ee*). Daicel CHIRALCEL® OD-3×2, hexane 100%, 0.5 mL/min, 40 °C, *S* isomer: t_S = 26.63 min (major), *R* isomer: t_R = 28.00 min (minor); for the racemic compound: t_S = 26.75 min, t_R = 27.84 min.

7. Procedure of Protonation Study.

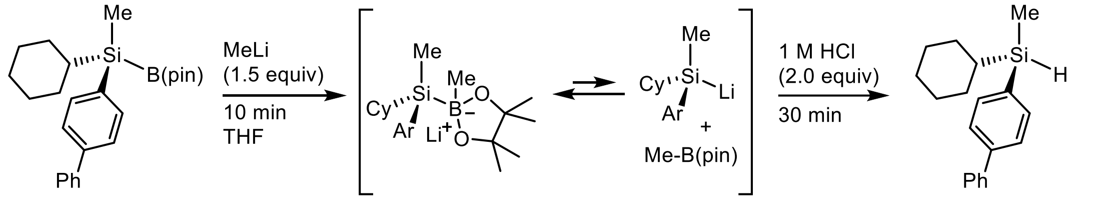

(–)-(*R*)-**2a**, >99% *ee* (–)-(*R*)-**1a**, >99% *ee*

(–)-(*R*)-[(1,1'-Biphenyl)-4-yl](cyclohexyl)methyl(4,4,5,5-tetramethyl-1,3,2-dioxaborolan-2-yl)silane [(–)-(*R*)-**2a**] (40.6 mg, 0.100 mmol, 1.0 equiv) was placed in a vial with a screw cap containing a Teflon®-coated rubber septum. After the vial was connected a manifold with nitrogen and vacuum line through a needle, it was evacuated and backfilled with nitrogen. This cycle was repeated three times. Dry THF (0.5 mL) was added to the vial through the rubber septum using a syringe, and the mixture was cooled to –78 °C. Then, MeLi (1.2 M in Et_2O, 125 μL, 0.150 mmol, 1.5 equiv) was added to the vial through the rubber septum using a syringe. After the mixture was stirred at –78 °C for 10 min, 200 μL aqueous HCl (1.0 M, 0.200 mmol, 2.0 equiv) was added dropwise to the vial at –78 °C. The mixture was allowed to warm to room temperature and stirred for 30 min. After that, the mixture was extracted with Et_2O three times and dried over Na_2SO_4. The organic layer was filtered and concentrated under reduced pressure. The crude product was purified by flash chromatography with hexane/Et_2O eluent (100:0 to 99:1) to give the corresponding product (–)-(*R*)-**1a** in 83% yield (23.1 mg, 0.083 mmol) with >99% *ee*.

Table S1. The Configurational Stability of Silicon-Stereogenic Optically Active Silyllithium under Various Conditions

entry	base	temp. (°C)	solvent	yield (%)	*ee* %	recovery (%)
1	MeLi	–78	THF	83	>99	-
2	MeLi	–40	THF	68	>99	26
3	MeLi	rt	THF	21	>99	17
4	*n*BuLi	–78	THF	54	>99	-
5	sBuLi	–78	THF	20	>99	-
6	*t*BuOLi	–78	THF	NR	-	95
7	*t*BuOK	–78	THF	8	>99	78
8	MeMgBr	–78	THF	6	95	50
9	MeLi	–78	toluene	32	>99	63
10	MeLi	–78	hexane	43	>99	51
11[b]	MeLi	–78	THF	87	>99	-

[a]Conditions: (–)-(*R*)-**2a** (0.1 mmol), base (0.15 mmol), and HCl aq. (1.0 M, 200 μL) in 0.5 mL solvent. The yields are isolated yields. The *ee* values were determined by HPLC with a chiral stationary phase. [b](–)-(*R*)-**2a** with methyllithium in THF at –78 °C for 2 h.

8. Typical Procedure of Palladium-Catalyzed Silylation of Aryl Bromides.

(–)-(*S*)-[(1,1'-Biphenyl)-4-yl](cyclohexyl)methyl(naphthalen-1-yl)silane [(–)-(*S*)-6a].

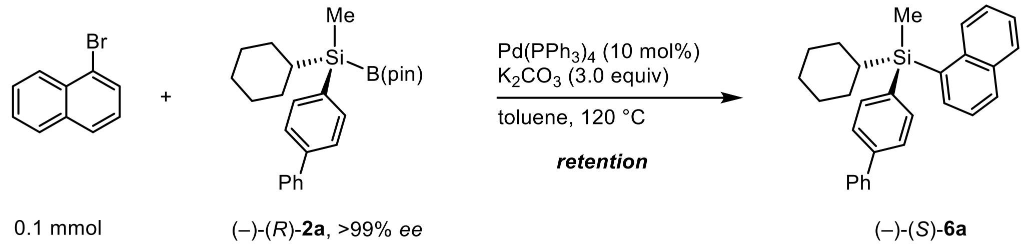

The palladium-catalyzed silylation was carried out according to the reported procedure.[9] A vial with a screw cap containing a Teflon®-coated rubber septum was charged with (–)-(*R*)-**2a** (61.1 mg, 0.150 mmol, 1.5 equiv), 1-bromonaphthalene (20.7 mg, 0.100 mmol, 1.0 equiv) and K_2CO_3 (41.5 mg, 0.300 mmol, 3.0 equiv) in air. The vial was placed in a glove box, and then $Pd(PPh_3)_4$ (11.6 mg, 0.010 mmol, 10 mol%) was added to the vial in the glove box under an argon atmosphere. After the reaction vial was removed from the glove box, and then dry toluene (0.5 mL) was added to the vial via a syringe. After the resulting mixture was stirred for 49 h at 120 °C, the mixture was directly filtered through a short silica-gel column with Et_2O as an eluent, then the resultant solution was concentrated under reduced pressure. The crude product was purified by silica-gel column chromatography with hexane/Et_2O eluent (100:0 to 99:1) and then purified by GPC to give the corresponding product (–)-(*S*)-**6a** in 42% yield (17.2 mg, 0.042 mmol, >99% *ee*) as a white solid.

^{1}H NMR (399.0 MHz, $CDCl_3$, δ): 0.69 (s, 3H), 1.13–1.39 (m, 5H), 1.50–1.60 (m, 1H), 1.65–1.80 (m, 4H), 1.93 (d, *J* = 11.0 Hz, 1H), 7.30–7.38 (m, 2H), 7.39–7.46 (m, 3H), 7.49 (dd, *J* = 8.2, 6.9 Hz, 1H), 7.54 (d, *J* = 8.2 Hz, 2H), 7.56–7.64 (m, 4H), 7.77–7.81 (m, 1H), 7.85 (d, *J* = 8.2 Hz, 1H), 7.90 (d, *J* = 8.2 Hz, 1H), 7.95 (d, *J* = 8.2 Hz, 1H). ^{13}C NMR (100.3 MHz, $CDCl_3$, δ): –4.6 (CH_3), 25.0 (*C*H), 26.9 (CH_2), 27.9 (CH_2), 28.19 (CH_2), 28.23 (CH_2), 125.0 (*C*H), 125.3 (*C*H), 125.5 (*C*H), 126.4 (*C*H), 127.1 (*C*H), 127.3 (*C*H), 128.7 (*C*H), 128.8 (*C*H), 128.9 (*C*H), 130.1 (*C*H), 133.4 (*C*), 134.3 (*C*), 135.1 (*C*H), 135.3 (*C*H), 136.2 (*C*), 137.3 (*C*), 141.0 (*C*), 141.4 (*C*). ^{29}Si NMR (77.8 MHz, $CDCl_3$, δ): –6.3. EI (*m*/*z*): $[M]^+$ calcd for $C_{29}H_{30}Si$: 406.2117, found: 406.2105. $[\alpha]_D^{26}$ –0.66 (*c* 1.06 in $CHCl_3$, >99% *ee*). Daicel CHIRALCEL® OD-3, hexane 100%, 0.5 mL/min, 40 °C, *S* isomer: t_S = 38.85 min; for the racemic compound: *R* isomer: t_R = 29.25 min, *S* isomer: t_S = 36.69 min.

9. Procedure of Palladium-Catalyzed Silylation of 1-(Bromomethyl)naphthalene.

(–)-(*S*)-[(1,1'-Biphenyl) -4-yl] (cyclohexyl)methyl(naphthalen-1-ylmethyl)silane [(–)-(*S*)-7a].

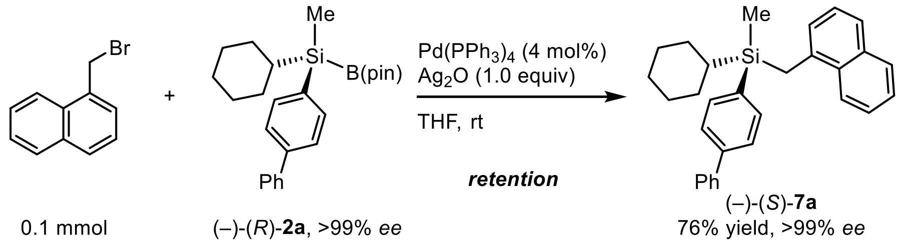

The palladium-catalyzed silylation of 1-(bromomethyl)naphthalene **6** was carried out according to the reported procedure.[10] A vial with a screw cap containing a Teflon®-coated rubber septum was charged with (–)-(*R*)-**2a** (73.1 mg, 0.180 mmol, 1.2 equiv), 1-(bromomethyl)naphthalene (33.2 mg, 0.150 mmol, 1.0 equiv) and Ag_2O (34.8 mg, 0.180 mmol, 1.2 equiv) in air. The vial was placed in a glove box, and then $Pd(PPh_3)_4$ (6.9 mg, 0.006 mmol, 4 mol%) was added to the vial in the glove box under an argon atmosphere. After closing the vial, the reaction vial was removed from the glove box, and then dry THF (1.0 mL) was added to the vial via a syringe. Then the resulting mixture was stirred at room temperature for 24 h. The mixture was directly filtered through a short silica-gel column with Et_2O as an eluent, then the resultant solution was concentrated under reduced pressure. The crude product was purified by silica-gel column chromatography with hexane/Et_2O eluent (100:0 to 99:1) and then purified by GPC to give the corresponding product (–)-(*S*)-**7a** in 76% yield (48.0 mg, 0.114 mmol, >99% *ee*) as a white solid.

^{1}H NMR (399.0 MHz, $CDCl_3$, δ): 0.06 (s, 3H), 0.94–1.07 (m, 1H), 1.07–1.34 (m, 5H), 1.62–1.90 (m, 5H), 2.79 (d, J = 14.2 Hz, 1H), 2.91 (d, J = 14.2 Hz, 1H), 7.08 (d, J = 7.3 Hz, 1H), 7.25–7.42 (m, 4H), 7.42–7.50 (m, 4H), 7.52 (d, J = 7.8 Hz, 2H), 7.59 (t, J = 8.0 Hz, 3H), 7.78 (d, J = 7.8 Hz, 1H), 7.82 (d, J = 8.7 Hz, 1H). ^{13}C NMR (100.3 MHz, $CDCl_3$, δ): –7.4 (CH_3), 19.0 (CH_2), 24.8 (CH), 26.8 (CH_2), 27.5 (CH_2), 27.7 (CH_2), 28.0 (CH_2), 124.6 (CH), 124.8 (CH), 124.9 (CH), 125.2 (CH), 125.4 (CH), 125.7 (CH), 126.2 (CH), 127.1 (CH), 127.3 (CH), 128.5 (CH), 128.7 (CH), 131.8 (C), 133.8 (C), 134.8 (CH), 135.5 (C), 136.5 (C), 141.0 (C), 141.5 (C). ^{29}Si NMR (77.8 MHz, $CDCl_3$, δ): –1.7. EI (*m*/*z*): $[M]^+$ calcd for $C_{30}H_{32}Si$: 420.2273, found: 420.2261. $[\alpha]_D^{30}$ –148.1 (*c* 0.204 in $CHCl_3$, >99% *ee*). Daicel CHIRALCEL® OD-3, IPA/hexane 5/95, 0.5 mL/min, 40 °C, *S* isomer: t_S = 9.84 min; for racemic compound: *S* isomer: t_S = 10.05 min, *R* isomer: t_R = 10.76 min.

10. Typical Procedure of Copper-Catalyzed Silyl Conjugate Addition.

(+)-Phenyl {3-[(1,1'-biphenyl)-4-yl](cyclohexyl)(methyl)silyl}propanoate [(+)-8a].

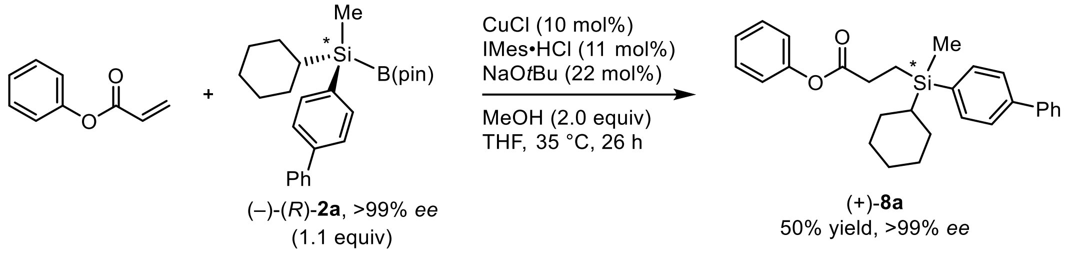

The copper-catalyzed silyl conjugate addition was carried out according to the reported procedure.[11] A vial with a screw cap containing a Teflon®-coated rubber septum was charged with (–)-(*R*)-**2a** (44.9 mg, 0.110 mmol, 1.1 equiv) in air. The vial was placed in a glove box, and then CuCl (1.0 mg, 0.010 mmol, 10 mol %), IMes·HCl (3.9 mg, 0.011 mmol, 11 mol %), *t*-BuONa (2.2 mg, 0.023 mmol, 23 mol %) were added to the vial in the glove box under an argon atmosphere. After closing the vial, the reaction vial was removed from the glove box, and then dry THF (0.3 mL) was added to the vial via a syringe. After the resulting mixture was stirred 10 min at 35 °C, phenyl acrylate (15.0 mg, 0.101 mmol, 1.0 equiv) and MeOH (6 μL, 0.212 mmol, 2.0 equiv) were added to the vial via a syringe, and the resulting mixture was stirred for 26 h at 35 °C. The mixture was directly filtered through Celite with Et_2O as an eluent, and then the resultant solution was concentrated under reduced pressure. The crude product was purified by silica-gel column chromatography with hexane/Et_2O eluent (100:0 to 99:1) and then purified by GPC and PTLC (hexane/ ethyl acetate 95:5) to give the corresponding product (+)-**8a** in 50% yield (21.8 mg, 0.050 mmol, >99% *ee*) as a viscous oil.

^{1}H NMR (400.5 MHz, $CDCl_3$, δ): 0.34 (s, 3H), 0.90–1.02 (m, 1H), 1.08–1.37 (m, 7H), 1.63–1.84 (m, 5H), 2.45–2.60 (m, 2H), 6.97–7.07 (m, 2H), 7.20 (t, *J* = 7.3 Hz, 1H), 7.30–7.40 (m, 3H), 7.45 (t, *J* = 7.6 Hz, 2H), 7.54–7.66 (m, 6H). ^{13}C NMR (100.3 MHz, $CDCl_3$, δ): –7.7 (CH_3), 7.3 (CH_2), 24.7 (*C*H), 26.8 (CH_2), 27.4 (CH_2), 27.5 (CH_2), 28.0 (CH_2), 29.1 (CH_2), 121.5 (*C*H), 125.6 (*C*H), 126.5 (*C*H), 127.1 (*C*H), 127.4 (*C*H), 128.8 (*C*H), 129.3 (*C*H), 134.7 (*C*H), 140.9 (*C*), 141.8 (*C*), 150.8 (*C*), 173.5 (*C*). ^{29}Si NMR (77.8 MHz, $CDCl_3$, δ): –0.8. ESI (*m*/*z*): $[M+Na]^+$ calcd for $C_{28}H_{32}O_2SiNa$: 451.2064, found: 451.2054. $[\alpha]_D^{23}$ +8.5 (*c* 1.01 in $CHCl_3$, >99% *ee*). Daicel CHIRALCEL® OD-3, IPA/hexane 1/99, 1.0 mL/min, 40 °C, t_1 = 10.15 min; for racemic compound: t_1 = 10.16 min, t_2 = 12.01 min.

11. Comparison with Other Catalytic Systems.

Comparison with rhodium-based catalyst

We screened the optimal catalyst system for the borylation of the chiral hydrosilanes. When rhodium-based catalyst was used as catalyst, (+)-**2d** was obtained in 67% yield with >99% *ee,* as shown in the following scheme. The absolute configuration of the product is the same as using the $Pt(PPh_3)_4$ catalyst.

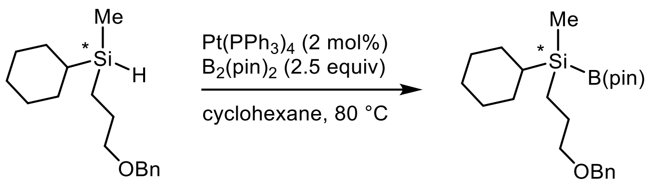

(–)-**1d** >99% *ee* → (+)-**2d** 67%, >99% *ee*

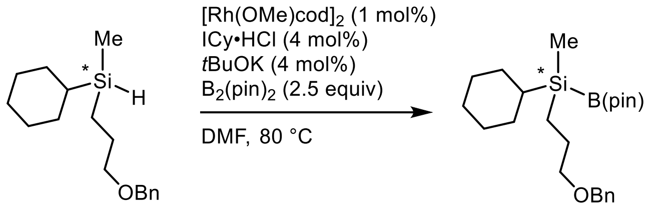

(–)-**1d** >99% *ee* → (+)-**2d** 67%, >99% *ee*

Comparison with Pt/C catalyst

When Pt/C was used as the catalyst, (–)-**2e** was obtained in 57% yield with >99% ee, but the reproducibility was low, although the absolute configuration of the product is the same as using the $Pt(PPh_3)_4$ catalyst. The reaction yield varied from 0% to 84% for a racemic substrate, even with other Pt/C catalysts that were purchased from different companies.

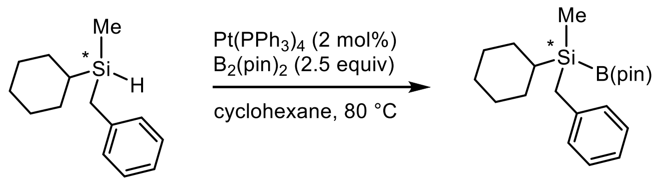

(+)-**1e** >99% *ee* → (–)-**2e** 71%, >99% *ee*

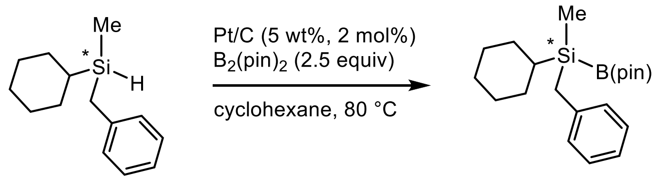

(+)-**1e** >99% *ee* → (–)-**2e** 57%, >99% *ee*

12. $^{11}B\{^1H\}$ and $^{29}Si\{^1H\}$ NMR Experiments.

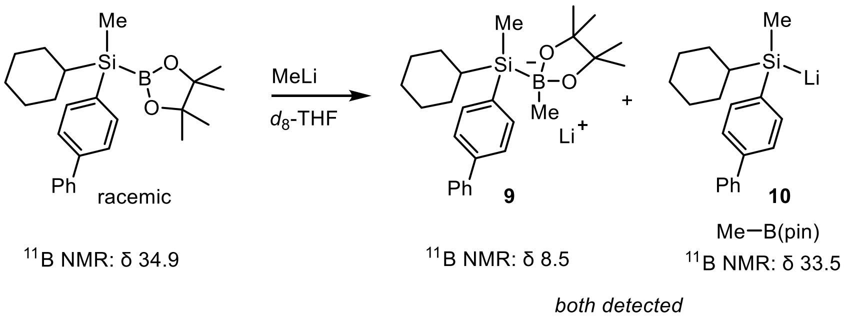

(±)-[(1,1'-Biphenyl)-4-yl](cyclohexyl)methyl(4,4,5,5-tetramethyl-1,3,2-dioxaborolan-2-yl)silane [(±)-**2a**] (40.6 mg, 0.100 mmol, 1.0 equiv) was placed in a vial with a screw cap containing a Teflon®-coated rubber septum. After the vial was connected a manifold with nitrogen and a vacuum line through a needle, it was evacuated and backfilled with nitrogen. This cycle was repeated three times. Dry THF-d_8 (0.5 mL) was added to the vial through the rubber septum using a syringe. Then TMS (14 µL, 0.100 mmol, 1.0 equiv) was added by a syringe, and the mixture was cooled to –78 °C. Then, MeLi (1.2 M in Et_2O, 125 µL, 0.150 mmol, 1.5 equiv) was added to the vial. After the solution was stirred at –78 °C for 20 min, the resulting mixture was transferred to an NMR tube as soon as possible under nitrogen and analyzed by $^{11}B\{^1H\}$ and $^{29}Si\{^1H\}$ NMR spectroscopy (JEOL JNM-ECX400P) at room temperature and $^{29}Si\{^1H\}$ NMR spectroscopy (JEOL ECA600) at –95 °C. $^{11}B\{^1H\}$ and $^{29}Si\{^1H\}$ NMR analysis revealed that ate complex **9**, and silyllithium **10** were generated in the reaction mixture.

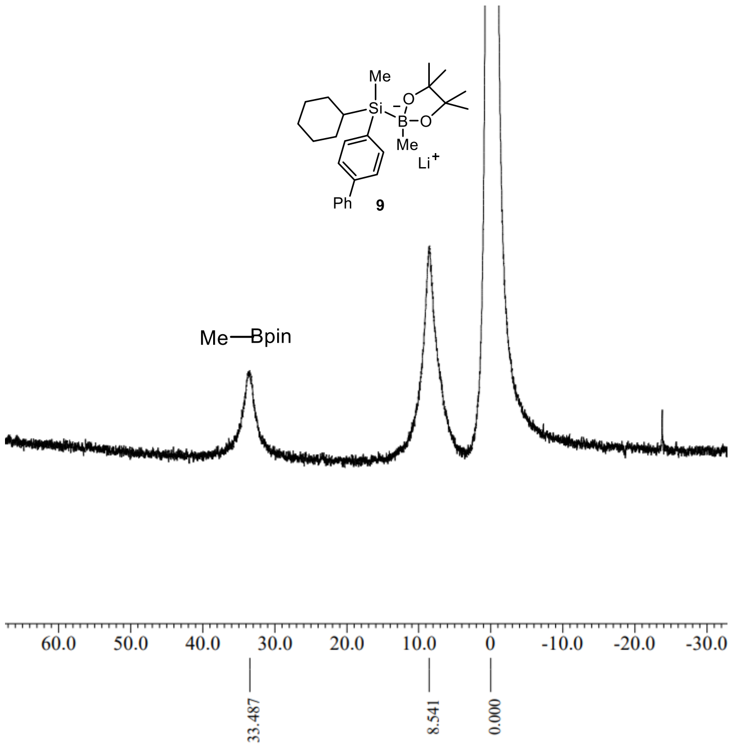

Figure S1. $^{11}B\{^1H\}$ NMR observation of (±)-{[(1,1'-biphenyl)-4-yl](cyclohexyl)(methyl)silyl}lithium **10** at room temperature. $BF_3{\cdot}OEt_2$ was used as an external standard to calibrate the $^{11}B\{^1H\}$ NMR spectra.

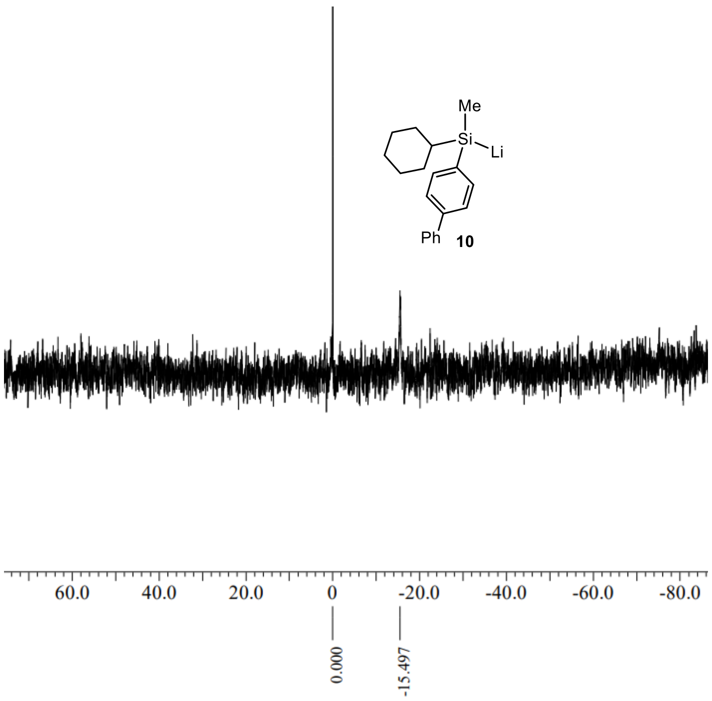

Figure S2. $^{29}Si\{^1H\}$ NMR observation of (±)-{[(1,1'-biphenyl)-4-yl](cyclohexyl)(methyl)silyl}lithium **10** at room temperature.

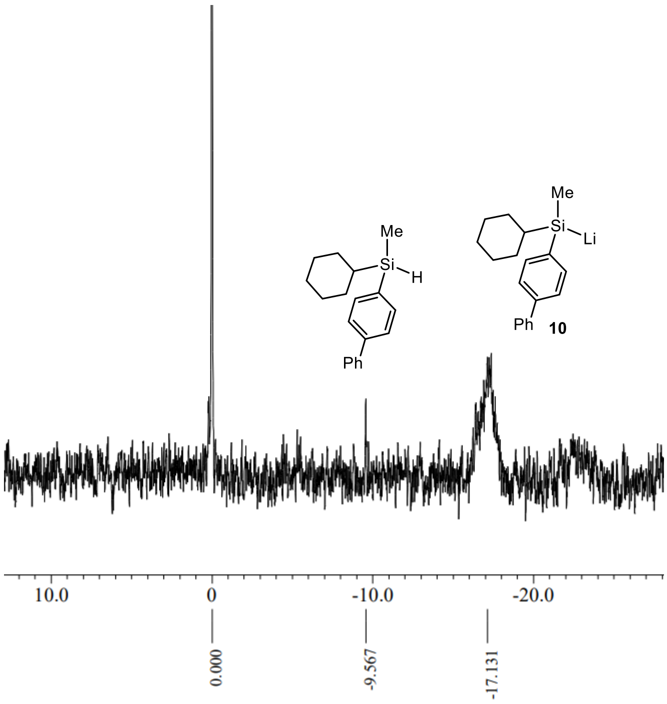

Figure S3. $^{29}Si\{^1H\}$ NMR observation of (±)-{[(1,1'-biphenyl)-4-yl](cyclohexyl)(methyl)silyl}lithium **10** at –95 °C.

13. Single Crystal Structure Analysis.

Molecular structure and X-ray crystallographic data of (–)-(*R*)-1a.

The stereochemistry of (–)-(*R*)-**1a** was determined by X-ray crystallographic analysis. Details are summarized in **Figure S4** and **Table S2.**

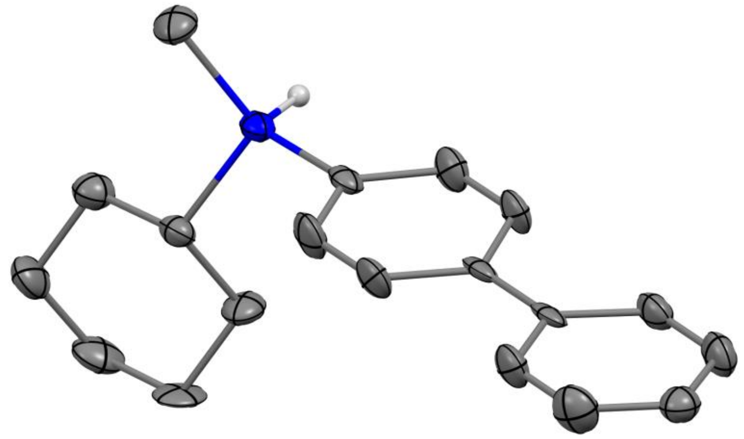

Figure S4. Molecular structure of (–)-(*R*)-**1a**.

Table S2. Summary of X-ray crystallographic data of (–)-(*R*)-**1a.**

CCDC Name	2193709
Empirical formula	$C_{19}H_{24}Si$
Formula weight	280.47
Temperature/K	123
Crystal system	orthorhombic
Space group	$P2_12_12_1$
a/Å	5.8032(2)
b/Å	7.7338(2)
c/Å	36.1780(10)
α/°	90
β/°	90
γ/°	90
Volume/Å^3	1623.70(8)
Z	4
ρ_{calc}g/cm^3	1.147
μ/mm^{-1}	1.156
F(000)	608.0
Crystal size/mm^3	0.25 × 0.12 × 0.03
Radiation	CuKα (λ = 1.54184)
2Θ range for data collection/°	4.886 to 153.176
Index ranges	$-4 \leq h \leq 6$, $-9 \leq k \leq 9$, $-44 \leq l \leq 44$
Reflections collected	3283
Independent reflections	3283 [R_{int} = ?,[a] R_{sigma} = 0.0079]
Data/restraints/parameters	3283/64/183
Goodness-of-fit on F^2	1.133
Final R indexes [I>=2σ (I)]	R_1 = 0.0650, wR_2 = 0.1878
Final R indexes [all data]	R_1 = 0.0656, wR_2 = 0.1882
Largest diff. peak/hole / e Å^{-3}	1.04/-0.43
Flack parameter	0.05(5)

[a]Not available because of the twin analyses.

Molecular structure and X-ray crystallographic data of (–)-(*R*)-2a.

The stereochemistry of (–)-(*R*)-**2a** was determined by X-ray crystallographic analysis. Details are summarized in **Figure S5** and **Table S3.**

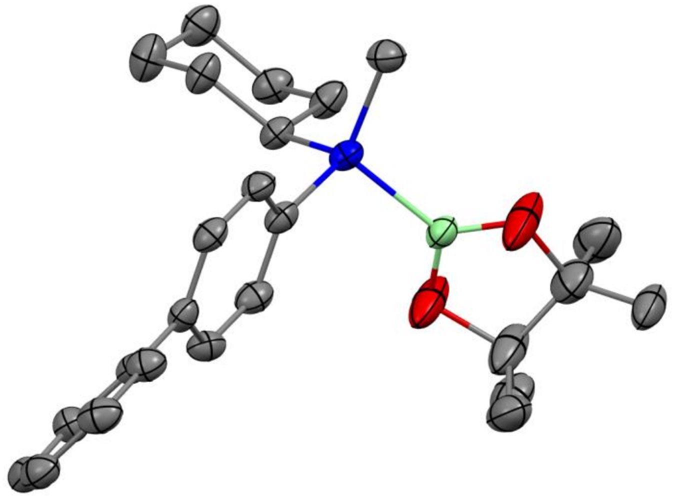

Figure S5. Molecular structure of (–)-(*R*)-**2a**.

Table S3. Summary of X-ray crystallographic data of (–)-(*R*)-**2a.**

CCDC Name	2193707
Empirical formula	$C_{25}H_{35}BO_2Si$
Formula weight	406.43
Temperature/K	123
Crystal system	orthorhombic
Space group	$P2_12_12_1$
a/Å	6.63825(12)
b/Å	12.60245(20)
c/Å	28.9806(5)
α/°	90
β/°	90
γ/°	90
Volume/Å^3	2424.47(7)
Z	4
ρ_{calc}g/cm^3	1.113
μ/mm^{-1}	0.972
F(000)	880.0
Crystal size/mm^3	0.2 × 0.17 × 0.03
Radiation	CuKα (λ = 1.54184)
2Θ range for data collection/°	6.1 to 152.78
Index ranges	$-8 \leq h \leq 7$, $-15 \leq k \leq 15$, $-36 \leq l \leq 29$
Reflections collected	12597
Independent reflections	4880 [R_{int} = 0.0474, R_{sigma} = 0.0390]
Data/restraints/parameters	4880/0/267
Goodness-of-fit on F^2	1.028
Final R indexes [I>=2σ (I)]	R_1 = 0.0833, wR_2 = 0.2160
Final R indexes [all data]	R_1 = 0.0855, wR_2 = 0.2187
Largest diff. peak/hole / e Å^{-3}	1.24/-0.70
Flack parameter	-0.02(2)

Molecular structure and X-ray crystallographic data of (+)-(*S*)-3a.

The stereochemistry of (+)-(*S*)-**3a** was determined by X-ray crystallographic analysis. Details are summarized in F**igure S6** and **Table S4**.

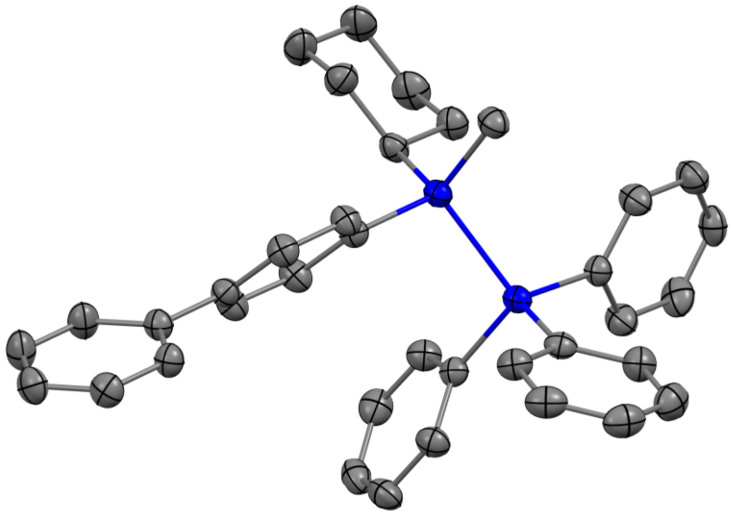

Figure S6. Molecular structure of (+)-(*S*)-**3a**.

Table S4. Summary of X-ray crystallographic data of (+)-(*S*)-**3a.**

CCDC Name	2193708
Empirical formula	$C_{37}H_{38}Si_2$
Formula weight	538.85
Temperature/K	123
Crystal system	triclinic
Space group	P1
a/Å	10.01360(10)
b/Å	10.9188(2)
c/Å	15.4908(2)
α/°	99.6450(10)
β/°	93.1920(10)
γ/°	112.4160(10)
Volume/Å^3	1530.26(4)
Z	2
ρ_{calc}g/cm^3	1.169
μ/mm^{-1}	1.214
F(000)	576.0
Crystal size/mm^3	0.35 × 0.35 × 0.35
Radiation	CuKα (λ = 1.54184)
2Θ range for data collection/°	5.838 to 152.756
Index ranges	$-12 \leq h \leq 12$, $-13 \leq k \leq 13$, $-19 \leq l \leq 19$
Reflections collected	38826
Independent reflections	11307 [R_{int} = 0.0244, R_{sigma} = 0.0169]
Data/restraints/parameters	11307/3/706
Goodness-of-fit on F^2	1.029
Final R indexes [I>=2σ (I)]	R_1 = 0.0281, wR_2 = 0.0771
Final R indexes [all data]	R_1 = 0.0282, wR_2 = 0.0772
Largest diff. peak/hole / e Å^{-3}	0.24/-0.21
Flack parameter	0.001(9)

Molecular structure and X-ray crystallographic data of (+)-(*R*)-1b.

The stereochemistry of (+)-(*R*)-**1b** was determined by X-ray crystallographic analysis. Details are summarized in **Figure S7** and **Table S5**.

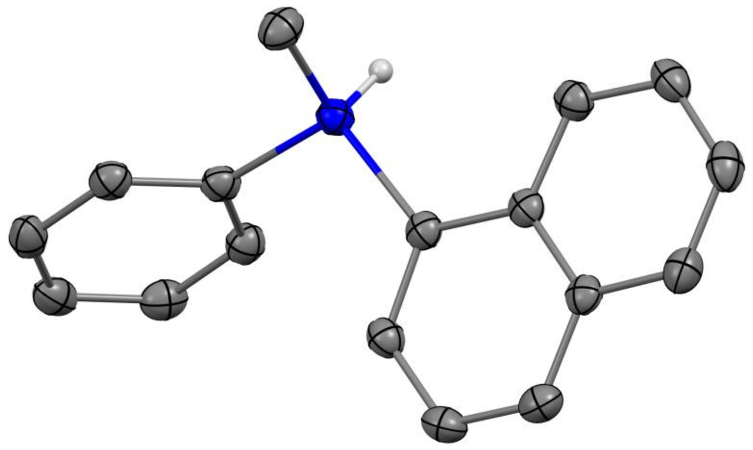

Figure S7. Molecular structure of (+)-(*R*)-**1b**.

Table S5. Summary of X-ray crystallographic data of (+)-(*R*)-**1b**.

CCDC Name	2193712
Empirical formula	$C_{17}H_{16}Si$
Formula weight	248.39
Temperature/K	123
Crystal system	orthorhombic
Space group	$P2_12_12_1$
a/Å	7.75780(10)
b/Å	8.75340(10)
c/Å	19.8761(3)
α/°	90
β/°	90
γ/°	90
Volume/Å^3	1349.73(3)
Z	4
ρ_{calc}g/cm^3	1.222
μ/mm^{-1}	1.336
F(000)	528.0
Crystal size/mm^3	0.2 × 0.2 × 0.1
Radiation	CuKα (λ = 1.54184)
2Θ range for data collection/°	8.898 to 143.54
Index ranges	$-8 \le h \le 9$, $-8 \le k \le 10$, $-24 \le l \le 24$
Reflections collected	6252
Independent reflections	2569 [R_{int} = 0.0176, R_{sigma} = 0.0175]
Data/restraints/parameters	2569/0/164
Goodness-of-fit on F^2	1.038
Final R indexes [I>=2σ (I)]	R_1 = 0.0272, wR_2 = 0.0752
Final R indexes [all data]	R_1 = 0.0274, wR_2 = 0.0754
Largest diff. peak/hole / e Å^{-3}	0.25/-0.29
Flack parameter	0.008(13)

Molecular structure and X-ray crystallographic data of (–)-(*R*)-1f.

The stereochemistry of (–)-(*R*)-**1f** was determined by X-ray crystallographic analysis. Details are summarized in **Figure S8** and **Table S6**.

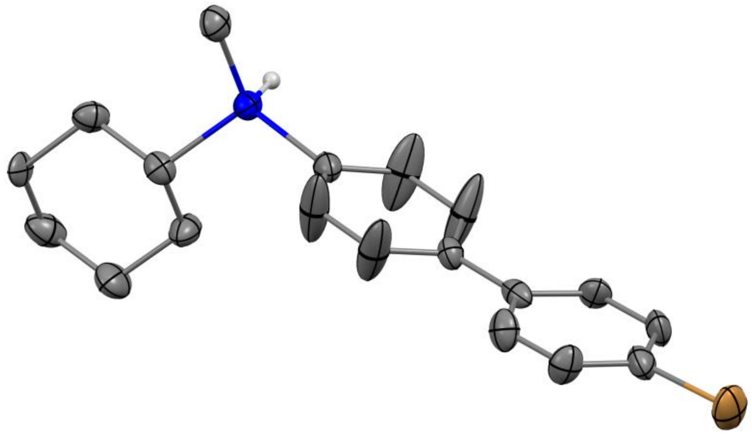

Figure S8. Molecular structure of (–)-(*R*)-**1f**.

Table S6. Summary of X-ray crystallographic data of (–)-(*R*)-**1f**.

CCDC Name	2193705
Empirical formula	$C_{19}H_{23}BrSi$
Formula weight	359.37
Temperature/K	123
Crystal system	monoclinic
Space group	$P2_1$
a/Å	7.7664(2)
b/Å	5.71330(10)
c/Å	19.7068(4)
α/°	90
β/°	93.184(2)
γ/°	90
Volume/Å^3	873.08(3)
Z	2
ρ_{calc}g/cm^3	1.367
μ/mm^{-1}	3.778
F(000)	372.0
Crystal size/mm^3	0.3 × 0.2 × 0.03
Radiation	CuKα (λ = 1.54184)
2Θ range for data collection/°	4.49 to 150.07
Index ranges	$-9 \le h \le 9$, $-6 \le k \le 6$, $-24 \le l \le 24$
Reflections collected	6015
Independent reflections	6015 [R_{int} = ?,[a] R_{sigma} = 0.0168]
Data/restraints/parameters	6015/1/192
Goodness-of-fit on F^2	1.178
Final R indexes [I>=2σ (I)]	R_1 = 0.0612, wR_2 = 0.1673
Final R indexes [all data]	R_1 = 0.0735, wR_2 = 0.2070
Largest diff. peak/hole / e Å^{-3}	0.67/-0.71
Flack parameter	-0.08(5)

[a]Not available because of the twin analyses.

Molecular structure and X-ray crystallographic data of (–)-(*R*)-2f.

The stereochemistry of (–)-(*R*)-**2f** was determined by X-ray crystallographic analysis. Details are summarized in **Figure S9** and **Table S7**.

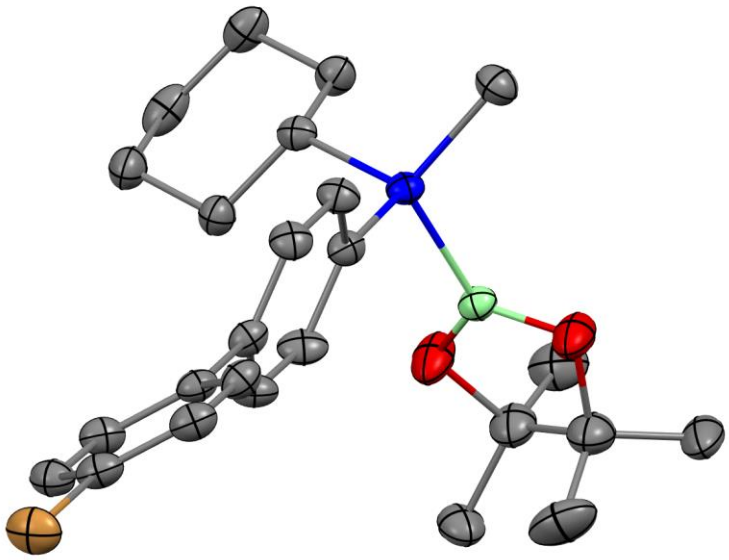

Figure S9. Molecular structure of (–)-(*R*)-**2f**.

Table S7. Summary of X-ray crystallographic data of (–)-(*R*)-**2f**.

CCDC Name	2193706
Empirical formula	$C_{25}H_{34}BBrO_2Si$
Formula weight	485.33
Temperature/K	123
Crystal system	orthorhombic
Space group	$P2_12_12_1$
a/Å	6.6570(2)
b/Å	15.3607(3)
c/Å	24.6653(5)
α/°	90
β/°	90
γ/°	90
Volume/Å^3	2522.18(10)
Z	4
ρ_{calc}g/cm^3	1.278
μ/mm^{-1}	2.805
F(000)	1016.0
Crystal size/mm^3	0.25 × 0.1 × 0.02
Radiation	CuKα (λ = 1.54184)
2Θ range for data collection/°	6.78 to 153.424
Index ranges	$-6 \leq h \leq 8$, $-14 \leq k \leq 19$, $-30 \leq l \leq 22$
Reflections collected	11172
Independent reflections	5040 [R_{int} = 0.0265, R_{sigma} = 0.0274]
Data/restraints/parameters	5040/0/276
Goodness-of-fit on F^2	1.061
Final R indexes [I>=2σ (I)]	R_1 = 0.0404, wR_2 = 0.1070
Final R indexes [all data]	R_1 = 0.0413, wR_2 = 0.1076
Largest diff. peak/hole / e Å^{-3}	1.18/-0.59
Flack parameter	-0.034(11)

Molecular structure and X-ray crystallographic data of (+)-(*S*)-3g.

The stereochemistry of (+)-(*S*)-**3g** was determined by X-ray crystallographic analysis. Details are summarized in **Figure S10** and **Table S8**.

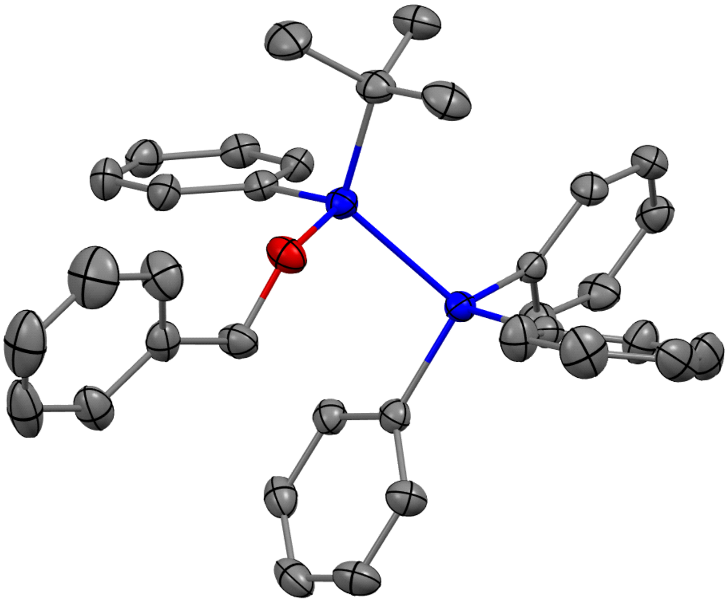

Figure S10. Molecular structure of (+)-(*S*)-**3g**.

Table S8. Summary of X-ray crystallographic data of (+)-(*S*)-**3g**.

CCDC Name	2244288
Empirical formula	$C_{35}H_{36}OSi_2$
Formula weight	528.82
Temperature/K	123
Crystal system	tetragonal
Space group	$P4_32_12$
a/Å	12.40117(6)
b/Å	12.40117(6)
c/Å	39.0559(2)
α/°	90
β/°	90
γ/°	90
Volume/Å^3	6006.36(6)
Z	8
ρ_{calc}g/cm^3	1.170
μ/mm^{-1}	1.254
F(000)	2256.0
Crystal size/mm^3	0.3 × 0.2 × 0.2
Radiation	CuKα (λ = 1.54184)
2Θ range for data collection/°	7.48 to 154.678
Index ranges	$-14 \leq h \leq 15$, $-13 \leq k \leq 14$, $-49 \leq l \leq 48$
Reflections collected	39359
Independent reflections	6297 [R_{int} = 0.0252, R_{sigma} = 0.0147]
Data/restraints/parameters	6297/0/347
Goodness-of-fit on F^2	1.065
Final R indexes [I>=2σ (I)]	R_1 = 0.0253, wR_2 = 0.0659
Final R indexes [all data]	R_1 = 0.0257, wR_2 = 0.0662
Largest diff. peak/hole / e Å^{-3}	0.22/-0.19
Flack parameter	-0.012(5)

Molecular structure and X-ray crystallographic data of (–)-(*S*)-6a.

The stereochemistry of (–)-(*S*)-**6a** was determined by X-ray crystallographic analysis. Details are summarized in **Figure S11** and **Table S9**.

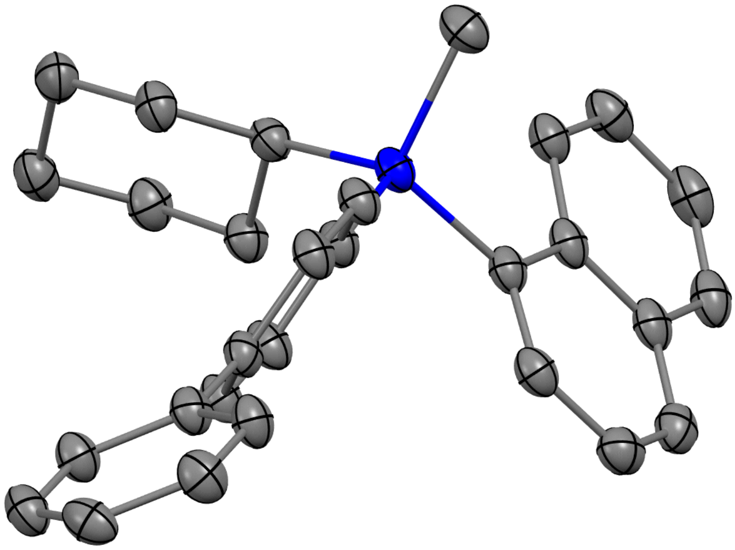

Figure S11. Molecular structure of (–)-(*S*)-**6a**.

Table S9. Summary of X-ray crystallographic data of (–)-(*S*)-**6a**.

CCDC Name	2193710
Empirical formula	$C_{29}H_{30}Si$
Formula weight	406.62
Temperature/K	123
Crystal system	orthorhombic
Space group	$P2_12_12_1$
a/Å	9.5189(2)
b/Å	11.6681(2)
c/Å	20.2169(4)
α/°	90
β/°	90
γ/°	90
Volume/Å^3	2245.44(8)
Z	4
ρ_{calc}g/cm^3	1.203
μ/mm^{-1}	0.997
F(000)	872.0
Crystal size/mm^3	0.4 × 0.2 × 0.2
Radiation	CuKα (λ = 1.54184)
2Θ range for data collection/°	8.748 to 143.848
Index ranges	$-9 \le h \le 11$, $-13 \le k \le 11$, $-23 \le l \le 24$
Reflections collected	9509
Independent reflections	4289 [R_{int} = 0.0376, R_{sigma} = 0.0327]
Data/restraints/parameters	4289/0/272
Goodness-of-fit on F^2	1.034
Final R indexes [I>=2σ (I)]	R_1 = 0.0460, wR_2 = 0.1242
Final R indexes [all data]	R_1 = 0.0463, wR_2 = 0.1245
Largest diff. peak/hole / e Å^{-3}	0.51/-0.33
Flack parameter	-0.01(2)

Molecular structure and X-ray crystallographic data of (–)-(*S*)-7a.

The stereochemistry of (–)-(*S*)-**7a** was determined by X-ray crystallographic analysis. Details are summarized in **Figure S12** and **Table S10**.

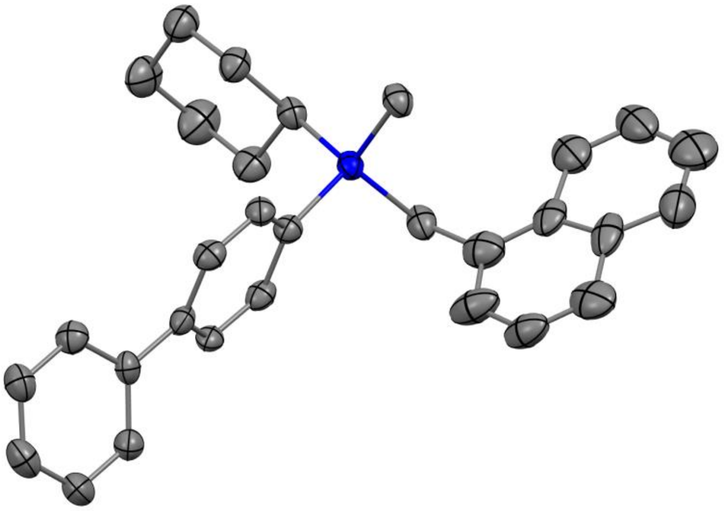

Figure S12. Molecular structure of (–)-(*S*)-**7a**.

Table S10. Summary of X-ray crystallographic data of (–)-(*S*)-**7a**.

CCDC Name	2193711
Empirical formula	$C_{30}H_{32}Si$
Formula weight	420.64
Temperature/K	123
Crystal system	monoclinic
Space group	$P2_1$
a/Å	10.1268(3)
b/Å	12.0884(3)
c/Å	10.3355(3)
α/°	90
β/°	108.488(3)
γ/°	90
Volume/Å^3	1199.94(6)
Z	2
ρ_{calc}g/cm^3	1.164
μ/mm^{-1}	0.948
F(000)	452.0
Crystal size/mm^3	0.4 × 0.3 × 0.2
Radiation	CuKα (λ = 1.54184)
2Θ range for data collection/°	9.022 to 143.55
Index ranges	$-12 \leq h \leq 12$, $-14 \leq k \leq 13$, $-12 \leq l \leq 10$
Reflections collected	11428
Independent reflections	4353 [R_{int} = 0.0179, R_{sigma} = 0.0166]
Data/restraints/parameters	4353/103/281
Goodness-of-fit on F^2	1.024
Final R indexes [I>=2σ (I)]	R_1 = 0.0780, wR_2 = 0.2106
Final R indexes [all data]	R_1 = 0.0783, wR_2 = 0.2109
Largest diff. peak/hole / e Å^{-3}	1.31/-0.43
Flack parameter	0.018(16)

14. DFT Calculations for the Stereospecific Reaction between Silylborane and MeLi followed by Protonation.

Computational Methods

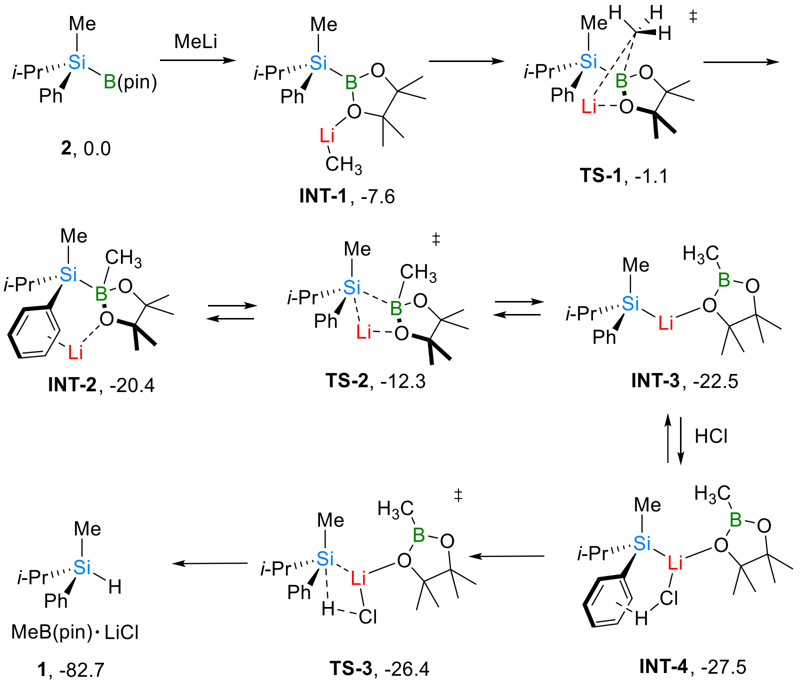

All of the calculations were performed at the DFT level of theory with the B3LYP hybrid functional[12] as implemented in Gaussian 16.[13] To describe the dispersion properly, an explicit dispersion correction term developed by Grimme and co-workers,[14] was also employed in the DFT calculations. The Def2SVP basis set[15] was used for all the atoms involved in this study during both the geometry optimization and the single-point calculation processes. To describe the solvation environment in which the molecule stays, the implicit solvation model, IEF-PCM,[16] is applied to all the calculations involved in this study with cyclohexane (ε=2.0165) used as the solvent. All the minima and transition states were fully optimized without any constraints. The free energies were computed at 298.15 K and 1 atm.

Cartesian Coordinates for Optimized Structures

2

E_0 = -1090.29185421 a.u. (G_{298K} = -1089.94151375 a.u.)

0 1

Si	-0.896807292072	-0.194113920890	1.044927017693
C	-2.415817165068	-0.737466288865	0.048953877179

H	-2.224143997941	-0.689271687569	-1.035410625338
H	-2.701230319001	-1.772695360190	0.295737187580
H	-3.274241208913	-0.081833634788	0.269380380925
C	-0.472037936609	1.622995350437	0.600058800970
H	-1.384782062279	2.190947528359	0.865078358201
C	-0.189226688556	1.824339244665	-0.895928742601
C	0.684529685398	2.152137287438	1.463064230481
H	0.451291619145	2.094360033105	2.539004083922
H	1.609621271867	1.575612729438	1.289202444874
H	0.908503423528	3.208515246398	1.230593032815
H	0.702389571659	1.256534301119	-1.212041265040
H	0.002109429245	2.887607883366	-1.126671872004
H	-1.031381347678	1.497382164793	-1.527641466549
C	0.582040132220	-1.296809480483	0.602570401165
C	1.500955090413	-1.722175257700	1.582930487832
C	0.833339334274	-1.669762043934	-0.733664363487
C	2.626268916713	-2.481109381959	1.242520316964
C	1.955540466128	-2.429168006034	-1.079805458497
C	2.857434812565	-2.835363260666	-0.090348844376
H	1.325864671752	-1.470427989432	2.632103382551
H	0.141548348826	-1.365437162373	-1.525017974256
H	3.323393961955	-2.800998245059	2.022159215476
H	2.127027128295	-2.705622174221	-2.123929539110
H	3.735594595429	-3.429488943848	-0.357717781215
B	-1.266557932228	-0.237782227925	3.038098737781
O	-0.810459436872	-1.181368376740	3.921748781835
C	-1.427409398595	-0.928694554328	5.217037266985
C	-1.834442190817	0.589482323086	5.097372643316
O	-1.984979556213	0.752113133845	3.658072628756
C	-3.147836957140	0.960979237001	5.774731769236
H	-3.993347430030	0.401708640002	5.354337240719
H	-3.095021005063	0.762556335838	6.856542407033
H	-3.347433681872	2.033415834416	5.631530134968
C	-0.721683964227	1.549228084992	5.529679146339
H	-0.580275021874	1.541527794044	6.620420069426
H	0.233038055431	1.290774996465	5.048591909036
H	-0.992031164399	2.569048450416	5.219007234005
C	-2.629310641895	-1.871933245706	5.321285814018
H	-3.371832101399	-1.656140628943	4.539186227222
H	-2.282009222347	-2.906350071045	5.183586164500
H	-3.119443479591	-1.795090425903	6.302973065993
C	-0.412949864227	-1.241097199507	6.310464708068
H	-0.817006245888	-0.976526897447	7.299982413264
H	-0.186883265475	-2.317800266646	6.308403528153

H	0.528775524541	-0.698476116466	6.157918892131

CH_3Li

E_0 = -47.38879819 a.u. (G_{298K} = -47.37714012 a.u.)

0 1

C	-1.156736712724	-0.000040013068	-1.737775761477
H	-2.171624255472	-0.007798200596	-1.283624544757
H	-0.641474424818	-0.874840579436	-1.284233817431
H	-0.655728702389	0.883880597697	-1.285891442370
Li	-1.156320482304	-0.001202054596	-3.764296968866

HCl

E_0 = -460.67127742 a.u. (G_{298K} = -460.68180324 a.u.)

0 1

H	-0.286261943684	-1.284257638769	0.251744610307
Cl	-0.286261943679	-1.284257638780	1.542468635751

INT-1

E_0 = -1137.71163636 a.u. (G_{298K} = -1137.33079936 a.u.)

0 1

Si	1.080276000000	-1.115608000000	0.750581000000
C	1.380853000000	-1.258010000000	2.612373000000
H	2.434854000000	-1.494716000000	2.830974000000
H	1.127426000000	-0.320539000000	3.132804000000
H	0.759718000000	-2.063063000000	3.037125000000
C	1.527541000000	-2.761905000000	-0.111789000000
H	0.887834000000	-3.512094000000	0.392445000000
C	2.998354000000	-3.150368000000	0.106121000000
C	1.164634000000	-2.758135000000	-1.605403000000
H	0.102068000000	-2.514861000000	-1.771802000000
H	1.770321000000	-2.023585000000	-2.162645000000
H	1.353337000000	-3.745392000000	-2.062100000000
H	3.676940000000	-2.406033000000	-0.344644000000
H	3.224956000000	-4.125506000000	-0.359737000000
H	3.255801000000	-3.229160000000	1.174766000000
C	2.115370000000	0.315246000000	0.044526000000
C	1.859923000000	0.805666000000	-1.256454000000
C	3.147774000000	0.931449000000	0.779651000000
C	2.603035000000	1.860483000000	-1.798047000000
C	3.893750000000	1.987048000000	0.243076000000
C	3.623101000000	2.454107000000	-1.046463000000
H	1.062208000000	0.360887000000	-1.859659000000
H	3.375162000000	0.588361000000	1.792763000000
H	2.382605000000	2.222743000000	-2.805545000000

H	4.685231000000	2.451155000000	0.837290000000
H	4.199996000000	3.283448000000	-1.463135000000
B	-0.847368000000	-0.631170000000	0.343895000000
O	-1.210777000000	0.673621000000	0.042426000000
C	-2.687123000000	0.739204000000	-0.008780000000
C	-3.050540000000	-0.768769000000	-0.276930000000
O	-1.907369000000	-1.478711000000	0.292422000000
C	-4.317432000000	-1.249397000000	0.418271000000
H	-4.240921000000	-1.156382000000	1.508878000000
H	-5.187876000000	-0.669935000000	0.074511000000
H	-4.491860000000	-2.308501000000	0.177990000000
C	-3.083219000000	-1.130884000000	-1.763944000000
H	-3.955353000000	-0.688926000000	-2.266782000000
H	-2.171843000000	-0.790039000000	-2.276980000000
H	-3.139432000000	-2.224734000000	-1.862216000000
C	-3.143208000000	1.255058000000	1.356234000000
H	-2.848944000000	0.566751000000	2.162310000000
H	-2.669420000000	2.230990000000	1.539487000000
H	-4.235005000000	1.382178000000	1.383786000000
C	-3.094086000000	1.709201000000	-1.109662000000
H	-4.187741000000	1.699461000000	-1.235081000000
H	-2.783091000000	2.727385000000	-0.833460000000
H	-2.629333000000	1.455023000000	-2.071133000000
C	-0.725847000000	4.165106000000	0.858325000000
H	-1.656237000000	4.536825000000	0.370263000000
H	-0.958094000000	4.220220000000	1.947125000000
H	0.009801000000	4.984654000000	0.696784000000
Li	-0.124082000000	2.302623000000	0.254198000000

TS-1

E_0 = -1137.70538116 a.u. (G_{298K} = -1137.32039983 a.u.)

0 1

Si	-0.950588000000	0.991890000000	0.651220000000
C	-1.354989000000	1.536453000000	2.411206000000
H	-2.370767000000	1.957154000000	2.485948000000
H	-1.266549000000	0.692076000000	3.109157000000
H	-0.642233000000	2.315783000000	2.726947000000
C	-1.077333000000	2.535224000000	-0.494706000000
H	-0.307260000000	3.219393000000	-0.086831000000
C	-2.441692000000	3.235498000000	-0.401185000000
C	-0.713286000000	2.236796000000	-1.957431000000
H	0.253796000000	1.715403000000	-2.051249000000
H	-1.476461000000	1.600045000000	-2.435184000000
H	-0.650144000000	3.165408000000	-2.552284000000

H	-3.252882000000	2.571706000000	-0.747475000000
H	-2.471183000000	4.143310000000	-1.030585000000
H	-2.683049000000	3.542613000000	0.628938000000
C	-2.215595000000	-0.306862000000	0.047806000000
C	-2.052666000000	-0.954257000000	-1.199207000000
C	-3.344266000000	-0.659996000000	0.815784000000
C	-2.971065000000	-1.910799000000	-1.653226000000
C	-4.266664000000	-1.609537000000	0.365204000000
C	-4.082211000000	-2.239752000000	-0.870823000000
H	-1.190593000000	-0.711379000000	-1.827643000000
H	-3.503507000000	-0.190502000000	1.790210000000
H	-2.820316000000	-2.393782000000	-2.622796000000
H	-5.132336000000	-1.863300000000	0.983182000000
H	-4.802116000000	-2.983643000000	-1.222157000000
B	0.898070000000	0.202152000000	0.448696000000
O	1.165501000000	-0.810836000000	-0.498220000000
C	2.613486000000	-1.020155000000	-0.556595000000
C	3.168881000000	0.321531000000	0.073769000000
O	2.055621000000	0.802641000000	0.863582000000
C	4.372769000000	0.141339000000	0.993621000000
H	4.135475000000	-0.508521000000	1.845105000000
H	5.226301000000	-0.285474000000	0.444461000000
H	4.675719000000	1.120917000000	1.391859000000
C	3.463477000000	1.405355000000	-0.969182000000
H	4.338901000000	1.152927000000	-1.585336000000
H	2.600410000000	1.566473000000	-1.630853000000
H	3.665277000000	2.351240000000	-0.445853000000
C	2.949596000000	-2.261149000000	0.273937000000
H	2.642810000000	-2.138375000000	1.320472000000
H	2.419883000000	-3.134080000000	-0.141429000000
H	4.025773000000	-2.482000000000	0.236020000000
C	2.989641000000	-1.247525000000	-2.016633000000
H	4.082504000000	-1.315479000000	-2.129113000000
H	2.551828000000	-2.193628000000	-2.369838000000
H	2.617955000000	-0.441627000000	-2.661841000000
C	0.247669000000	-1.602482000000	2.483971000000
H	0.686008000000	-2.589907000000	2.763088000000
H	0.932874000000	-0.854926000000	2.927653000000
H	-0.695355000000	-1.527315000000	3.063061000000
Li	-0.085455000000	-1.976753000000	0.487686000000

INT-2

E_0 = -1137.73993535 a.u. (G_{298K} = -1137.35123221 a.u.)

0 1

Si	-0.835871157113	1.029315950976	0.607404525985
C	-1.453048451355	1.845121322803	2.215549103563
H	-2.441483451973	2.322024607623	2.111504265187
H	-1.511955466627	1.109525341970	3.033828053827
H	-0.734878262739	2.622067089358	2.527725058088
C	-0.735345760949	2.425901398416	-0.726079048366
H	0.197266774100	2.963212266289	-0.468106384123
C	-1.894690812107	3.431943329675	-0.636386304590
C	-0.597849407687	1.910290882535	-2.168103959443
H	0.173261541272	1.130130806206	-2.269816706104
H	-1.548773276084	1.480140711799	-2.523391544113
H	-0.336621923269	2.729329260018	-2.861958852520
H	-2.867135803195	2.937473619255	-0.807997303658
H	-1.794569074851	4.225538707950	-1.399735534952
H	-1.944671261886	3.926985173610	0.345762632474
C	-2.227612314304	-0.200875656619	0.063630404048
C	-2.120001812779	-0.956764201580	-1.131907528102
C	-3.324485578830	-0.507609675777	0.899345167203
C	-3.048550616045	-1.953749313582	-1.470823461639
C	-4.253816019722	-1.500380090951	0.569665836416
C	-4.119888820559	-2.232088308437	-0.615847639773
H	-1.299643742865	-0.753770655279	-1.827394040877
H	-3.458825753521	0.042131138459	1.834667597311
H	-2.937588266317	-2.504111779311	-2.409705716727
H	-5.090423208156	-1.705424945288	1.243761945697
H	-4.847020445143	-3.006327859757	-0.873670114763
B	0.816867173830	-0.222102145391	0.989448355309
O	1.096465494238	-1.047211892982	-0.293717132532
C	2.508147114908	-1.068771850672	-0.533416226121
C	2.998908001018	0.248852782347	0.193498325443
O	2.107587767207	0.377024546598	1.284931971862
C	4.426987873431	0.159023065064	0.738537234286
H	4.510834643593	-0.613049576799	1.514406155179
H	5.149773139219	-0.059394475852	-0.064105022731
H	4.703729142610	1.121310191711	1.195826916263
C	2.890230989735	1.489972909758	-0.708315615678
H	3.652375279393	1.497762747623	-1.502672539032
H	1.901714718139	1.556277593903	-1.175183309262
H	3.029259932378	2.384275310913	-0.082630435533
C	3.102251095700	-2.338085917354	0.097447479196
H	2.980961583939	-2.330604813826	1.188823614099
H	2.580172730903	-3.222389786358	-0.304775430462
H	4.172148779474	-2.450295729735	-0.134062965174
C	2.750178981821	-1.090304700353	-2.042476588812

H	3.822615111095	-0.985573292022	-2.270426294323
H	2.406671723006	-2.047405886331	-2.467622447815
H	2.204913947656	-0.282122612594	-2.546773485708
C	0.360582400272	-1.230208617634	2.220467088798
H	1.139075759336	-1.985667041763	2.433707760233
H	0.236619001772	-0.636448113564	3.141092474544
H	-0.610316700111	-1.767301850520	2.121393727592
Li	-0.340460311862	-2.077068964532	0.042439940332

TS-2

E_0 = -1137.72611764 a.u. (G_{298K} = -1137.33824496 a.u.)

0 1

Si	-1.190840000000	1.042709000000	0.565918000000
C	-2.030872000000	2.045114000000	1.987638000000
H	-3.076039000000	2.322087000000	1.762946000000
H	-2.034056000000	1.498654000000	2.946736000000
H	-1.464458000000	2.977160000000	2.156013000000
C	-1.356553000000	2.254886000000	-0.950147000000
H	-0.937377000000	3.196743000000	-0.543278000000
C	-2.820335000000	2.520186000000	-1.335940000000
C	-0.528423000000	1.889172000000	-2.190508000000
H	0.533029000000	1.744326000000	-1.946277000000
H	-0.894019000000	0.959330000000	-2.659762000000
H	-0.599912000000	2.678020000000	-2.962643000000
H	-3.293246000000	1.606207000000	-1.733904000000
H	-2.897702000000	3.297982000000	-2.118994000000
H	-3.427514000000	2.854263000000	-0.479505000000
C	-2.403232000000	-0.424138000000	0.234118000000
C	-3.334191000000	-0.869571000000	1.201467000000
C	-2.314526000000	-1.196048000000	-0.949739000000
C	-4.120157000000	-2.011575000000	1.005791000000
C	-3.102829000000	-2.331793000000	-1.158065000000
C	-4.009250000000	-2.751119000000	-0.176606000000
H	-3.460360000000	-0.304302000000	2.130343000000
H	-1.615395000000	-0.897372000000	-1.736100000000
H	-4.832657000000	-2.319883000000	1.777263000000
H	-3.011113000000	-2.893566000000	-2.092695000000
H	-4.625191000000	-3.640601000000	-0.334411000000
Li	0.245703000000	-0.684174000000	1.720620000000
B	1.824065000000	0.994144000000	0.728211000000
O	2.024207000000	-0.282766000000	1.367155000000
O	2.324374000000	0.928269000000	-0.558494000000
C	2.711041000000	-0.420261000000	-0.881452000000
C	1.562224000000	-1.092097000000	-1.641129000000

H	0.643539000000	-1.133303000000	-1.040055000000
H	1.334220000000	-0.499714000000	-2.537732000000
H	1.828662000000	-2.111567000000	-1.956307000000
H	4.739534000000	0.256154000000	-1.321642000000
C	3.953207000000	-0.366539000000	-1.767069000000
H	3.689632000000	0.070878000000	-2.741327000000
H	4.353594000000	-1.377510000000	-1.941345000000
C	2.961457000000	-1.041946000000	0.546048000000
C	4.360187000000	-0.746036000000	1.094383000000
H	5.131923000000	-1.327481000000	0.569436000000
H	4.389019000000	-1.011190000000	2.161560000000
H	4.601616000000	0.322497000000	1.001633000000
C	2.657043000000	-2.531431000000	0.666097000000
H	2.845773000000	-2.874568000000	1.695087000000
H	1.616701000000	-2.772685000000	0.402583000000
H	3.311145000000	-3.107439000000	-0.005947000000
C	1.781630000000	2.355824000000	1.519275000000
H	1.282638000000	3.143314000000	0.933990000000
H	1.273614000000	2.276575000000	2.491388000000
H	2.821561000000	2.691083000000	1.702927000000

INT-3

E_0 = -1137.73760168 a.u. (G_{298K} = -1137.35446554 a.u.)

0 1

Si	-1.295821454919	-0.108426700560	0.173115349983
C	-0.984203717430	-1.013681562237	1.857098982224
H	-1.647698729056	-0.682924584875	2.676089790851
H	-1.116216279216	-2.104377320720	1.745902060075
H	0.054858433046	-0.848213332650	2.192979544510
C	-1.224512612190	1.789655351879	0.685945473752
H	-0.196751288040	1.925474462939	1.080627437627
C	-2.208055945091	2.177527294727	1.798911361628
C	-1.390167902991	2.726678570212	-0.518776043498
H	-0.661695469023	2.508851286517	-1.319402055440
H	-2.396602972804	2.626695520428	-0.961146877246
H	-1.267005172159	3.789589124211	-0.236543962560
H	-3.251049791519	2.034871602701	1.468085836808
H	-2.098968652928	3.239365284270	2.092805844401
H	-2.069359832721	1.571150120804	2.708425889788
C	-3.176462956233	-0.379179836862	-0.170166587307
C	-4.060393331815	-0.984637724541	0.746904874957
C	-3.736121554955	0.048309466023	-1.393918918285
C	-5.421751060497	-1.151881989336	0.463487815377
C	-5.096245803572	-0.100180938128	-1.683707442541

C	-5.948157624904	-0.706557980810	-0.753144910605
H	-3.683649582249	-1.334028870042	1.712682257400
H	-3.085670602480	0.506839314713	-2.147734294307
H	-6.076587968771	-1.629115845388	1.199288325585
H	-5.493401463043	0.250913440331	-2.641226679341
H	-7.011295464569	-0.833419987896	-0.976367183890
Li	0.462072078880	-0.745039376266	-1.536930781203
B	2.688119273064	-1.743060327928	-0.028842388321
O	2.313653670737	-0.825515548637	-1.018896722820
O	3.634605782686	-1.226207400709	0.795429937161
C	3.765919626518	0.197186519898	0.528881625440
C	2.853791705400	0.918101374900	1.524823496486
H	1.799446363895	0.648554586080	1.368483652610
H	3.134696444302	0.616180892031	2.543943041795
H	2.951369652052	2.010183354815	1.445002202284
H	5.904226057447	-0.029926112273	0.156395310083
C	5.219183087778	0.603433112058	0.734543837947
H	5.481070866409	0.503568134993	1.797967783655
H	5.371525009586	1.653305701683	0.440974134523
C	3.258818312193	0.304937381652	-0.956599753221
C	4.344280065188	0.014062460243	-1.992816829495
H	5.067686454108	0.839795736689	-2.050835413104
H	3.877758872182	-0.105883104398	-2.981793868253
H	4.887312726916	-0.911908995361	-1.754145586691
C	2.530117170375	1.601945661180	-1.286082014044
H	2.239208231463	1.620972562955	-2.348278143203
H	1.626738171144	1.735695031172	-0.676529486240
H	3.197265534565	2.459516157515	-1.111931381069
C	2.040860962705	-3.157387666856	0.114130049405
H	1.078497216042	-3.053274603867	0.647448218132
H	1.827028749383	-3.628113463768	-0.858690183301
H	2.675109715110	-3.832070233508	0.708000371498

INT-4

E_0 = -1598.43289417 a.u. (G_{298K} = -1598.04429960 a.u.)

0 1

Si	-1.248680000000	1.271804000000	0.059131000000
C	-0.610786000000	1.756952000000	1.820403000000
H	-1.421173000000	1.938229000000	2.549078000000
H	0.049252000000	0.981054000000	2.245445000000
H	-0.011933000000	2.682022000000	1.752600000000
C	-2.355877000000	2.810732000000	-0.424416000000
H	-1.657115000000	3.659934000000	-0.281771000000
C	-3.559183000000	3.031052000000	0.503942000000

C	-2.798299000000	2.829059000000	-1.895007000000
H	-1.947269000000	2.697051000000	-2.584602000000
H	-3.520854000000	2.022318000000	-2.109531000000
H	-3.301325000000	3.778458000000	-2.159133000000
H	-4.282701000000	2.202644000000	0.413181000000
H	-4.099498000000	3.966643000000	0.263038000000
H	-3.261816000000	3.087163000000	1.563589000000
C	-2.513473000000	-0.145583000000	0.434981000000
C	-2.532707000000	-0.856876000000	1.655791000000
C	-3.392746000000	-0.620835000000	-0.573020000000
C	-3.357355000000	-1.969019000000	1.857199000000
C	-4.236332000000	-1.724811000000	-0.372935000000
C	-4.214670000000	-2.413235000000	0.843663000000
H	-1.889804000000	-0.532324000000	2.478776000000
H	-3.441134000000	-0.097159000000	-1.533971000000
H	-3.335277000000	-2.492460000000	2.817944000000
H	-4.906843000000	-2.048251000000	-1.174650000000
H	-4.859348000000	-3.281514000000	1.001849000000
Li	0.420495000000	-0.037154000000	-1.368005000000
B	2.131255000000	-1.208456000000	0.661976000000
O	2.097287000000	-0.576532000000	-0.587482000000
O	3.223667000000	-0.826698000000	1.376273000000
C	3.867845000000	0.285677000000	0.699010000000
C	3.320433000000	1.567264000000	1.333061000000
H	2.236628000000	1.660340000000	1.173483000000
H	3.500864000000	1.529649000000	2.417056000000
H	3.816537000000	2.461242000000	0.928291000000
H	5.755345000000	-0.806716000000	0.617807000000
C	5.373618000000	0.181899000000	0.903572000000
H	5.612727000000	0.343608000000	1.964841000000
H	5.896847000000	0.949652000000	0.313000000000
C	3.393076000000	0.085622000000	-0.787108000000
C	4.262697000000	-0.892928000000	-1.577555000000
H	5.239771000000	-0.450619000000	-1.819521000000
H	3.755235000000	-1.147295000000	-2.519857000000
H	4.427931000000	-1.823669000000	-1.015679000000
C	3.185669000000	1.375631000000	-1.570084000000
H	2.897171000000	1.152099000000	-2.609451000000
H	2.409872000000	2.009205000000	-1.120147000000
H	4.124812000000	1.948046000000	-1.606314000000
C	1.030270000000	-2.197888000000	1.154219000000
H	0.105033000000	-1.639643000000	1.380595000000
H	0.760431000000	-2.929227000000	0.376900000000
H	1.339017000000	-2.725608000000	2.068255000000

H	-1.876926000000	-1.702951000000	-1.343687000000
Cl	-0.840671000000	-2.143168000000	-2.048573000000

TS-3

E_0 = -1598.42913583 a.u. (G_{298K} = -1598.04252731 a.u.)

0 1

Si	-1.085001000000	0.629898000000	0.292689000000
C	-0.756285000000	0.633310000000	2.196287000000
H	-1.469257000000	1.254909000000	2.767099000000
H	-0.805057000000	-0.390509000000	2.605126000000
H	0.258421000000	1.013475000000	2.406501000000
C	-1.111420000000	2.534684000000	-0.154658000000
H	-0.139914000000	2.895987000000	0.239724000000
C	-2.228375000000	3.317303000000	0.549685000000
C	-1.127835000000	2.811077000000	-1.664507000000
H	-0.310686000000	2.287301000000	-2.190029000000
H	-2.075785000000	2.478352000000	-2.121832000000
H	-1.030798000000	3.890835000000	-1.885267000000
H	-3.220522000000	2.980988000000	0.203240000000
H	-2.159542000000	4.403428000000	0.348257000000
H	-2.204009000000	3.183837000000	1.643220000000
C	-2.938812000000	0.080035000000	0.159829000000
C	-3.706530000000	-0.353855000000	1.263233000000
C	-3.575187000000	0.039946000000	-1.103435000000
C	-5.027505000000	-0.793026000000	1.118020000000
C	-4.898765000000	-0.381246000000	-1.257105000000
C	-5.632715000000	-0.804775000000	-0.142786000000
H	-3.267464000000	-0.345824000000	2.264806000000
H	-3.012502000000	0.334512000000	-1.996203000000
H	-5.590232000000	-1.124282000000	1.996125000000
H	-5.357852000000	-0.389617000000	-2.250222000000
H	-6.664950000000	-1.146617000000	-0.257994000000
Li	0.475740000000	-1.023740000000	-0.925051000000
B	2.550061000000	-1.289054000000	0.955623000000
O	2.318398000000	-1.084671000000	-0.409284000000
O	3.533419000000	-0.472572000000	1.418584000000
C	3.853426000000	0.510414000000	0.397345000000
C	3.024822000000	1.759780000000	0.709061000000
H	1.947467000000	1.551866000000	0.637110000000
H	3.241718000000	2.079195000000	1.738451000000
H	3.269279000000	2.587525000000	0.027940000000
H	5.951661000000	-0.090160000000	0.420197000000
C	5.342970000000	0.822187000000	0.462007000000
H	5.569906000000	1.338393000000	1.406240000000

H	5.634497000000	1.482950000000	-0.368960000000
C	3.394773000000	-0.219114000000	-0.919213000000
C	4.456160000000	-1.152874000000	-1.500810000000
H	5.278193000000	-0.582787000000	-1.956818000000
H	3.997344000000	-1.781376000000	-2.278250000000
H	4.874044000000	-1.813706000000	-0.727530000000
C	2.838280000000	0.702524000000	-1.996646000000
H	2.563151000000	0.122566000000	-2.891787000000
H	1.952453000000	1.252277000000	-1.650844000000
H	3.604224000000	1.433052000000	-2.296950000000
C	1.774477000000	-2.328537000000	1.826045000000
H	1.315778000000	-1.824508000000	2.691938000000
H	0.985900000000	-2.862327000000	1.275831000000
H	2.480984000000	-3.074599000000	2.228707000000
H	-1.731399000000	-1.733621000000	-0.456660000000
Cl	-1.280940000000	-2.901333000000	-0.905899000000

1

E_0 = -1598.52491552 a.u. (G_{298K} = -1598.13227194 a.u.)

0 1

Si	1.435711000000	-0.508496000000	0.374609000000
C	0.913041000000	0.297162000000	1.993196000000
H	1.557364000000	-0.041684000000	2.820170000000
H	0.993071000000	1.392341000000	1.905129000000
H	-0.130966000000	0.045951000000	2.242528000000
C	1.019426000000	-2.368712000000	0.380436000000
H	-0.072982000000	-2.360259000000	0.565110000000
C	1.661532000000	-3.151063000000	1.539390000000
C	1.251269000000	-3.052611000000	-0.977151000000
H	0.745196000000	-2.521150000000	-1.799957000000
H	2.321706000000	-3.099637000000	-1.233483000000
H	0.871866000000	-4.089494000000	-0.968191000000
H	2.757622000000	-3.207135000000	1.441118000000
H	1.286159000000	-4.188955000000	1.568129000000
H	1.443773000000	-2.693805000000	2.518013000000
C	3.221204000000	-0.086831000000	-0.061309000000
C	4.233735000000	-1.060253000000	-0.168885000000
C	3.574989000000	1.263238000000	-0.274146000000
C	5.551398000000	-0.705291000000	-0.478274000000
C	4.891089000000	1.618659000000	-0.584832000000
C	5.882516000000	0.636619000000	-0.687593000000
H	4.002956000000	-2.116001000000	-0.009981000000
H	2.815229000000	2.048971000000	-0.200603000000
H	6.319868000000	-1.479252000000	-0.556001000000

H	5.142375000000	2.670364000000	-0.747075000000
H	6.911248000000	0.916844000000	-0.930268000000
Li	-0.524827000000	1.695711000000	-0.766049000000
B	-2.868111000000	1.118771000000	0.928894000000
O	-2.185608000000	0.857540000000	-0.265995000000
O	-3.956128000000	0.308920000000	1.055327000000
C	-3.920045000000	-0.705633000000	0.020077000000
C	-3.263207000000	-1.944200000000	0.636918000000
H	-2.229416000000	-1.732310000000	0.946876000000
H	-3.830868000000	-2.236920000000	1.531726000000
H	-3.253397000000	-2.791492000000	-0.063717000000
H	-5.892922000000	-0.108994000000	-0.694821000000
C	-5.346011000000	-1.018518000000	-0.415562000000
H	-5.887135000000	-1.497008000000	0.413802000000
H	-5.346185000000	-1.711319000000	-1.271046000000
C	-3.031646000000	-0.022542000000	-1.084542000000
C	-3.825204000000	0.887965000000	-2.023638000000
H	-4.455955000000	0.301837000000	-2.706968000000
H	-3.128299000000	1.485916000000	-2.630344000000
H	-4.466714000000	1.580444000000	-1.459810000000
C	-2.151053000000	-0.978433000000	-1.877527000000
H	-1.568458000000	-0.428909000000	-2.632973000000
H	-1.447824000000	-1.519562000000	-1.234671000000
H	-2.776717000000	-1.711319000000	-2.408742000000
C	-2.424703000000	2.185905000000	1.976312000000
H	-2.040613000000	1.681309000000	2.879863000000
H	-1.638175000000	2.856583000000	1.599420000000
H	-3.294856000000	2.781807000000	2.297615000000
H	0.570508000000	0.050259000000	-0.726666000000
Cl	0.543619000000	3.415064000000	-0.108053000000

15. Preliminary Study on Asymmetric Si–H Borylation.

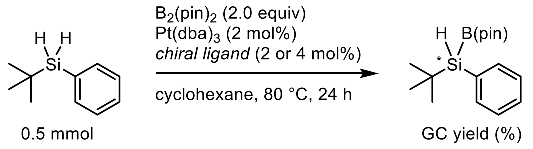

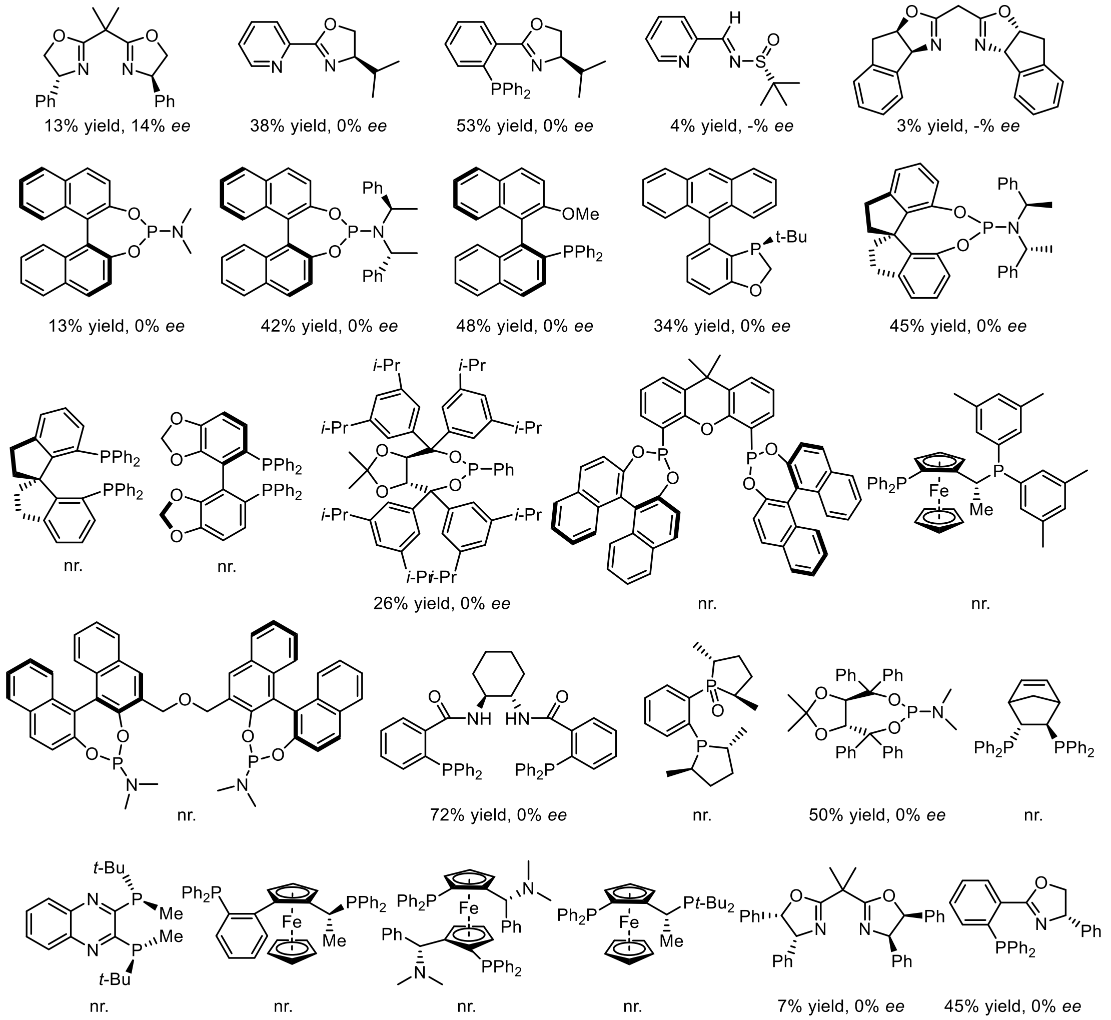

Figure S13. Chiral ligand screening for platinum-catalyzed asymmetric Si–H borylation.

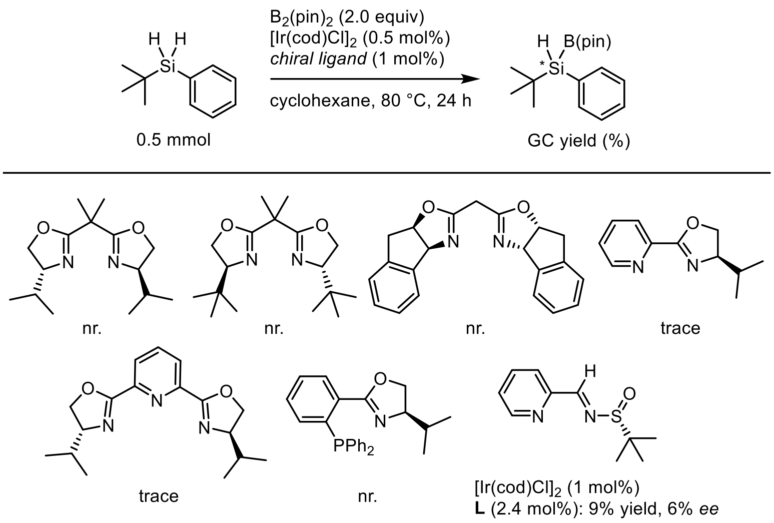

Figure S14. Chiral ligand screening for iridium-catalyzed asymmetric Si–H borylation.

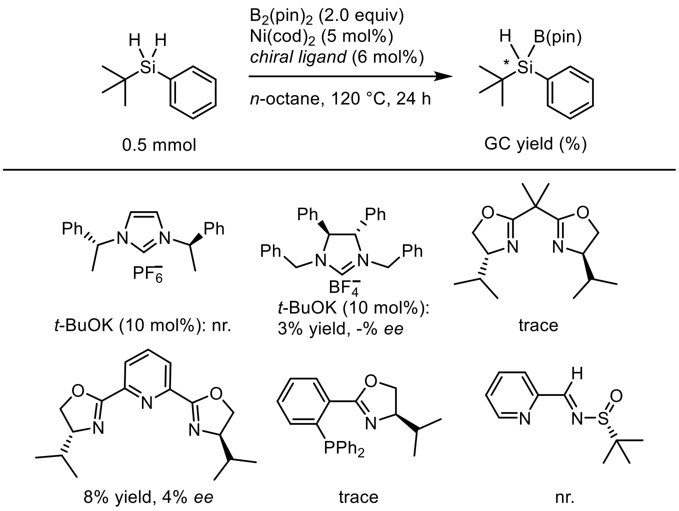

Figure S15. Chiral ligand screening for nickel-catalyzed asymmetric Si–H borylation.

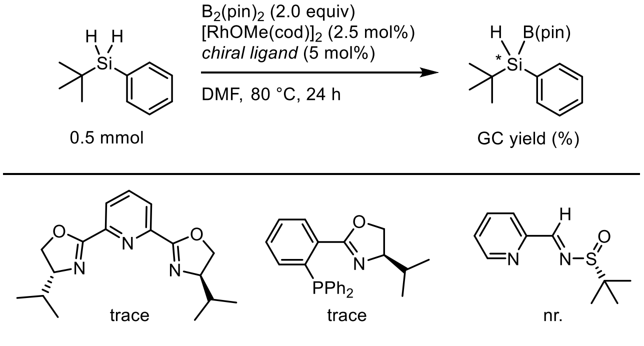

Figure S16. Chiral ligand screening for rhodium-catalyzed asymmetric Si–H borylation.

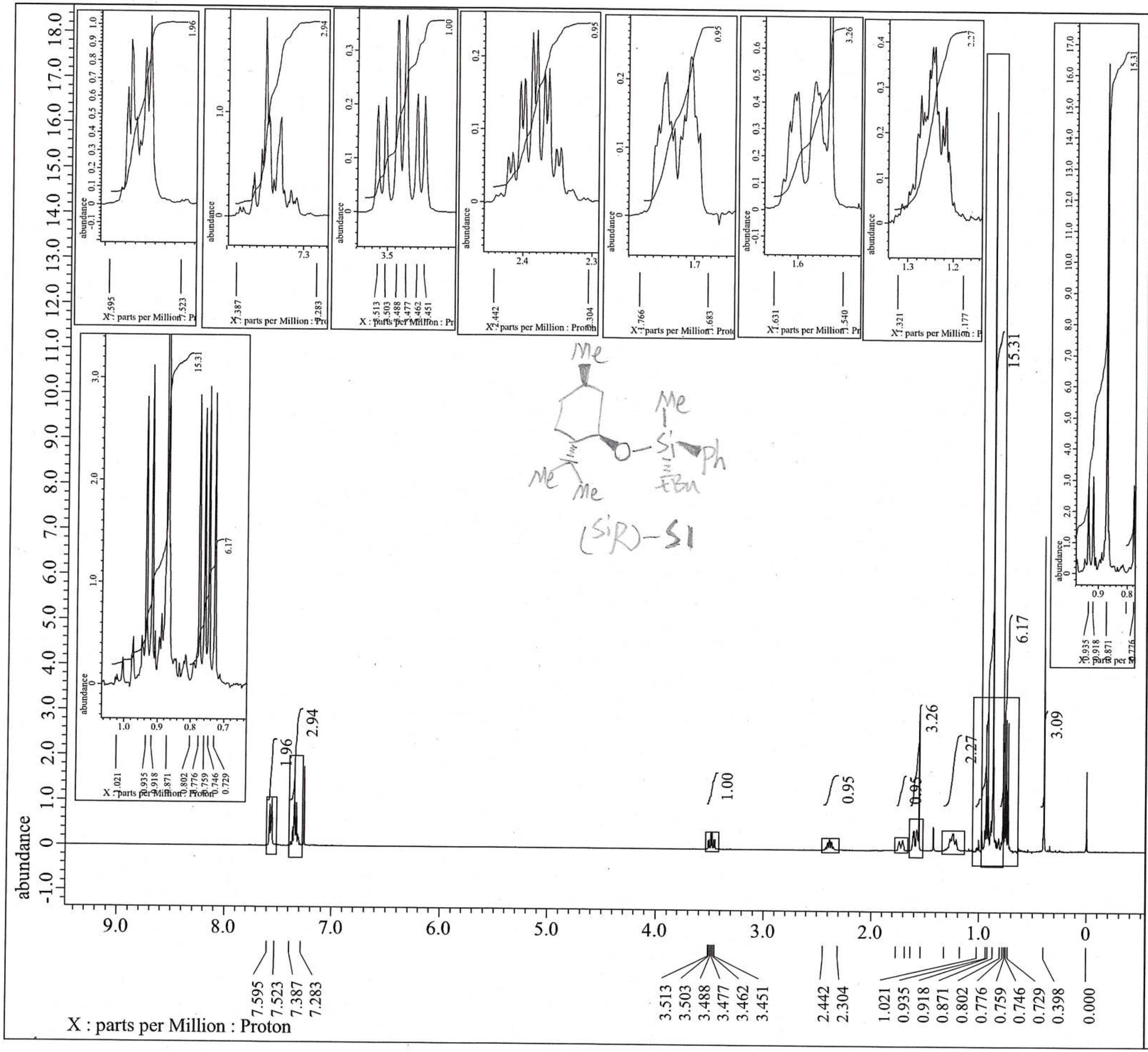


```
---- PROCESSING PARAMETERS ----
dc_balance( 0, FALSE )
sexp( 0.2[Hz], 0.0[s] )
trapezoid( 0[%], 0[%], 80[%], 100[%] )
zerofill( 1, TRUE )
fft( 1, TRUE, TRUE )
machinephase
ppm
```

数据来源: wxh-176-20_Proton-1-1.jdf

```
Filename            = wxh-176-20_Proton-1-3.jdf
Author              = element
Experiment          = proton.jxp
Sample_Id           = wxh-176-20
Solvent             = CHLOROFORM-D
Actual_Start_Time   =  4-NOV-2021 14:51:18
Revision_Time       = 27-JUN-2023 19:26:33

Comment             = single_pulse
Data_Format         = 1D COMPLEX
Dim_Size            = 13107
X_Domain            = Proton
Dim_Title           = Proton
Dim_Units           = [ppm]
Dimensions          = X
Spectrometer        = DELTA2_NMR

Field_Strength      = 9.4073814[T] (400[MHz])
X_Acq_Duration      = 2.18103808[s]
X_Domain            = 1H
X_Freq              = 400.53219825[MHz]
X_Offset            = 5[ppm]
X_Points            = 16384
X_Prescans          = 1
X_Resolution        = 0.45849727[Hz]
X_Sweep             = 7.51201923[kHz]
X_Sweep_Clipped     = 6.00961538[kHz]
Irr_Domain          = Proton
Irr_Freq            = 400.53219825[MHz]
Irr_Offset          = 5[ppm]
Tri_Domain          = Proton
Tri_Freq            = 400.53219825[MHz]
Tri_Offset          = 5[ppm]
Clipped             = FALSE
Scans               = 8
Total_Scans         = 8

Relaxation_Delay    = 5[s]
Recvr_Gain          = 36
Temp_Get            = 19[dC]
X_90_Width          = 6[us]
X_Acq_Time          = 2.18103808[s]
X_Angle             = 45[deg]
X_Atn               = 0.8[dB]
X_Pulse             = 3[us]
Irr_Mode            = Off
Tri_Mode            = Off
Dante_Presat        = FALSE
Initial_Wait        = 1[s]
Repetition_Time     = 7.18103808[s]
```

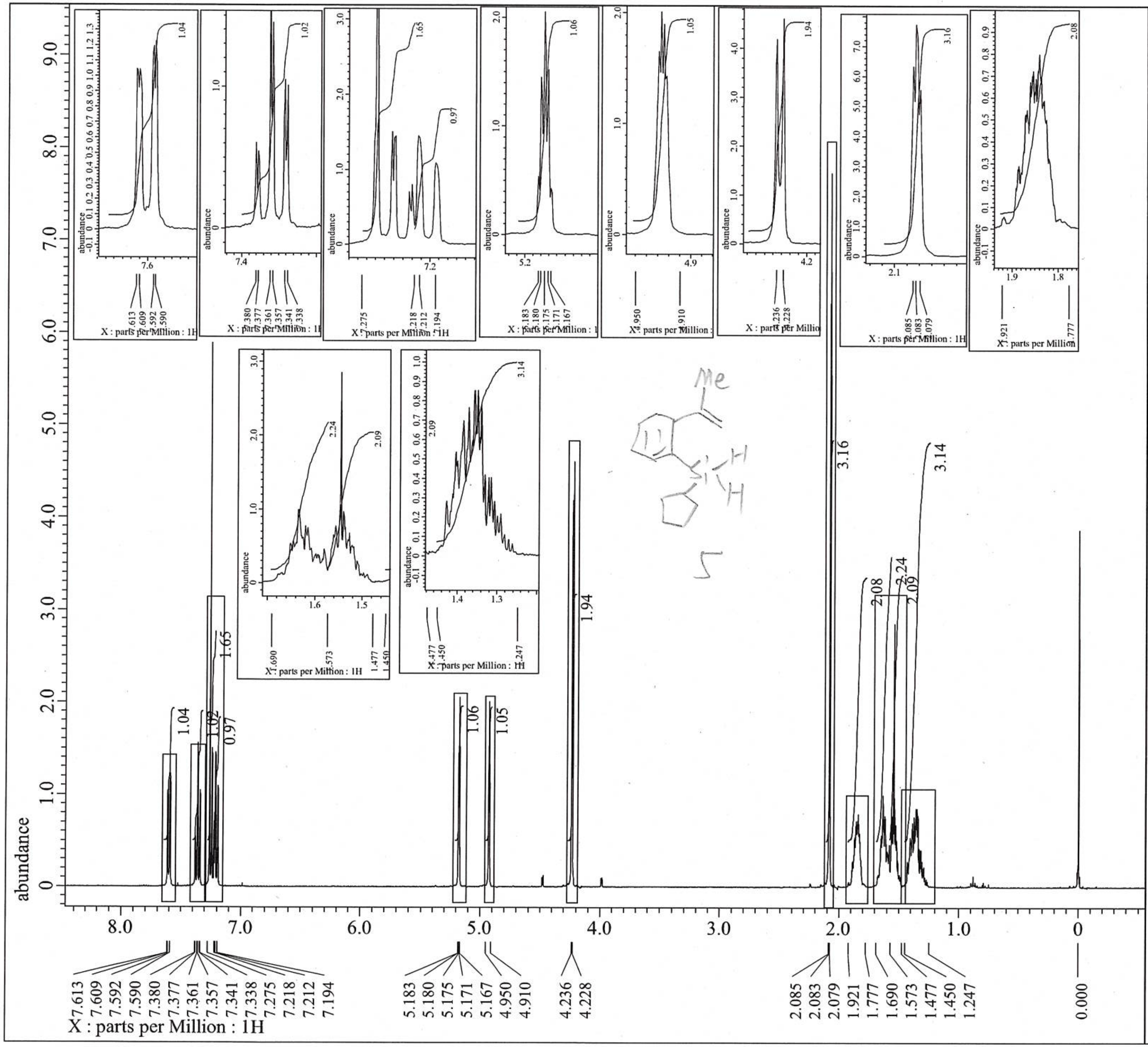


```
---- PROCESSING PARAMETERS ----
dc_balance( 0, FALSE )
sexp( 0.2[Hz], 0.0[s] )
trapezoid3( 0[%], 80[%], 100[%] )
zerofill( 1, TRUE )
fft( 1, TRUE, TRUE )
machinephase
ppm

数据来源: wxh-404-1.jdf

Filename           = wxh-404-2.jdf
Author             = element
Experiment         = single_pulse.ex2
Sample_Id          = S#522401
Solvent            = CHLOROFORM-D
Actual_Start_Time  = 10-FEB-2023 23:18:52
Revision_Time      = 27-JUN-2023 17:17:51

Comment            = single_pulse
Data_Format        = 1D COMPLEX
Dim_Size           = 13107
X_Domain           = 1H
Dim_Title          = 1H
Dim_Units          = [ppm]
Dimensions         = X
Site               = ECS 400
Spectrometer       = JNM-ECS400

Field_Strength     = 9.20197068[T] (390[MHz])
X_Acq_Duration     = 2.228224[s]
X_Domain           = 1H
X_Freq             = 391.78655441[MHz]
X_Offset           = 5[ppm]
X_Points           = 16384
X_Prescans         = 1
X_Resolution       = 0.44878791[Hz]
X_Sweep            = 7.35294118[kHz]
Irr_Domain         = 1H
Irr_Freq           = 391.78655441[MHz]
Irr_Offset         = 5[ppm]
Tri_Domain         = 1H
Tri_Freq           = 391.78655441[MHz]
Tri_Offset         = 5[ppm]
Clipped            = FALSE
Scans              = 8
Total_Scans        = 8

Relaxation_Delay   = 5[s]
Recvr_Gain         = 46
Temp_Get           = 18.2[dC]
X_90_Width         = 11.2[us]
X_Acq_Time         = 2.228224[s]
X_Angle            = 45[deg]
X_Atn              = 1.9[dB]
X_Pulse            = 5.6[us]
Irr_Mode           = Off
Tri_Mode           = Off
Dante_Presat       = FALSE
Initial_Wait       = 1[s]
Repetition_Time    = 7.228224[s]
```

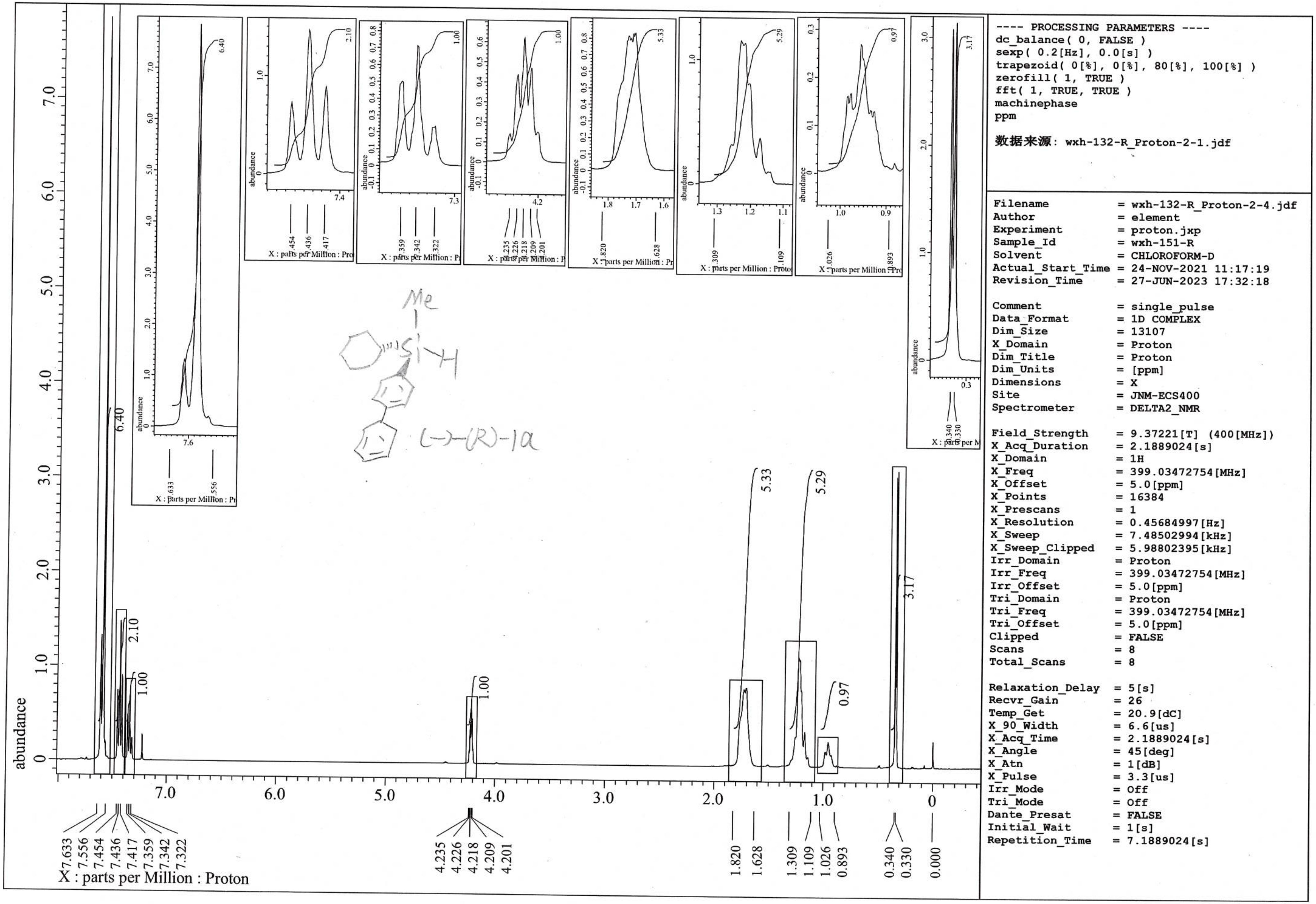
---- PROCESSING PARAMETERS ----
dc_balance(0, FALSE)
sexp(0.2[Hz], 0.0[s])
trapezoid(0[%], 0[%], 80[%], 100[%])
zerofill(1, TRUE)
fft(1, TRUE, TRUE)
machinephase
ppm
数据来源: wxh-132-R_Proton-2-1.jdf
Filename = wxh-132-R_Proton-2-4.jdf
Author = element
Experiment = proton.jxp
Sample_Id = wxh-151-R
Solvent = CHLOROFORM-D
Actual_Start_Time = 24-NOV-2021 11:17:19
Revision_Time = 27-JUN-2023 17:32:18
Comment = single_pulse
Data_Format = 1D COMPLEX
Dim_Size = 13107
X_Domain = Proton
Dim_Title = Proton
Dim_Units = [ppm]
Dimensions = X
Site = JNM-ECS400
Spectrometer = DELTA2_NMR
Field_Strength = 9.37221[T] (400[MHz])
X_Acq_Duration = 2.1889024[s]
X_Domain = 1H
X_Freq = 399.03472754[MHz]
X_Offset = 5.0[ppm]
X_Points = 16384
X_Prescans = 1
X_Resolution = 0.45684997[Hz]
X_Sweep = 7.48502994[kHz]
X_Sweep_Clipped = 5.98802395[kHz]
Irr_Domain = Proton
Irr_Freq = 399.03472754[MHz]
Irr_Offset = 5.0[ppm]
Tri_Domain = Proton
Tri_Freq = 399.03472754[MHz]
Tri_Offset = 5.0[ppm]
Clipped = FALSE
Scans = 8
Total_Scans = 8
Relaxation_Delay = 5[s]
Recvr_Gain = 26
Temp_Get = 20.9[dC]
X_90_Width = 6.6[us]
X_Acq_Time = 2.1889024[s]
X_Angle = 45[deg]
X_Atn = 1[dB]
X_Pulse = 3.3[us]
Irr_Mode = Off
Tri_Mode = Off
Dante_Presat = FALSE
Initial_Wait = 1[s]
Repetition_Time = 7.1889024[s]
Me
Si
H
(-)-(R)-1a
7.633 7.556 7.454 7.436 7.417 7.359 7.342 7.322
4.235 4.226 4.218 4.209 4.201
1.820 1.628 1.309 1.109 1.026 0.893 0.340 0.330 0.000
6.40 2.10 1.00 1.00 5.33 5.29 0.97 3.17
X : parts per Million : Proton
abundance

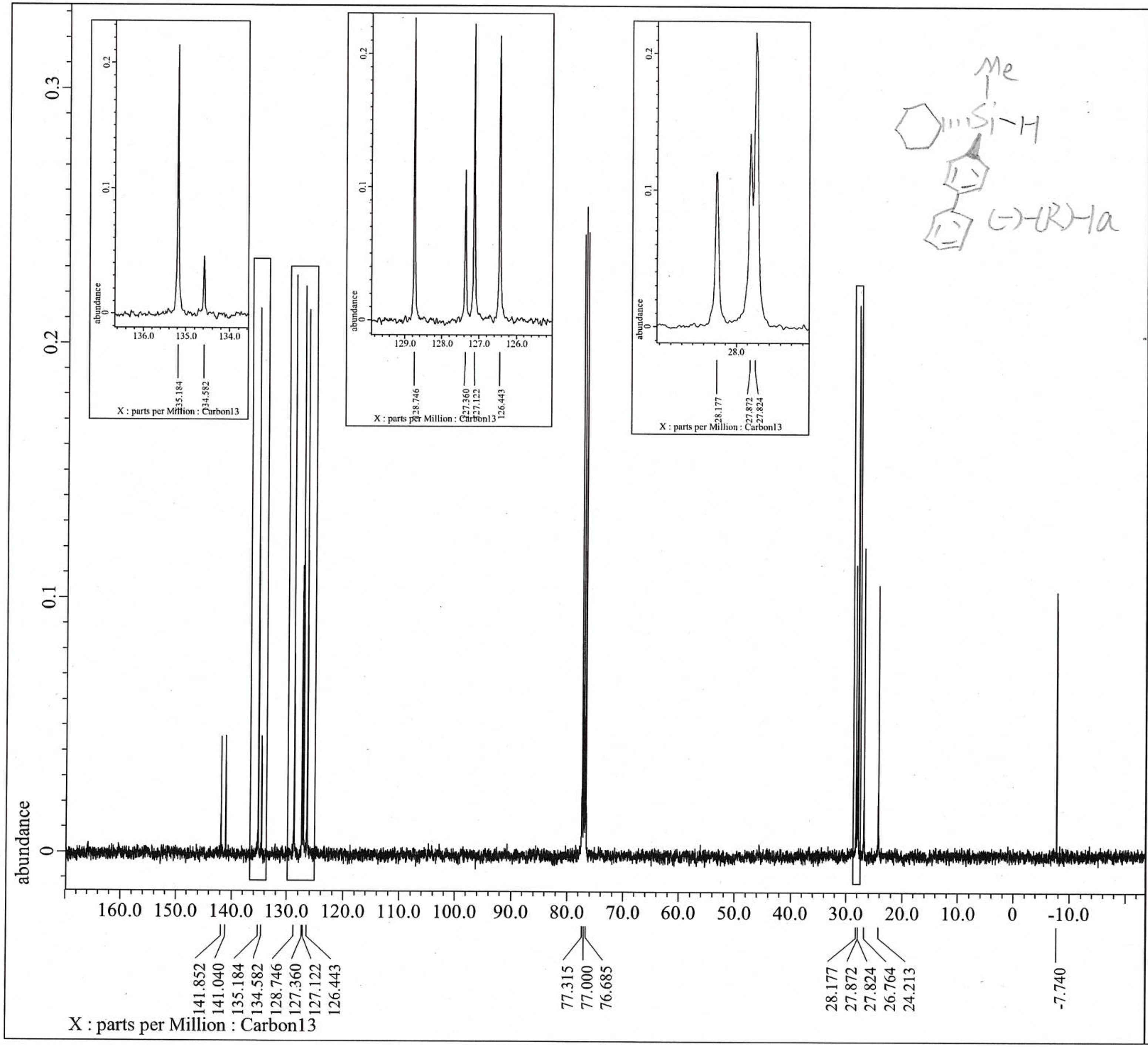


```
---- PROCESSING PARAMETERS ----
dc_balance( 0, FALSE )
sexp( 2.0[Hz], 0.0[s] )
trapezoid( 0[%], 0[%], 80[%], 100[%] )
zerofill( 1, TRUE )
fft( 1, TRUE, TRUE )
machinephase
ppm

数据来源: wxh-132-R_Carbon-1-1.jdf

Filename             = wxh-132-R_Carbon-1-2.jdf
Author               = element
Experiment           = carbon.jxp
Sample_Id            = wxh-151-R
Solvent              = CHLOROFORM-D
Actual_Start_Time    = 25-NOV-2021 17:28:43
Revision_Time        =  6-DEC-2021 16:04:55

Comment              = single pulse decoupled ga
Data_Format          = 1D COMPLEX
Dim_Size             = 26214
X_Domain             = Carbon
Dim_Title            = Carbon13
Dim_Units            = [ppm]
Dimensions           = X
Site                 = JNM-ECS400
Spectrometer         = DELTA2_NMR

Field_Strength       = 9.37221[T] (400[MHz])
X_Acq_Duration       = 1.04333312[s]
X_Domain             = 13C
X_Freq               = 100.33735165[MHz]
X_Offset             = 100.0[ppm]
X_Points             = 32768
X_Prescans           = 4
X_Resolution         = 0.95846665[Hz]
X_Sweep              = 31.40703518[kHz]
X_Sweep_Clipped      = 25.12562814[kHz]
Irr_Domain           = Proton
Irr_Freq             = 399.03472754[MHz]
Irr_Offset           = 5.0[ppm]
Clipped              = FALSE
Scans                = 256
Total_Scans          = 256

Relaxation_Delay     = 2[s]
Recvr_Gain           = 50
Temp_Get             = 20.7[dC]
X_90_Width           = 10.9[us]
X_Acq_Time           = 1.04333312[s]
X_Angle              = 30[deg]
X_Atn                = 5.4[dB]
X_Pulse              = 3.63333333[us]
Irr_Atn_Dec          = 25.823[dB]
Irr_Atn_Noe          = 25.823[dB]
Irr_Noise            = WALTZ
Irr_Pwidth           = 0.115[ms]
Decoupling           = TRUE
Initial_Wait         = 1[s]
Noe                  = TRUE
Noe_Time             = 2[s]
Repetition_Time      = 3.04333312[s]
```

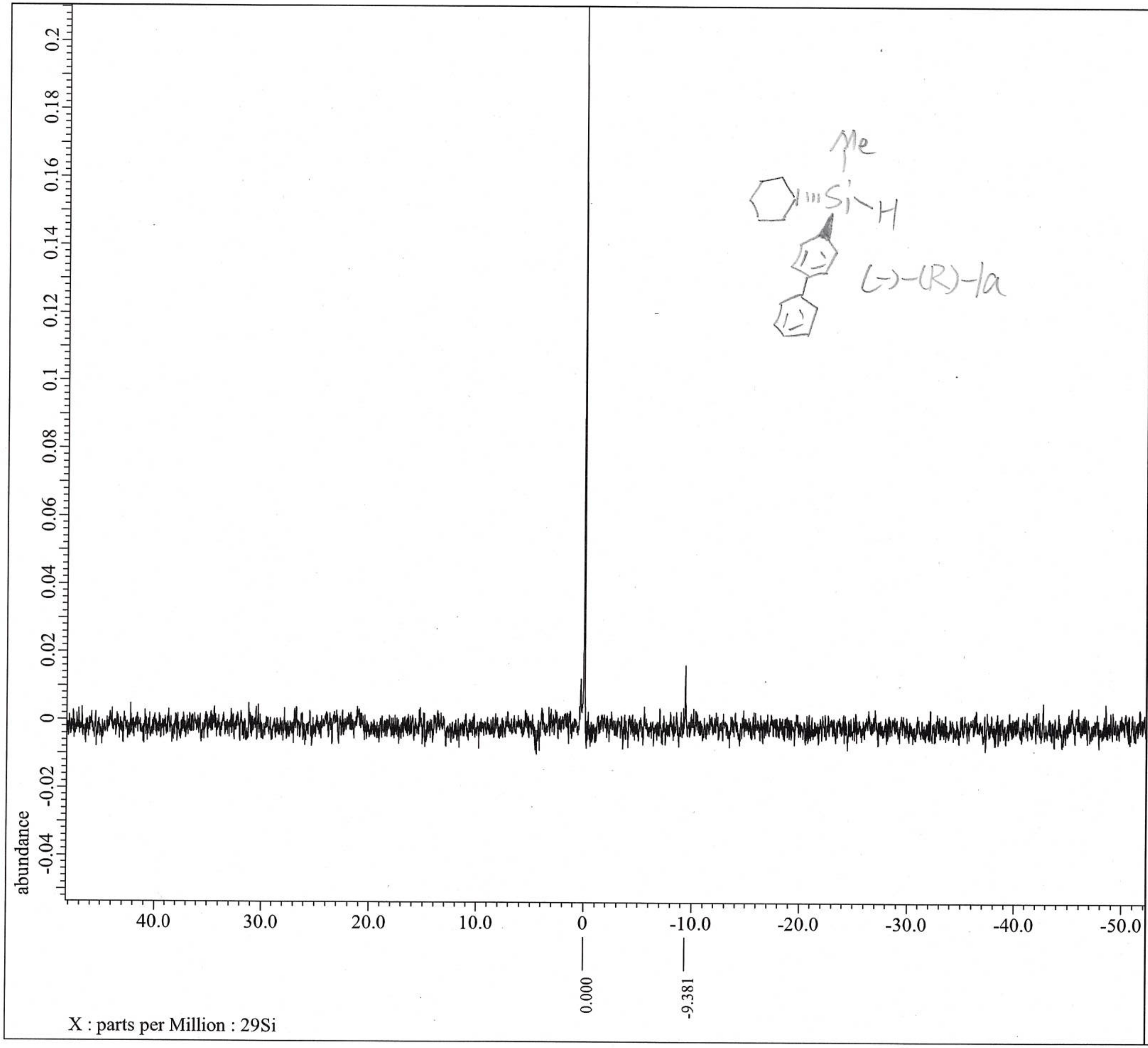


```
---- PROCESSING PARAMETERS ----
dc_balance( 0, FALSE )
sexp( 2.0[Hz], 0.0[s] )
trapezoid3( 0[%], 80[%], 100[%] )
zerofill( 1, TRUE )
fft( 1, TRUE, TRUE )
machinephase
ppm
phase( 57.79039, 0, 50[%] )

数据来源: wxh-132-Si-1-1.jdf

Filename           = wxh-132-Si-1-2.jdf
Author             = element
Experiment         = single_pulse_dec
Sample_Id          = S#484375
Solvent            = CHLOROFORM-D
Actual_Start_Time  =  4-DEC-2021 20:14:25
Revision_Time      = 25-APR-2022 11:16:54

Comment            = single pulse decoupled ga
Data_Format        = 1D COMPLEX
Dim_Size           = 26214
X_Domain           = 29Si
Dim_Title          = 29Si
Dim_Units          = [ppm]
Dimensions         = X
Site               = ECS 400
Spectrometer       = JNM-ECS400

Field_Strength     = 9.20197068[T] (390[MHz])
X_Acq_Duration     = 1.34217728[s]
X_Domain           = 29Si
X_Freq             = 77.83692472[MHz]
X_Offset           = 0[ppm]
X_Points           = 32768
X_Prescans         = 4
X_Resolution       = 0.74505806[Hz]
X_Sweep            = 24.4140625[kHz]
Irr_Domain         = 1H
Irr_Freq           = 391.78655441[MHz]
Irr_Offset         = 5[ppm]
Clipped            = FALSE
Scans              = 550
Total_Scans        = 550

Relaxation_Delay   = 9[s]
Recvr_Gain         = 60
Temp_Get           = 20.5[dC]
X_90_Width         = 10[us]
X_Acq_Time         = 1.34217728[s]
X_Angle            = 30[deg]
X_Atn              = 4.9[dB]
X_Pulse            = 3.33333333[us]
Irr_Atn_Dec        = 22.45[dB]
Irr_Noise          = WALTZ
Decoupling         = TRUE
Initial_Wait       = 1[s]
Noe                = FALSE
Repetition_Time    = 10.34217728[s]
```

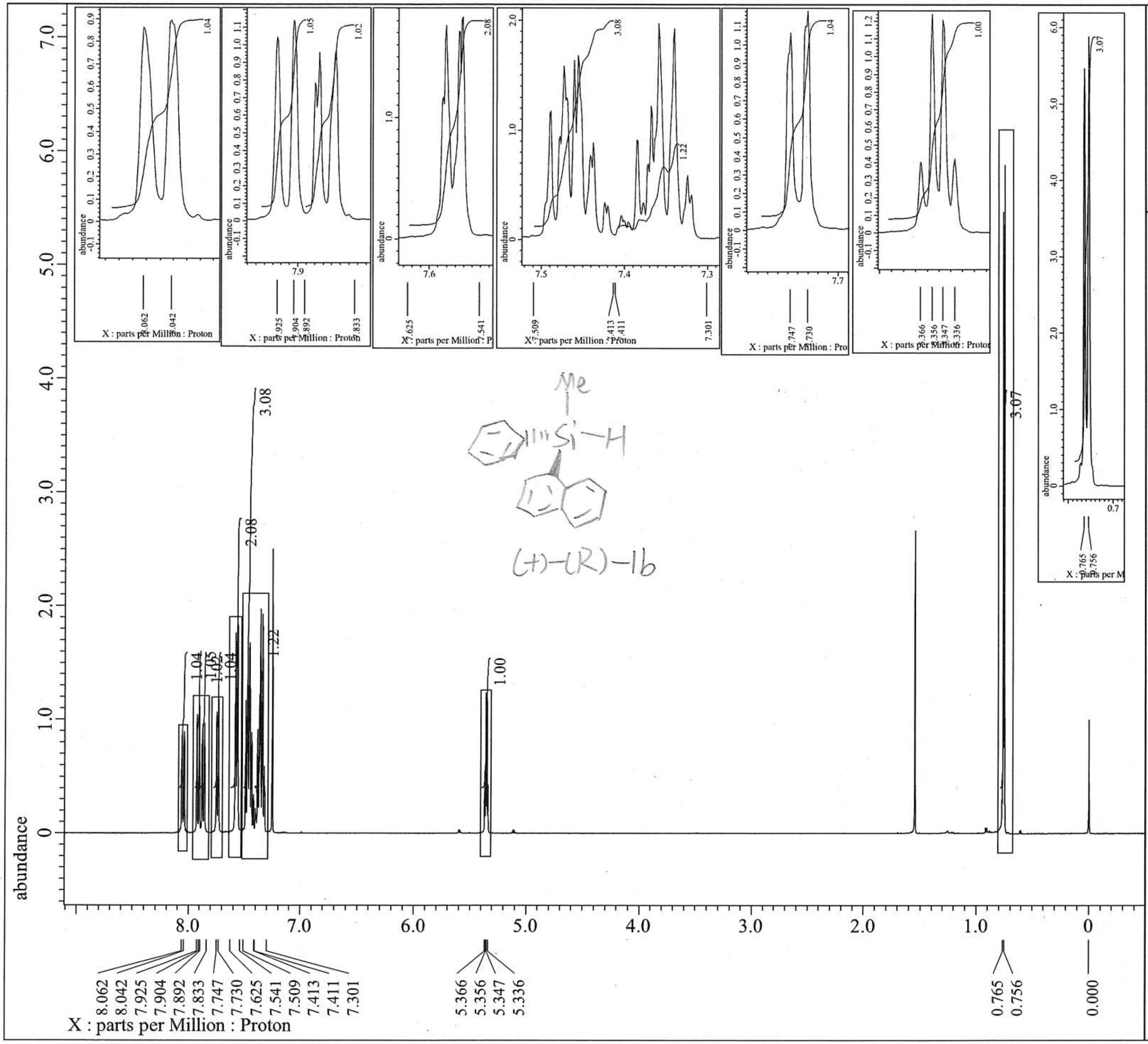

---- PROCESSING PARAMETERS ----
dc_balance(0, FALSE)
sexp(0.2[Hz], 0.0[s])
trapezoid(0[%], 0[%], 80[%], 100[%])
zerofill(1, TRUE)
fft(1, TRUE, TRUE)
machinephase
ppm

数据来源: wxh-145-3_Proton-1-1.jdf

Filename	= wxh-145-3_Proton-1-4.jdf
Author	= element
Experiment	= proton.jxp
Sample_Id	= wxh-145-2
Solvent	= CHLOROFORM-D
Actual_Start_Time	= 9-MAY-2022 15:44:48
Revision_Time	= 27-JUN-2023 19:44:06
Comment	= single_pulse
Data_Format	= 1D COMPLEX
Dim_Size	= 13107
X_Domain	= Proton
Dim_Title	= Proton
Dim_Units	= [ppm]
Dimensions	= X
Site	= JNM-ECS400
Spectrometer	= DELTA2_NMR
Field_Strength	= 9.37221[T] (400[MHz])
X_Acq_Duration	= 2.1889024[s]
X_Domain	= 1H
X_Freq	= 399.03472754[MHz]
X_Offset	= 5.0[ppm]
X_Points	= 16384
X_Prescans	= 1
X_Resolution	= 0.45684997[Hz]
X_Sweep	= 7.48502994[kHz]
X_Sweep_Clipped	= 5.98802395[kHz]
Irr_Domain	= Proton
Irr_Freq	= 399.03472754[MHz]
Irr_Offset	= 5.0[ppm]
Tri_Domain	= Proton
Tri_Freq	= 399.03472754[MHz]
Tri_Offset	= 5.0[ppm]
Clipped	= FALSE
Scans	= 8
Total_Scans	= 8
Relaxation_Delay	= 5[s]
Recvr_Gain	= 40
Temp_Get	= 17.5[dC]
X_90_Width	= 6.6[us]
X_Acq_Time	= 2.1889024[s]
X_Angle	= 45[deg]
X_Atn	= 1[dB]
X_Pulse	= 3.3[us]
Irr_Mode	= Off
Tri_Mode	= Off
Dante_Presat	= FALSE
Initial_Wait	= 1[s]
Repetition_Time	= 7.1889024[s]

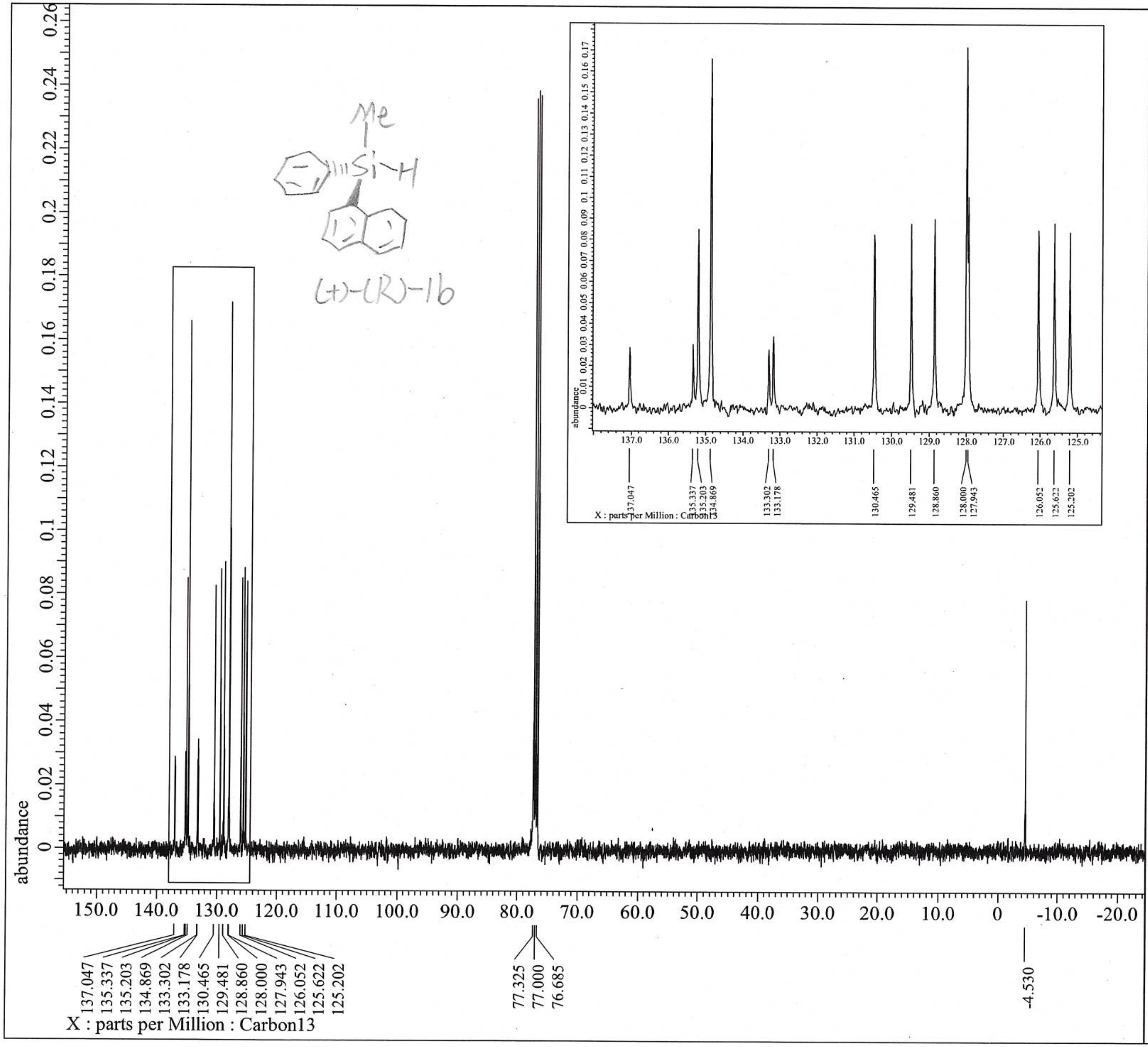

---- PROCESSING PARAMETERS ----
dc_balance(0, FALSE)
sexp(2.0[Hz], 0.0[s])
trapezoid(0[%], 0[%], 80[%], 100[%])
zerofill(1, TRUE)
fft(1, TRUE, TRUE)
machinephase
ppm

数据来源: wxh-145-2_Carbon-1-1.jdf

Filename	= wxh-145-2_Carbon-1-2.jdf
Author	= element
Experiment	= carbon.jxp
Sample_Id	= wxh-145-2
Solvent	= CHLOROFORM-D
Actual_Start_Time	= 23-APR-2022 14:25:46
Revision_Time	= 23-APR-2022 16:55:09
Comment	= single pulse decoupled ga
Data_Format	= 1D COMPLEX
Dim_Size	= 26214
X_Domain	= Carbon
Dim_Title	= Carbon13
Dim_Units	= [ppm]
Dimensions	= X
Site	= JNM-ECS400
Spectrometer	= DELTA2_NMR
Field_Strength	= 9.37221[T] (400[MHz])
X_Acq_Duration	= 1.04333312[s]
X_Domain	= 13C
X_Freq	= 100.33735165[MHz]
X_Offset	= 100.0[ppm]
X_Points	= 32768
X_Prescans	= 4
X_Resolution	= 0.95846665[Hz]
X_Sweep	= 31.40703518[kHz]
X_Sweep_Clipped	= 25.12562814[kHz]
Irr_Domain	= Proton
Irr_Freq	= 399.03472754[MHz]
Irr_Offset	= 5.0[ppm]
Clipped	= FALSE
Scans	= 256
Total_Scans	= 256
Relaxation_Delay	= 2[s]
Recvr_Gain	= 50
Temp_Get	= 18.3[dC]
X_90_Width	= 10.9[us]
X_Acq_Time	= 1.04333312[s]
X_Angle	= 30[deg]
X_Atn	= 5.4[dB]
X_Pulse	= 3.63333333[us]
Irr_Atn_Dec	= 25.823[dB]
Irr_Atn_Noe	= 25.823[dB]
Irr_Noise	= WALTZ
Irr_Pwidth	= 0.115[ms]
Decoupling	= TRUE
Initial_Wait	= 1[s]
Noe	= TRUE
Noe_Time	= 2[s]
Repetition_Time	= 3.04333312[s]

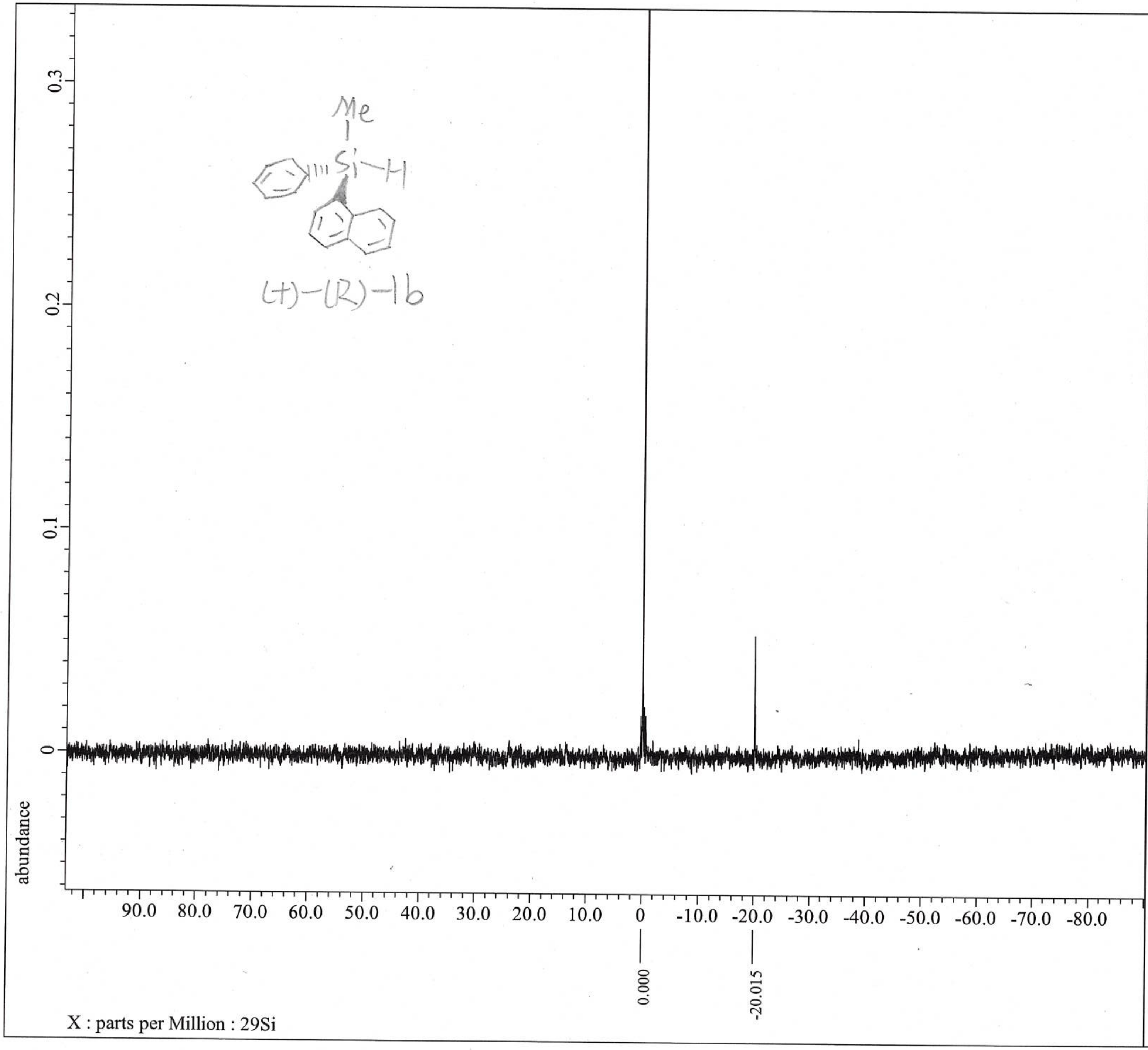


```
---- PROCESSING PARAMETERS ----
dc_balance( 0, FALSE )
sexp( 2.0[Hz], 0.0[s] )
trapezoid3( 0[%], 80[%], 100[%] )
zerofill( 1, TRUE )
fft( 1, TRUE, TRUE )
machinephase
ppm
phase( 19.25009, 0, 50[%] )

数据来源: wxh-145-Si-1-1.jdf

Filename            = wxh-145-Si-1-2.jdf
Author              = element
Experiment          = single_pulse_dec
Sample_Id           = S#408201
Solvent             = CHLOROFORM-D
Actual_Start_Time   = 23-AUG-2022 18:03:43
Revision_Time       = 21-JAN-2023 13:54:47

Comment             = single pulse decoupled ga
Data_Format         = 1D COMPLEX
Dim_Size            = 26214
X_Domain            = 29Si
Dim_Title           = 29Si
Dim_Units           = [ppm]
Dimensions          = X
Site                = ECS 400
Spectrometer        = JNM-ECS400

Field_Strength      = 9.20197068[T] (390[MHz])
X_Acq_Duration      = 1.34217728[s]
X_Domain            = 29Si
X_Freq              = 77.83692472[MHz]
X_Offset            = 0[ppm]
X_Points            = 32768
X_Prescans          = 4
X_Resolution        = 0.74505806[Hz]
X_Sweep             = 24.4140625[kHz]
Irr_Domain          = 1H
Irr_Freq            = 391.78655441[MHz]
Irr_Offset          = 5[ppm]
Clipped             = FALSE
Scans               = 560
Total_Scans         = 560

Relaxation_Delay    = 10[s]
Recvr_Gain          = 60
Temp_Get            = 21.7[dC]
X_90_Width          = 10[us]
X_Acq_Time          = 1.34217728[s]
X_Angle             = 30[deg]
X_Atn               = 4.9[dB]
X_Pulse             = 3.33333333[us]
Irr_Atn_Dec         = 22.05[dB]
Irr_Noise           = WALTZ
Decoupling          = TRUE
Initial_Wait        = 1[s]
Noe                 = FALSE
Repetition_Time     = 11.34217728[s]
```

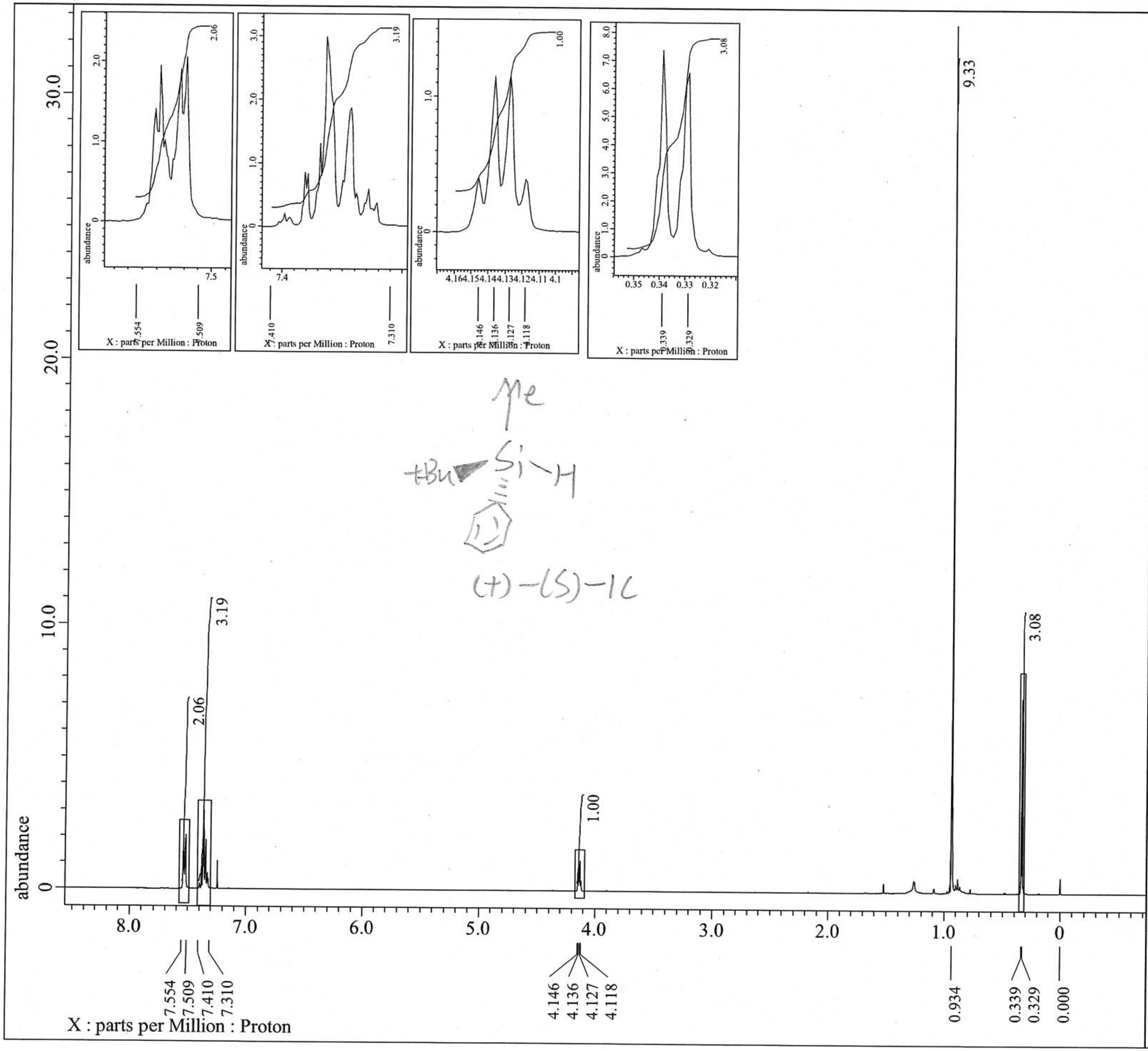


```
---- PROCESSING PARAMETERS ----
dc_balance( 0, FALSE )
sexp( 0.2[Hz], 0.0[s] )
trapezoid( 0[%], 0[%], 80[%], 100[%] )
zerofill( 1, TRUE )
fft( 1, TRUE, TRUE )
machinephase
ppm

数据来源: wxh-183-5_Proton-1-1.jdf

Filename            = wxh-183-5_Proton-1-2.jdf
Author              = element
Experiment          = proton.jxp
Sample_Id           = wxh-183-5
Solvent             = CHLOROFORM-D
Actual_Start_Time   = 16-DEC-2021 17:47:25
Revision_Time       = 27-JUN-2023 19:57:58

Comment             = single_pulse
Data_Format         = 1D COMPLEX
Dim_Size            = 13107
X_Domain            = Proton
Dim_Title           = Proton
Dim_Units           = [ppm]
Dimensions          = X
Site                = JNM-ECS400
Spectrometer        = DELTA2_NMR

Field_Strength      = 9.37221[T] (400[MHz])
X_Acq_Duration      = 2.1889024[s]
X_Domain            = 1H
X_Freq              = 399.03472754[MHz]
X_Offset            = 5.0[ppm]
X_Points            = 16384
X_Prescans          = 1
X_Resolution        = 0.45684997[Hz]
X_Sweep             = 7.48502994[kHz]
X_Sweep_Clipped     = 5.98802395[kHz]
Irr_Domain          = Proton
Irr_Freq            = 399.03472754[MHz]
Irr_Offset          = 5.0[ppm]
Tri_Domain          = Proton
Tri_Freq            = 399.03472754[MHz]
Tri_Offset          = 5.0[ppm]
Clipped             = FALSE
Scans               = 8
Total_Scans         = 8

Relaxation_Delay    = 5[s]
Recvr_Gain          = 28
Temp_Get            = 20.5[dC]
X_90_Width          = 6.6[us]
X_Acq_Time          = 2.1889024[s]
X_Angle             = 45[deg]
X_Atn               = 1[dB]
X_Pulse             = 3.3[us]
Irr_Mode            = Off
Tri_Mode            = Off
Dante_Presat        = FALSE
Initial_Wait        = 1[s]
Repetition_Time     = 7.1889024[s]
```

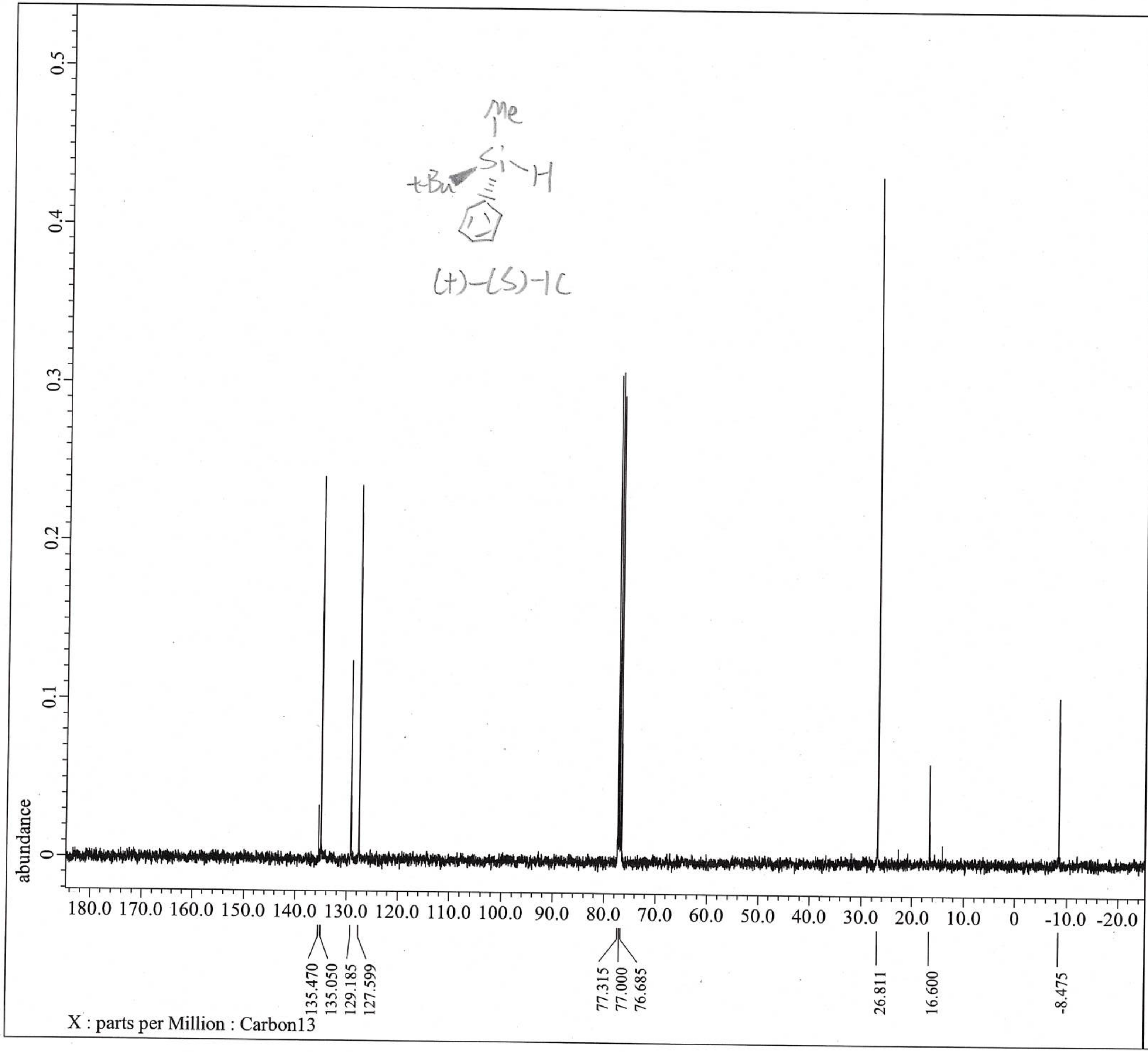


```
---- PROCESSING PARAMETERS ----
dc_balance( 0, FALSE )
sexp( 2.0[Hz], 0.0[s] )
trapezoid( 0[%], 0[%], 80[%], 100[%] )
zerofill( 1, TRUE )
fft( 1, TRUE, TRUE )
machinephase
ppm

数据来源: wxh-183-5_Carbon-2-1.jdf
```

```
Filename            = wxh-183-5_Carbon-2-2.jdf
Author              = element
Experiment          = carbon.jxp
Sample_Id           = wxh-183-5
Solvent             = CHLOROFORM-D
Actual_Start_Time   = 16-DEC-2021 19:38:46
Revision_Time       = 27-DEC-2021 16:41:04

Comment             = single pulse decoupled ga
Data_Format         = 1D COMPLEX
Dim_Size            = 26214
X_Domain            = Carbon
Dim_Title           = Carbon13
Dim_Units           = [ppm]
Dimensions          = X
Site                = JNM-ECS400
Spectrometer        = DELTA2_NMR

Field_Strength      = 9.37221[T] (400[MHz])
X_Acq_Duration      = 1.04333312[s]
X_Domain            = 13C
X_Freq              = 100.33735165[MHz]
X_Offset            = 100.0[ppm]
X_Points            = 32768
X_Prescans          = 4
X_Resolution        = 0.95846665[Hz]
X_Sweep             = 31.40703518[kHz]
X_Sweep_Clipped     = 25.12562814[kHz]
Irr_Domain          = Proton
Irr_Freq            = 399.03472754[MHz]
Irr_Offset          = 5.0[ppm]
Clipped             = FALSE
Scans               = 128
Total_Scans         = 128

Relaxation_Delay    = 2[s]
Recvr_Gain          = 50
Temp_Get            = 20.5[dC]
X_90_Width          = 10.9[us]
X_Acq_Time          = 1.04333312[s]
X_Angle             = 30[deg]
X_Atn               = 5.4[dB]
X_Pulse             = 3.63333333[us]
Irr_Atn_Dec         = 25.823[dB]
Irr_Atn_Noe         = 25.823[dB]
Irr_Noise           = WALTZ
Irr_Pwidth          = 0.115[ms]
Decoupling          = TRUE
Initial_Wait        = 1[s]
Noe                 = TRUE
Noe_Time            = 2[s]
Repetition_Time     = 3.04333312[s]
```

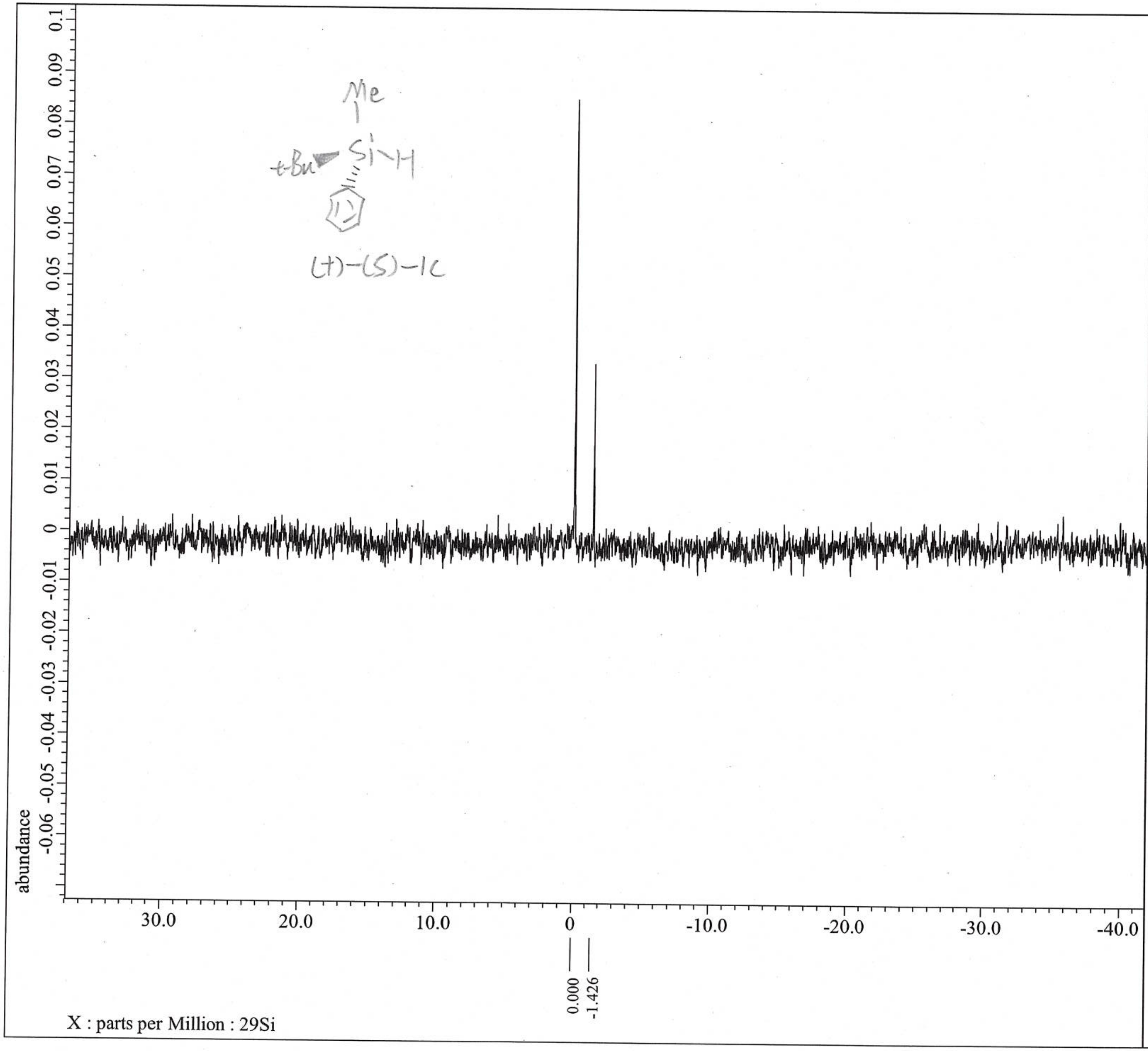


```
---- PROCESSING PARAMETERS ----
dc_balance( 0, FALSE )
sexp( 2.0[Hz], 0.0[s] )
trapezoid3( 0[%], 80[%], 100[%] )
zerofill( 1, TRUE )
fft( 1, TRUE, TRUE )
machinephase
ppm
phase( 78.561, 0, 50[%] )

数据来源: wxh-183-Si-1.jdf
```

```
Filename            = wxh-183-Si-2.jdf
Author              = element
Experiment          = single_pulse_dec
Sample_Id           = S#426154
Solvent             = CHLOROFORM-D
Actual_Start_Time = 18-DEC-2021 18:35:14
Revision_Time       = 27-DEC-2021 09:36:46

Comment             = single pulse decoupled ga
Data_Format         = 1D COMPLEX
Dim_Size            = 26214
X_Domain            = 29Si
Dim_Title           = 29Si
Dim_Units           = [ppm]
Dimensions          = X
Site                = ECS 400
Spectrometer        = JNM-ECS400

Field_Strength      = 9.20197068[T] (390[MHz])
X_Acq_Duration      = 1.34217728[s]
X_Domain            = 29Si
X_Freq              = 77.83692472[MHz]
X_Offset            = 0[ppm]
X_Points            = 32768
X_Prescans          = 4
X_Resolution        = 0.74505806[Hz]
X_Sweep             = 24.4140625[kHz]
Irr_Domain          = 1H
Irr_Freq            = 391.78655441[MHz]
Irr_Offset          = 5[ppm]
Clipped             = FALSE
Scans               = 800
Total_Scans         = 800

Relaxation_Delay    = 7[s]
Recvr_Gain          = 60
Temp_Get            = 20.9[dC]
X_90_Width          = 10[us]
X_Acq_Time          = 1.34217728[s]
X_Angle             = 30[deg]
X_Atn               = 4.9[dB]
X_Pulse             = 3.33333333[us]
Irr_Atn_Dec         = 22.45[dB]
Irr_Noise           = WALTZ
Decoupling          = TRUE
Initial_Wait        = 1[s]
Noe                 = FALSE
Repetition_Time     = 8.34217728[s]
```

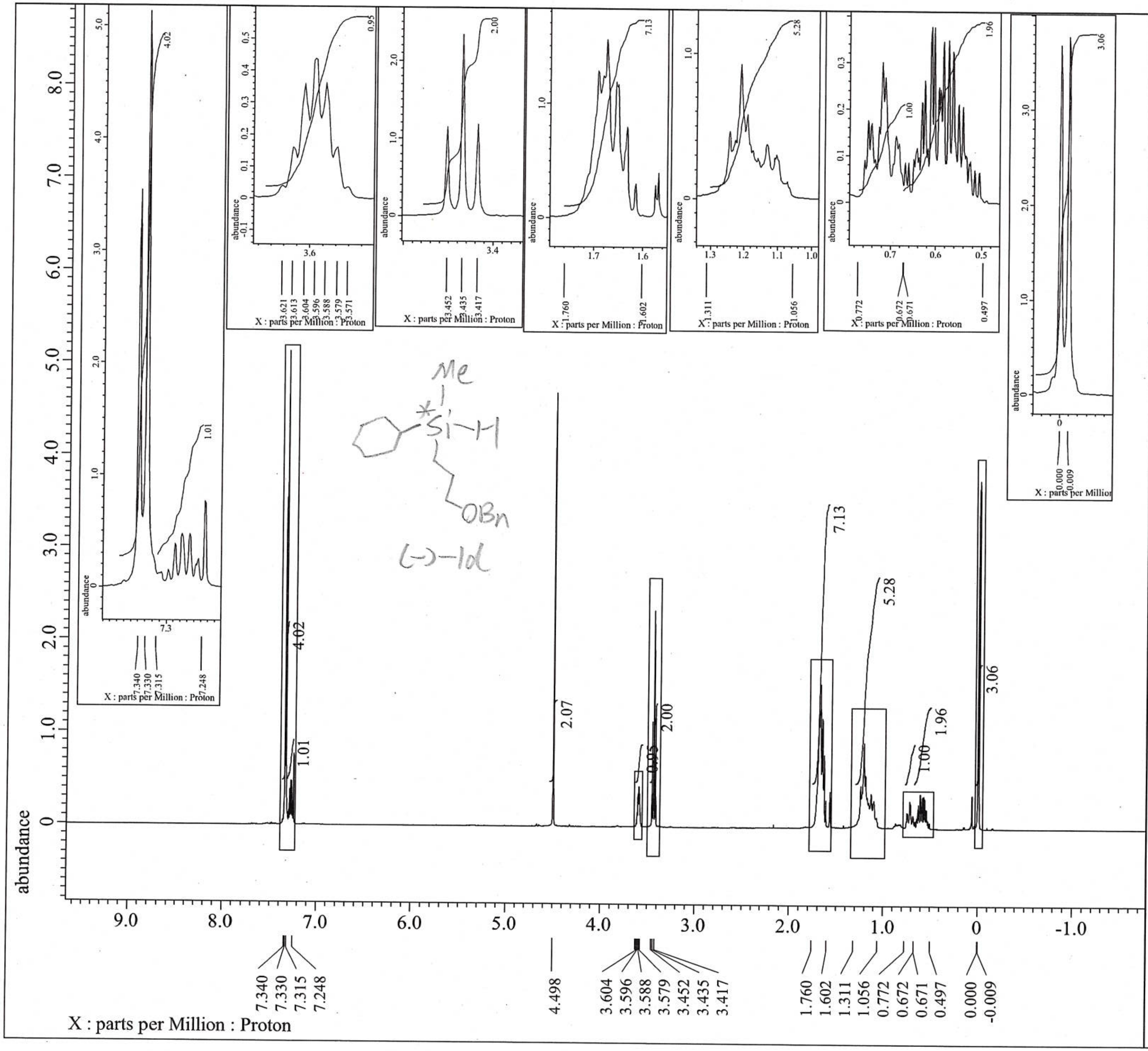


```
---- PROCESSING PARAMETERS ----
dc_balance( 0, FALSE )
sexp( 0.2[Hz], 0.0[s] )
trapezoid( 0[%], 0[%], 80[%], 100[%] )
zerofill( 1, TRUE )
fft( 1, TRUE, TRUE )
machinephase
ppm

数据来源: wxh-237-3_Proton-1-1.jdf
```

```
Filename           = wxh-237-3_Proton-1-3.jdf
Author             = element
Experiment         = proton.jxp
Sample_Id          = wxh-237-2
Solvent            = CHLOROFORM-D
Actual_Start_Time  = 23-APR-2022 09:57:55
Revision_Time      = 27-JUN-2023 20:24:15

Comment            = single_pulse
Data_Format        = 1D COMPLEX
Dim_Size           = 13107
X_Domain           = Proton
Dim_Title          = Proton
Dim_Units          = [ppm]
Dimensions         = X
Site               = JNM-ECS400
Spectrometer       = DELTA2_NMR

Field_Strength     = 9.37221[T] (400[MHz])
X_Acq_Duration     = 2.1889024[s]
X_Domain           = 1H
X_Freq             = 399.03472754[MHz]
X_Offset           = 5.0[ppm]
X_Points           = 16384
X_Prescans         = 1
X_Resolution       = 0.45684997[Hz]
X_Sweep            = 7.48502994[kHz]
X_Sweep_Clipped    = 5.98802395[kHz]
Irr_Domain         = Proton
Irr_Freq           = 399.03472754[MHz]
Irr_Offset         = 5.0[ppm]
Tri_Domain         = Proton
Tri_Freq           = 399.03472754[MHz]
Tri_Offset         = 5.0[ppm]
Clipped            = FALSE
Scans              = 8
Total_Scans        = 8

Relaxation_Delay   = 5[s]
Recvr_Gain         = 28
Temp_Get           = 18[dC]
X_90_Width         = 6.6[us]
X_Acq_Time         = 2.1889024[s]
X_Angle            = 45[deg]
X_Atn              = 1[dB]
X_Pulse            = 3.3[us]
Irr_Mode           = Off
Tri_Mode           = Off
Dante_Presat       = FALSE
Initial_Wait       = 1[s]
Repetition_Time    = 7.1889024[s]
```

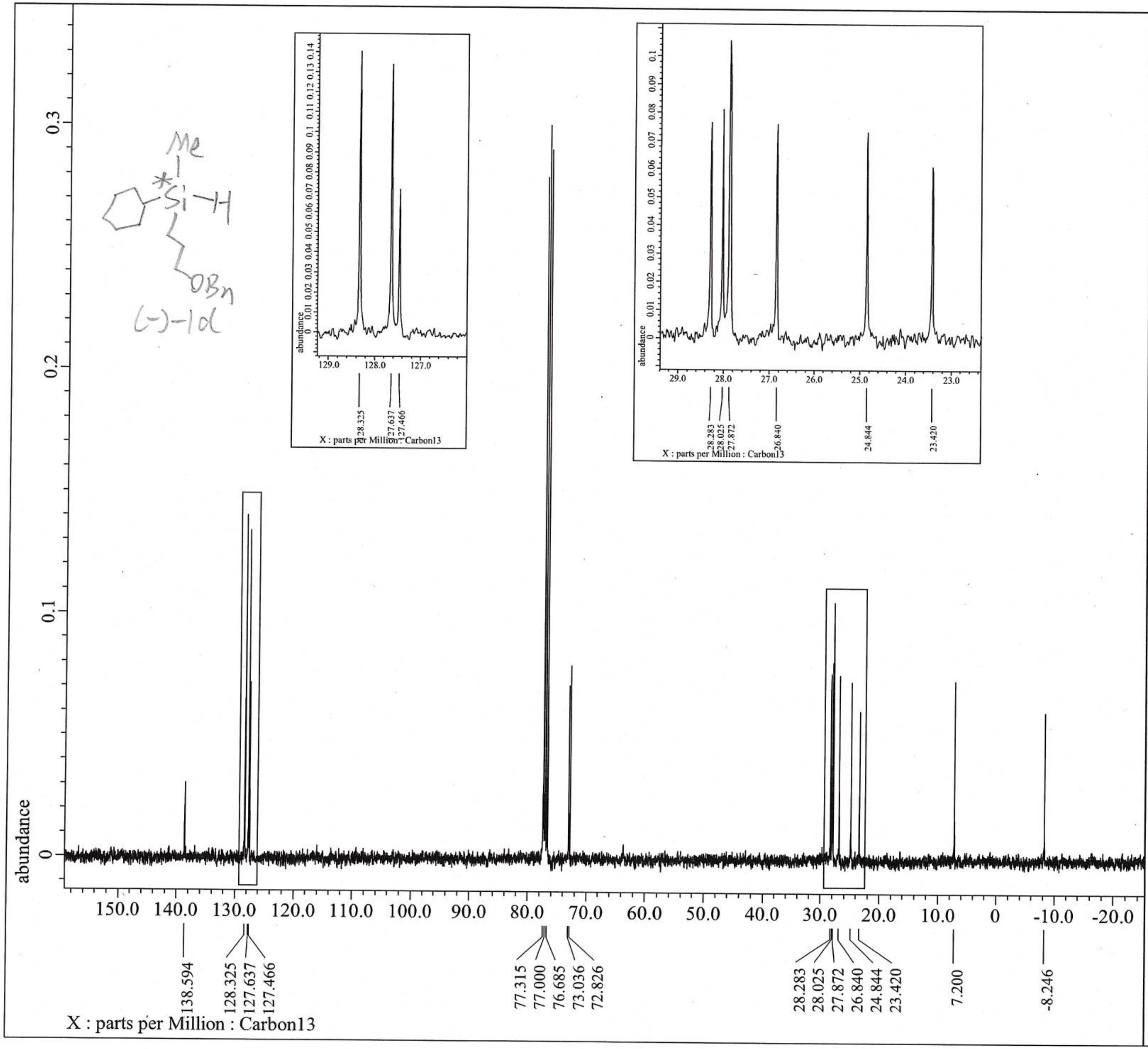

---- PROCESSING PARAMETERS ----
dc_balance(0, FALSE)
sexp(2.0[Hz], 0.0[s])
trapezoid(0[%], 0[%], 80[%], 100[%])
zerofill(1, TRUE)
fft(1, TRUE, TRUE)
machinephase
ppm

数据来源: wxh-237-2_Carbon-1-1.jdf

Filename	= wxh-237-2_Carbon-1-2.jdf
Author	= element
Experiment	= carbon.jxp
Sample_Id	= wxh-237-2
Solvent	= CHLOROFORM-D
Actual_Start_Time	= 23-APR-2022 09:59:15
Revision_Time	= 23-APR-2022 17:34:47
Comment	= single pulse decoupled ga
Data_Format	= 1D COMPLEX
Dim_Size	= 26214
X_Domain	= Carbon
Dim_Title	= Carbon13
Dim_Units	= [ppm]
Dimensions	= X
Site	= JNM-ECS400
Spectrometer	= DELTA2_NMR
Field_Strength	= 9.37221[T] (400[MHz])
X_Acq_Duration	= 1.04333312[s]
X_Domain	= 13C
X_Freq	= 100.33735165[MHz]
X_Offset	= 100.0[ppm]
X_Points	= 32768
X_Prescans	= 4
X_Resolution	= 0.95846665[Hz]
X_Sweep	= 31.40703518[kHz]
X_Sweep_Clipped	= 25.12562814[kHz]
Irr_Domain	= Proton
Irr_Freq	= 399.03472754[MHz]
Irr_Offset	= 5.0[ppm]
Clipped	= FALSE
Scans	= 256
Total_Scans	= 256
Relaxation_Delay	= 2[s]
Recvr_Gain	= 50
Temp_Get	= 17.8[dC]
X_90_Width	= 10.9[us]
X_Acq_Time	= 1.04333312[s]
X_Angle	= 30[deg]
X_Atn	= 5.4[dB]
X_Pulse	= 3.63333333[us]
Irr_Atn_Dec	= 25.823[dB]
Irr_Atn_Noe	= 25.823[dB]
Irr_Noise	= WALTZ
Irr_Pwidth	= 0.115[ms]
Decoupling	= TRUE
Initial_Wait	= 1[s]
Noe	= TRUE
Noe_Time	= 2[s]
Repetition_Time	= 3.04333312[s]

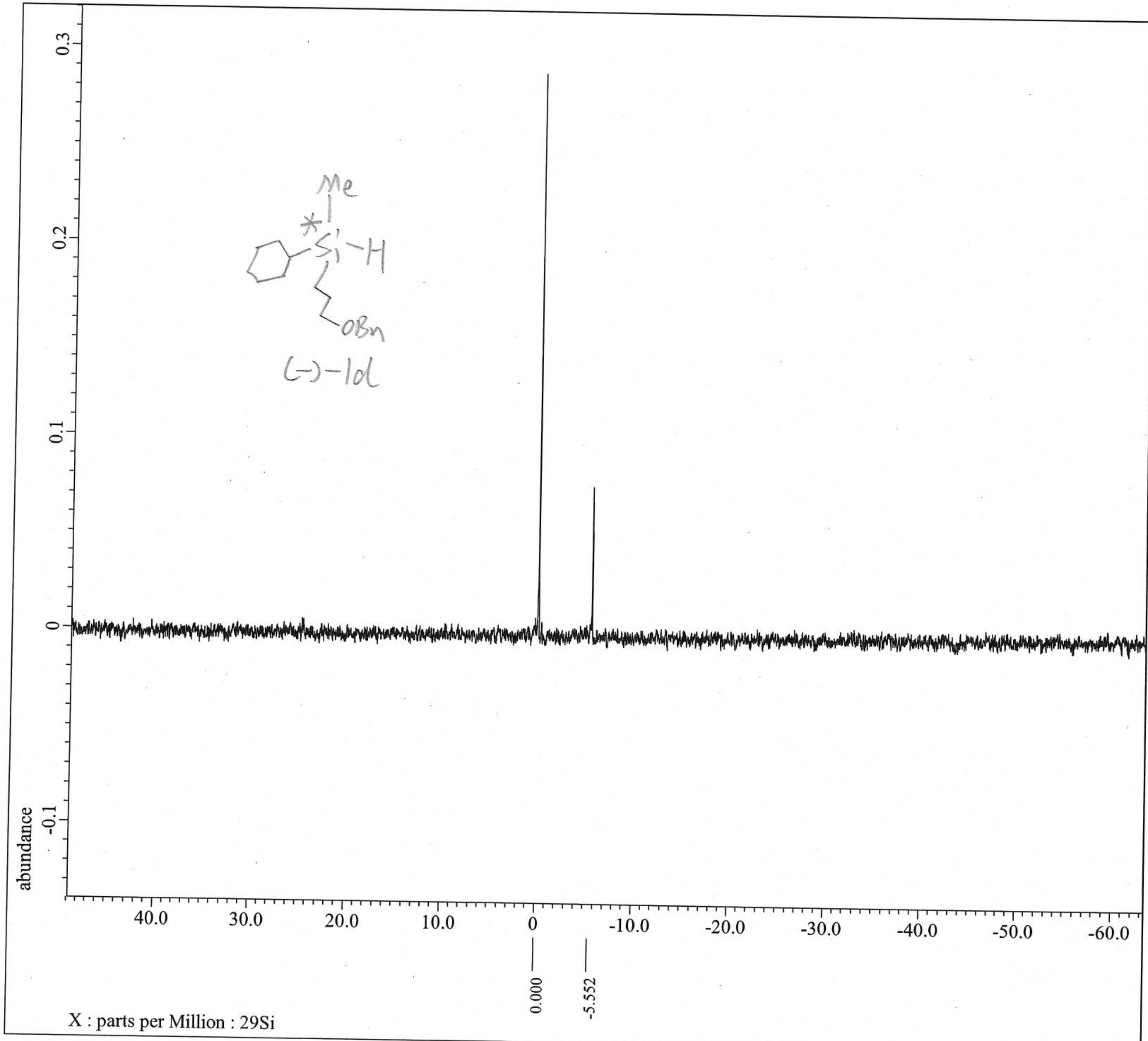


```
---- PROCESSING PARAMETERS ----
dc_balance( 0, FALSE )
sexp( 2.0[Hz], 0.0[s] )
trapezoid3( 0[%], 80[%], 100[%] )
zerofill( 1, TRUE )
fft( 1, TRUE, TRUE )
machinephase
ppm
phase( 53.82958, 0, 50[%] )

数据来源: wxh-237-Si-1.jdf

Filename            = wxh-237-Si-2.jdf
Author              = element
Experiment          = single_pulse_dec
Sample_Id           = S#365873
Solvent             = CHLOROFORM-D
Actual_Start_Time   = 23-APR-2022 16:54:58
Revision_Time       = 23-APR-2022 17:14:41

Comment             = single pulse decoupled ga
Data_Format         = 1D COMPLEX
Dim_Size            = 26214
X_Domain            = 29Si
Dim_Title           = 29Si
Dim_Units           = [ppm]
Dimensions          = X
Site                = ECS 400
Spectrometer        = JNM-ECS400

Field_Strength      = 9.20197068[T] (390[MHz])
X_Acq_Duration      = 1.34217728[s]
X_Domain            = 29Si
X_Freq              = 77.83692472[MHz]
X_Offset            = 0[ppm]
X_Points            = 32768
X_Prescans          = 4
X_Resolution        = 0.74505806[Hz]
X_Sweep             = 24.4140625[kHz]
Irr_Domain          = 1H
Irr_Freq            = 391.78655441[MHz]
Irr_Offset          = 5[ppm]
Clipped             = FALSE
Scans               = 600
Total_Scans         = 600

Relaxation_Delay    = 9[s]
Recvr_Gain          = 60
Temp_Get            = 18.4[dC]
X_90_Width          = 10[us]
X_Acq_Time          = 1.34217728[s]
X_Angle             = 30[deg]
X_Atn               = 4.9[dB]
X_Pulse             = 3.33333333[us]
Irr_Atn_Dec         = 22.45[dB]
Irr_Noise           = WALTZ
Decoupling          = TRUE
Initial_Wait        = 1[s]
Noe                 = FALSE
Repetition_Time     = 10.34217728[s]
```

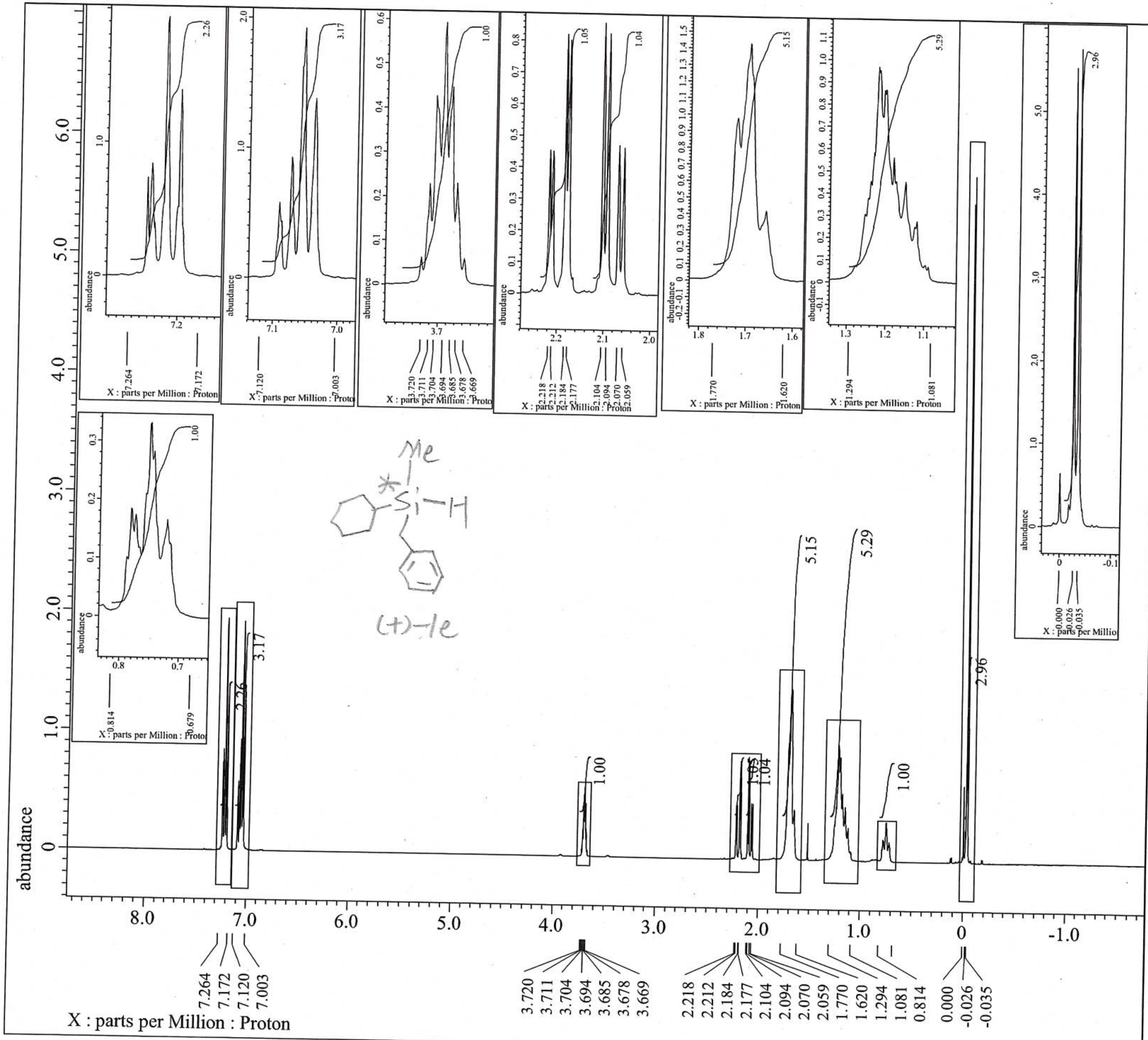


```
---- PROCESSING PARAMETERS ----
dc_balance( 0, FALSE )
sexp( 0.2[Hz], 0.0[s] )
trapezoid( 0[%], 0[%], 80[%], 100[%] )
zerofill( 1, TRUE )
fft( 1, TRUE, TRUE )
machinephase
ppm

数据来源: wxh-106-R_Proton-1-1.jdf

Filename            = wxh-106-R_Proton-1-4.jdf
Author              = element
Experiment          = proton.jxp
Sample_Id           = wxh-106-R
Solvent             = CHLOROFORM-D
Actual_Start_Time   = 16-DEC-2021 09:59:59
Revision_Time       = 27-JUN-2023 20:42:07

Comment             = single_pulse
Data_Format         = 1D COMPLEX
Dim_Size            = 13107
X_Domain            = Proton
Dim_Title           = Proton
Dim_Units           = [ppm]
Dimensions          = X
Spectrometer        = DELTA2_NMR

Field_Strength      = 9.4073814[T] (400[MHz])
X_Acq_Duration      = 2.18103808[s]
X_Domain            = 1H
X_Freq              = 400.53219825[MHz]
X_Offset            = 5[ppm]
X_Points            = 16384
X_Prescans          = 1
X_Resolution        = 0.45849727[Hz]
X_Sweep             = 7.51201923[kHz]
X_Sweep_Clipped     = 6.00961538[kHz]
Irr_Domain          = Proton
Irr_Freq            = 400.53219825[MHz]
Irr_Offset          = 5[ppm]
Tri_Domain          = Proton
Tri_Freq            = 400.53219825[MHz]
Tri_Offset          = 5[ppm]
Clipped             = FALSE
Scans               = 8
Total_Scans         = 8

Relaxation_Delay    = 5[s]
Recvr_Gain          = 26
Temp_Get            = 18.7[dC]
X_90_Width          = 6[us]
X_Acq_Time          = 2.18103808[s]
X_Angle             = 45[deg]
X_Atn               = 0.8[dB]
X_Pulse             = 3[us]
Irr_Mode            = Off
Tri_Mode            = Off
Dante_Presat        = FALSE
Initial_Wait        = 1[s]
Repetition_Time     = 7.18103808[s]
```

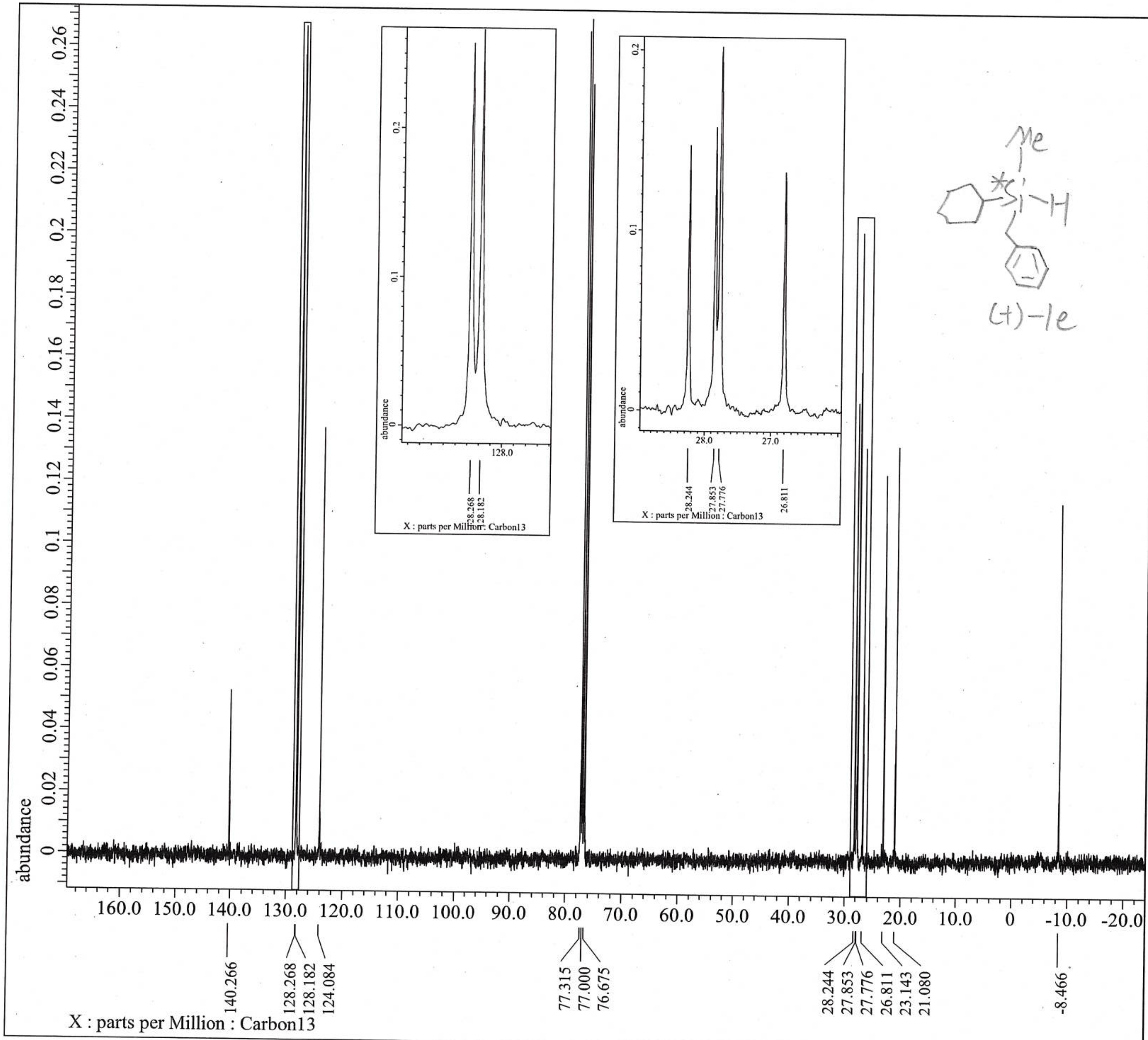

---- PROCESSING PARAMETERS ----
dc_balance(0, FALSE)
sexp(2.0[Hz], 0.0[s])
trapezoid(0[%], 0[%], 80[%], 100[%])
zerofill(1, TRUE)
fft(1, TRUE, TRUE)
machinephase
ppm

数据来源: wxh-106-R_Carbon-1-1.jdf

Filename	= wxh-106-R_Carbon-1-2.jdf
Author	= element
Experiment	= carbon.jxp
Sample_Id	= wxh-106-R
Solvent	= CHLOROFORM-D
Actual_Start_Time	= 16-DEC-2021 11:49:35
Revision_Time	= 6-JAN-2022 13:21:15
Comment	= single pulse decoupled ga
Data_Format	= 1D COMPLEX
Dim_Size	= 26214
X_Domain	= Carbon
Dim_Title	= Carbon13
Dim_Units	= [ppm]
Dimensions	= X
Site	= JNM-ECS400
Spectrometer	= DELTA2_NMR
Field_Strength	= 9.37221[T] (400[MHz])
X_Acq_Duration	= 1.04333312[s]
X_Domain	= 13C
X_Freq	= 100.33735165[MHz]
X_Offset	= 100.0[ppm]
X_Points	= 32768
X_Prescans	= 4
X_Resolution	= 0.95846665[Hz]
X_Sweep	= 31.40703518[kHz]
X_Sweep_Clipped	= 25.12562814[kHz]
Irr_Domain	= Proton
Irr_Freq	= 399.03472754[MHz]
Irr_Offset	= 5.0[ppm]
Clipped	= FALSE
Scans	= 256
Total_Scans	= 256
Relaxation_Delay	= 2[s]
Recvr_Gain	= 50
Temp_Get	= 20.3[dC]
X_90_Width	= 10.9[us]
X_Acq_Time	= 1.04333312[s]
X_Angle	= 30[deg]
X_Atn	= 5.4[dB]
X_Pulse	= 3.63333333[us]
Irr_Atn_Dec	= 25.823[dB]
Irr_Atn_Noe	= 25.823[dB]
Irr_Noise	= WALTZ
Irr_Pwidth	= 0.115[ms]
Decoupling	= TRUE
Initial_Wait	= 1[s]
Noe	= TRUE
Noe_Time	= 2[s]
Repetition_Time	= 3.04333312[s]

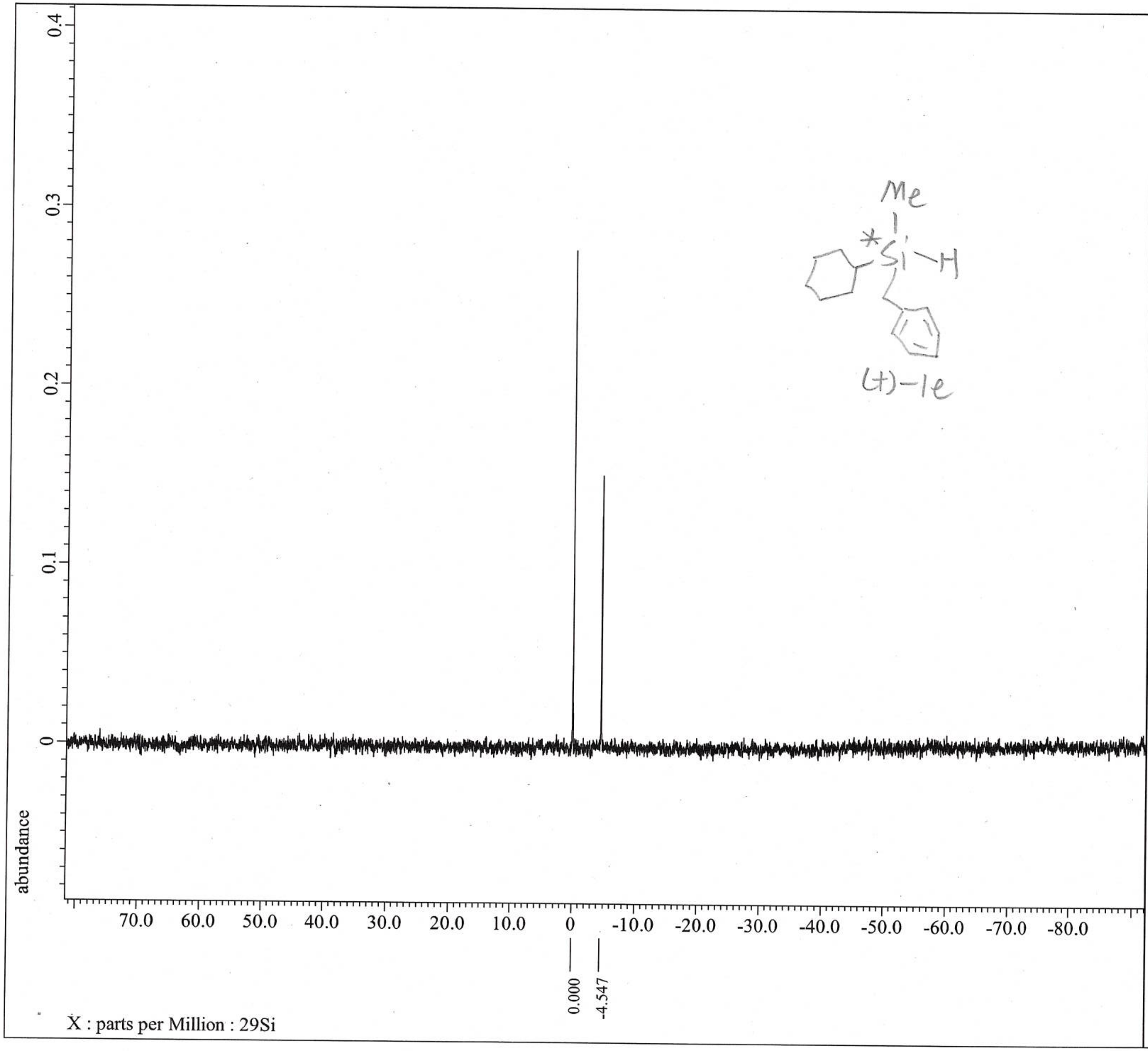


```
---- PROCESSING PARAMETERS ----
dc_balance( 0, FALSE )
sexp( 2.0[Hz], 0.0[s] )
trapezoid3( 0[%], 80[%], 100[%] )
zerofill( 1, TRUE )
fft( 1, TRUE, TRUE )
machinephase
ppm
phase( 59.15062, 0, 50[%] )

数据来源: wxh-106-Si-1.jdf

Filename            = wxh-106-Si-2.jdf
Author              = element
Experiment          = single_pulse_dec
Sample_Id           = S#490523
Solvent             = CHLOROFORM-D
Actual_Start_Time   = 24-AUG-2022 20:22:46
Revision_Time       = 21-JAN-2023 13:52:37

Comment             = single pulse decoupled ga
Data_Format         = 1D COMPLEX
Dim_Size            = 26214
X_Domain            = 29Si
Dim_Title           = 29Si
Dim_Units           = [ppm]
Dimensions          = X
Site                = ECS 400
Spectrometer        = JNM-ECS400

Field_Strength      = 9.20197068[T] (390[MHz])
X_Acq_Duration      = 1.34217728[s]
X_Domain            = 29Si
X_Freq              = 77.83692472[MHz]
X_Offset            = 0[ppm]
X_Points            = 32768
X_Prescans          = 4
X_Resolution        = 0.74505806[Hz]
X_Sweep             = 24.4140625[kHz]
Irr_Domain          = 1H
Irr_Freq            = 391.78655441[MHz]
Irr_Offset          = 5[ppm]
Clipped             = FALSE
Scans               = 560
Total_Scans         = 560

Relaxation_Delay    = 10[s]
Recvr_Gain          = 60
Temp_Get            = 21.3[dC]
X_90_Width          = 10[us]
X_Acq_Time          = 1.34217728[s]
X_Angle             = 30[deg]
X_Atn               = 4.9[dB]
X_Pulse             = 3.33333333[us]
Irr_Atn_Dec         = 22.05[dB]
Irr_Noise           = WALTZ
Decoupling          = TRUE
Initial_Wait        = 1[s]
Noe                 = FALSE
Repetition_Time     = 11.34217728[s]
```

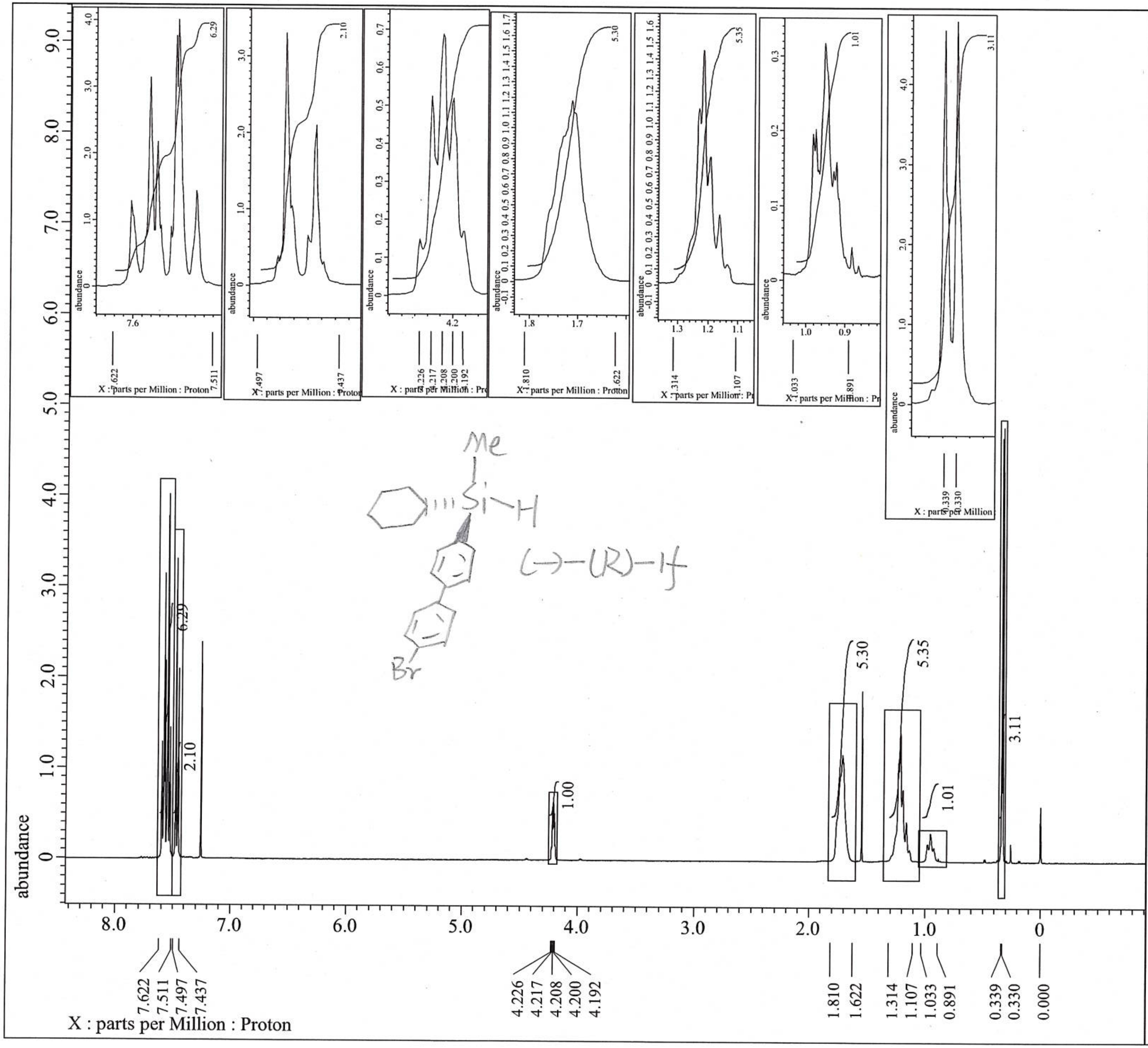

---- PROCESSING PARAMETERS ----
dc_balance(0, FALSE)
sexp(0.2[Hz], 0.0[s])
trapezoid(0[%], 0[%], 80[%], 100[%])
zerofill(1, TRUE)
fft(1, TRUE, TRUE)
machinephase
ppm

数据来源: wxh-128-R_Proton-1-1.jdf

Filename	= wxh-128-R_Proton-1-3.jdf
Author	= element
Experiment	= proton.jxp
Sample_Id	= wxh-128-R
Solvent	= CHLOROFORM-D
Actual_Start_Time	= 7-APR-2022 14:24:07
Revision_Time	= 27-JUN-2023 20:56:16
Comment	= single_pulse
Data_Format	= 1D COMPLEX
Dim_Size	= 13107
X_Domain	= Proton
Dim_Title	= Proton
Dim_Units	= [ppm]
Dimensions	= X
Spectrometer	= DELTA2_NMR
Field_Strength	= 9.4073814[T] (400[MHz])
X_Acq_Duration	= 2.18103808[s]
X_Domain	= 1H
X_Freq	= 400.53219825[MHz]
X_Offset	= 5[ppm]
X_Points	= 16384
X_Prescans	= 1
X_Resolution	= 0.45849727[Hz]
X_Sweep	= 7.51201923[kHz]
X_Sweep_Clipped	= 6.00961538[kHz]
Irr_Domain	= Proton
Irr_Freq	= 400.53219825[MHz]
Irr_Offset	= 5[ppm]
Tri_Domain	= Proton
Tri_Freq	= 400.53219825[MHz]
Tri_Offset	= 5[ppm]
Clipped	= FALSE
Scans	= 8
Total_Scans	= 8
Relaxation_Delay	= 5[s]
Recvr_Gain	= 38
Temp_Get	= 18.4[dC]
X_90_Width	= 6[us]
X_Acq_Time	= 2.18103808[s]
X_Angle	= 45[deg]
X_Atn	= 0.8[dB]
X_Pulse	= 3[us]
Irr_Mode	= Off
Tri_Mode	= Off
Dante_Presat	= FALSE
Initial_Wait	= 1[s]
Repetition_Time	= 7.18103808[s]

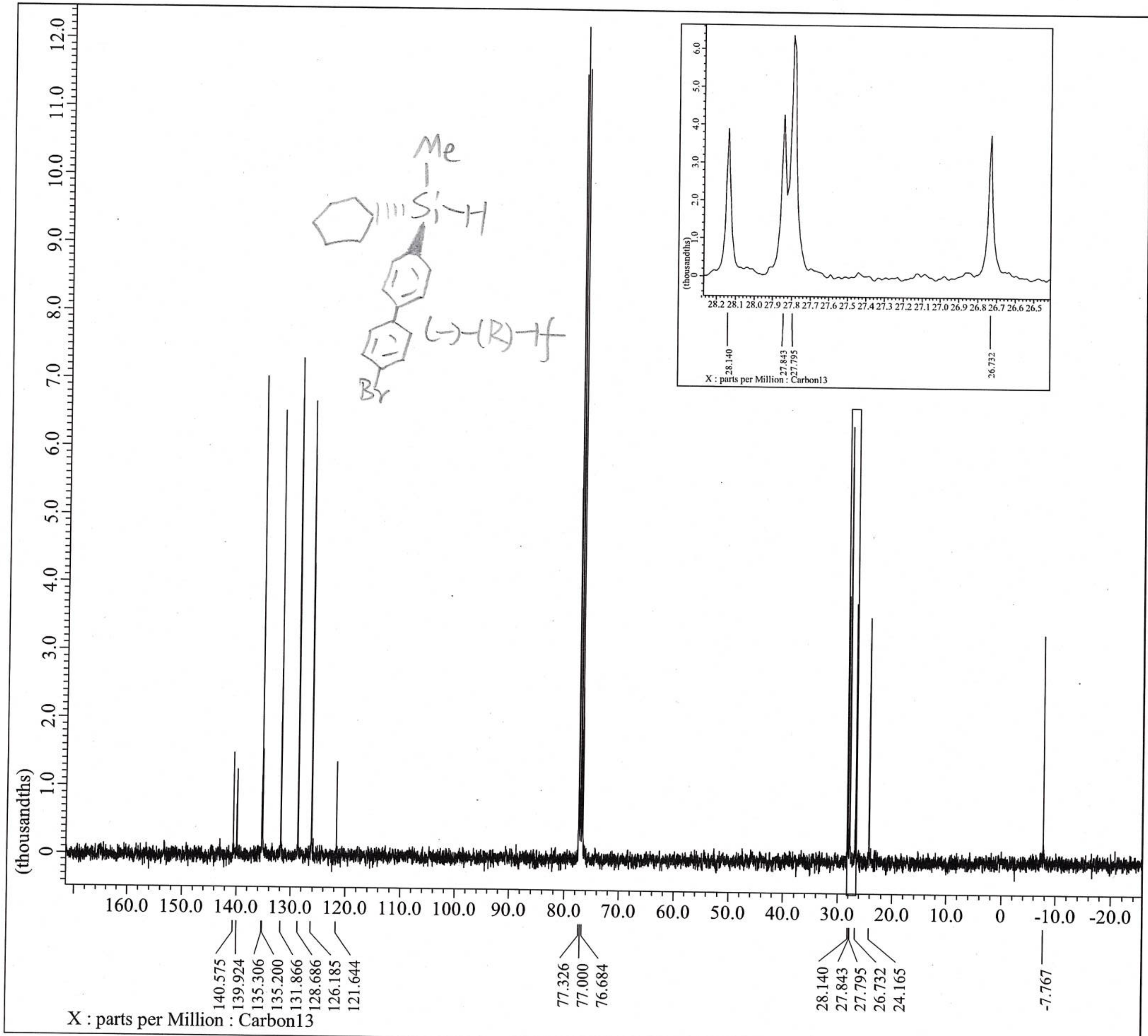


```
---- PROCESSING PARAMETERS ----
sexp( 2.0[Hz], 0.0[s] )
trapezoid( 0[%], 0[%], 80[%], 100[%] )
zerofill( 1, TRUE )
fft( 1, TRUE, TRUE )
machinephase
ppm

数据来源: wxh-128-R_Carbon-1-1.jdf
```

```
Filename                    = wxh-128-R_Carbon-1
Author                      = element
Experiment                  = carbon_auto.jxp
Sample_Id                   = wxh-128-R
Solvent                     = CHLOROFORM-D
Actual_Start_Time           =  7-APR-2022 14:15:
Revision_Time               = 25-APR-2022 11:41:

Comment                     = single pulse decou
Data_Format                 = 1D COMPLEX
Dim_Size                    = 26214
X_Domain                    = Carbon13
Dim_Title                   = Carbon13
Dim_Units                   = [ppm]
Dimensions                  = X
Spectrometer                = DELTA2_NMR

Field_Strength              = 9.2982153[T] (400[
X_Acq_Duration              = 1.048576[s]
X_Domain                    = Carbon13
X_Freq                      = 99.54517646[MHz]
X_Offset                    = 100[ppm]
X_Points                    = 32768
X_Prescans                  = 4
X_Resolution                = 0.95367432[Hz]
X_Sweep                     = 31.25[kHz]
X_Sweep_Clipped             = 25[kHz]
Irr_Domain                  = Proton
Irr_Freq                    = 395.88430144[MHz]
Irr_Offset                  = 5[ppm]
Blanking                    = 5.0[us]
Clipped                     = TRUE
Scans                       = 256
Total_Scans                 = 256

Relaxation_Delay            = 2[s]
Recvr_Gain                  = 50
Temp_Get                    = 18.8[dC]
X_90_Width                  = 9.65[us]
X_Acq_Time                  = 1.048576[s]
X_Angle                     = 30[deg]
X_Atn                       = 8[dB]
X_Pulse                     = 3.21666667[us]
Irr_Atn_Dec                 = 25.059[dB]
Irr_Atn_Dec_Calc            = 25.059[dB]
Irr_Atn_Dec_Default_Calc    = 25.059[dB]
Irr_Atn_Noe                 = 25.059[dB]
Irr_Dec_Bandwidth_Hz        = 4.7826087[kHz]
Irr_Dec_Bandwidth_Ppm       = 12.08082432[ppm]
Irr_Dec_Freq                = 395.88430144[MHz]
Irr_Dec_Merit_Factor        = 2.2
Irr_Decoupling              = TRUE
Irr_Noe                     = TRUE
Irr_Noise                   = WALTZ
Irr_Offset_Default          = 5[ppm]
```

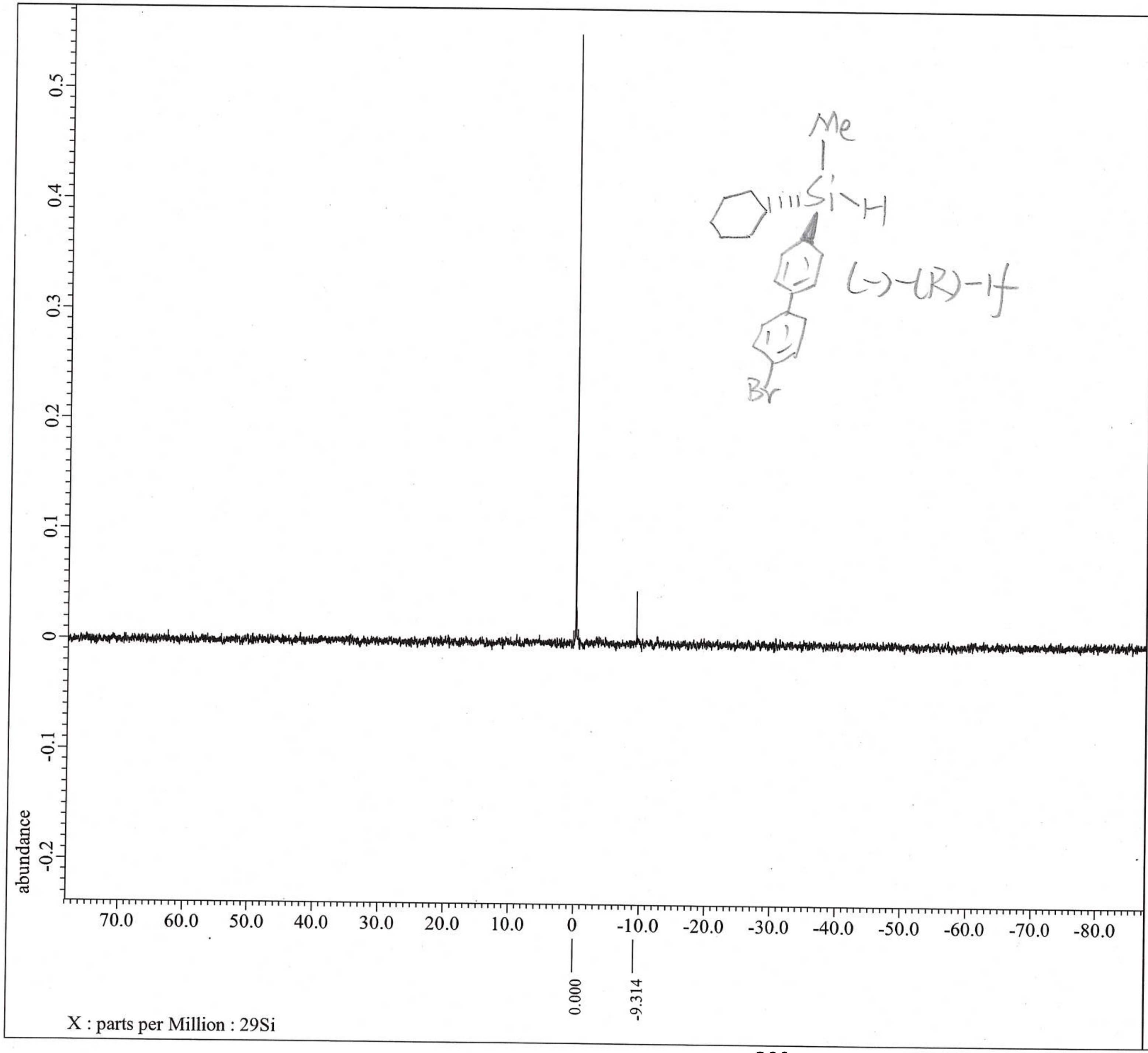


```
---- PROCESSING PARAMETERS ----
dc_balance( 0, FALSE )
sexp( 2.0[Hz], 0.0[s] )
trapezoid3( 0[%], 80[%], 100[%] )
zerofill( 1, TRUE )
fft( 1, TRUE, TRUE )
machinephase
ppm
phase( -69.58255, 0, 50[%] )

数据来源: wxh-128-R-Si-2-1.jdf
```

```
Filename            = wxh-128-R-Si-2-2.jdf
Author              = element
Experiment          = single_pulse_dec
Sample_Id           = S#445402
Solvent             = CHLOROFORM-D
Actual_Start_Time   =  8-APR-2022 19:10:10
Revision_Time       = 19-APR-2022 20:23:08

Comment             = single pulse decoupled ga
Data_Format         = 1D COMPLEX
Dim_Size            = 26214
X_Domain            = 29Si
Dim_Title           = 29Si
Dim_Units           = [ppm]
Dimensions          = X
Site                = ECS 400
Spectrometer        = JNM-ECS400

Field_Strength      = 9.20197068[T] (390[MHz])
X_Acq_Duration      = 1.34217728[s]
X_Domain            = 29Si
X_Freq              = 77.83692472[MHz]
X_Offset            = 0[ppm]
X_Points            = 32768
X_Prescans          = 4
X_Resolution        = 0.74505806[Hz]
X_Sweep             = 24.4140625[kHz]
Irr_Domain          = 1H
Irr_Freq            = 391.78655441[MHz]
Irr_Offset          = 5[ppm]
Clipped             = FALSE
Scans               = 600
Total_Scans         = 600

Relaxation_Delay    = 9[s]
Recvr_Gain          = 60
Temp_Get            = 20.1[dC]
X_90_Width          = 10[us]
X_Acq_Time          = 1.34217728[s]
X_Angle             = 30[deg]
X_Atn               = 4.9[dB]
X_Pulse             = 3.33333333[us]
Irr_Atn_Dec         = 22.45[dB]
Irr_Noise           = WALTZ
Decoupling          = TRUE
Initial_Wait        = 1[s]
Noe                 = FALSE
Repetition_Time     = 10.34217728[s]
```

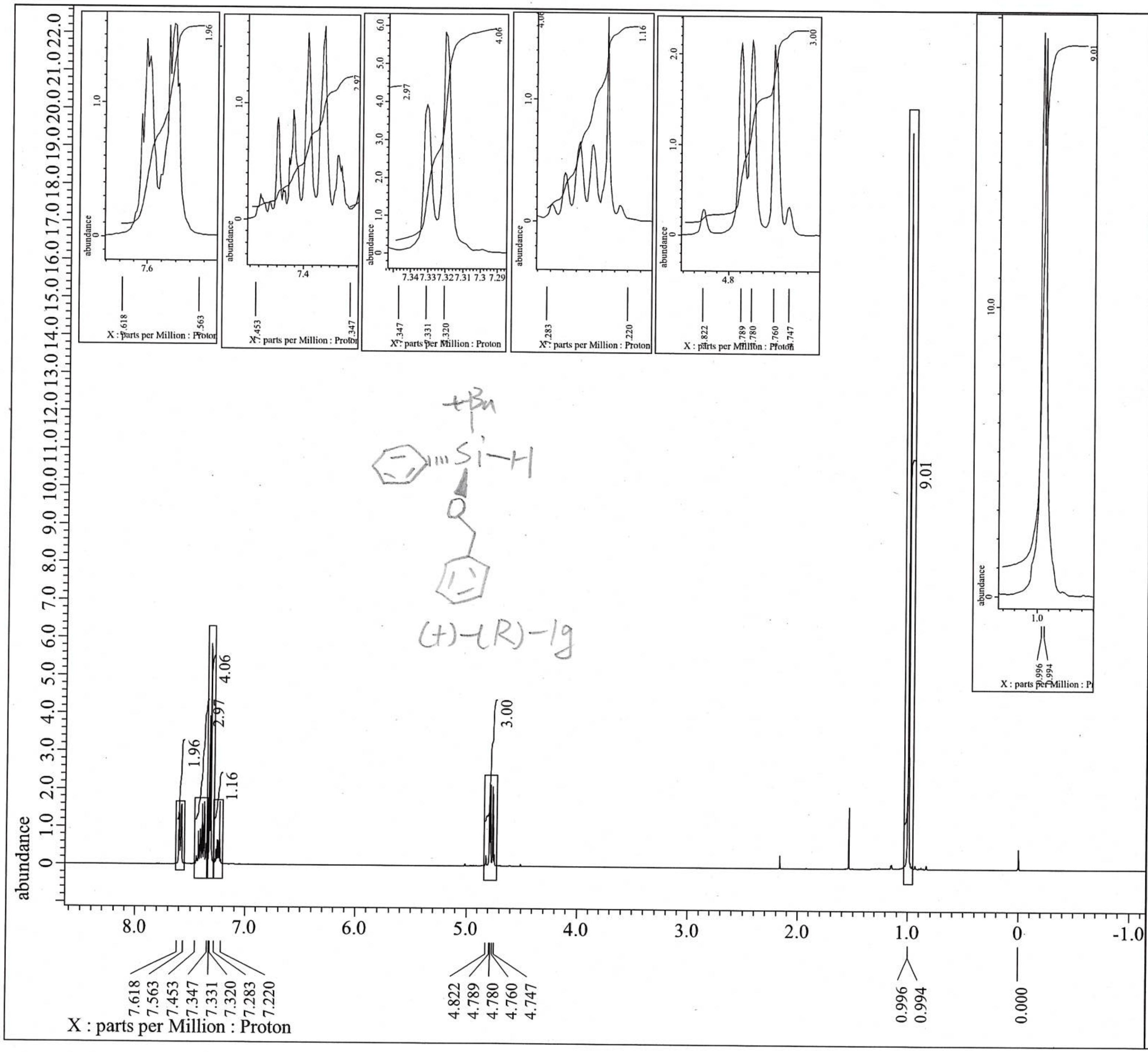


```
---- PROCESSING PARAMETERS ----
dc_balance( 0, FALSE )
sexp( 0.2[Hz], 0.0[s] )
trapezoid( 0[%], 0[%], 80[%], 100[%] )
zerofill( 1, TRUE )
fft( 1, TRUE, TRUE )
machinephase
ppm

数据来源: wxh-284-1_Proton-1-1.jdf

Filename            = wxh-284-1_Proton-1-3.jdf
Author              = element
Experiment          = proton.jxp
Sample_Id           = wxh-284-1
Solvent             = CHLOROFORM-D
Actual_Start_Time   = 29-OCT-2022 12:57:20
Revision_Time       = 28-JUN-2023 09:23:17

Comment             = single_pulse
Data_Format         = 1D COMPLEX
Dim_Size            = 13107
X_Domain            = Proton
Dim_Title           = Proton
Dim_Units           = [ppm]
Dimensions          = X
Site                = JNM-ECS400
Spectrometer        = DELTA2_NMR

Field_Strength      = 9.37221[T] (400[MHz])
X_Acq_Duration      = 2.1889024[s]
X_Domain            = 1H
X_Freq              = 399.03472754[MHz]
X_Offset            = 5.0[ppm]
X_Points            = 16384
X_Prescans          = 1
X_Resolution        = 0.45684997[Hz]
X_Sweep             = 7.48502994[kHz]
X_Sweep_Clipped     = 5.98802395[kHz]
Irr_Domain          = Proton
Irr_Freq            = 399.03472754[MHz]
Irr_Offset          = 5.0[ppm]
Tri_Domain          = Proton
Tri_Freq            = 399.03472754[MHz]
Tri_Offset          = 5.0[ppm]
Clipped             = FALSE
Scans               = 8
Total_Scans         = 8

Relaxation_Delay    = 5[s]
Recvr_Gain          = 30
Temp_Get            = 17.9[dC]
X_90_Width          = 6.6[us]
X_Acq_Time          = 2.1889024[s]
X_Angle             = 45[deg]
X_Atn               = 1[dB]
X_Pulse             = 3.3[us]
Irr_Mode            = Off
Tri_Mode            = Off
Dante_Presat        = FALSE
Initial_Wait        = 1[s]
Repetition_Time     = 7.1889024[s]
```

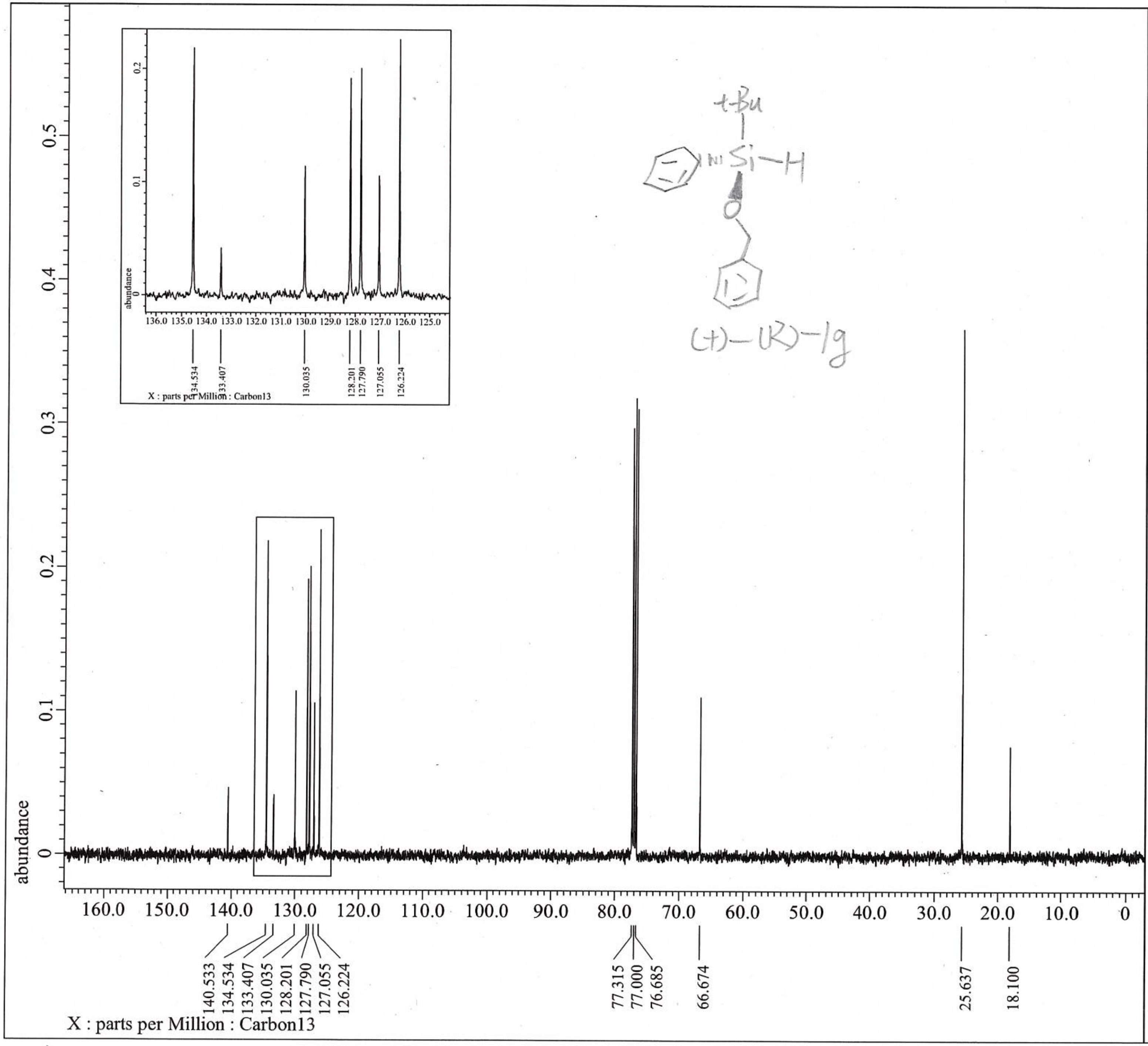


```
---- PROCESSING PARAMETERS ----
dc_balance( 0, FALSE )
sexp( 2.0[Hz], 0.0[s] )
trapezoid( 0[%], 0[%], 80[%], 100[%] )
zerofill( 1, TRUE )
fft( 1, TRUE, TRUE )
machinephase
ppm

数据来源: wxh-284-1_Carbon-1-1.jdf
```

```
Filename            = wxh-284-1_Carbon-1-2.jdf
Author              = element
Experiment          = carbon.jxp
Sample_Id           = wxh-284-1
Solvent             = CHLOROFORM-D
Actual_Start_Time   = 29-OCT-2022 12:58:40
Revision_Time       = 13-JAN-2023 11:57:11

Comment             = single pulse decoupled ga
Data_Format         = 1D COMPLEX
Dim_Size            = 26214
X_Domain            = Carbon
Dim_Title           = Carbon13
Dim_Units           = [ppm]
Dimensions          = X
Site                = JNM-ECS400
Spectrometer        = DELTA2_NMR

Field_Strength      = 9.37221[T] (400[MHz])
X_Acq_Duration      = 1.04333312[s]
X_Domain            = 13C
X_Freq              = 100.33735165[MHz]
X_Offset            = 100.0[ppm]
X_Points            = 32768
X_Prescans          = 4
X_Resolution        = 0.95846665[Hz]
X_Sweep             = 31.40703518[kHz]
X_Sweep_Clipped     = 25.12562814[kHz]
Irr_Domain          = Proton
Irr_Freq            = 399.03472754[MHz]
Irr_Offset          = 5.0[ppm]
Clipped             = FALSE
Scans               = 128
Total_Scans         = 128

Relaxation_Delay    = 2[s]
Recvr_Gain          = 50
Temp_Get            = 17.8[dC]
X_90_Width          = 10.9[us]
X_Acq_Time          = 1.04333312[s]
X_Angle             = 30[deg]
X_Atn               = 5.4[dB]
X_Pulse             = 3.63333333[us]
Irr_Atn_Dec         = 25.823[dB]
Irr_Atn_Noe         = 25.823[dB]
Irr_Noise           = WALTZ
Irr_Pwidth          = 0.115[ms]
Decoupling          = TRUE
Initial_Wait        = 1[s]
Noe                 = TRUE
Noe_Time            = 2[s]
Repetition_Time     = 3.04333312[s]
```

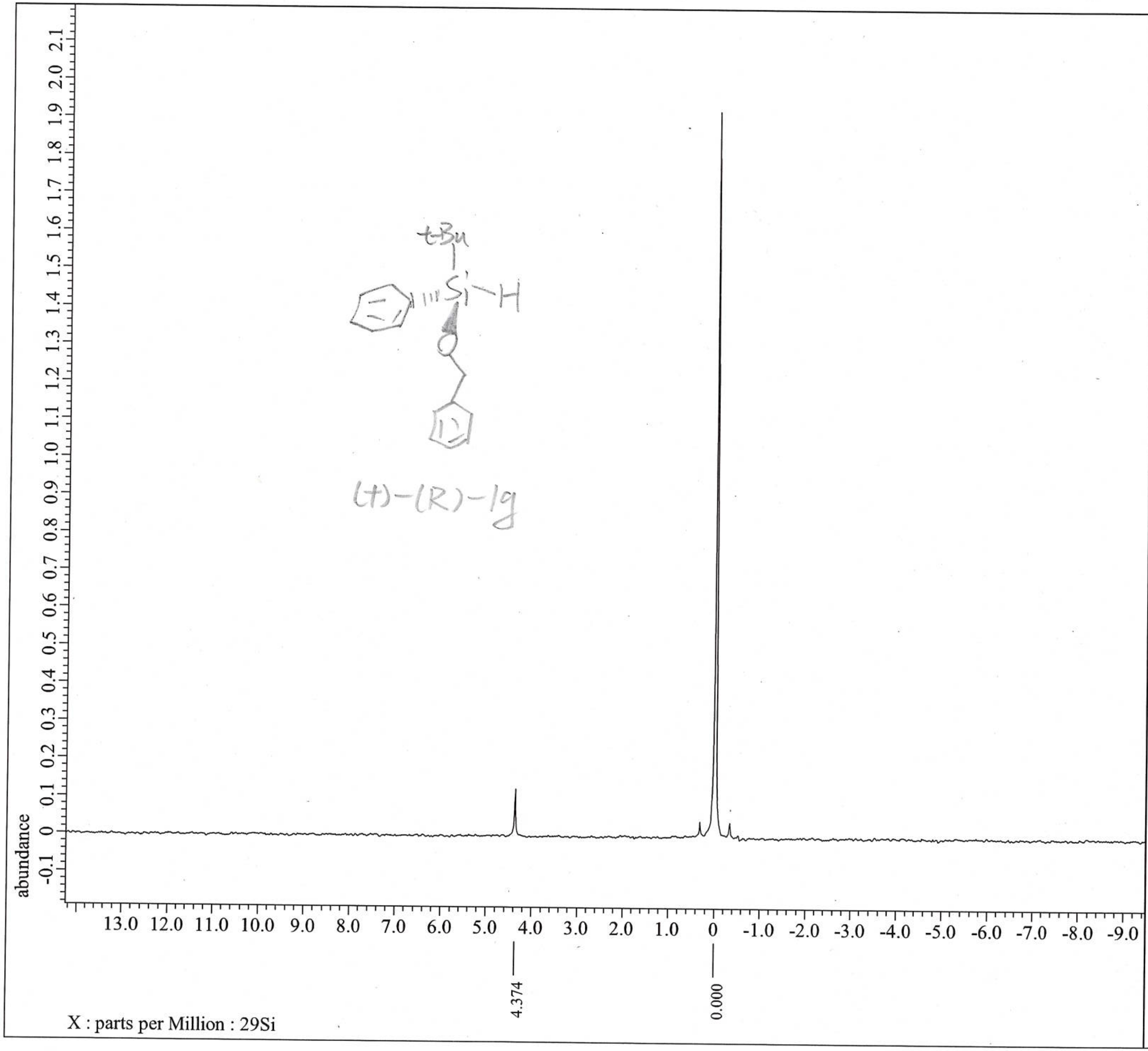


```
---- PROCESSING PARAMETERS ----
dc_balance( 0, FALSE )
sexp( 2.0[Hz], 0.0[s] )
trapezoid3( 0[%], 80[%], 100[%] )
zerofill( 1, TRUE )
fft( 1, TRUE, TRUE )
machinephase
ppm

数据来源: wxh-284-1-Si-3.jdf
```

```
Filename            = wxh-284-1-Si-4.jdf
Author              = element
Experiment          = single_pulse_dec
Sample_Id           = S#539982
Solvent             = CHLOROFORM-D
Actual_Start_Time   = 31-OCT-2022 21:44:54
Revision_Time       = 13-JAN-2023 11:49:52

Comment             = single pulse decoupled ga
Data_Format         = 1D COMPLEX
Dim_Size            = 26214
X_Domain            = 29Si
Dim_Title           = 29Si
Dim_Units           = [ppm]
Dimensions          = X
Site                = ECS 400
Spectrometer        = JNM-ECS400

Field_Strength      = 9.20197068[T] (390[MHz])
X_Acq_Duration      = 1.34217728[s]
X_Domain            = 29Si
X_Freq              = 77.83692472[MHz]
X_Offset            = 0[ppm]
X_Points            = 32768
X_Prescans          = 4
X_Resolution        = 0.74505806[Hz]
X_Sweep             = 24.4140625[kHz]
Irr_Domain          = 1H
Irr_Freq            = 391.78655441[MHz]
Irr_Offset          = 5[ppm]
Clipped             = FALSE
Scans               = 500
Total_Scans         = 500

Relaxation_Delay    = 10[s]
Recvr_Gain          = 58
Temp_Get            = 18[dC]
X_90_Width          = 10[us]
X_Acq_Time          = 1.34217728[s]
X_Angle             = 30[deg]
X_Atn               = 4.9[dB]
X_Pulse             = 3.33333333[us]
Irr_Atn_Dec         = 22.45[dB]
Irr_Noise           = WALTZ
Decoupling          = TRUE
Initial_Wait        = 1[s]
Noe                 = FALSE
Repetition_Time     = 11.34217728[s]
```

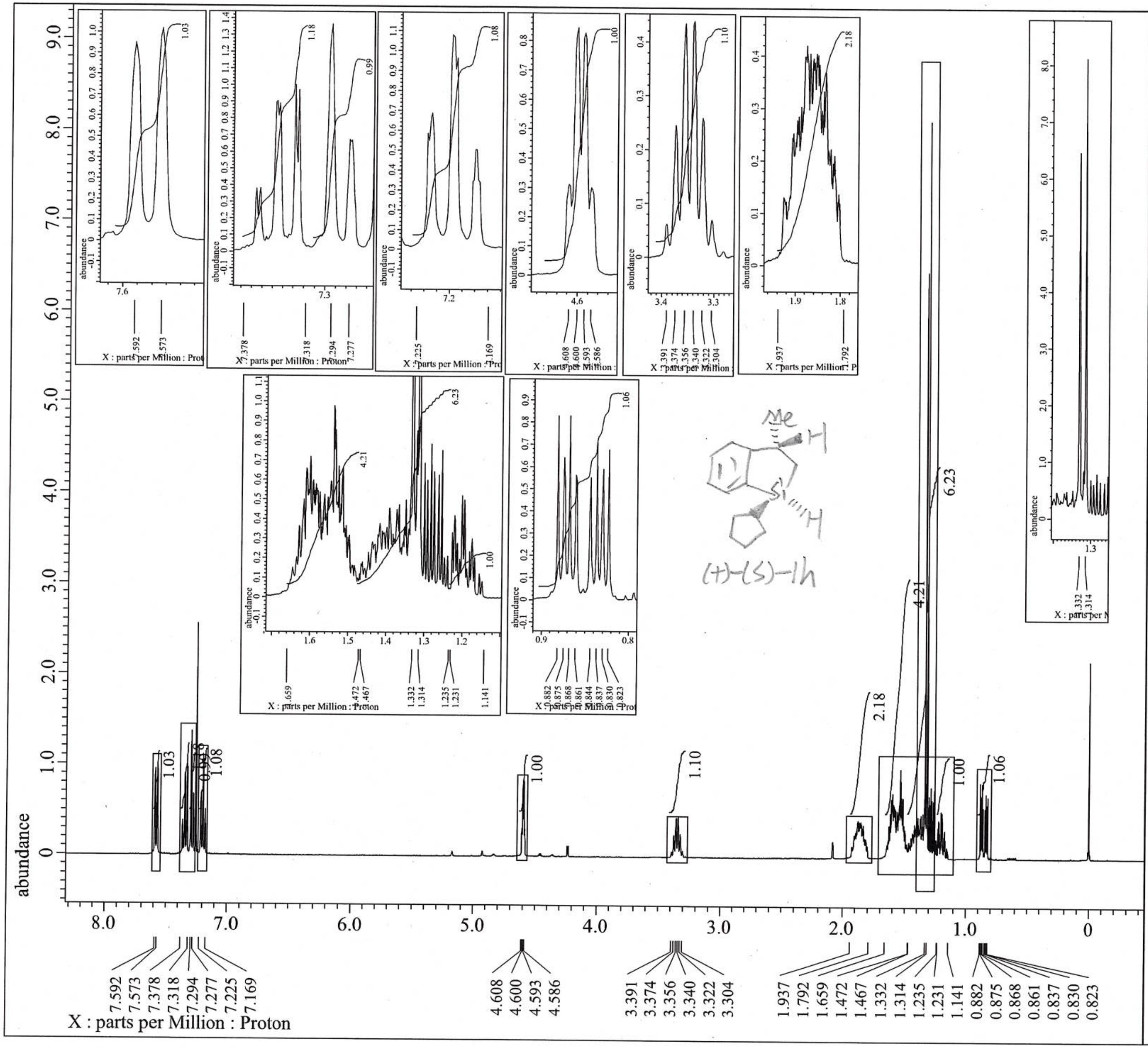


```
---- PROCESSING PARAMETERS ----
dc_balance( 0, FALSE )
sexp( 0.2[Hz], 0.0[s] )
trapezoid( 0[%], 0[%], 80[%], 100[%] )
zerofill( 1, TRUE )
fft( 1, TRUE, TRUE )
machinephase
ppm

数据来源: wxh-406-2_Proton-1-1.jdf

Filename            = wxh-406-2_Proton-1-2.jdf
Author              = element
Experiment          = proton.jxp
Sample_Id           = wxh-406-2
Solvent             = CHLOROFORM-D
Actual_Start_Time   =  7-FEB-2023 17:24:41
Revision_Time       = 28-JUN-2023 09:48:24

Comment             = single_pulse
Data_Format         = 1D COMPLEX
Dim_Size            = 13107
X_Domain            = Proton
Dim_Title           = Proton
Dim_Units           = [ppm]
Dimensions          = X
Site                = JNM-ECS400
Spectrometer        = DELTA2_NMR

Field_Strength      = 9.37221[T] (400[MHz])
X_Acq_Duration      = 2.1889024[s]
X_Domain            = 1H
X_Freq              = 399.03472754[MHz]
X_Offset            = 5.0[ppm]
X_Points            = 16384
X_Prescans          = 1
X_Resolution        = 0.45684997[Hz]
X_Sweep             = 7.48502994[kHz]
X_Sweep_Clipped     = 5.98802395[kHz]
Irr_Domain          = Proton
Irr_Freq            = 399.03472754[MHz]
Irr_Offset          = 5.0[ppm]
Tri_Domain          = Proton
Tri_Freq            = 399.03472754[MHz]
Tri_Offset          = 5.0[ppm]
Clipped             = FALSE
Scans               = 8
Total_Scans         = 8

Relaxation_Delay    = 5[s]
Recvr_Gain          = 34
Temp_Get            = 18.7[dC]
X_90_Width          = 6.6[us]
X_Acq_Time          = 2.1889024[s]
X_Angle             = 45[deg]
X_Atn               = 1[dB]
X_Pulse             = 3.3[us]
Irr_Mode            = Off
Tri_Mode            = Off
Dante_Presat        = FALSE
Initial_Wait        = 1[s]
Repetition_Time     = 7.1889024[s]
```

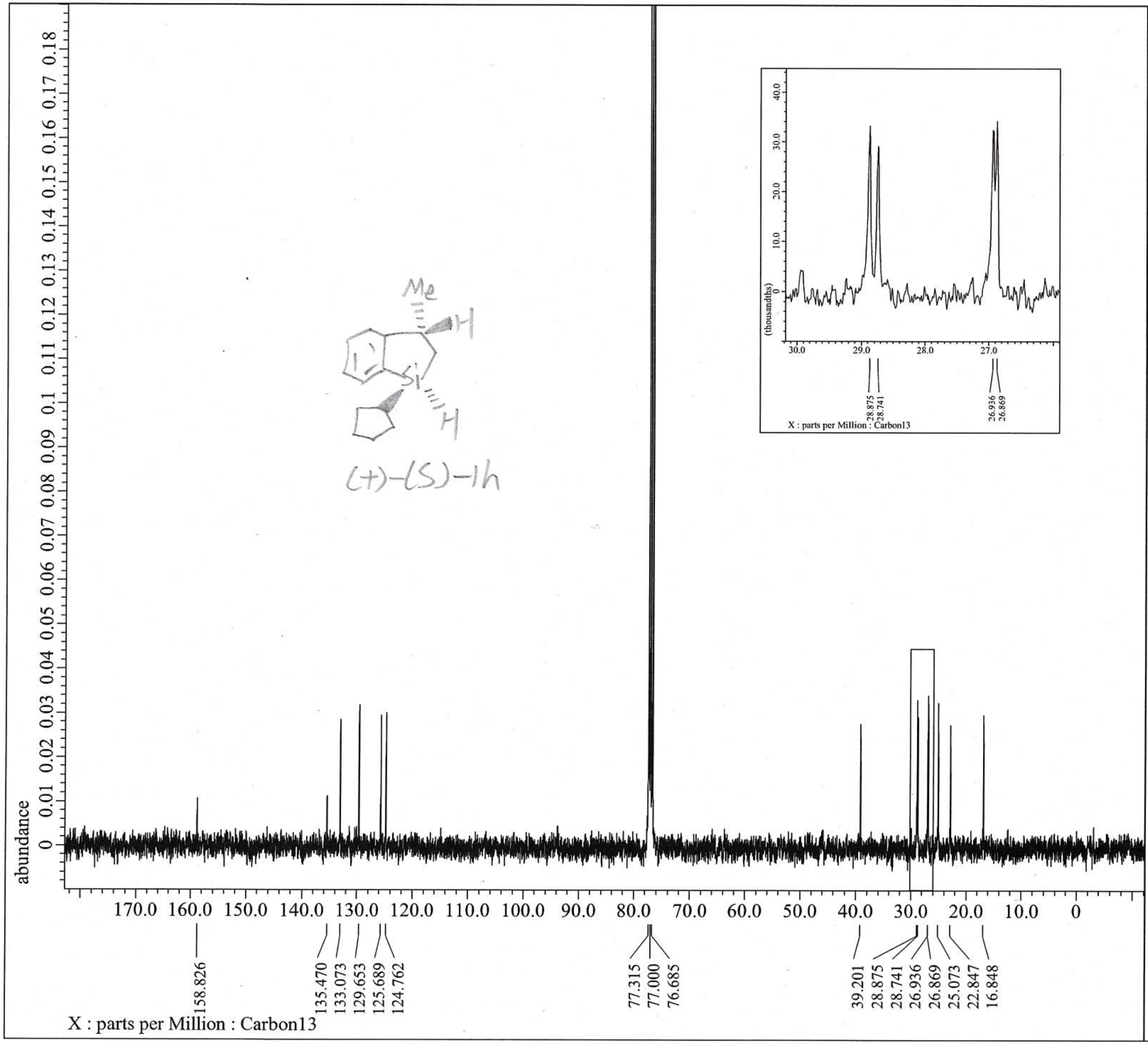


```
---- PROCESSING PARAMETERS ----
dc_balance( 0, FALSE )
sexp( 2.0[Hz], 0.0[s] )
trapezoid( 0[%], 0[%], 80[%], 100[%] )
zerofill( 1, TRUE )
fft( 1, TRUE, TRUE )
machinephase
ppm

数据来源: wxh-406-2_Carbon-1-1.jdf

Filename            = wxh-406-2_Carbon-1-2.jdf
Author              = element
Experiment          = carbon.jxp
Sample_Id           = wxh-406-2
Solvent             = CHLOROFORM-D
Actual_Start_Time   =  7-FEB-2023 19:46:12
Revision_Time       = 10-FEB-2023 19:49:45

Comment             = single pulse decoupled ga
Data_Format         = 1D COMPLEX
Dim_Size            = 26214
X_Domain            = Carbon
Dim_Title           = Carbon13
Dim_Units           = [ppm]
Dimensions          = X
Site                = JNM-ECS400
Spectrometer        = DELTA2_NMR

Field_Strength      = 9.37221[T] (400[MHz])
X_Acq_Duration      = 1.04333312[s]
X_Domain            = 13C
X_Freq              = 100.33735165[MHz]
X_Offset            = 100.0[ppm]
X_Points            = 32768
X_Prescans          = 4
X_Resolution        = 0.95846665[Hz]
X_Sweep             = 31.40703518[kHz]
X_Sweep_Clipped     = 25.12562814[kHz]
Irr_Domain          = Proton
Irr_Freq            = 399.03472754[MHz]
Irr_Offset          = 5.0[ppm]
Clipped             = FALSE
Scans               = 256
Total_Scans         = 256

Relaxation_Delay    = 2[s]
Recvr_Gain          = 50
Temp_Get            = 18.2[dC]
X_90_Width          = 10.9[us]
X_Acq_Time          = 1.04333312[s]
X_Angle             = 30[deg]
X_Atn               = 5.4[dB]
X_Pulse             = 3.63333333[us]
Irr_Atn_Dec         = 25.823[dB]
Irr_Atn_Noe         = 25.823[dB]
Irr_Noise           = WALTZ
Irr_Pwidth          = 0.115[ms]
Decoupling          = TRUE
Initial_Wait        = 1[s]
Noe                 = TRUE
Noe_Time            = 2[s]
Repetition_Time     = 3.04333312[s]
```

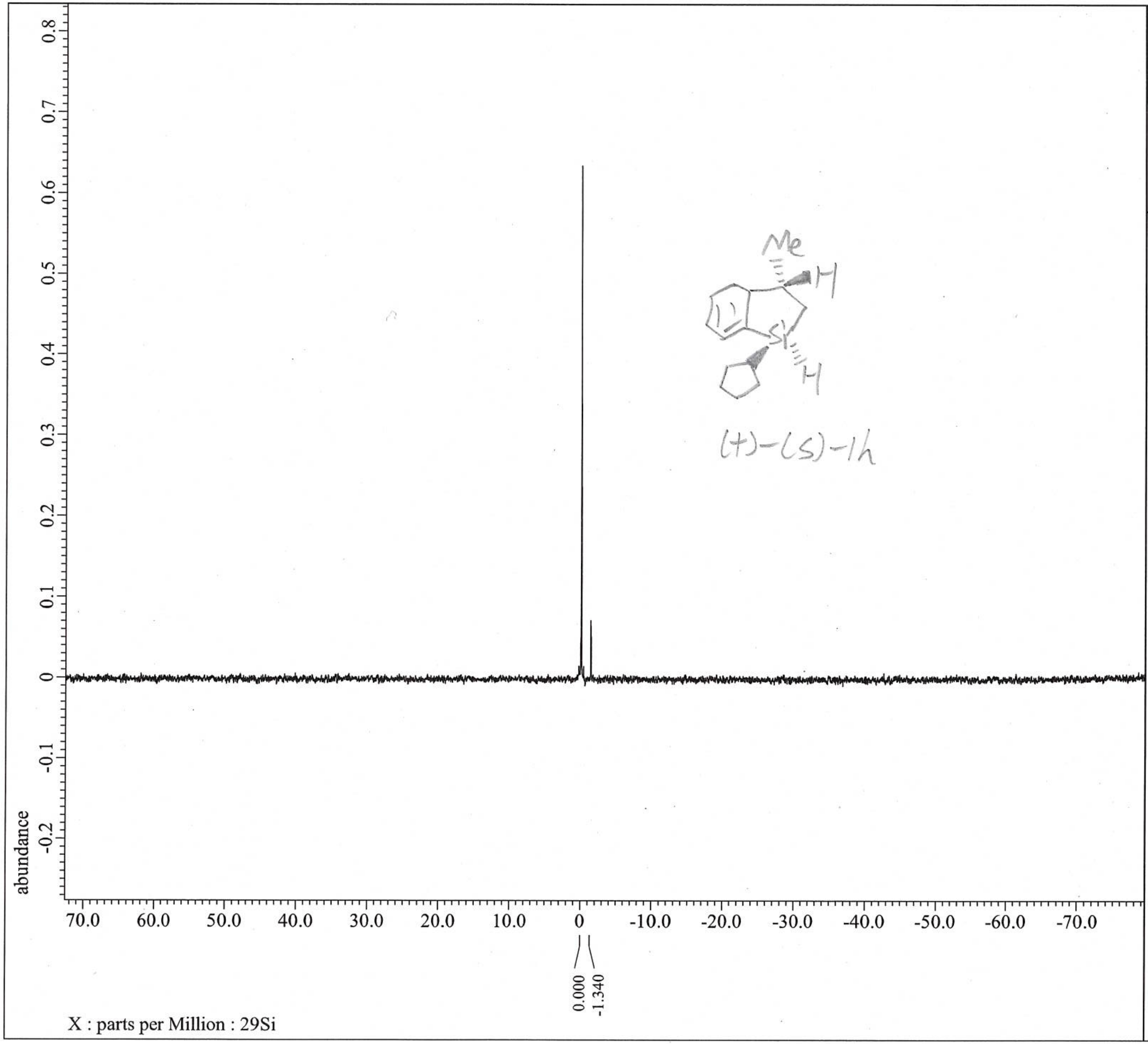

---- PROCESSING PARAMETERS ----
dc_balance(0, FALSE)
sexp(2.0[Hz], 0.0[s])
trapezoid3(0[%], 80[%], 100[%])
zerofill(1, TRUE)
fft(1, TRUE, TRUE)
machinephase
ppm

数据来源: wxh-406-Si-1.jdf

Filename	= wxh-406-Si-2.jdf
Author	= element
Experiment	= single_pulse_dec
Sample_Id	= S#670681
Solvent	= CHLOROFORM-D
Actual_Start_Time	= 9-FEB-2023 03:27:35
Revision_Time	= 10-FEB-2023 19:37:21
Comment	= single pulse decoupled ga
Data_Format	= 1D COMPLEX
Dim_Size	= 26214
X_Domain	= 29Si
Dim_Title	= 29Si
Dim_Units	= [ppm]
Dimensions	= X
Site	= ECS 400
Spectrometer	= JNM-ECS400
Field_Strength	= 9.20197068[T] (390[MHz])
X_Acq_Duration	= 1.34217728[s]
X_Domain	= 29Si
X_Freq	= 77.83692472[MHz]
X_Offset	= 0[ppm]
X_Points	= 32768
X_Prescans	= 4
X_Resolution	= 0.74505806[Hz]
X_Sweep	= 24.4140625[kHz]
Irr_Domain	= 1H
Irr_Freq	= 391.78655441[MHz]
Irr_Offset	= 5[ppm]
Clipped	= FALSE
Scans	= 500
Total_Scans	= 500
Relaxation_Delay	= 10[s]
Recvr_Gain	= 60
Temp_Get	= 18.1[dC]
X_90_Width	= 10[us]
X_Acq_Time	= 1.34217728[s]
X_Angle	= 30[deg]
X_Atn	= 4.9[dB]
X_Pulse	= 3.33333333[us]
Irr_Atn_Dec	= 22.45[dB]
Irr_Noise	= WALTZ
Decoupling	= TRUE
Initial_Wait	= 1[s]
Noe	= FALSE
Repetition_Time	= 11.34217728[s]

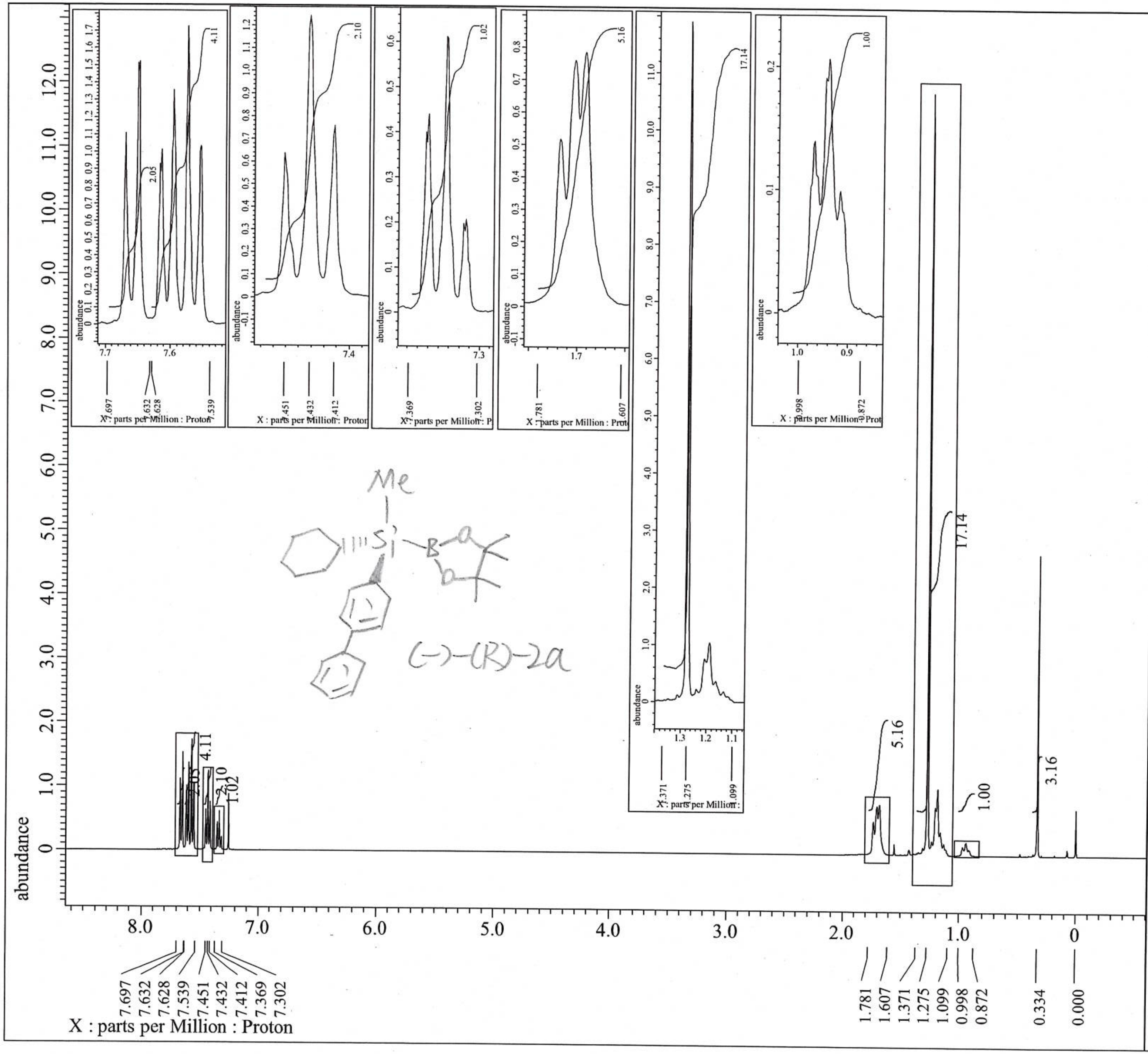

---- PROCESSING PARAMETERS ----
dc_balance(0, FALSE)
sexp(0.2[Hz], 0.0[s])
trapezoid(0[%], 0[%], 80[%], 100[%])
zerofill(1, TRUE)
fft(1, TRUE, TRUE)
machinephase
ppm

数据来源: wxh-138-1_Proton-1-1.jdf

Filename	= wxh-138-1_Proton-1-2.jdf
Author	= element
Experiment	= proton.jxp
Sample_Id	= wxh-138-1
Solvent	= CHLOROFORM-D
Actual_Start_Time	= 27-DEC-2021 14:16:48
Revision_Time	= 28-JUN-2023 10:34:40
Comment	= single_pulse
Data_Format	= 1D COMPLEX
Dim_Size	= 13107
X_Domain	= Proton
Dim_Title	= Proton
Dim_Units	= [ppm]
Dimensions	= X
Spectrometer	= DELTA2_NMR
Field_Strength	= 9.4073814[T] (400[MHz])
X_Acq_Duration	= 2.18103808[s]
X_Domain	= 1H
X_Freq	= 400.53219825[MHz]
X_Offset	= 5[ppm]
X_Points	= 16384
X_Prescans	= 1
X_Resolution	= 0.45849727[Hz]
X_Sweep	= 7.51201923[kHz]
X_Sweep_Clipped	= 6.00961538[kHz]
Irr_Domain	= Proton
Irr_Freq	= 400.53219825[MHz]
Irr_Offset	= 5[ppm]
Tri_Domain	= Proton
Tri_Freq	= 400.53219825[MHz]
Tri_Offset	= 5[ppm]
Clipped	= FALSE
Scans	= 8
Total_Scans	= 8
Relaxation_Delay	= 5[s]
Recvr_Gain	= 36
Temp_Get	= 18.7[dC]
X_90_Width	= 6[us]
X_Acq_Time	= 2.18103808[s]
X_Angle	= 45[deg]
X_Atn	= 0.8[dB]
X_Pulse	= 3[us]
Irr_Mode	= Off
Tri_Mode	= Off
Dante_Presat	= FALSE
Initial_Wait	= 1[s]
Repetition_Time	= 7.18103808[s]

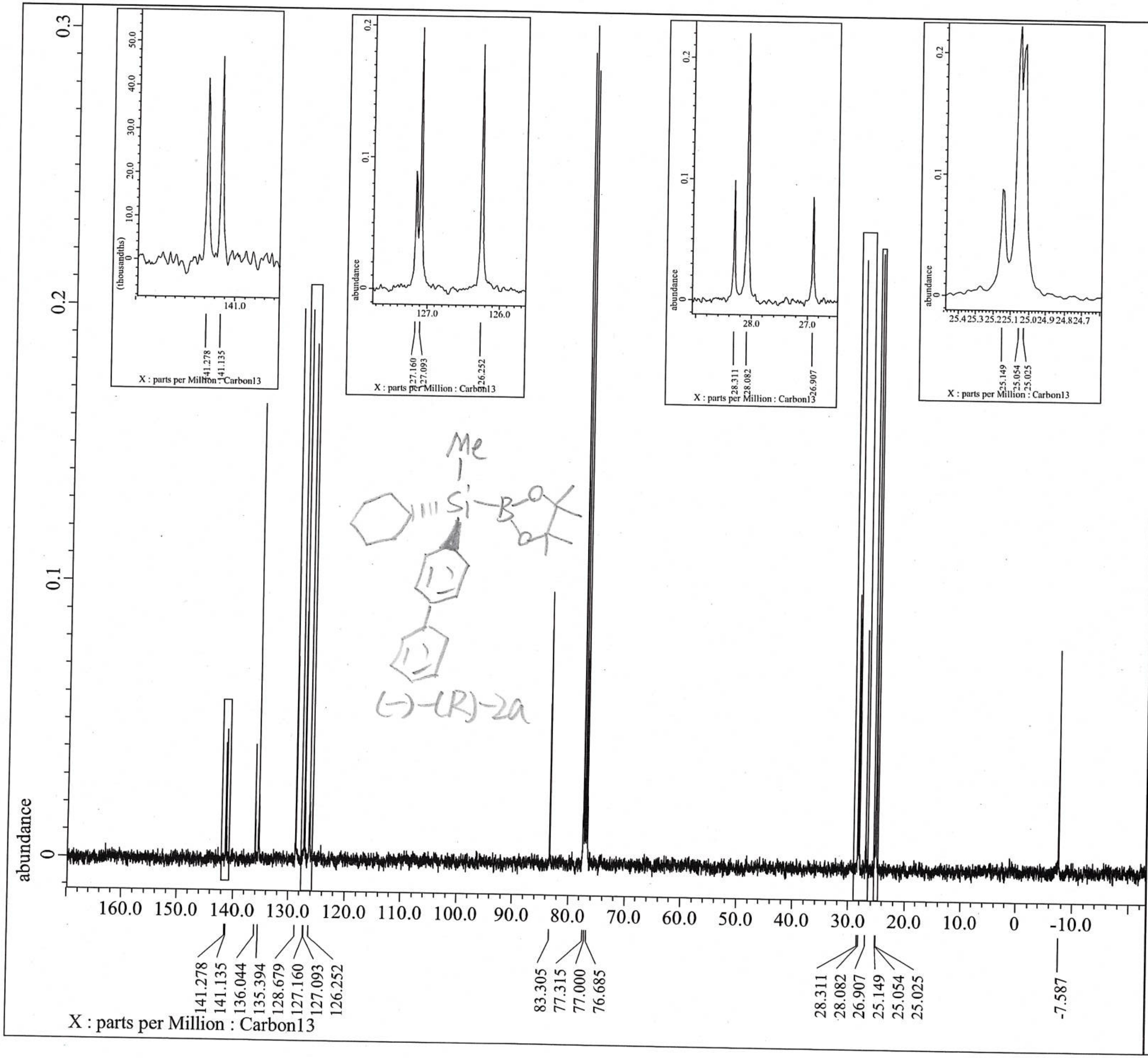


```
---- PROCESSING PARAMETERS ----
dc_balance( 0, FALSE )
sexp( 2.0[Hz], 0.0[s] )
trapezoid( 0[%], 0[%], 80[%], 100[%] )
zerofill( 1, TRUE )
fft( 1, TRUE, TRUE )
machinephase
ppm

数据来源: wxh-138-1_Carbon-1-1.jdf

Filename            = wxh-138-1_Carbon-1-2.jdf
Author              = element
Experiment          = carbon.jxp
Sample_Id           = wxh-138-1
Solvent             = CHLOROFORM-D
Actual_Start_Time   = 27-DEC-2021 14:57:35
Revision_Time       =  6-JAN-2022 10:48:44

Comment             = single pulse decoupled ga
Data_Format         = 1D COMPLEX
Dim_Size            = 26214
X_Domain            = Carbon
Dim_Title           = Carbon13
Dim_Units           = [ppm]
Dimensions          = X
Site                = JNM-ECS400
Spectrometer        = DELTA2_NMR

Field_Strength      = 9.37221[T] (400[MHz])
X_Acq_Duration      = 1.04333312[s]
X_Domain            = 13C
X_Freq              = 100.33735165[MHz]
X_Offset            = 100.0[ppm]
X_Points            = 32768
X_Prescans          = 4
X_Resolution        = 0.95846665[Hz]
X_Sweep             = 31.40703518[kHz]
X_Sweep_Clipped     = 25.12562814[kHz]
Irr_Domain          = Proton
Irr_Freq            = 399.03472754[MHz]
Irr_Offset          = 5.0[ppm]
Clipped             = FALSE
Scans               = 256
Total_Scans         = 256

Relaxation_Delay    = 2[s]
Recvr_Gain          = 50
Temp_Get            = 19.4[dC]
X_90_Width          = 10.9[us]
X_Acq_Time          = 1.04333312[s]
X_Angle             = 30[deg]
X_Atn               = 5.4[dB]
X_Pulse             = 3.63333333[us]
Irr_Atn_Dec         = 25.823[dB]
Irr_Atn_Noe         = 25.823[dB]
Irr_Noise           = WALTZ
Irr_Pwidth          = 0.115[ms]
Decoupling          = TRUE
Initial_Wait        = 1[s]
Noe                 = TRUE
Noe_Time            = 2[s]
Repetition_Time     = 3.04333312[s]
```

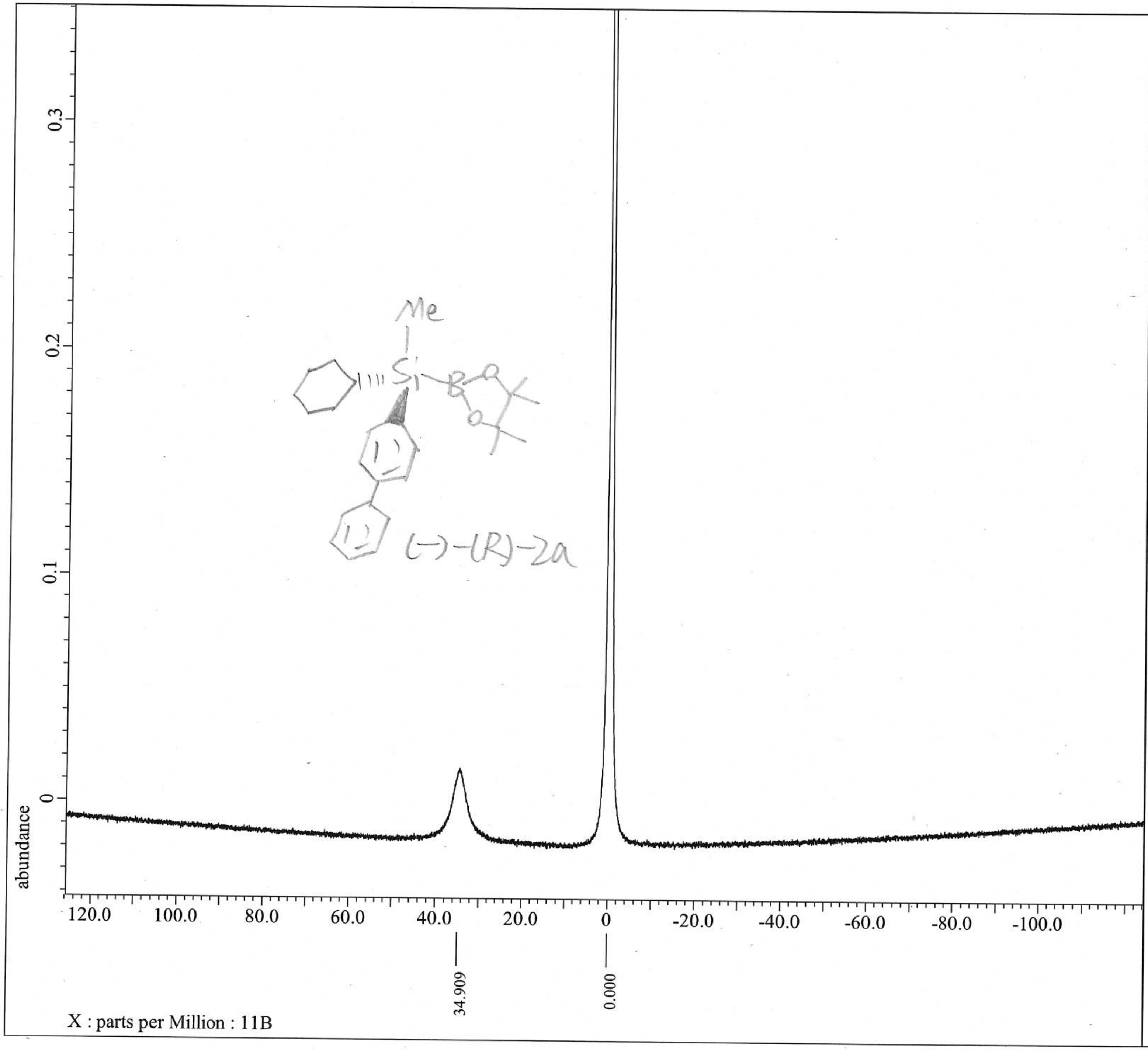

---- PROCESSING PARAMETERS ----
dc_balance(0, FALSE)
sexp(2.0[Hz], 0.0[s])
trapezoid3(0[%], 80[%], 100[%])
zerofill(1, TRUE)
fft(1, TRUE, TRUE)
machinephase
ppm

数据来源: wxh-138-B-1.jdf

Filename	= wxh-138-B-3.jdf
Author	= element
Experiment	= single_pulse_dec
Sample_Id	= S#566674
Solvent	= CHLOROFORM-D
Actual_Start_Time	= 5-JAN-2022 22:38:00
Revision_Time	= 11-JAN-2022 09:19:26
Comment	= single pulse decoupled ga
Data_Format	= 1D COMPLEX
Dim_Size	= 26214
X_Domain	= 11B
Dim_Title	= 11B
Dim_Units	= [ppm]
Dimensions	= X
Site	= ECS 400
Spectrometer	= JNM-ECS400
Field_Strength	= 9.20197068[T] (390[MHz])
X_Acq_Duration	= 0.83361792[s]
X_Domain	= 11B
X_Freq	= 125.70081325[MHz]
X_Offset	= 0[ppm]
X_Points	= 32768
X_Prescans	= 4
X_Resolution	= 1.19959034[Hz]
X_Sweep	= 39.3081761[kHz]
Irr_Domain	= 1H
Irr_Freq	= 391.78655441[MHz]
Irr_Offset	= 5[ppm]
Clipped	= FALSE
Scans	= 918
Total_Scans	= 918
Relaxation_Delay	= 2[s]
Recvr_Gain	= 46
Temp_Get	= 20.8[dC]
X_90_Width	= 10[us]
X_Acq_Time	= 0.83361792[s]
X_Angle	= 30[deg]
X_Atn	= 5.5[dB]
X_Pulse	= 3.33333333[us]
Irr_Atn_Dec	= 22.45[dB]
Irr_Atn_Noe	= 22.45[dB]
Irr_Noise	= WALTZ
Decoupling	= TRUE
Initial_Wait	= 1[s]
Noe	= TRUE
Noe_Time	= 2[s]
Repetition_Time	= 2.83361792[s]

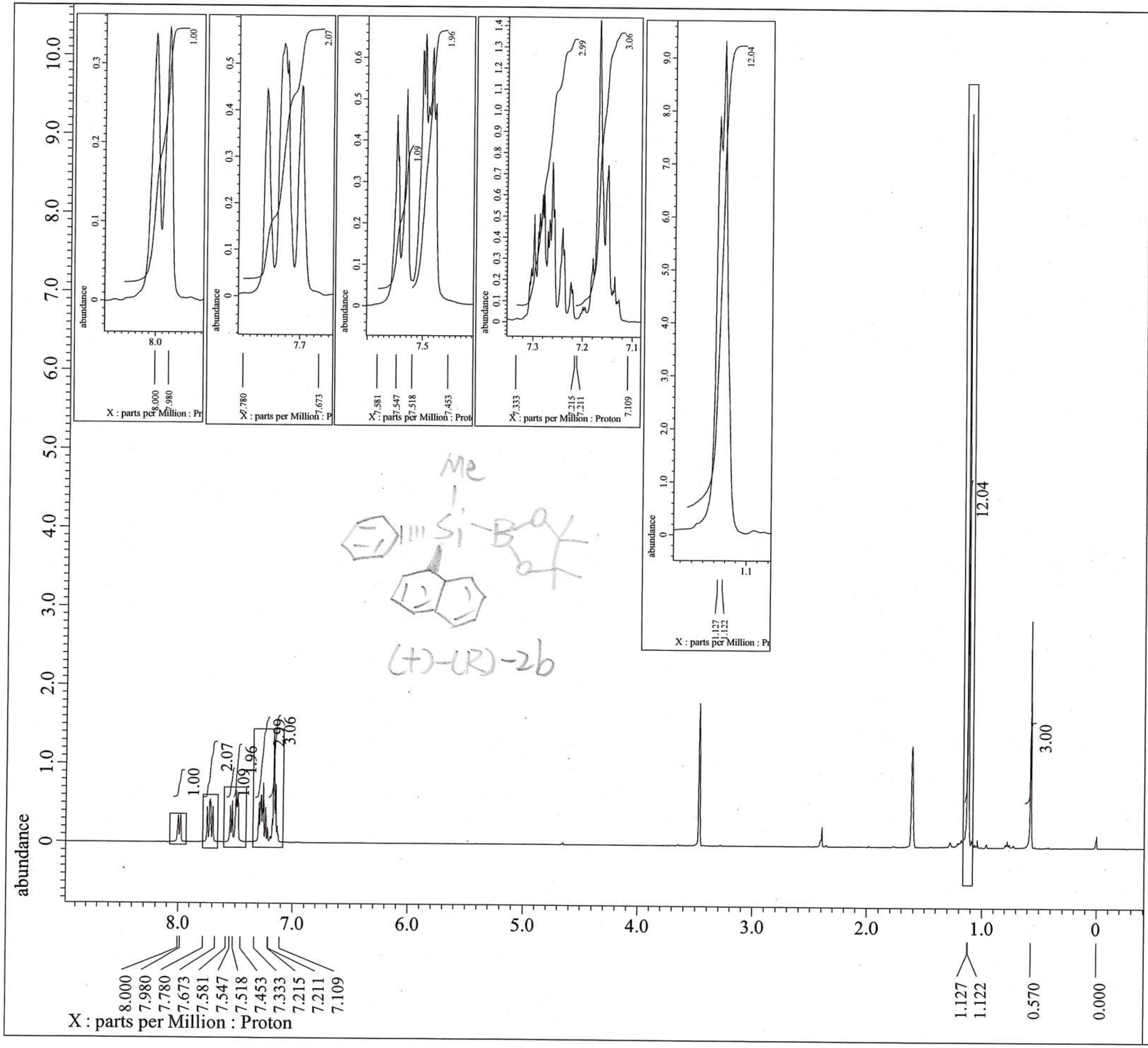

---- PROCESSING PARAMETERS ----
dc_balance(0, FALSE)
sexp(0.2[Hz], 0.0[s])
trapezoid(0[%], 0[%], 80[%], 100[%])
zerofill(1, TRUE)
fft(1, TRUE, TRUE)
machinephase
ppm

数据来源: wxh-224_Proton-1-1.jdf

Filename	= wxh-224_Proton-1-2.jdf
Author	= element
Experiment	= proton.jxp
Sample_Id	= wxh-224
Solvent	= TETRAHYDROFURAN-D8
Actual_Start_Time	= 17-FEB-2022 12:03:55
Revision_Time	= 28-JUN-2023 11:10:03
Comment	= single_pulse
Data_Format	= 1D COMPLEX
Dim_Size	= 13107
X_Domain	= Proton
Dim_Title	= Proton
Dim_Units	= [ppm]
Dimensions	= X
Site	= JNM-ECS400
Spectrometer	= DELTA2_NMR
Field_Strength	= 9.37221[T] (400[MHz])
X_Acq_Duration	= 2.1889024[s]
X_Domain	= 1H
X_Freq	= 399.03472754[MHz]
X_Offset	= 5.0[ppm]
X_Points	= 16384
X_Prescans	= 1
X_Resolution	= 0.45684997[Hz]
X_Sweep	= 7.48502994[kHz]
X_Sweep_Clipped	= 5.98802395[kHz]
Irr_Domain	= Proton
Irr_Freq	= 399.03472754[MHz]
Irr_Offset	= 5.0[ppm]
Tri_Domain	= Proton
Tri_Freq	= 399.03472754[MHz]
Tri_Offset	= 5.0[ppm]
Clipped	= FALSE
Scans	= 8
Total_Scans	= 8
Relaxation_Delay	= 5[s]
Recvr_Gain	= 30
Temp_Get	= 16.2[dC]
X_90_Width	= 6.6[us]
X_Acq_Time	= 2.1889024[s]
X_Angle	= 45[deg]
X_Atn	= 1[dB]
X_Pulse	= 3.3[us]
Irr_Mode	= Off
Tri_Mode	= Off
Dante_Presat	= FALSE
Initial_Wait	= 1[s]
Repetition_Time	= 7.1889024[s]

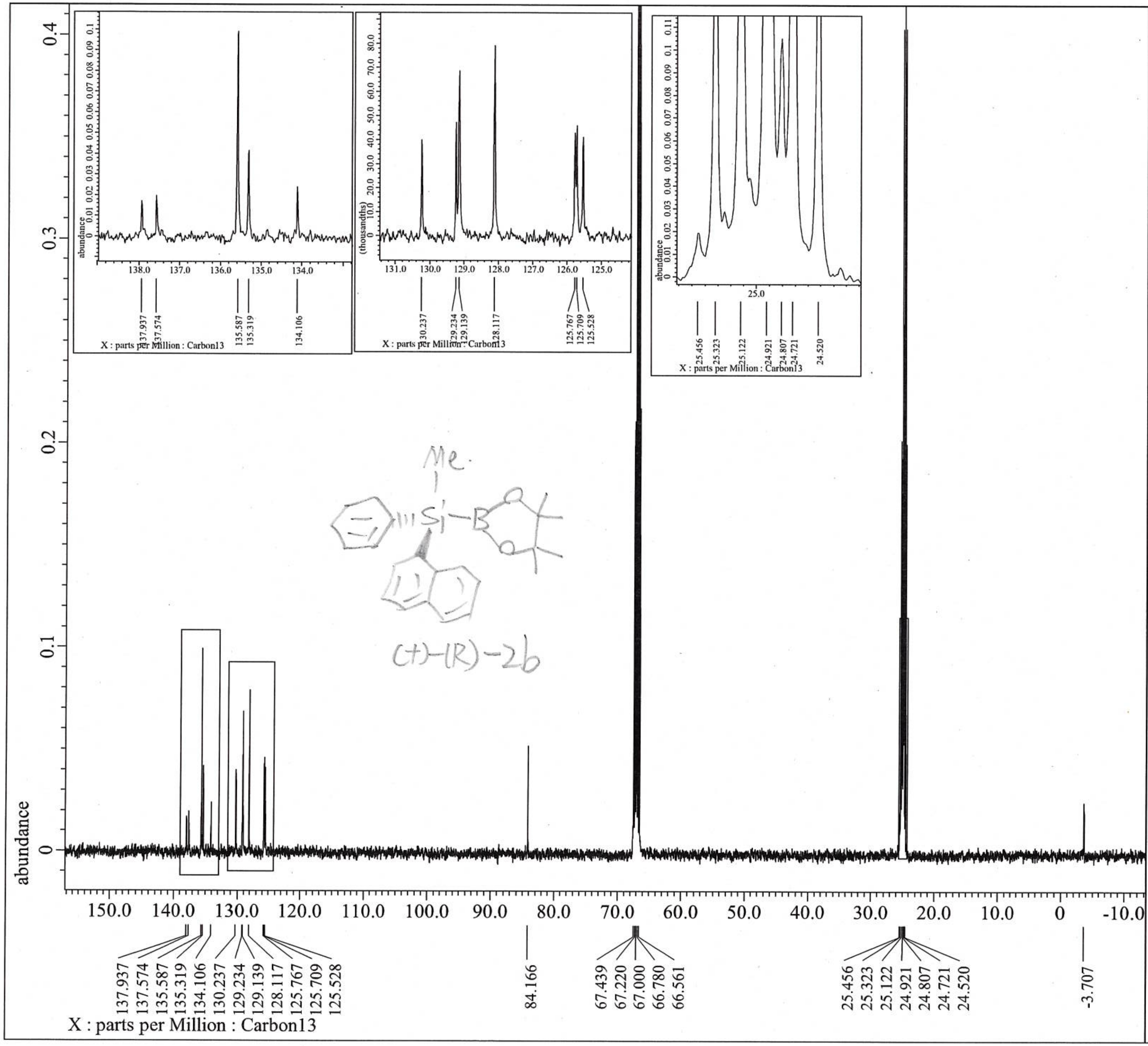


```
---- PROCESSING PARAMETERS ----
dc_balance( 0, FALSE )
sexp( 2.0[Hz], 0.0[s] )
trapezoid( 0[%], 0[%], 80[%], 100[%] )
zerofill( 1, TRUE )
fft( 1, TRUE, TRUE )
machinephase
ppm

数据来源: wxh-224_Carbon-1-1.jdf
```

```
Filename            = wxh-224_Carbon-1-2.jdf
Author              = element
Experiment          = carbon.jxp
Sample_Id           = wxh-224
Solvent             = TETRAHYDROFURAN-D8
Actual_Start_Time   = 17-FEB-2022 12:10:21
Revision_Time       = 20-APR-2022 16:21:44

Comment             = single pulse decoupled ga
Data_Format         = 1D COMPLEX
Dim_Size            = 26214
X_Domain            = Carbon
Dim_Title           = Carbon13
Dim_Units           = [ppm]
Dimensions          = X
Site                = JNM-ECS400
Spectrometer        = DELTA2_NMR

Field_Strength      = 9.37221[T] (400[MHz])
X_Acq_Duration      = 1.04333312[s]
X_Domain            = 13C
X_Freq              = 100.33735165[MHz]
X_Offset            = 100.0[ppm]
X_Points            = 32768
X_Prescans          = 4
X_Resolution        = 0.95846665[Hz]
X_Sweep             = 31.40703518[kHz]
X_Sweep_Clipped     = 25.12562814[kHz]
Irr_Domain          = Proton
Irr_Freq            = 399.03472754[MHz]
Irr_Offset          = 5.0[ppm]
Clipped             = FALSE
Scans               = 256
Total_Scans         = 256

Relaxation_Delay    = 2[s]
Recvr_Gain          = 50
Temp_Get            = 16.4[dC]
X_90_Width          = 10.9[us]
X_Acq_Time          = 1.04333312[s]
X_Angle             = 30[deg]
X_Atn               = 5.4[dB]
X_Pulse             = 3.63333333[us]
Irr_Atn_Dec         = 25.823[dB]
Irr_Atn_Noe         = 25.823[dB]
Irr_Noise           = WALTZ
Irr_Pwidth          = 0.115[ms]
Decoupling          = TRUE
Initial_Wait        = 1[s]
Noe                 = TRUE
Noe_Time            = 2[s]
Repetition_Time     = 3.04333312[s]
```

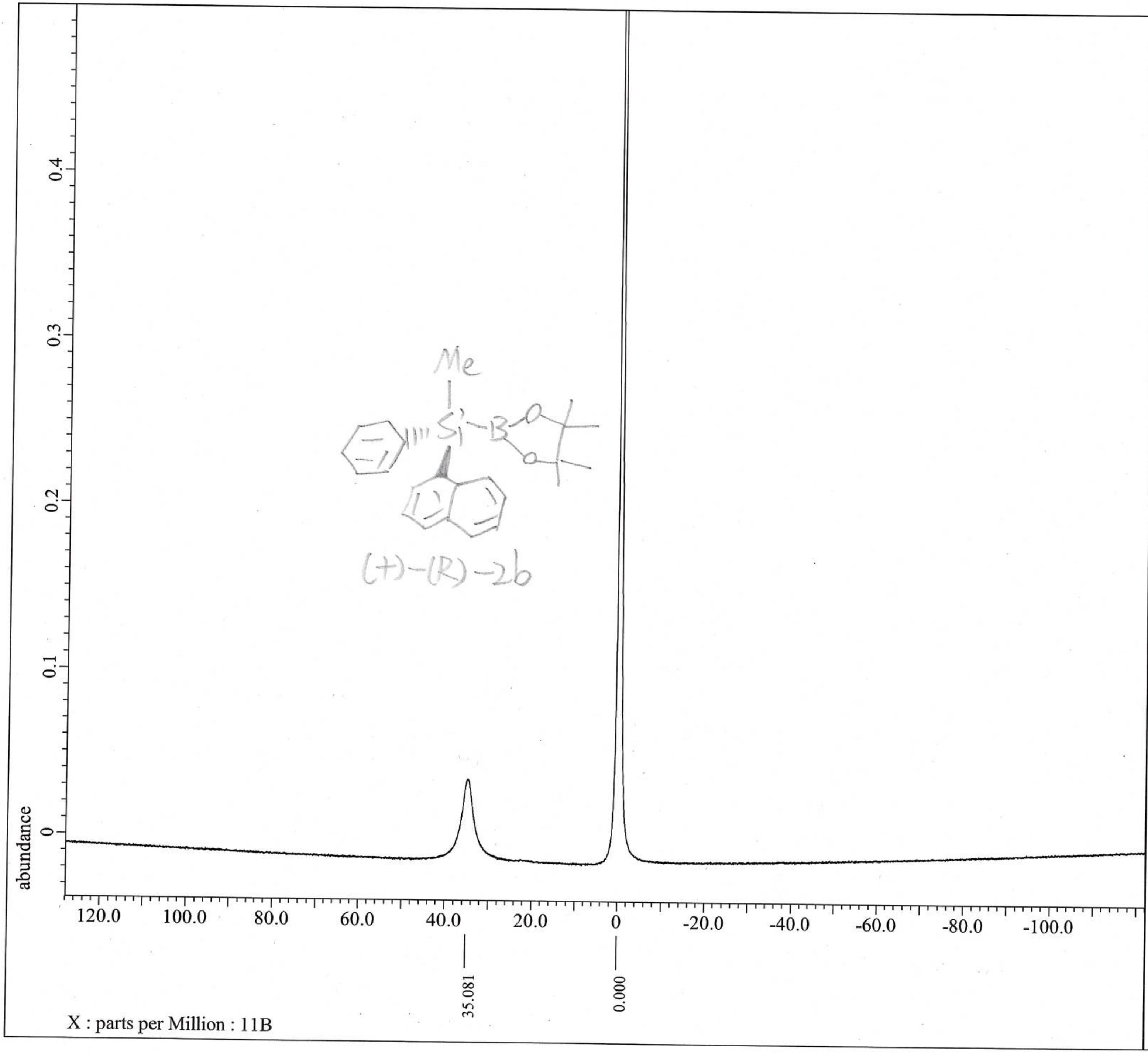

---- PROCESSING PARAMETERS ----
dc_balance(0, FALSE)
sexp(2.0[Hz], 0.0[s])
trapezoid3(0[%], 80[%], 100[%])
zerofill(1, TRUE)
fft(1, TRUE, TRUE)
machinephase
ppm

数据来源: WXH-224-B-1.jdf

Filename = WXH-224-B-2.jdf
Author = element
Experiment = single_pulse_dec
Sample_Id = S#670420
Solvent = TETRAHYDROFURAN-
Actual_Start_Time = 18-FEB-2022 01:26:11
Revision_Time = 20-APR-2022 17:11:10

Comment = single pulse decoupled ga
Data_Format = 1D COMPLEX
Dim_Size = 26214
X_Domain = 11B
Dim_Title = 11B
Dim_Units = [ppm]
Dimensions = X
Site = ECS 400
Spectrometer = JNM-ECS400

Field_Strength = 9.20197068[T] (390[MHz])
X_Acq_Duration = 0.83361792[s]
X_Domain = 11B
X_Freq = 125.70081325[MHz]
X_Offset = 0[ppm]
X_Points = 32768
X_Prescans = 4
X_Resolution = 1.19959034[Hz]
X_Sweep = 39.3081761[kHz]
Irr_Domain = 1H
Irr_Freq = 391.78655441[MHz]
Irr_Offset = 5[ppm]
Clipped = FALSE
Scans = 2200
Total_Scans = 2200

Relaxation_Delay = 2[s]
Recvr_Gain = 44
Temp_Get = 16.9[dC]
X_90_Width = 10[us]
X_Acq_Time = 0.83361792[s]
X_Angle = 30[deg]
X_Atn = 5.5[dB]
X_Pulse = 3.33333333[us]
Irr_Atn_Dec = 22.45[dB]
Irr_Atn_Noe = 22.45[dB]
Irr_Noise = WALTZ
Decoupling = TRUE
Initial_Wait = 1[s]
Noe = TRUE
Noe_Time = 2[s]
Repetition_Time = 2.83361792[s]

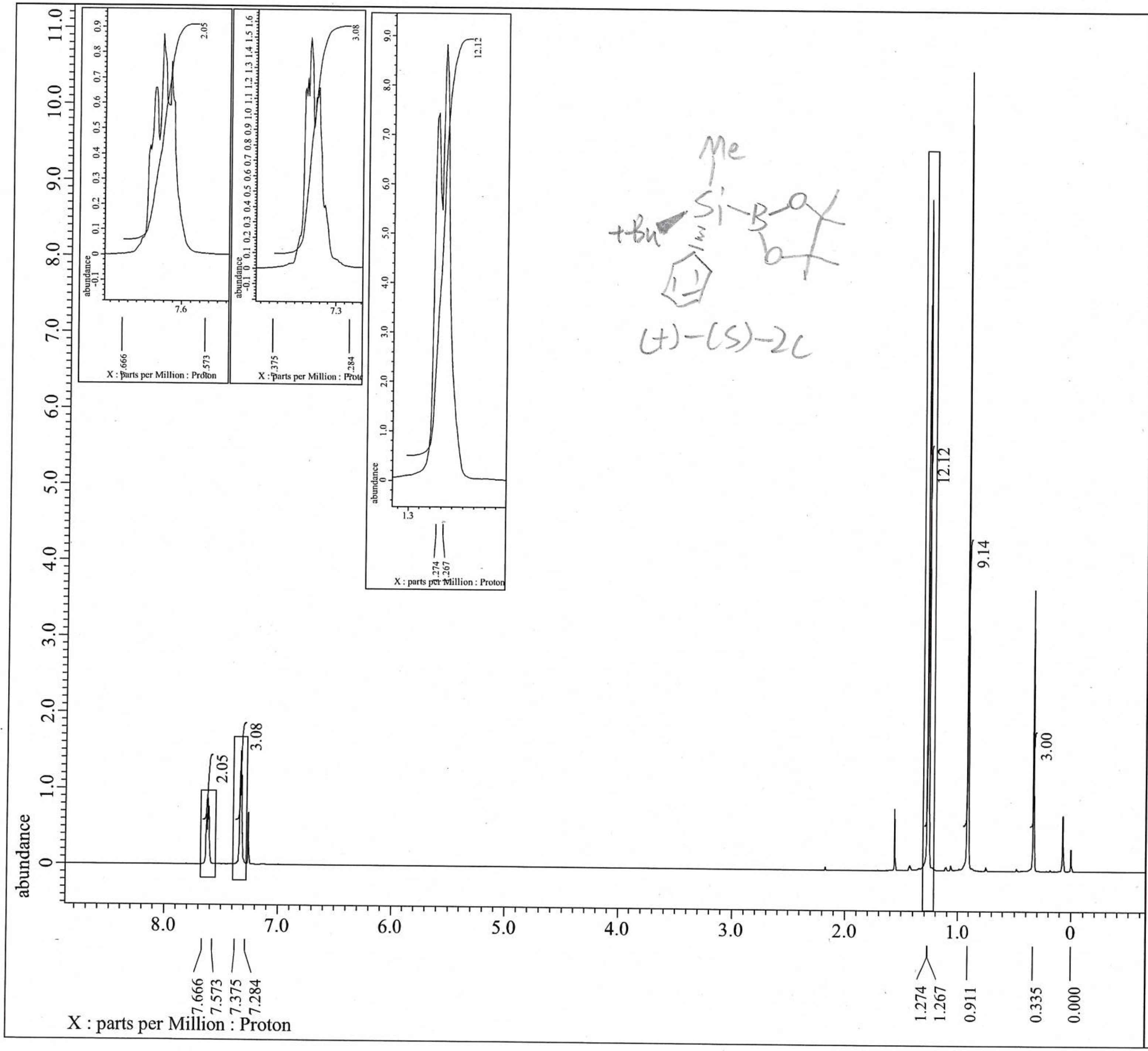

---- PROCESSING PARAMETERS ----
dc_balance(0, FALSE)
sexp(0.2[Hz], 0.0[s])
trapezoid(0[%], 0[%], 80[%], 100[%])
zerofill(1, TRUE)
fft(1, TRUE, TRUE)
machinephase
ppm

数据来源: wxh-184-2_Proton-1-1.jdf

Filename	= wxh-184-2_Proton-1-3.jdf
Author	= element
Experiment	= proton.jxp
Sample_Id	= wxh-184-1
Solvent	= CHLOROFORM-D
Actual_Start_Time	= 20-APR-2022 10:20:55
Revision_Time	= 28-JUN-2023 11:27:25
Comment	= single_pulse
Data_Format	= 1D COMPLEX
Dim_Size	= 13107
X_Domain	= Proton
Dim_Title	= Proton
Dim_Units	= [ppm]
Dimensions	= X
Site	= JNM-ECS400
Spectrometer	= DELTA2_NMR
Field_Strength	= 9.37221[T] (400[MHz])
X_Acq_Duration	= 2.1889024[s]
X_Domain	= 1H
X_Freq	= 399.03472754[MHz]
X_Offset	= 5.0[ppm]
X_Points	= 16384
X_Prescans	= 1
X_Resolution	= 0.45684997[Hz]
X_Sweep	= 7.48502994[kHz]
X_Sweep_Clipped	= 5.98802395[kHz]
Irr_Domain	= Proton
Irr_Freq	= 399.03472754[MHz]
Irr_Offset	= 5.0[ppm]
Tri_Domain	= Proton
Tri_Freq	= 399.03472754[MHz]
Tri_Offset	= 5.0[ppm]
Clipped	= FALSE
Scans	= 8
Total_Scans	= 8
Relaxation_Delay	= 5[s]
Recvr_Gain	= 36
Temp_Get	= 19.7[dC]
X_90_Width	= 6.6[us]
X_Acq_Time	= 2.1889024[s]
X_Angle	= 45[deg]
X_Atn	= 1[dB]
X_Pulse	= 3.3[us]
Irr_Mode	= Off
Tri_Mode	= Off
Dante_Presat	= FALSE
Initial_Wait	= 1[s]
Repetition_Time	= 7.1889024[s]

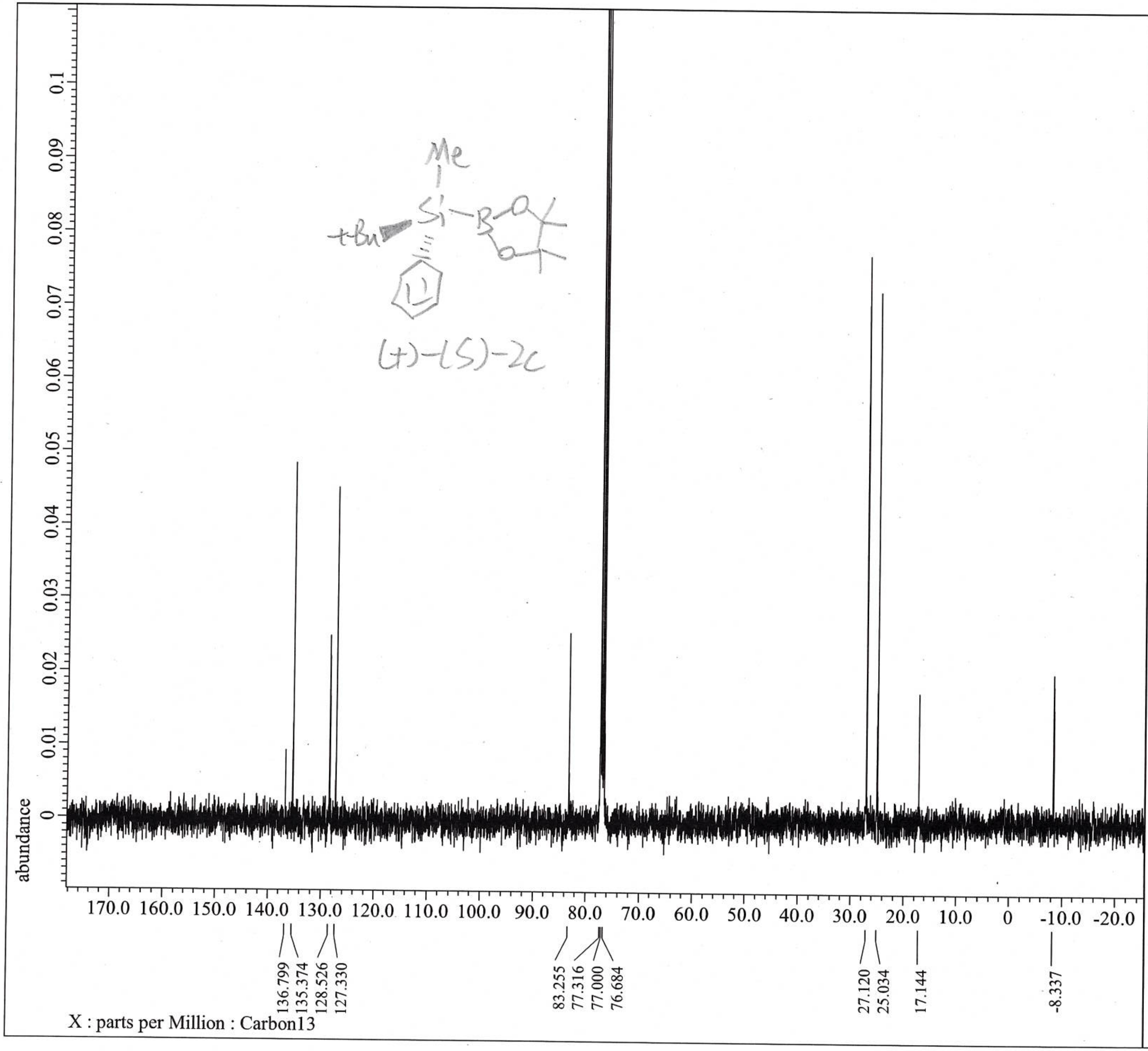


```
---- PROCESSING PARAMETERS ----
dc_balance( 0, FALSE )
sexp( 2.0[Hz], 0.0[s] )
trapezoid( 0[%], 0[%], 80[%], 100[%] )
zerofill( 1, TRUE )
fft( 1, TRUE, TRUE )
machinephase
ppm

数据来源: wxh-184-2_Carbon-1-1.jdf
```

```
Filename            = wxh-184-2_Carbon-1-2.jdf
Author              = element
Experiment          = carbon.jxp
Sample_Id           = wxh-184-2
Solvent             = CHLOROFORM-D
Actual_Start_Time   = 20-APR-2022 10:36:00
Revision_Time       = 22-APR-2022 15:17:30

Comment             = single pulse decoupled ga
Data_Format         = 1D COMPLEX
Dim_Size            = 26214
X_Domain            = Carbon
Dim_Title           = Carbon13
Dim_Units           = [ppm]
Dimensions          = X
Spectrometer        = DELTA2_NMR

Field_Strength      = 9.4073814[T] (400[MHz])
X_Acq_Duration      = 1.03809024[s]
X_Domain            = 13C
X_Freq              = 100.71389092[MHz]
X_Offset            = 100[ppm]
X_Points            = 32768
X_Prescans          = 4
X_Resolution        = 0.96330739[Hz]
X_Sweep             = 31.56565657[kHz]
X_Sweep_Clipped     = 25.25252525[kHz]
Irr_Domain          = Proton
Irr_Freq            = 400.53219825[MHz]
Irr_Offset          = 5[ppm]
Clipped             = FALSE
Scans               = 256
Total_Scans         = 256

Relaxation_Delay    = 2[s]
Recvr_Gain          = 50
Temp_Get            = 18.5[dC]
X_90_Width          = 10.9[us]
X_Acq_Time          = 1.03809024[s]
X_Angle             = 30[deg]
X_Atn               = 4[dB]
X_Pulse             = 3.63333333[us]
Irr_Atn_Dec         = 26.45[dB]
Irr_Atn_Noe         = 26.45[dB]
Irr_Noise           = WALTZ
Irr_Pwidth          = 0.115[ms]
Decoupling          = TRUE
Initial_Wait        = 1[s]
Noe                 = TRUE
Noe_Time            = 2[s]
Repetition_Time     = 3.03809024[s]
```

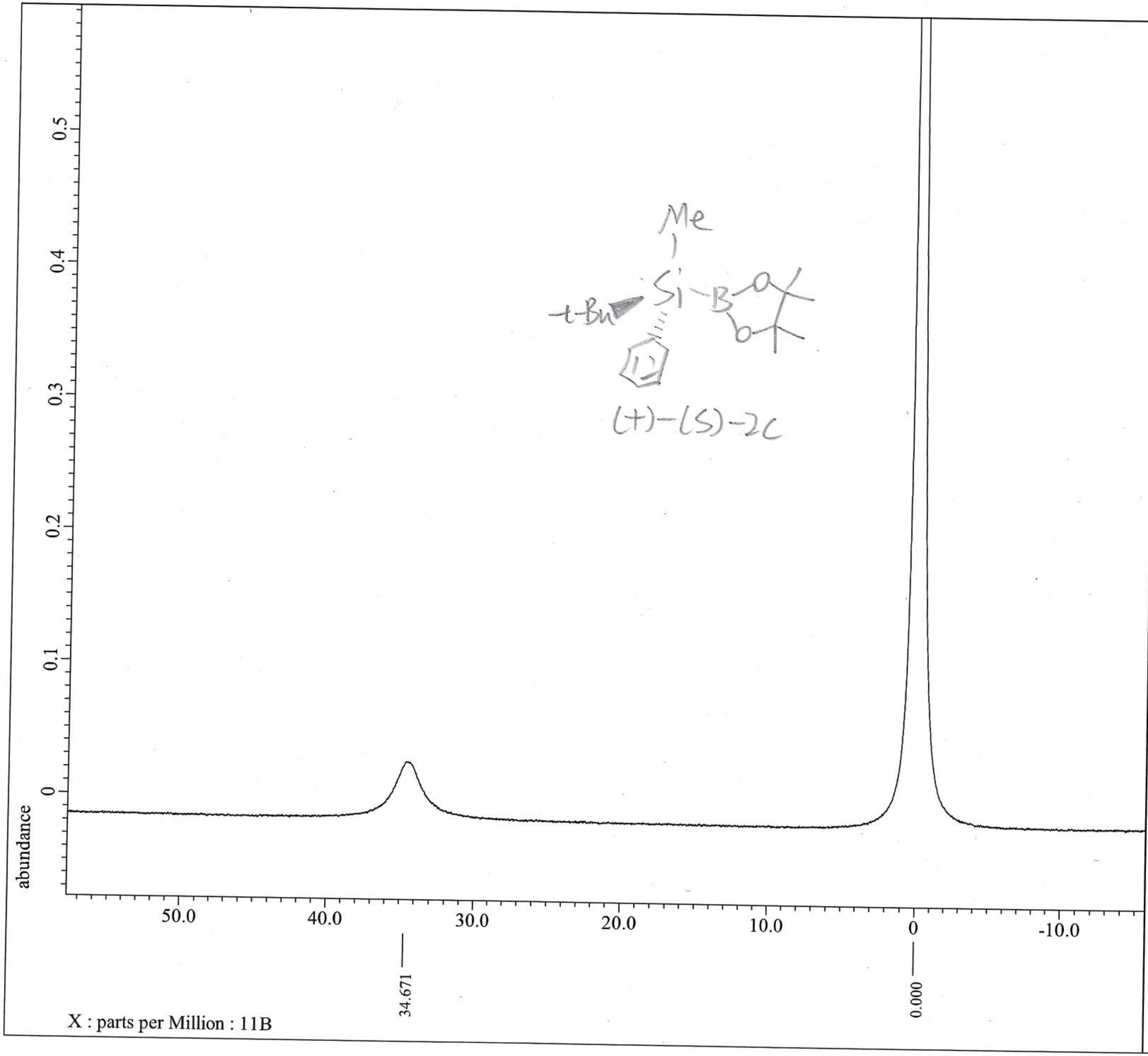


```
---- PROCESSING PARAMETERS ----
dc_balance( 0, FALSE )
sexp( 2.0[Hz], 0.0[s] )
trapezoid3( 0[%], 80[%], 100[%] )
zerofill( 1, TRUE )
fft( 1, TRUE, TRUE )
machinephase
ppm

数据来源: wxh-184-B-1.jdf

Filename            = wxh-184-B-3.jdf
Author              = element
Experiment          = single_pulse_dec
Sample_Id           = S#403829
Solvent             = CHLOROFORM-D
Actual_Start_Time   = 20-APR-2022 17:59:57
Revision_Time       =  6-MAY-2022 15:23:24

Comment             = single pulse decoupled ga
Data_Format         = 1D COMPLEX
Dim_Size            = 26214
X_Domain            = 11B
Dim_Title           = 11B
Dim_Units           = [ppm]
Dimensions          = X
Site                = ECS 400
Spectrometer        = JNM-ECS400

Field_Strength      = 9.20197068[T] (390[MHz])
X_Acq_Duration      = 0.83361792[s]
X_Domain            = 11B
X_Freq              = 125.70081325[MHz]
X_Offset            = 0[ppm]
X_Points            = 32768
X_Prescans          = 4
X_Resolution        = 1.19959034[Hz]
X_Sweep             = 39.3081761[kHz]
Irr_Domain          = 1H
Irr_Freq            = 391.78655441[MHz]
Irr_Offset          = 5[ppm]
Clipped             = FALSE
Scans               = 2000
Total_Scans         = 2000

Relaxation_Delay    = 2[s]
Recvr_Gain          = 46
Temp_Get            = 19.5[dC]
X_90_Width          = 10[us]
X_Acq_Time          = 0.83361792[s]
X_Angle             = 30[deg]
X_Atn               = 5.5[dB]
X_Pulse             = 3.33333333[us]
Irr_Atn_Dec         = 22.45[dB]
Irr_Atn_Noe         = 22.45[dB]
Irr_Noise           = WALTZ
Decoupling          = TRUE
Initial_Wait        = 1[s]
Noe                 = TRUE
Noe_Time            = 2[s]
Repetition_Time     = 2.83361792[s]
```

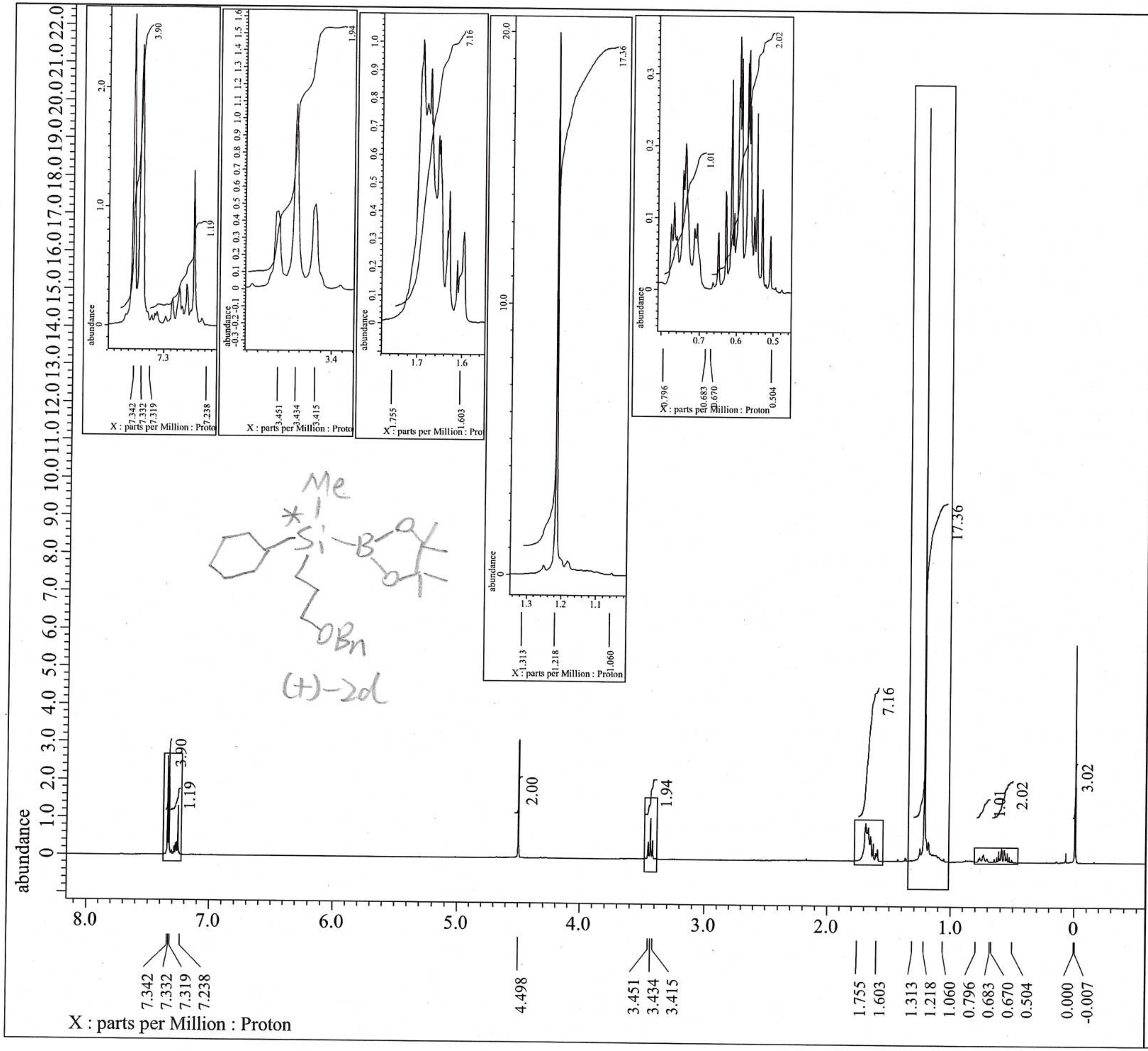


```
---- PROCESSING PARAMETERS ----
dc_balance( 0, FALSE )
sexp( 0.2[Hz], 0.0[s] )
trapezoid( 0[%], 0[%], 80[%], 100[%] )
zerofill( 1, TRUE )
fft( 1, TRUE, TRUE )
machinephase
ppm

数据来源: wxh-252-2_Proton-1-1.jdf

Filename             = wxh-252-2_Proton-1-2.jdf
Author               = element
Experiment           = proton.jxp
Sample_Id            = wxh-252-2
Solvent              = CHLOROFORM-D
Actual_Start_Time    = 19-APR-2022 10:53:10
Revision_Time        = 28-JUN-2023 11:47:40

Comment              = single_pulse
Data_Format          = 1D COMPLEX
Dim_Size             = 13107
X_Domain             = Proton
Dim_Title            = Proton
Dim_Units            = [ppm]
Dimensions           = X
Site                 = JNM-ECS400
Spectrometer         = DELTA2_NMR

Field_Strength       = 9.37221[T] (400[MHz])
X_Acq_Duration       = 2.1889024[s]
X_Domain             = 1H
X_Freq               = 399.03472754[MHz]
X_Offset             = 5.0[ppm]
X_Points             = 16384
X_Prescans           = 1
X_Resolution         = 0.45684997[Hz]
X_Sweep              = 7.48502994[kHz]
X_Sweep_Clipped      = 5.98802395[kHz]
Irr_Domain           = Proton
Irr_Freq             = 399.03472754[MHz]
Irr_Offset           = 5.0[ppm]
Tri_Domain           = Proton
Tri_Freq             = 399.03472754[MHz]
Tri_Offset           = 5.0[ppm]
Clipped              = FALSE
Scans                = 8
Total_Scans          = 8

Relaxation_Delay     = 5[s]
Recvr_Gain           = 30
Temp_Get             = 19.5[dC]
X_90_Width           = 6.6[us]
X_Acq_Time           = 2.1889024[s]
X_Angle              = 45[deg]
X_Atn                = 1[dB]
X_Pulse              = 3.3[us]
Irr_Mode             = Off
Tri_Mode             = Off
Dante_Presat         = FALSE
Initial_Wait         = 1[s]
Repetition_Time      = 7.1889024[s]
```

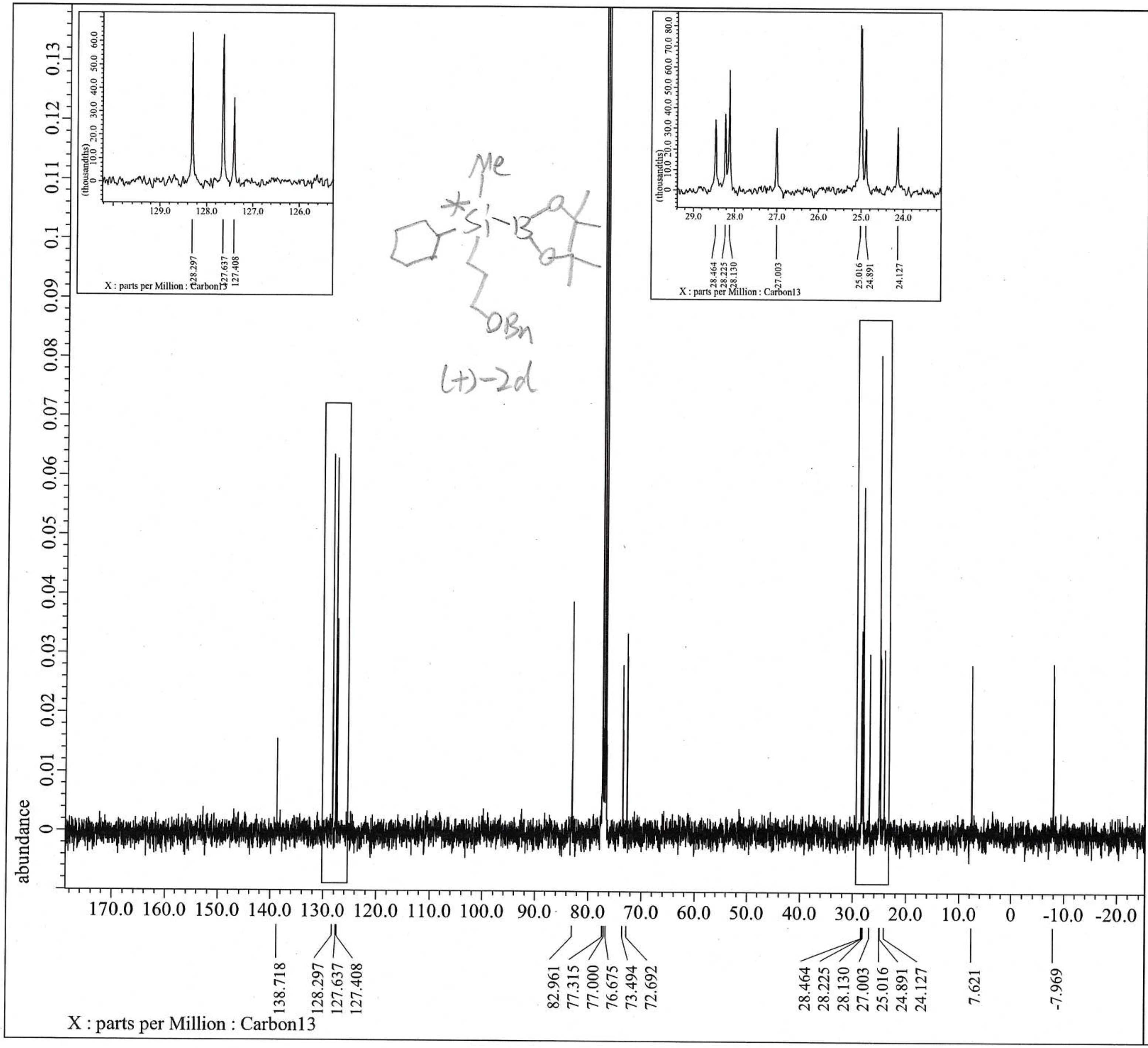

---- PROCESSING PARAMETERS ----
dc_balance(0, FALSE)
sexp(2.0[Hz], 0.0[s])
trapezoid(0[%], 0[%], 80[%], 100[%])
zerofill(1, TRUE)
fft(1, TRUE, TRUE)
machinephase
ppm

数据来源: wxh-252-2_Carbon-1-1.jdf

Filename	= wxh-252-2_Carbon-1-2.jdf
Author	= element
Experiment	= carbon.jxp
Sample_Id	= wxh-252-2
Solvent	= CHLOROFORM-D
Actual_Start_Time	= 19-APR-2022 11:01:39
Revision_Time	= 22-APR-2022 15:51:06
Comment	= single pulse decoupled ga
Data_Format	= 1D COMPLEX
Dim_Size	= 26214
X_Domain	= Carbon
Dim_Title	= Carbon13
Dim_Units	= [ppm]
Dimensions	= X
Site	= JNM-ECS400
Spectrometer	= DELTA2_NMR
Field_Strength	= 9.37221[T] (400[MHz])
X_Acq_Duration	= 1.04333312[s]
X_Domain	= 13C
X_Freq	= 100.33735165[MHz]
X_Offset	= 100.0[ppm]
X_Points	= 32768
X_Prescans	= 4
X_Resolution	= 0.95846665[Hz]
X_Sweep	= 31.40703518[kHz]
X_Sweep_Clipped	= 25.12562814[kHz]
Irr_Domain	= Proton
Irr_Freq	= 399.03472754[MHz]
Irr_Offset	= 5.0[ppm]
Clipped	= FALSE
Scans	= 256
Total_Scans	= 256
Relaxation_Delay	= 2[s]
Recvr_Gain	= 50
Temp_Get	= 19.9[dC]
X_90_Width	= 10.9[us]
X_Acq_Time	= 1.04333312[s]
X_Angle	= 30[deg]
X_Atn	= 5.4[dB]
X_Pulse	= 3.63333333[us]
Irr_Atn_Dec	= 25.823[dB]
Irr_Atn_Noe	= 25.823[dB]
Irr_Noise	= WALTZ
Irr_Pwidth	= 0.115[ms]
Decoupling	= TRUE
Initial_Wait	= 1[s]
Noe	= TRUE
Noe_Time	= 2[s]
Repetition_Time	= 3.04333312[s]

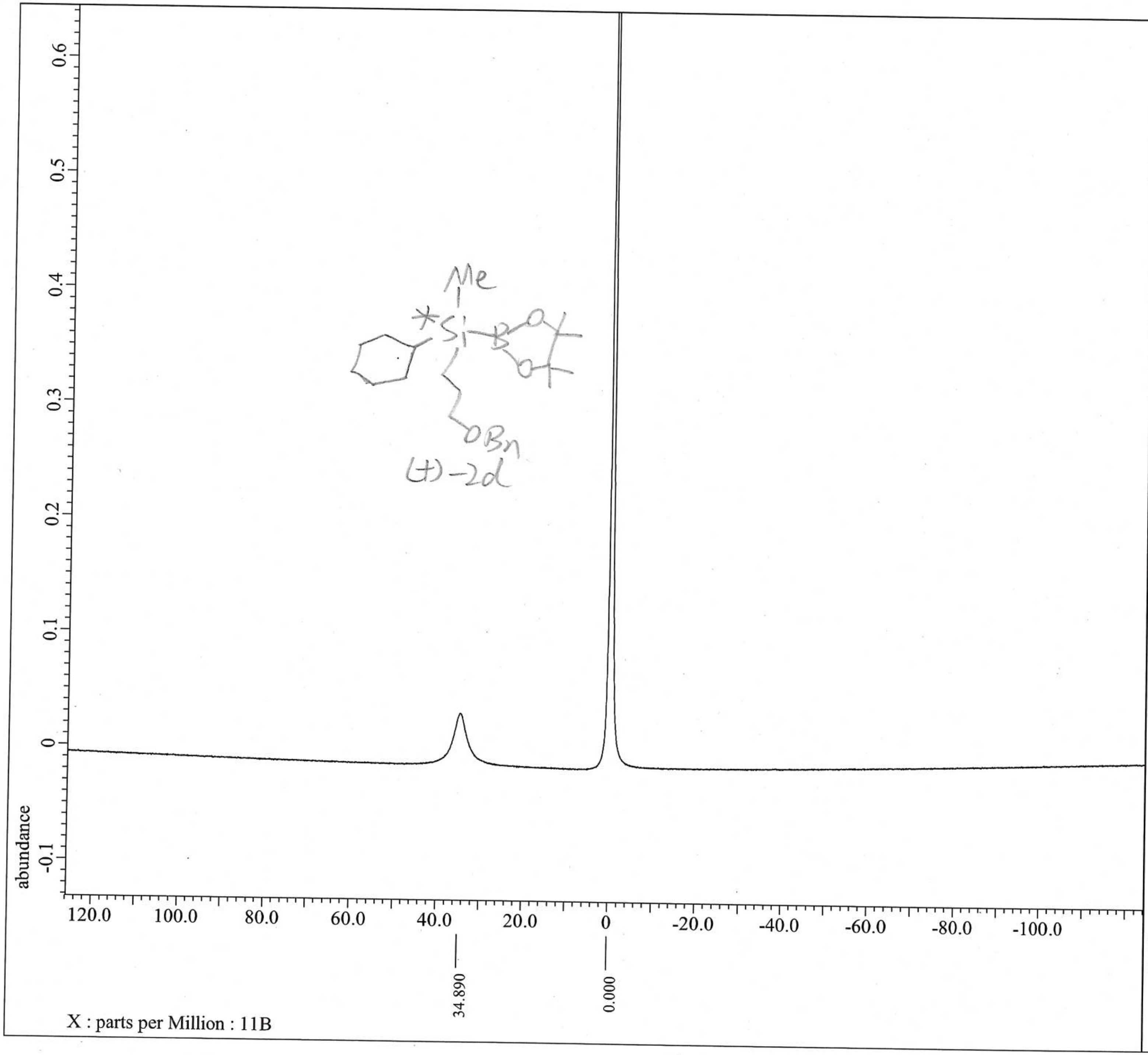


```
---- PROCESSING PARAMETERS ----
dc_balance( 0, FALSE )
sexp( 2.0[Hz], 0.0[s] )
trapezoid3( 0[%], 80[%], 100[%] )
zerofill( 1, TRUE )
fft( 1, TRUE, TRUE )
machinephase
ppm

数据来源: wxh-252-B-1.jdf
```

```
Filename            = wxh-252-B-2.jdf
Author              = element
Experiment          = single_pulse_dec
Sample_Id           = S#455972
Solvent             = CHLOROFORM-D
Actual_Start_Time   = 19-APR-2022 19:27:01
Revision_Time       = 22-APR-2022 17:11:30

Comment             = single pulse decoupled ga
Data_Format         = 1D COMPLEX
Dim_Size            = 26214
X_Domain            = 11B
Dim_Title           = 11B
Dim_Units           = [ppm]
Dimensions          = X
Site                = ECS 400
Spectrometer        = JNM-ECS400

Field_Strength      = 9.20197068[T] (390[MHz])
X_Acq_Duration      = 0.83361792[s]
X_Domain            = 11B
X_Freq              = 125.70081325[MHz]
X_Offset            = 0[ppm]
X_Points            = 32768
X_Prescans          = 4
X_Resolution        = 1.19959034[Hz]
X_Sweep             = 39.3081761[kHz]
Irr_Domain          = 1H
Irr_Freq            = 391.78655441[MHz]
Irr_Offset          = 5[ppm]
Clipped             = FALSE
Scans               = 2500
Total_Scans         = 2500

Relaxation_Delay    = 2[s]
Recvr_Gain          = 44
Temp_Get            = 18.4[dC]
X_90_Width          = 10[us]
X_Acq_Time          = 0.83361792[s]
X_Angle             = 30[deg]
X_Atn               = 5.5[dB]
X_Pulse             = 3.33333333[us]
Irr_Atn_Dec         = 22.45[dB]
Irr_Atn_Noe         = 22.45[dB]
Irr_Noise           = WALTZ
Decoupling          = TRUE
Initial_Wait        = 1[s]
Noe                 = TRUE
Noe_Time            = 2[s]
Repetition_Time     = 2.83361792[s]
```

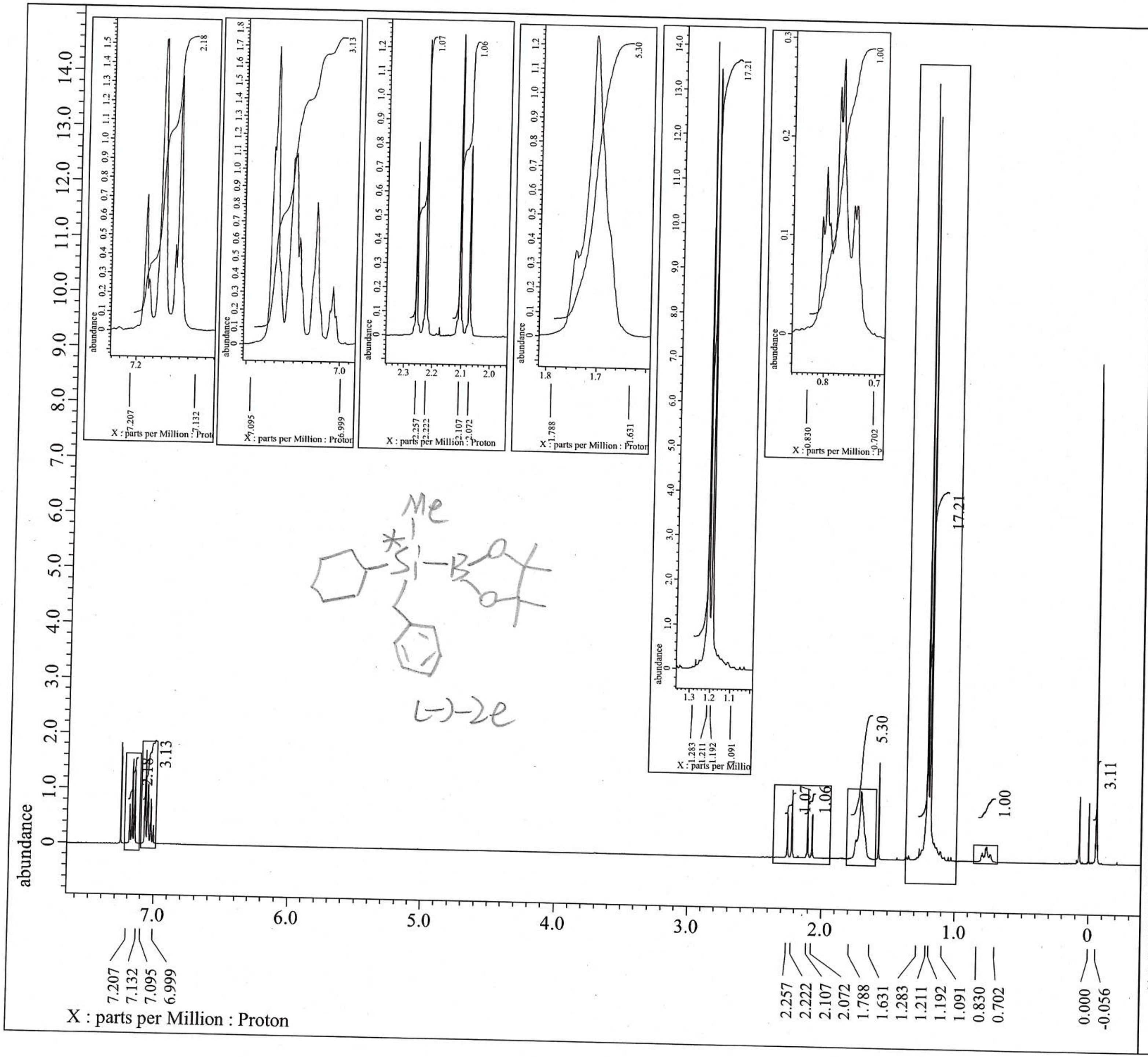

---- PROCESSING PARAMETERS ----
dc_balance(0, FALSE)
sexp(0.2[Hz], 0.0[s])
trapezoid(0[%], 0[%], 80[%], 100[%])
zerofill(1, TRUE)
fft(1, TRUE, TRUE)
machinephase
ppm

数据来源: wxh-142-2_Proton-1-1.jdf

Filename	= wxh-142-2_Proton-1-2.jdf
Author	= element
Experiment	= proton.jxp
Sample_Id	= wxh-142-2
Solvent	= CHLOROFORM-D
Actual_Start_Time	= 22-APR-2022 10:37:45
Revision_Time	= 28-JUN-2023 12:04:23
Comment	= single_pulse
Data_Format	= 1D COMPLEX
Dim_Size	= 13107
X_Domain	= Proton
Dim_Title	= Proton
Dim_Units	= [ppm]
Dimensions	= X
Site	= JNM-ECS400
Spectrometer	= DELTA2_NMR
Field_Strength	= 9.37221[T] (400[MHz])
X_Acq_Duration	= 2.1889024[s]
X_Domain	= 1H
X_Freq	= 399.03472754[MHz]
X_Offset	= 5.0[ppm]
X_Points	= 16384
X_Prescans	= 1
X_Resolution	= 0.45684997[Hz]
X_Sweep	= 7.48502994[kHz]
X_Sweep_Clipped	= 5.98802395[kHz]
Irr_Domain	= Proton
Irr_Freq	= 399.03472754[MHz]
Irr_Offset	= 5.0[ppm]
Tri_Domain	= Proton
Tri_Freq	= 399.03472754[MHz]
Tri_Offset	= 5.0[ppm]
Clipped	= FALSE
Scans	= 8
Total_Scans	= 8
Relaxation_Delay	= 5[s]
Recvr_Gain	= 34
Temp_Get	= 17.5[dC]
X_90_Width	= 6.6[us]
X_Acq_Time	= 2.1889024[s]
X_Angle	= 45[deg]
X_Atn	= 1[dB]
X_Pulse	= 3.3[us]
Irr_Mode	= Off
Tri_Mode	= Off
Dante_Presat	= FALSE
Initial_Wait	= 1[s]
Repetition_Time	= 7.1889024[s]

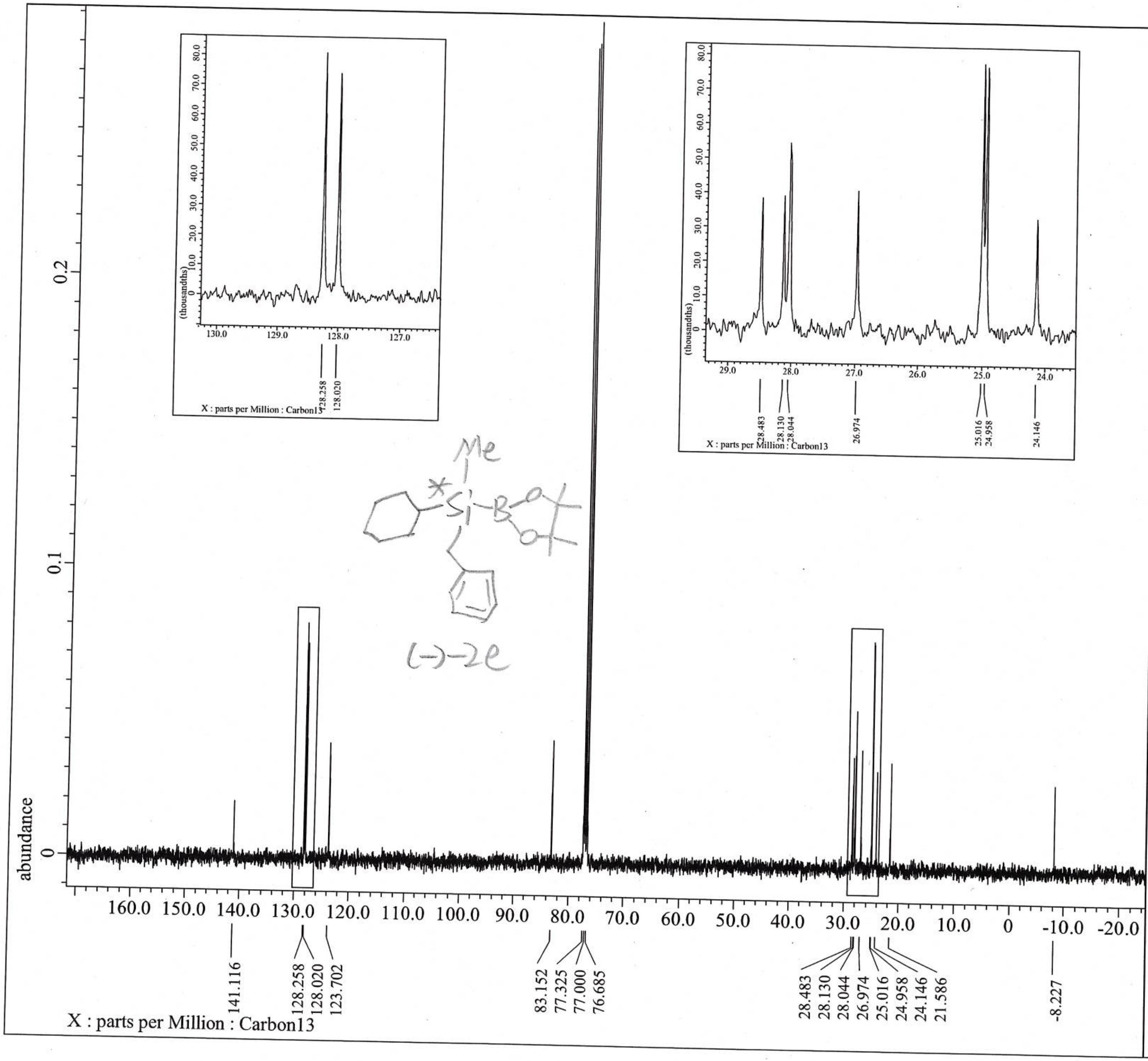

---- PROCESSING PARAMETERS ----
dc_balance(0, FALSE)
sexp(2.0[Hz], 0.0[s])
trapezoid(0[%], 0[%], 80[%], 100[%])
zerofill(1, TRUE)
fft(1, TRUE, TRUE)
machinephase
ppm

数据来源: wxh-142-2_Carbon-1-1.jdf

Filename	= wxh-142-2_Carbon-1-2.jdf
Author	= element
Experiment	= carbon.jxp
Sample_Id	= wxh-142-2
Solvent	= CHLOROFORM-D
Actual_Start_Time	= 22-APR-2022 10:46:02
Revision_Time	= 22-APR-2022 16:20:19
Comment	= single pulse decoupled ga
Data_Format	= 1D COMPLEX
Dim_Size	= 26214
X_Domain	= Carbon
Dim_Title	= Carbon13
Dim_Units	= [ppm]
Dimensions	= X
Site	= JNM-ECS400
Spectrometer	= DELTA2_NMR
Field_Strength	= 9.37221[T] (400[MHz])
X_Acq_Duration	= 1.04333312[s]
X_Domain	= 13C
X_Freq	= 100.33735165[MHz]
X_Offset	= 100.0[ppm]
X_Points	= 32768
X_Prescans	= 4
X_Resolution	= 0.95846665[Hz]
X_Sweep	= 31.40703518[kHz]
X_Sweep_Clipped	= 25.12562814[kHz]
Irr_Domain	= Proton
Irr_Freq	= 399.03472754[MHz]
Irr_Offset	= 5.0[ppm]
Clipped	= FALSE
Scans	= 256
Total_Scans	= 256
Relaxation_Delay	= 2[s]
Recvr_Gain	= 50
Temp_Get	= 17.5[dC]
X_90_Width	= 10.9[us]
X_Acq_Time	= 1.04333312[s]
X_Angle	= 30[deg]
X_Atn	= 5.4[dB]
X_Pulse	= 3.63333333[us]
Irr_Atn_Dec	= 25.823[dB]
Irr_Atn_Noe	= 25.823[dB]
Irr_Noise	= WALTZ
Irr_Pwidth	= 0.115[ms]
Decoupling	= TRUE
Initial_Wait	= 1[s]
Noe	= TRUE
Noe_Time	= 2[s]
Repetition_Time	= 3.04333312[s]

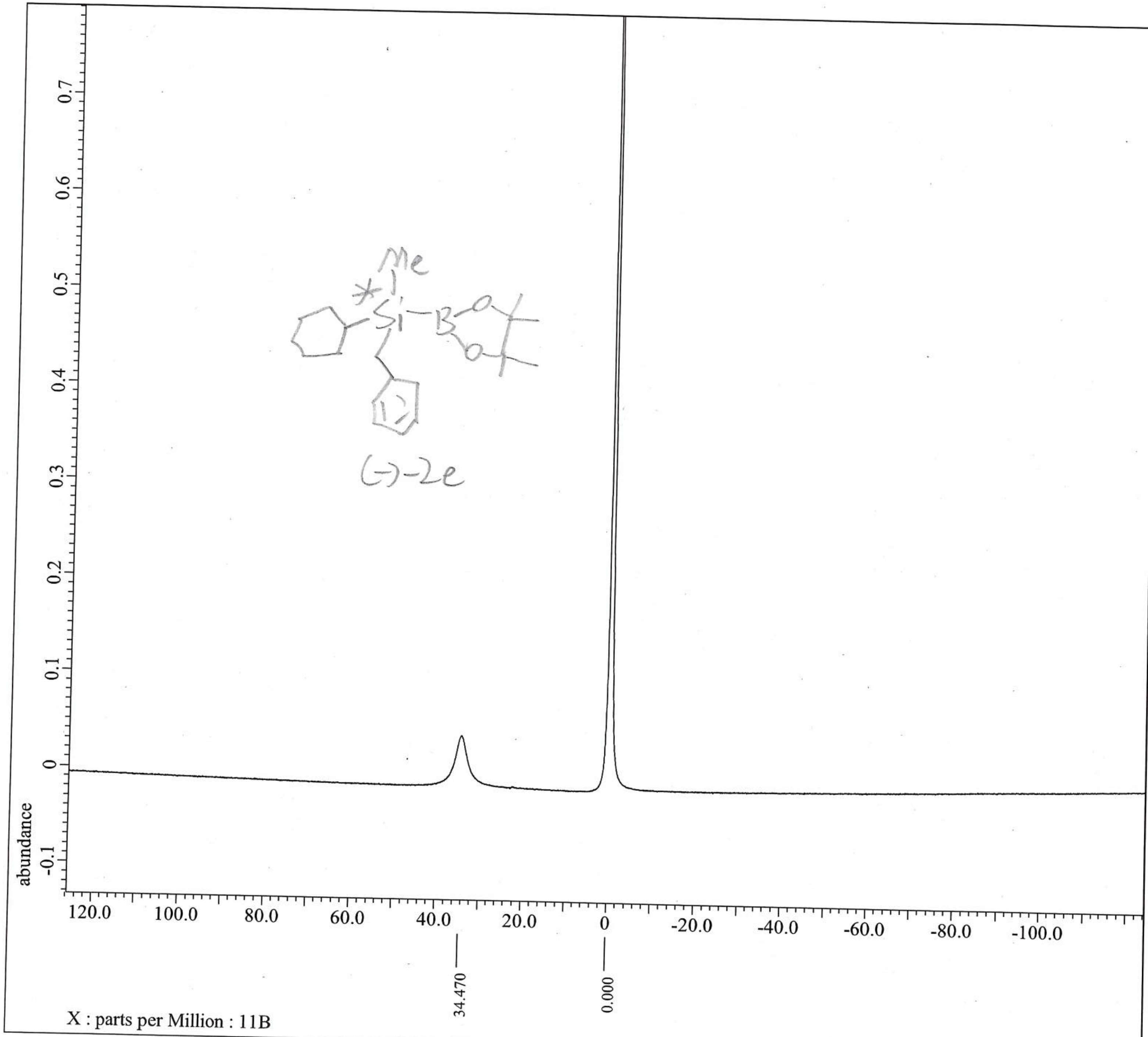


```
---- PROCESSING PARAMETERS ----
dc_balance( 0, FALSE )
sexp( 2.0[Hz], 0.0[s] )
trapezoid3( 0[%], 80[%], 100[%] )
zerofill( 1, TRUE )
fft( 1, TRUE, TRUE )
machinephase
ppm

数据来源: wxh-142-B-1.jdf
```

```
Filename            = wxh-142-B-2.jdf
Author              = element
Experiment          = single_pulse_dec
Sample_Id           = S#379298
Solvent             = CHLOROFORM-D
Actual_Start_Time   = 22-APR-2022 17:18:31
Revision_Time       = 22-APR-2022 16:52:14

Comment             = single pulse decoupled ga
Data_Format         = 1D COMPLEX
Dim_Size            = 26214
X_Domain            = 11B
Dim_Title           = 11B
Dim_Units           = [ppm]
Dimensions          = X
Site                = ECS 400
Spectrometer        = JNM-ECS400

Field_Strength      = 9.20197068[T] (390[MHz])
X_Acq_Duration      = 0.83361792[s]
X_Domain            = 11B
X_Freq              = 125.70081325[MHz]
X_Offset            = 0[ppm]
X_Points            = 32768
X_Prescans          = 4
X_Resolution        = 1.19959034[Hz]
X_Sweep             = 39.3081761[kHz]
Irr_Domain          = 1H
Irr_Freq            = 391.78655441[MHz]
Irr_Offset          = 5[ppm]
Clipped             = FALSE
Scans               = 2200
Total_Scans         = 2200

Relaxation_Delay    = 2[s]
Recvr_Gain          = 44
Temp_Get            = 18.4[dC]
X_90_Width          = 10[us]
X_Acq_Time          = 0.83361792[s]
X_Angle             = 30[deg]
X_Atn               = 5.5[dB]
X_Pulse             = 3.33333333[us]
Irr_Atn_Dec         = 22.45[dB]
Irr_Atn_Noe         = 22.45[dB]
Irr_Noise           = WALTZ
Decoupling          = TRUE
Initial_Wait        = 1[s]
Noe                 = TRUE
Noe_Time            = 2[s]
Repetition_Time     = 2.83361792[s]
```

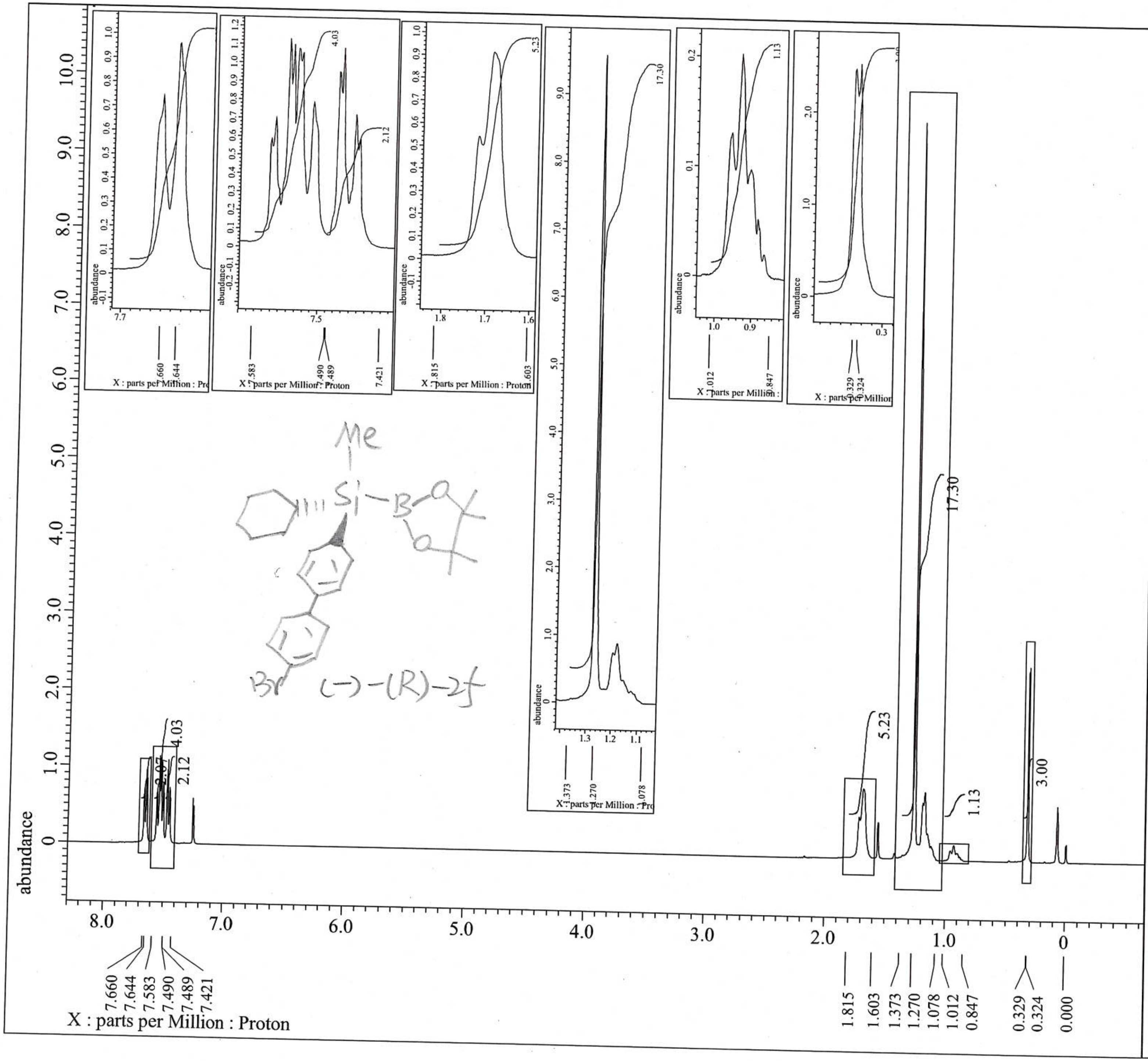

---- PROCESSING PARAMETERS ----
dc_balance(0, FALSE)
sexp(0.2[Hz], 0.0[s])
trapezoid(0[%], 0[%], 80[%], 100[%])
zerofill(1, TRUE)
fft(1, TRUE, TRUE)
machinephase
ppm

数据来源: wxh-137-2_Proton-1-1.jdf

Filename	= wxh-137-2_Proton-1-3.jdf
Author	= element
Experiment	= proton.jxp
Sample_Id	= wxh-137-1
Solvent	= CHLOROFORM-D
Actual_Start_Time	= 21-APR-2022 10:05:45
Revision_Time	= 28-JUN-2023 12:23:35
Comment	= single_pulse
Data_Format	= 1D COMPLEX
Dim_Size	= 13107
X_Domain	= Proton
Dim_Title	= Proton
Dim_Units	= [ppm]
Dimensions	= X
Site	= JNM-ECS400
Spectrometer	= DELTA2_NMR
Field_Strength	= 9.37221[T] (400[MHz])
X_Acq_Duration	= 2.1889024[s]
X_Domain	= 1H
X_Freq	= 399.03472754[MHz]
X_Offset	= 5.0[ppm]
X_Points	= 16384
X_Prescans	= 1
X_Resolution	= 0.45684997[Hz]
X_Sweep	= 7.48502994[kHz]
X_Sweep_Clipped	= 5.98802395[kHz]
Irr_Domain	= Proton
Irr_Freq	= 399.03472754[MHz]
Irr_Offset	= 5.0[ppm]
Tri_Domain	= Proton
Tri_Freq	= 399.03472754[MHz]
Tri_Offset	= 5.0[ppm]
Clipped	= FALSE
Scans	= 8
Total_Scans	= 8
Relaxation_Delay	= 5[s]
Recvr_Gain	= 36
Temp_Get	= 19.2[dC]
X_90_Width	= 6.6[us]
X_Acq_Time	= 2.1889024[s]
X_Angle	= 45[deg]
X_Atn	= 1[dB]
X_Pulse	= 3.3[us]
Irr_Mode	= Off
Tri_Mode	= Off
Dante_Presat	= FALSE
Initial_Wait	= 1[s]
Repetition_Time	= 7.1889024[s]

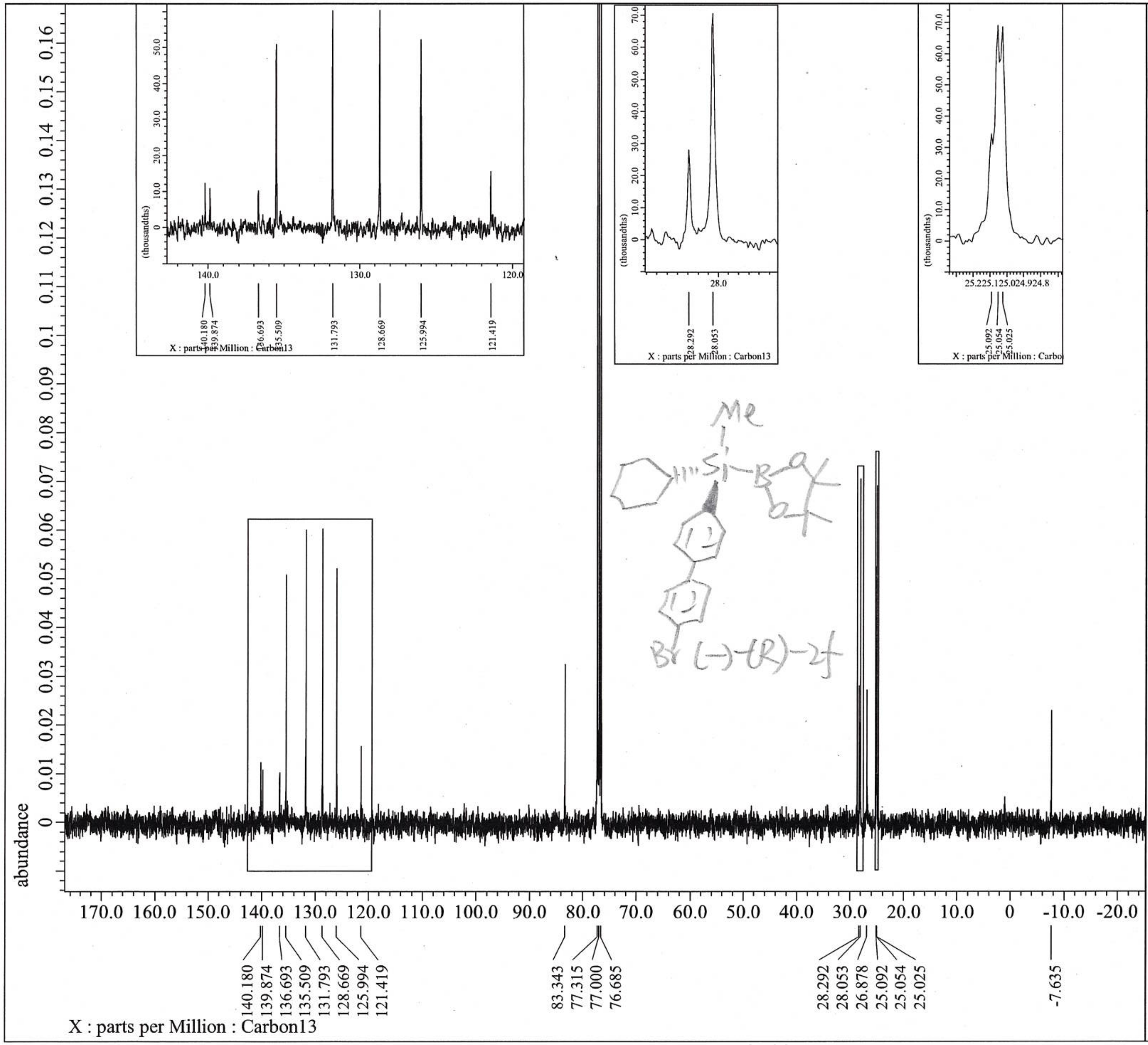

---- PROCESSING PARAMETERS ----
dc_balance(0, FALSE)
sexp(2.0[Hz], 0.0[s])
trapezoid(0[%], 0[%], 80[%], 100[%])
zerofill(1, TRUE)
fft(1, TRUE, TRUE)
machinephase
ppm

数据来源: wxh-137-2_Carbon-1-1.jdf

Filename	= wxh-137-2_Carbon-1-2.jdf
Author	= element
Experiment	= carbon.jxp
Sample_Id	= wxh-137-1
Solvent	= CHLOROFORM-D
Actual_Start_Time	= 21-APR-2022 10:14:01
Revision_Time	= 26-APR-2022 16:29:27
Comment	= single pulse decoupled ga
Data_Format	= 1D COMPLEX
Dim_Size	= 26214
X_Domain	= Carbon
Dim_Title	= Carbon13
Dim_Units	= [ppm]
Dimensions	= X
Site	= JNM-ECS400
Spectrometer	= DELTA2_NMR
Field_Strength	= 9.37221[T] (400[MHz])
X_Acq_Duration	= 1.04333312[s]
X_Domain	= 13C
X_Freq	= 100.33735165[MHz]
X_Offset	= 100.0[ppm]
X_Points	= 32768
X_Prescans	= 4
X_Resolution	= 0.95846665[Hz]
X_Sweep	= 31.40703518[kHz]
X_Sweep_Clipped	= 25.12562814[kHz]
Irr_Domain	= Proton
Irr_Freq	= 399.03472754[MHz]
Irr_Offset	= 5.0[ppm]
Clipped	= FALSE
Scans	= 256
Total_Scans	= 256
Relaxation_Delay	= 2[s]
Recvr_Gain	= 50
Temp_Get	= 18.4[dC]
X_90_Width	= 10.9[us]
X_Acq_Time	= 1.04333312[s]
X_Angle	= 30[deg]
X_Atn	= 5.4[dB]
X_Pulse	= 3.63333333[us]
Irr_Atn_Dec	= 25.823[dB]
Irr_Atn_Noe	= 25.823[dB]
Irr_Noise	= WALTZ
Irr_Pwidth	= 0.115[ms]
Decoupling	= TRUE
Initial_Wait	= 1[s]
Noe	= TRUE
Noe_Time	= 2[s]
Repetition_Time	= 3.04333312[s]

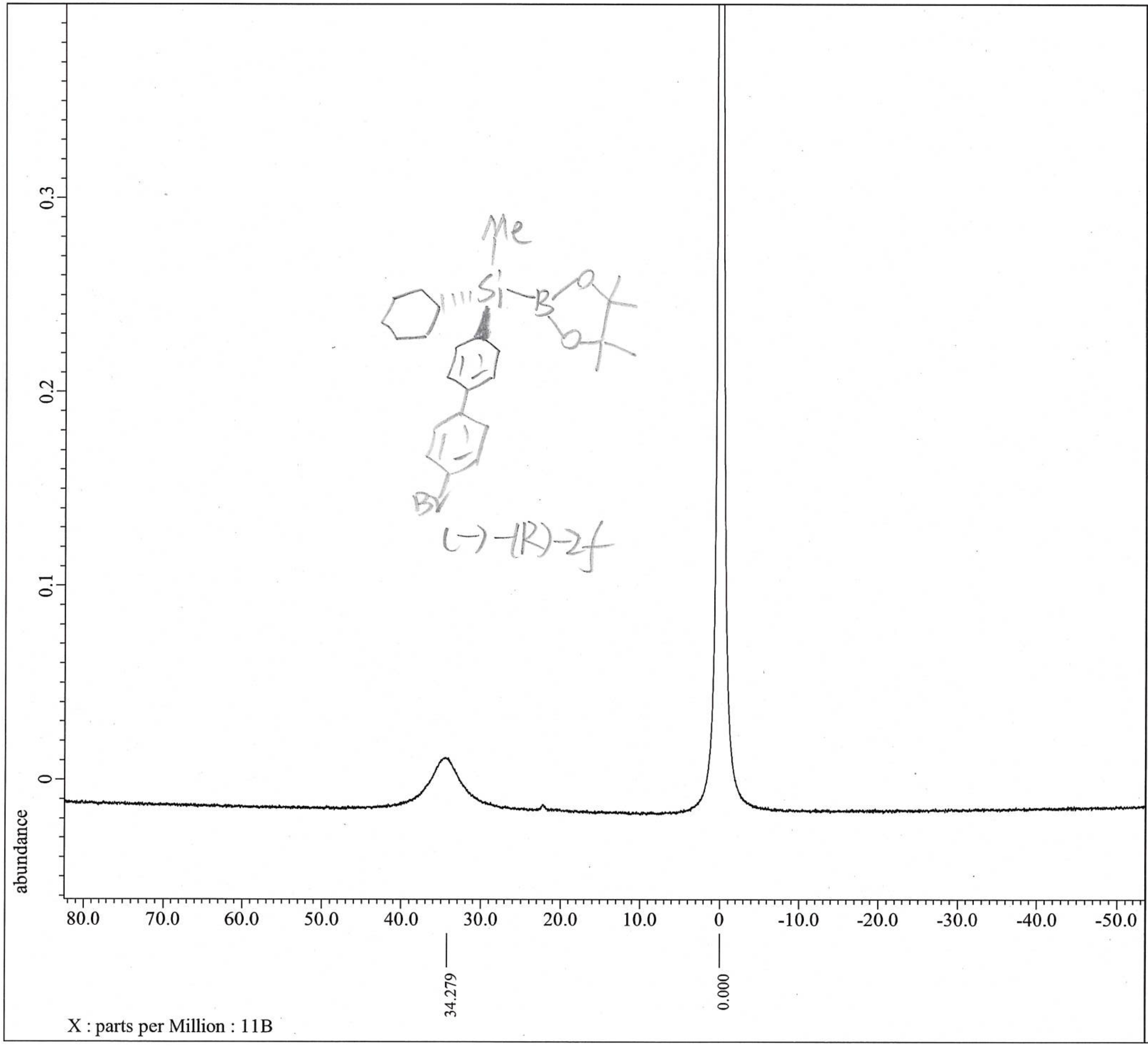


```
---- PROCESSING PARAMETERS ----
dc_balance( 0, FALSE )
sexp( 2.0[Hz], 0.0[s] )
trapezoid3( 0[%], 80[%], 100[%] )
zerofill( 1, TRUE )
fft( 1, TRUE, TRUE )
machinephase
ppm

数据来源: wxh-137-B-1.jdf
```

```
Filename            = wxh-137-B-2.jdf
Author              = element
Experiment          = single_pulse_dec
Sample_Id           = S#370476
Solvent             = CHLOROFORM-D
Actual_Start_Time   = 21-APR-2022 17:03:52
Revision_Time       = 26-APR-2022 16:15:07

Comment             = single pulse decoupled ga
Data_Format         = 1D COMPLEX
Dim_Size            = 26214
X_Domain            = 11B
Dim_Title           = 11B
Dim_Units           = [ppm]
Dimensions          = X
Site                = ECS 400
Spectrometer        = JNM-ECS400

Field_Strength      = 9.20197068[T] (390[MHz])
X_Acq_Duration      = 0.83361792[s]
X_Domain            = 11B
X_Freq              = 125.70081325[MHz]
X_Offset            = 0[ppm]
X_Points            = 32768
X_Prescans          = 4
X_Resolution        = 1.19959034[Hz]
X_Sweep             = 39.3081761[kHz]
Irr_Domain          = 1H
Irr_Freq            = 391.78655441[MHz]
Irr_Offset          = 5[ppm]
Clipped             = FALSE
Scans               = 2200
Total_Scans         = 2200

Relaxation_Delay    = 2[s]
Recvr_Gain          = 46
Temp_Get            = 18.8[dC]
X_90_Width          = 10[us]
X_Acq_Time          = 0.83361792[s]
X_Angle             = 30[deg]
X_Atn               = 5.5[dB]
X_Pulse             = 3.33333333[us]
Irr_Atn_Dec         = 22.45[dB]
Irr_Atn_Noe         = 22.45[dB]
Irr_Noise           = WALTZ
Decoupling          = TRUE
Initial_Wait        = 1[s]
Noe                 = TRUE
Noe_Time            = 2[s]
Repetition_Time     = 2.83361792[s]
```

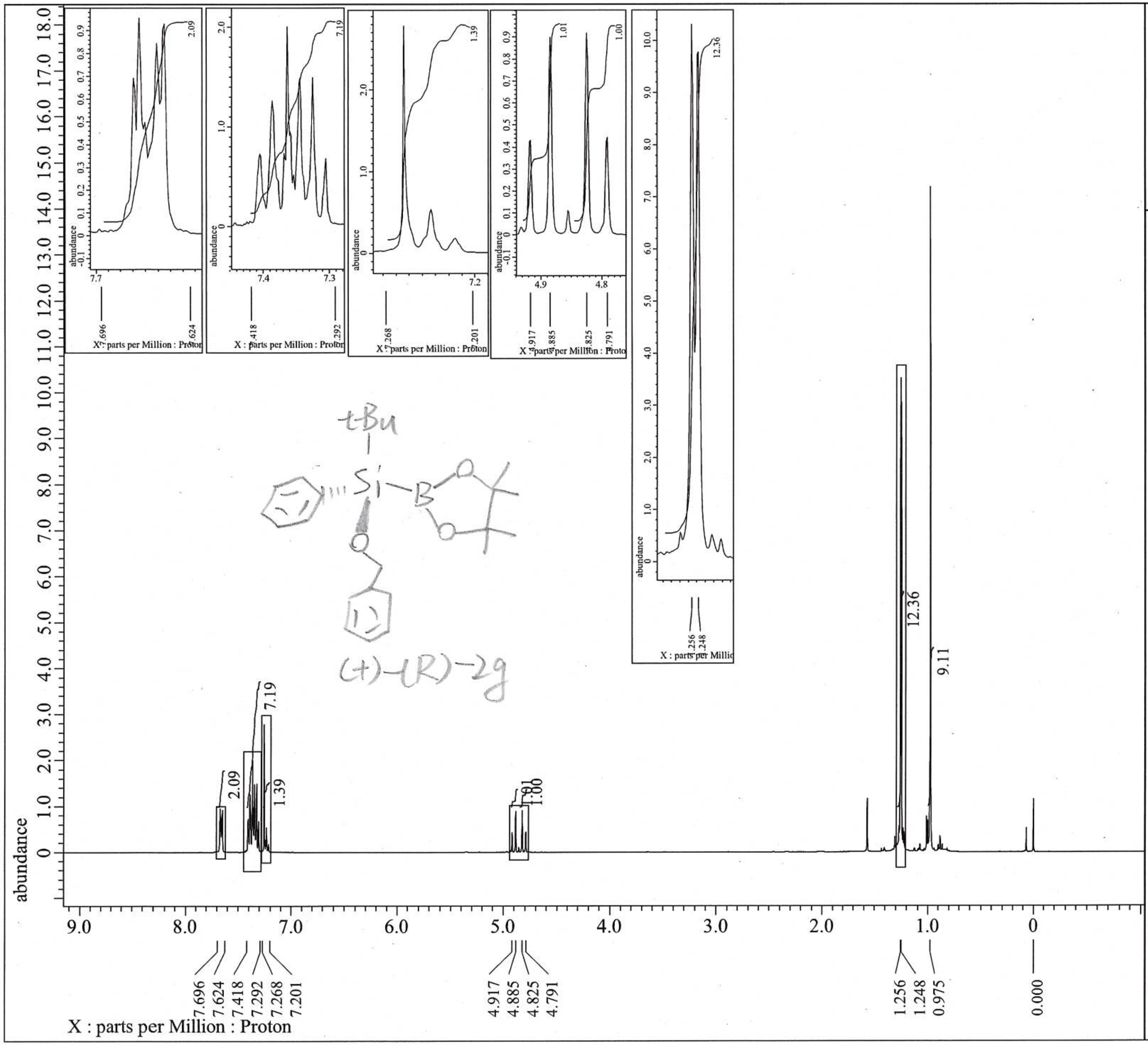

---- PROCESSING PARAMETERS ----
dc_balance(0, FALSE)
sexp(0.2[Hz], 0.0[s])
trapezoid(0[%], 0[%], 80[%], 100[%])
zerofill(1, TRUE)
fft(1, TRUE, TRUE)
machinephase
ppm

数据来源: wxh-332-2_Proton-1-1.jdf

Filename	= wxh-332-2_Proton-1-2.jdf
Author	= element
Experiment	= proton.jxp
Sample_Id	= wxh-332-2
Solvent	= CHLOROFORM-D
Actual_Start_Time	= 1-NOV-2022 16:50:42
Revision_Time	= 28-JUN-2023 13:31:51
Comment	= single_pulse
Data_Format	= 1D COMPLEX
Dim_Size	= 13107
X_Domain	= Proton
Dim_Title	= Proton
Dim_Units	= [ppm]
Dimensions	= X
Site	= JNM-ECS400
Spectrometer	= DELTA2_NMR
Field_Strength	= 9.37221[T] (400[MHz])
X_Acq_Duration	= 2.1889024[s]
X_Domain	= 1H
X_Freq	= 399.03472754[MHz]
X_Offset	= 5.0[ppm]
X_Points	= 16384
X_Prescans	= 1
X_Resolution	= 0.45684997[Hz]
X_Sweep	= 7.48502994[kHz]
X_Sweep_Clipped	= 5.98802395[kHz]
Irr_Domain	= Proton
Irr_Freq	= 399.03472754[MHz]
Irr_Offset	= 5.0[ppm]
Tri_Domain	= Proton
Tri_Freq	= 399.03472754[MHz]
Tri_Offset	= 5.0[ppm]
Clipped	= FALSE
Scans	= 8
Total_Scans	= 8
Relaxation_Delay	= 5[s]
Recvr_Gain	= 34
Temp_Get	= 17.5[dC]
X_90_Width	= 6.6[us]
X_Acq_Time	= 2.1889024[s]
X_Angle	= 45[deg]
X_Atn	= 1[dB]
X_Pulse	= 3.3[us]
Irr_Mode	= Off
Tri_Mode	= Off
Dante_Presat	= FALSE
Initial_Wait	= 1[s]
Repetition_Time	= 7.1889024[s]

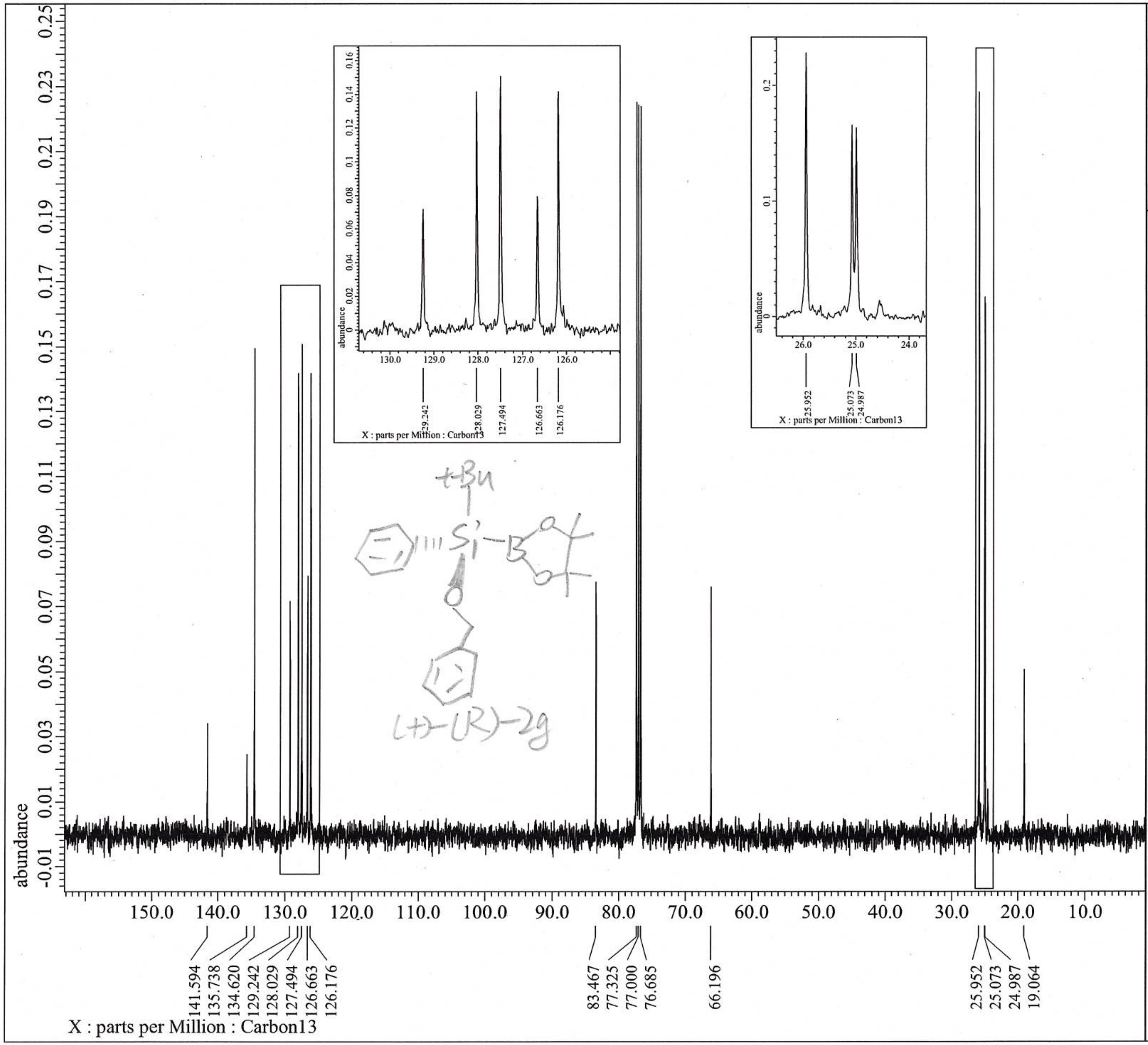

---- PROCESSING PARAMETERS ----
dc_balance(0, FALSE)
sexp(2.0[Hz], 0.0[s])
trapezoid(0[%], 0[%], 80[%], 100[%])
zerofill(1, TRUE)
fft(1, TRUE, TRUE)
machinephase
ppm

数据来源: wxh-332-2_Carbon-1-1.jdf

Filename = wxh-332-2_Carbon-1-2.jdf
Author = element
Experiment = carbon.jxp
Sample_Id = wxh-332-2
Solvent = CHLOROFORM-D
Actual_Start_Time = 29-OCT-2022 12:30:01
Revision_Time = 20-JAN-2023 16:38:19

Comment = single pulse decoupled ga
Data_Format = 1D COMPLEX
Dim_Size = 26214
X_Domain = Carbon
Dim_Title = Carbon13
Dim_Units = [ppm]
Dimensions = X
Site = JNM-ECS400
Spectrometer = DELTA2_NMR

Field_Strength = 9.37221[T] (400[MHz])
X_Acq_Duration = 1.04333312[s]
X_Domain = 13C
X_Freq = 100.33735165[MHz]
X_Offset = 100.0[ppm]
X_Points = 32768
X_Prescans = 4
X_Resolution = 0.95846665[Hz]
X_Sweep = 31.40703518[kHz]
X_Sweep_Clipped = 25.12562814[kHz]
Irr_Domain = Proton
Irr_Freq = 399.03472754[MHz]
Irr_Offset = 5.0[ppm]
Clipped = FALSE
Scans = 128
Total_Scans = 128

Relaxation_Delay = 2[s]
Recvr_Gain = 50
Temp_Get = 18.6[dC]
X_90_Width = 10.9[us]
X_Acq_Time = 1.04333312[s]
X_Angle = 30[deg]
X_Atn = 5.4[dB]
X_Pulse = 3.63333333[us]
Irr_Atn_Dec = 25.823[dB]
Irr_Atn_Noe = 25.823[dB]
Irr_Noise = WALTZ
Irr_Pwidth = 0.115[ms]
Decoupling = TRUE
Initial_Wait = 1[s]
Noe = TRUE
Noe_Time = 2[s]
Repetition_Time = 3.04333312[s]

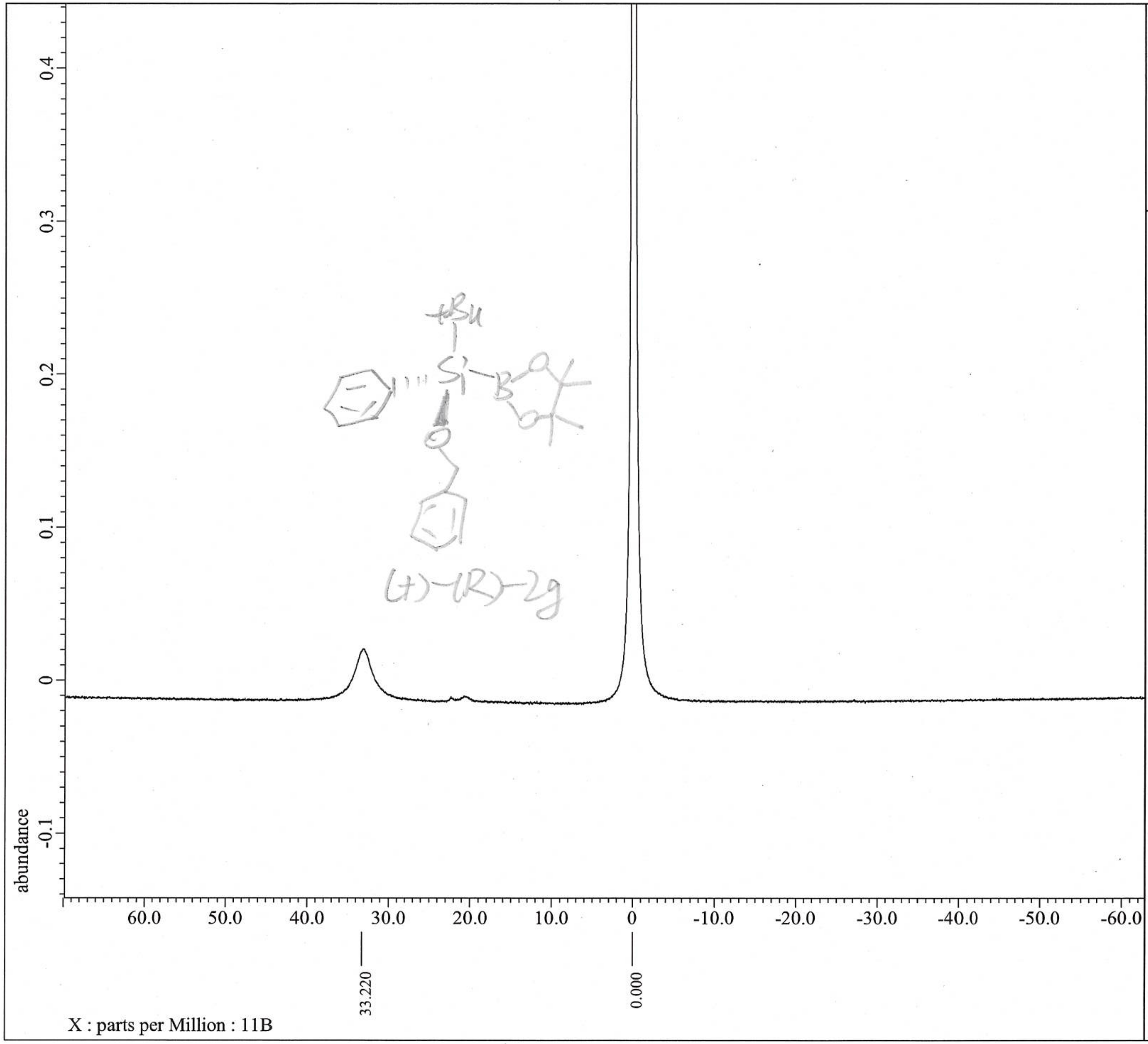


```
---- PROCESSING PARAMETERS ----
dc_balance( 0, FALSE )
sexp( 2.0[Hz], 0.0[s] )
trapezoid3( 0[%], 80[%], 100[%] )
zerofill( 1, TRUE )
fft( 1, TRUE, TRUE )
machinephase
ppm

数据来源: wxh-332-2-B-1.jdf
```

```
Filename            = wxh-332-2-B-2.jdf
Author              = element
Experiment          = single_pulse_dec
Sample_Id           = S#537040
Solvent             = CHLOROFORM-D
Actual_Start_Time   = 29-OCT-2022 21:38:55
Revision_Time       = 20-JAN-2023 17:03:22

Comment             = single pulse decoupled ga
Data_Format         = 1D COMPLEX
Dim_Size            = 26214
X_Domain            = 11B
Dim_Title           = 11B
Dim_Units           = [ppm]
Dimensions          = X
Site                = ECS 400
Spectrometer        = JNM-ECS400

Field_Strength      = 9.20197068[T] (390[MHz])
X_Acq_Duration      = 0.83361792[s]
X_Domain            = 11B
X_Freq              = 125.70081325[MHz]
X_Offset            = 0[ppm]
X_Points            = 32768
X_Prescans          = 4
X_Resolution        = 1.19959034[Hz]
X_Sweep             = 39.3081761[kHz]
Irr_Domain          = 1H
Irr_Freq            = 391.78655441[MHz]
Irr_Offset          = 5[ppm]
Clipped             = FALSE
Scans               = 1800
Total_Scans         = 1800

Relaxation_Delay    = 2[s]
Recvr_Gain          = 44
Temp_Get            = 17.9[dC]
X_90_Width          = 10[us]
X_Acq_Time          = 0.83361792[s]
X_Angle             = 30[deg]
X_Atn               = 5.5[dB]
X_Pulse             = 3.33333333[us]
Irr_Atn_Dec         = 22.45[dB]
Irr_Atn_Noe         = 22.45[dB]
Irr_Noise           = WALTZ
Decoupling          = TRUE
Initial_Wait        = 1[s]
Noe                 = TRUE
Noe_Time            = 2[s]
Repetition_Time     = 2.83361792[s]
```

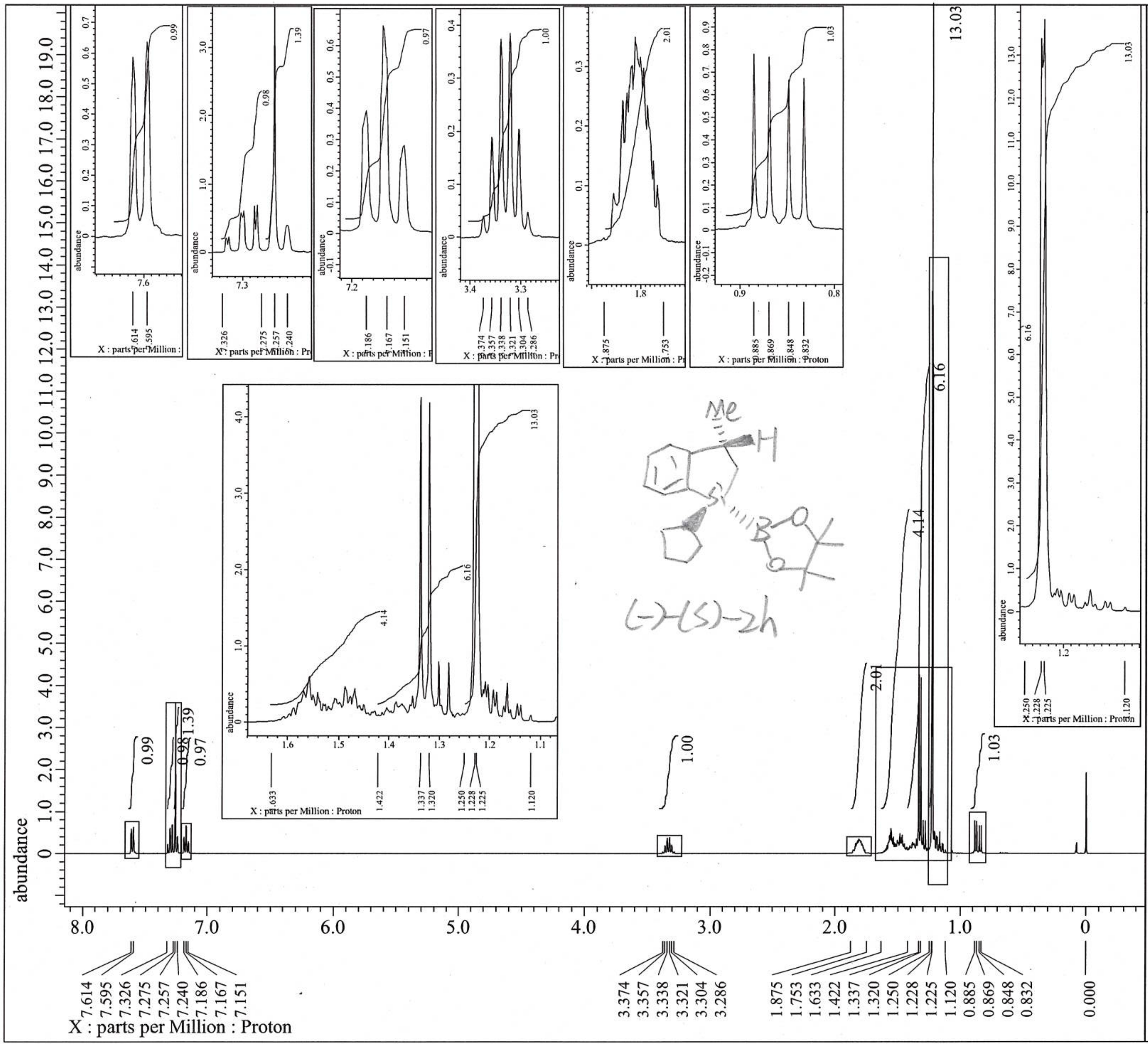

---- PROCESSING PARAMETERS ----
dc_balance(0, FALSE)
sexp(0.2[Hz], 0.0[s])
trapezoid(0[%], 0[%], 80[%], 100[%])
zerofill(1, TRUE)
fft(1, TRUE, TRUE)
machinephase
ppm

数据来源: wxh-407_Proton-1-1.jdf

Filename	= wxh-407_Proton-1-2.jdf
Author	= element
Experiment	= proton.jxp
Sample_Id	= wxh-407
Solvent	= CHLOROFORM-D
Actual_Start_Time	= 9-FEB-2023 10:39:34
Revision_Time	= 28-JUN-2023 14:05:15
Comment	= single_pulse
Data_Format	= 1D COMPLEX
Dim_Size	= 13107
X_Domain	= Proton
Dim_Title	= Proton
Dim_Units	= [ppm]
Dimensions	= X
Site	= JNM-ECS400
Spectrometer	= DELTA2_NMR
Field_Strength	= 9.37221[T] (400[MHz])
X_Acq_Duration	= 2.1889024[s]
X_Domain	= 1H
X_Freq	= 399.03472754[MHz]
X_Offset	= 5.0[ppm]
X_Points	= 16384
X_Prescans	= 1
X_Resolution	= 0.45684997[Hz]
X_Sweep	= 7.48502994[kHz]
X_Sweep_Clipped	= 5.98802395[kHz]
Irr_Domain	= Proton
Irr_Freq	= 399.03472754[MHz]
Irr_Offset	= 5.0[ppm]
Tri_Domain	= Proton
Tri_Freq	= 399.03472754[MHz]
Tri_Offset	= 5.0[ppm]
Clipped	= FALSE
Scans	= 8
Total_Scans	= 8
Relaxation_Delay	= 5[s]
Recvr_Gain	= 34
Temp_Get	= 18.2[dC]
X_90_Width	= 6.6[us]
X_Acq_Time	= 2.1889024[s]
X_Angle	= 45[deg]
X_Atn	= 1[dB]
X_Pulse	= 3.3[us]
Irr_Mode	= Off
Tri_Mode	= Off
Dante_Presat	= FALSE
Initial_Wait	= 1[s]
Repetition_Time	= 7.1889024[s]

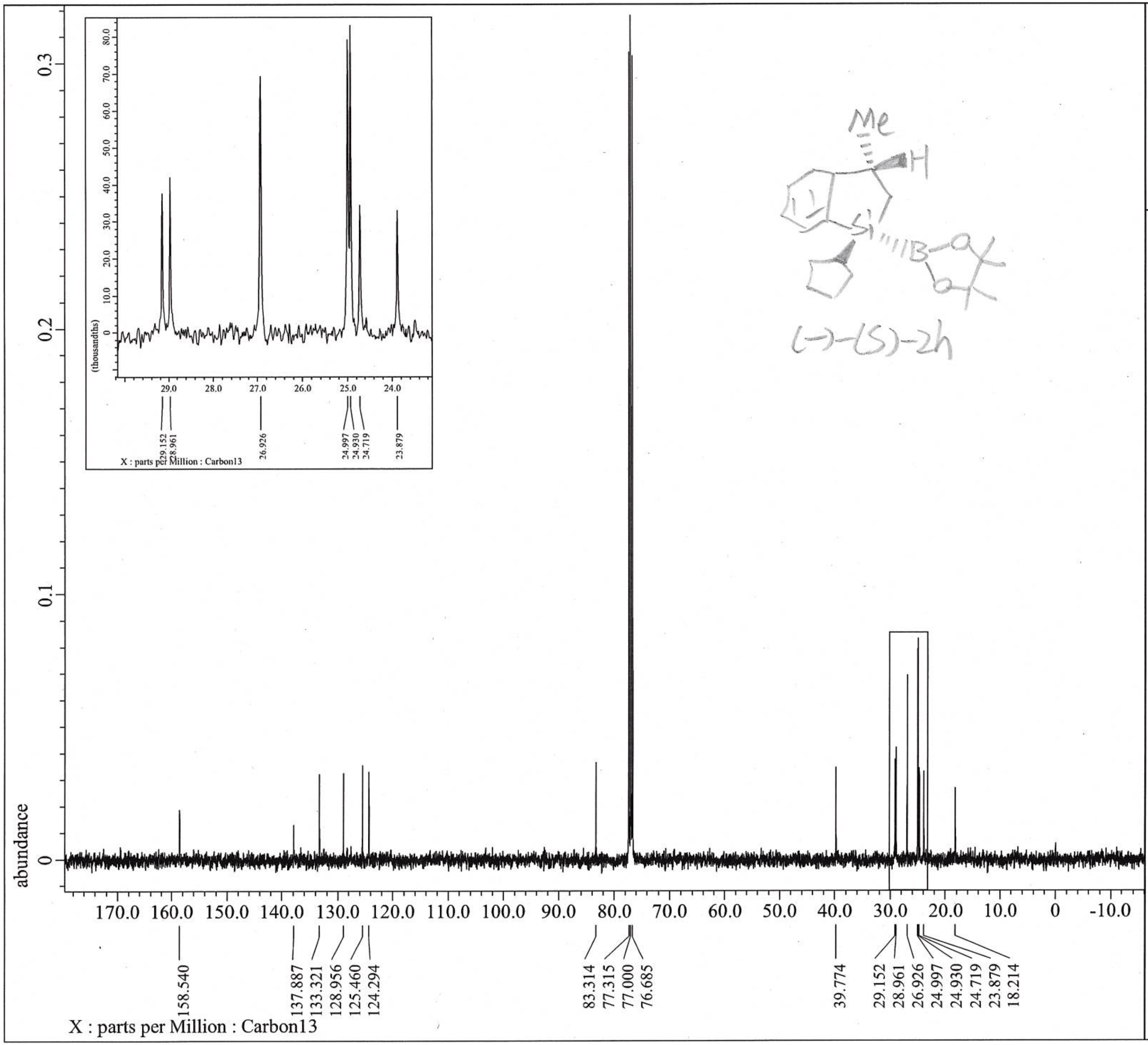

(−)-(S)-2h

```
---- PROCESSING PARAMETERS ----
dc_balance( 0, FALSE )
sexp( 2.0[Hz], 0.0[s] )
trapezoid( 0[%], 0[%], 80[%], 100[%] )
zerofill( 1, TRUE )
fft( 1, TRUE, TRUE )
machinephase
ppm

数据来源: wxh-407_Carbon-1-1.jdf
```

```
Filename            = wxh-407_Carbon-1-2.jdf
Author              = element
Experiment          = carbon.jxp
Sample_Id           = wxh-407
Solvent             = CHLOROFORM-D
Actual_Start_Time   =  9-FEB-2023 10:48:02
Revision_Time       = 13-FEB-2023 14:00:10

Comment             = single pulse decoupled ga
Data_Format         = 1D COMPLEX
Dim_Size            = 26214
X_Domain            = Carbon
Dim_Title           = Carbon13
Dim_Units           = [ppm]
Dimensions          = X
Site                = JNM-ECS400
Spectrometer        = DELTA2_NMR

Field_Strength      = 9.37221[T] (400[MHz])
X_Acq_Duration      = 1.04333312[s]
X_Domain            = 13C
X_Freq              = 100.33735165[MHz]
X_Offset            = 100.0[ppm]
X_Points            = 32768
X_Prescans          = 4
X_Resolution        = 0.95846665[Hz]
X_Sweep             = 31.40703518[kHz]
X_Sweep_Clipped     = 25.12562814[kHz]
Irr_Domain          = Proton
Irr_Freq            = 399.03472754[MHz]
Irr_Offset          = 5.0[ppm]
Clipped             = FALSE
Scans               = 256
Total_Scans         = 256

Relaxation_Delay    = 2[s]
Recvr_Gain          = 50
Temp_Get            = 18.1[dC]
X_90_Width          = 10.9[us]
X_Acq_Time          = 1.04333312[s]
X_Angle             = 30[deg]
X_Atn               = 5.4[dB]
X_Pulse             = 3.63333333[us]
Irr_Atn_Dec         = 25.823[dB]
Irr_Atn_Noe         = 25.823[dB]
Irr_Noise           = WALTZ
Irr_Pwidth          = 0.115[ms]
Decoupling          = TRUE
Initial_Wait        = 1[s]
Noe                 = TRUE
Noe_Time            = 2[s]
Repetition_Time     = 3.04333312[s]
```

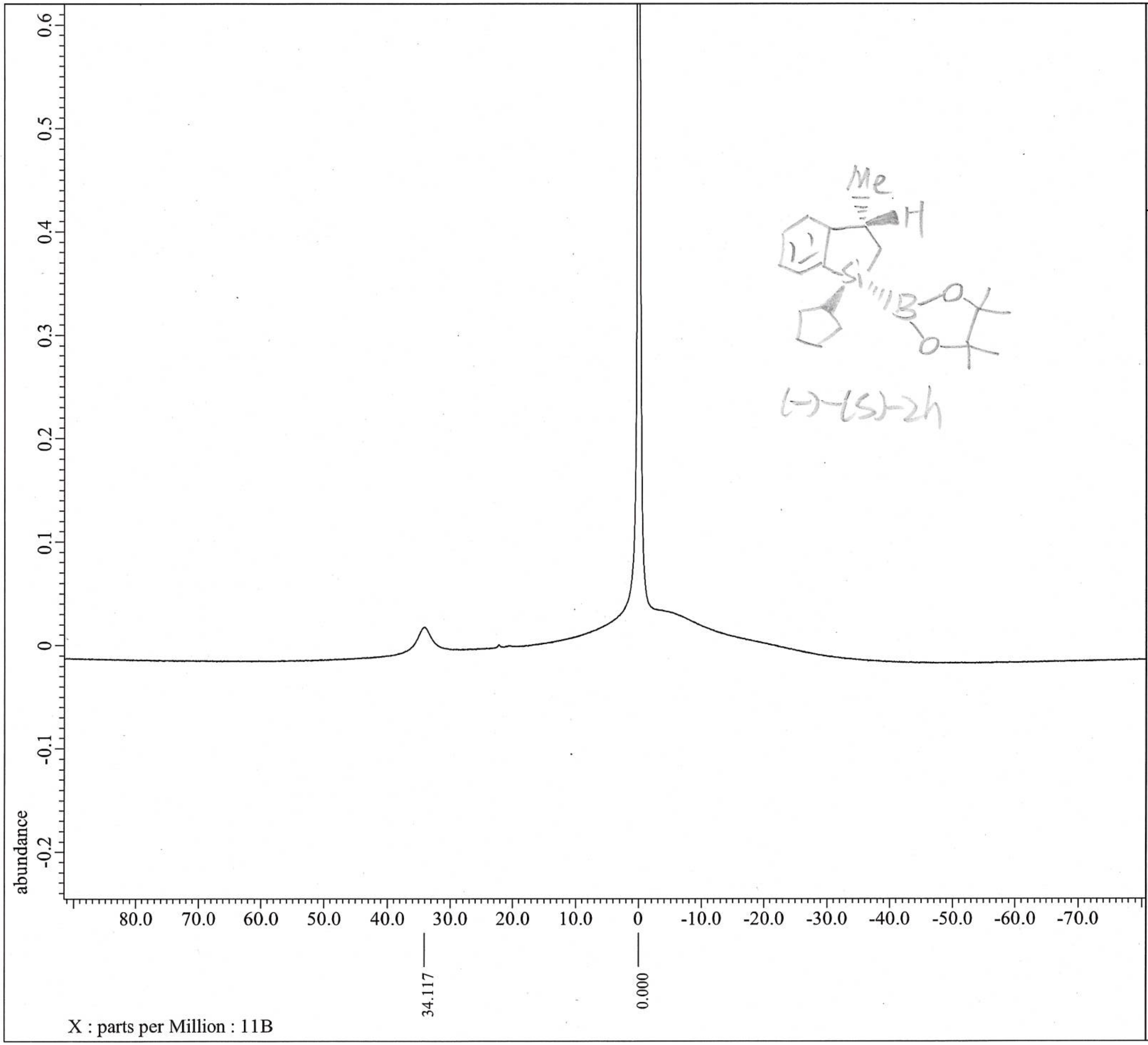

---- PROCESSING PARAMETERS ----
dc_balance(0, FALSE)
sexp(2.0[Hz], 0.0[s])
trapezoid3(0[%], 80[%], 100[%])
zerofill(1, TRUE)
fft(1, TRUE, TRUE)
machinephase
ppm

数据来源: wxh-407-B-1.jdf

Filename	= wxh-407-B-2.jdf
Author	= element
Experiment	= single_pulse_dec
Sample_Id	= S#485784
Solvent	= CHLOROFORM-D
Actual_Start_Time	= 9-FEB-2023 22:19:11
Revision_Time	= 13-FEB-2023 14:30:45
Comment	= single pulse decoupled ga
Data_Format	= 1D COMPLEX
Dim_Size	= 26214
X_Domain	= 11B
Dim_Title	= 11B
Dim_Units	= [ppm]
Dimensions	= X
Site	= ECS 400
Spectrometer	= JNM-ECS400
Field_Strength	= 9.20197068[T] (390[MHz])
X_Acq_Duration	= 0.83361792[s]
X_Domain	= 11B
X_Freq	= 125.70081325[MHz]
X_Offset	= 0[ppm]
X_Points	= 32768
X_Prescans	= 4
X_Resolution	= 1.19959034[Hz]
X_Sweep	= 39.3081761[kHz]
Irr_Domain	= 1H
Irr_Freq	= 391.78655441[MHz]
Irr_Offset	= 5[ppm]
Clipped	= FALSE
Scans	= 1800
Total_Scans	= 1800
Relaxation_Delay	= 2[s]
Recvr_Gain	= 40
Temp_Get	= 18.4[dC]
X_90_Width	= 10[us]
X_Acq_Time	= 0.83361792[s]
X_Angle	= 30[deg]
X_Atn	= 5.5[dB]
X_Pulse	= 3.33333333[us]
Irr_Atn_Dec	= 22.45[dB]
Irr_Atn_Noe	= 22.45[dB]
Irr_Noise	= WALTZ
Decoupling	= TRUE
Initial_Wait	= 1[s]
Noe	= TRUE
Noe_Time	= 2[s]
Repetition_Time	= 2.83361792[s]

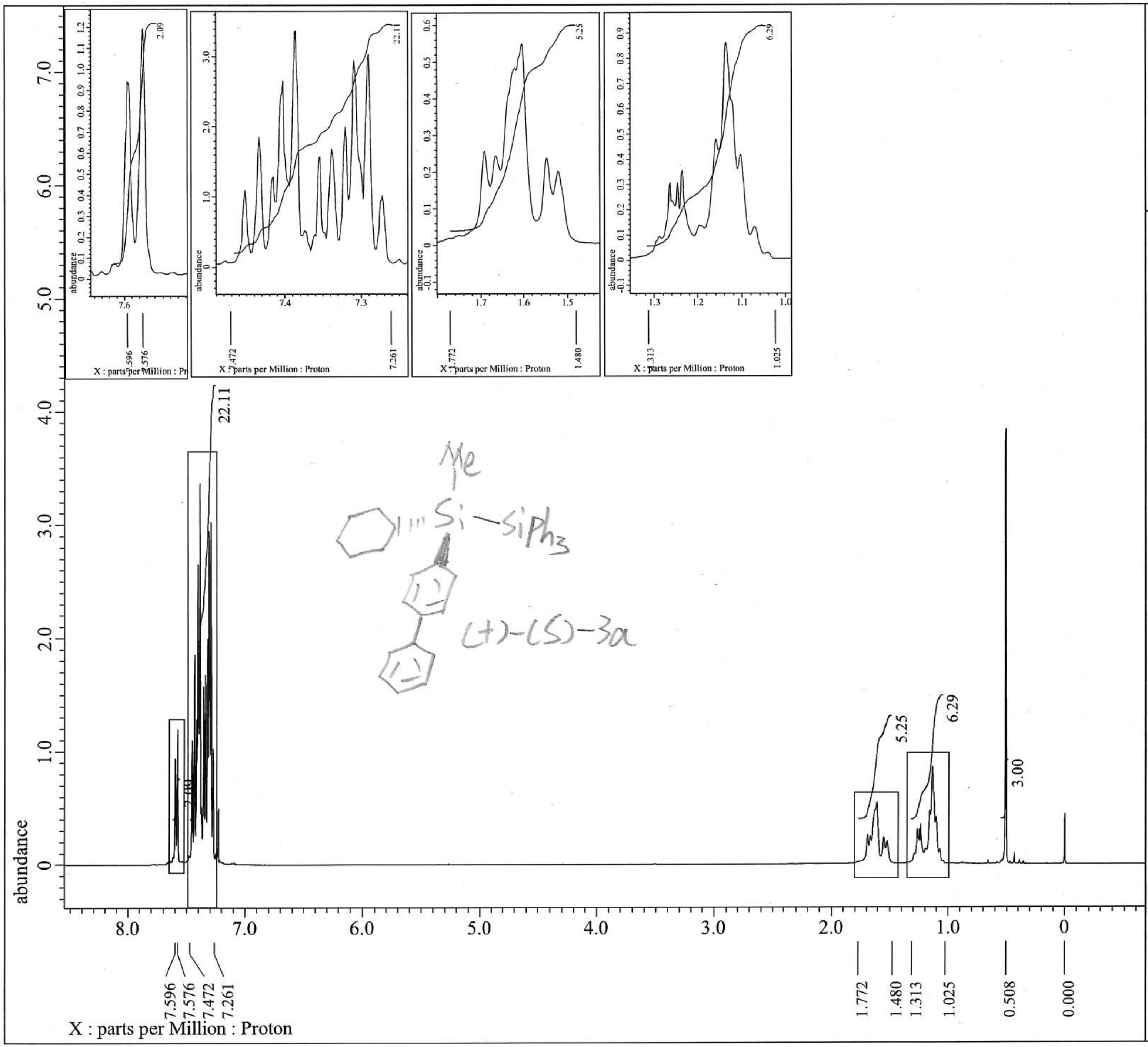

---- PROCESSING PARAMETERS ----
dc_balance(0, FALSE)
sexp(0.2[Hz], 0.0[s])
trapezoid(0[%], 0[%], 80[%], 100[%])
zerofill(1, TRUE)
fft(1, TRUE, TRUE)
machinephase
ppm

数据来源: wxh-139-3_Proton-1-1.jdf

Filename	= wxh-139-3_Proton-1-2.jdf
Author	= element
Experiment	= proton.jxp
Sample_Id	= wxh-139
Solvent	= CHLOROFORM-D
Actual_Start_Time	= 24-NOV-2021 11:19:02
Revision_Time	= 28-JUN-2023 14:27:39
Comment	= single_pulse
Data_Format	= 1D COMPLEX
Dim_Size	= 13107
X_Domain	= Proton
Dim_Title	= Proton
Dim_Units	= [ppm]
Dimensions	= X
Spectrometer	= DELTA2_NMR
Field_Strength	= 9.4073814[T] (400[MHz])
X_Acq_Duration	= 2.18103808[s]
X_Domain	= 1H
X_Freq	= 400.53219825[MHz]
X_Offset	= 5[ppm]
X_Points	= 16384
X_Prescans	= 1
X_Resolution	= 0.45849727[Hz]
X_Sweep	= 7.51201923[kHz]
X_Sweep_Clipped	= 6.00961538[kHz]
Irr_Domain	= Proton
Irr_Freq	= 400.53219825[MHz]
Irr_Offset	= 5[ppm]
Tri_Domain	= Proton
Tri_Freq	= 400.53219825[MHz]
Tri_Offset	= 5[ppm]
Clipped	= FALSE
Scans	= 8
Total_Scans	= 8
Relaxation_Delay	= 5[s]
Recvr_Gain	= 36
Temp_Get	= 18.7[dC]
X_90_Width	= 6[us]
X_Acq_Time	= 2.18103808[s]
X_Angle	= 45[deg]
X_Atn	= 0.8[dB]
X_Pulse	= 3[us]
Irr_Mode	= Off
Tri_Mode	= Off
Dante_Presat	= FALSE
Initial_Wait	= 1[s]
Repetition_Time	= 7.18103808[s]

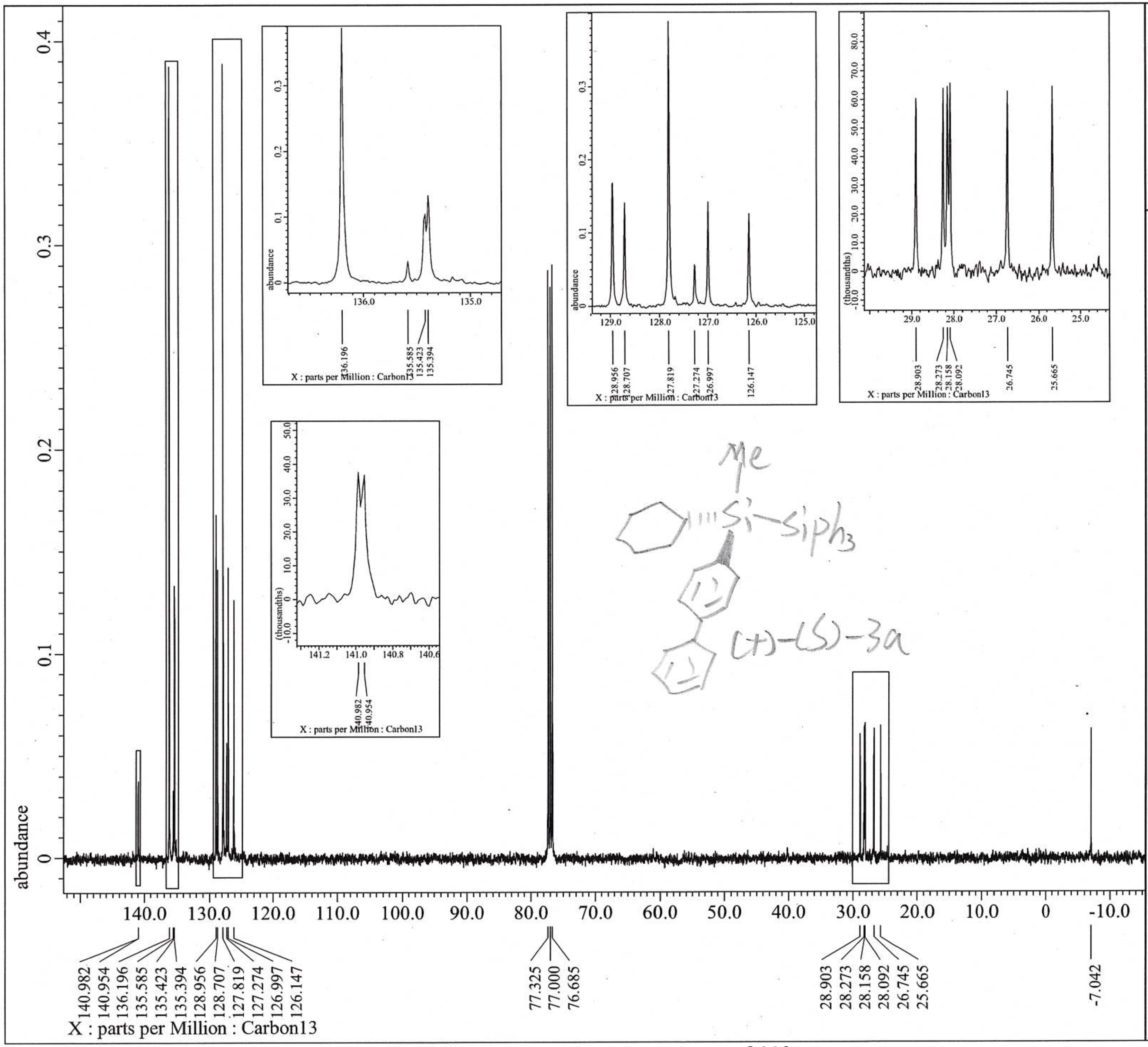


```
---- PROCESSING PARAMETERS ----
dc_balance( 0, FALSE )
sexp( 2.0[Hz], 0.0[s] )
trapezoid( 0[%], 0[%], 80[%], 100[%] )
zerofill( 1, TRUE )
fft( 1, TRUE, TRUE )
machinephase
ppm

数据来源: wxh-139-3_Carbon-1-1.jdf
```

```
Filename            = wxh-139-3_Carbon-1-2.jdf
Author              = element
Experiment          = carbon.jxp
Sample_Id           = WXH-139-3
Solvent             = CHLOROFORM-D
Actual_Start_Time   = 25-NOV-2021 18:02:02
Revision_Time       =  6-DEC-2021 17:44:33

Comment             = single pulse decoupled ga
Data_Format         = 1D COMPLEX
Dim_Size            = 26214
X_Domain            = Carbon
Dim_Title           = Carbon13
Dim_Units           = [ppm]
Dimensions          = X
Site                = JNM-ECS400
Spectrometer        = DELTA2_NMR

Field_Strength      = 9.37221[T] (400[MHz])
X_Acq_Duration      = 1.04333312[s]
X_Domain            = 13C
X_Freq              = 100.33735165[MHz]
X_Offset            = 100.0[ppm]
X_Points            = 32768
X_Prescans          = 4
X_Resolution        = 0.95846665[Hz]
X_Sweep             = 31.40703518[kHz]
X_Sweep_Clipped     = 25.12562814[kHz]
Irr_Domain          = Proton
Irr_Freq            = 399.03472754[MHz]
Irr_Offset          = 5.0[ppm]
Clipped             = FALSE
Scans               = 256
Total_Scans         = 256

Relaxation_Delay    = 2[s]
Recvr_Gain          = 50
Temp_Get            = 20.7[dC]
X_90_Width          = 10.9[us]
X_Acq_Time          = 1.04333312[s]
X_Angle             = 30[deg]
X_Atn               = 5.4[dB]
X_Pulse             = 3.63333333[us]
Irr_Atn_Dec         = 25.823[dB]
Irr_Atn_Noe         = 25.823[dB]
Irr_Noise           = WALTZ
Irr_Pwidth          = 0.115[ms]
Decoupling          = TRUE
Initial_Wait        = 1[s]
Noe                 = TRUE
Noe_Time            = 2[s]
Repetition_Time     = 3.04333312[s]
```

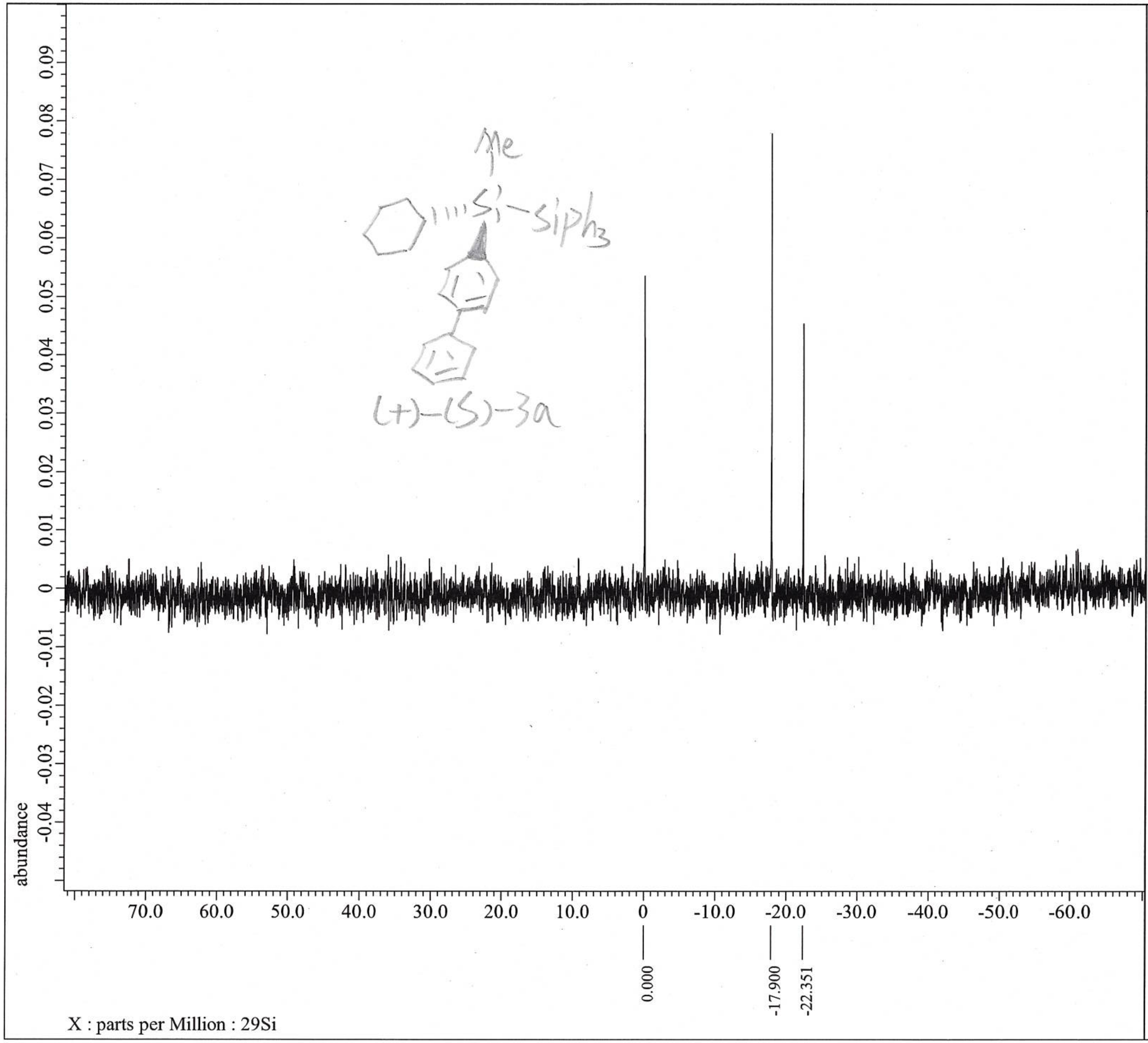

---- PROCESSING PARAMETERS ----
dc_balance(0, FALSE)
sexp(2.0[Hz], 0.0[s])
trapezoid3(0[%], 80[%], 100[%])
zerofill(1, TRUE)
fft(1, TRUE, TRUE)
machinephase
ppm
phase(52.10437, 0, 50[%])

数据来源: wxh-139-Si-1.jdf

Filename	= wxh-139-Si-2.jdf
Author	= element
Experiment	= single_pulse_dec
Sample_Id	= S#564766
Solvent	= CHLOROFORM-D
Actual_Start_Time	= 4-DEC-2021 22:26:40
Revision_Time	= 19-APR-2022 20:29:29
Comment	= single pulse decoupled ga
Data_Format	= 1D COMPLEX
Dim_Size	= 26214
X_Domain	= 29Si
Dim_Title	= 29Si
Dim_Units	= [ppm]
Dimensions	= X
Site	= ECS 400
Spectrometer	= JNM-ECS400
Field_Strength	= 9.20197068[T] (390[MHz])
X_Acq_Duration	= 1.34217728[s]
X_Domain	= 29Si
X_Freq	= 77.83692472[MHz]
X_Offset	= 0[ppm]
X_Points	= 32768
X_Prescans	= 4
X_Resolution	= 0.74505806[Hz]
X_Sweep	= 24.4140625[kHz]
Irr_Domain	= 1H
Irr_Freq	= 391.78655441[MHz]
Irr_Offset	= 5[ppm]
Clipped	= FALSE
Scans	= 600
Total_Scans	= 600
Relaxation_Delay	= 8[s]
Recvr_Gain	= 60
Temp_Get	= 20.5[dC]
X_90_Width	= 10[us]
X_Acq_Time	= 1.34217728[s]
X_Angle	= 30[deg]
X_Atn	= 4.9[dB]
X_Pulse	= 3.33333333[us]
Irr_Atn_Dec	= 22.45[dB]
Irr_Noise	= WALTZ
Decoupling	= TRUE
Initial_Wait	= 1[s]
Noe	= FALSE
Repetition_Time	= 9.34217728[s]

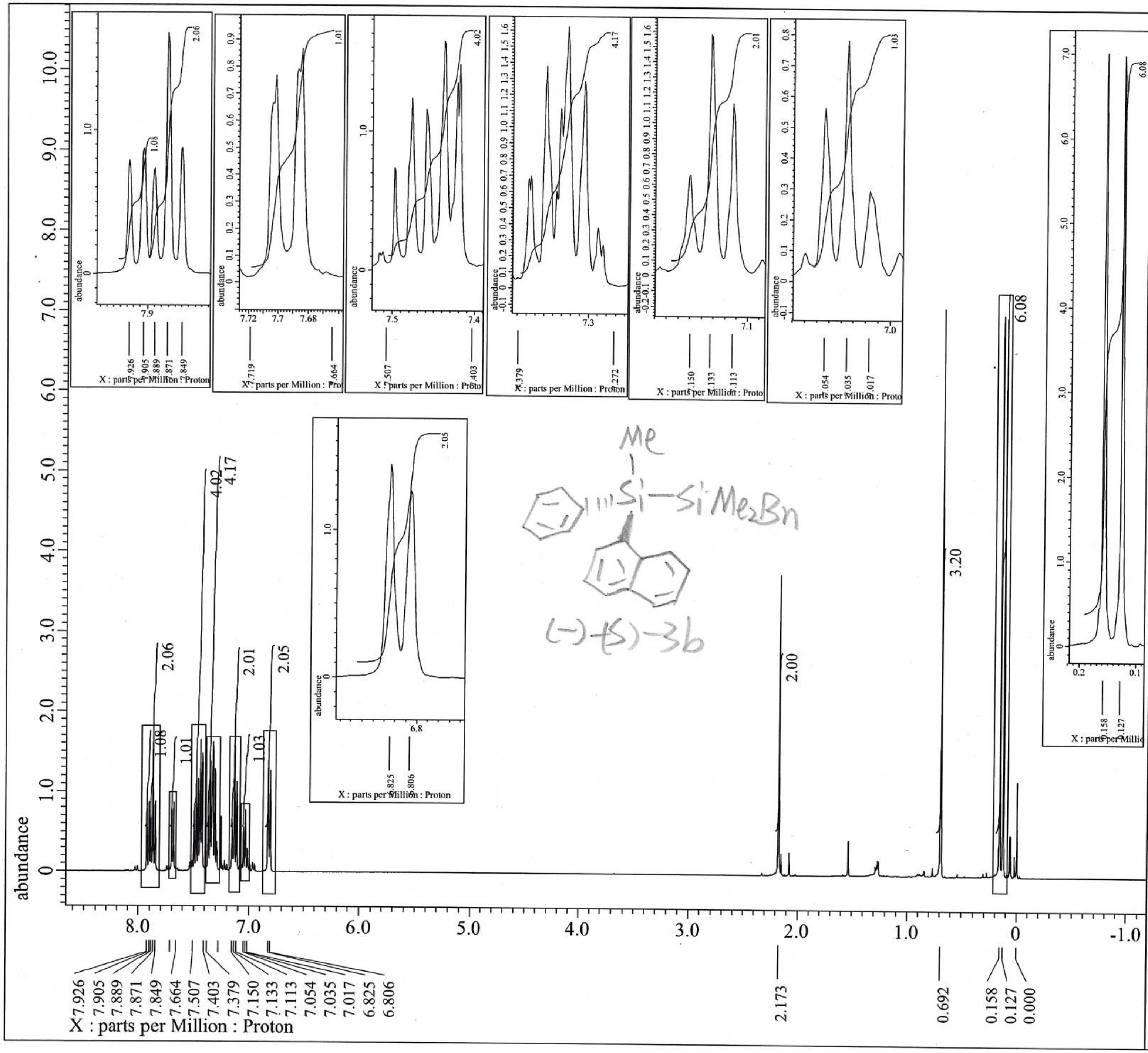

---- PROCESSING PARAMETERS ----
dc_balance(0, FALSE)
sexp(0.2[Hz], 0.0[s])
trapezoid(0[%], 0[%], 80[%], 100[%])
zerofill(1, TRUE)
fft(1, TRUE, TRUE)
machinephase
ppm

数据来源: wxh-228-2_Proton-1-1.jdf

Filename	= wxh-228-2_Proton-1-2.jdf
Author	= element
Experiment	= proton.jxp
Sample_Id	= wxh-228
Solvent	= CHLOROFORM-D
Actual_Start_Time	= 25-APR-2022 13:11:07
Revision_Time	= 28-JUN-2023 14:45:20
Comment	= single_pulse
Data_Format	= 1D COMPLEX
Dim_Size	= 13107
X_Domain	= Proton
Dim_Title	= Proton
Dim_Units	= [ppm]
Dimensions	= X
Site	= JNM-ECS400
Spectrometer	= DELTA2_NMR
Field_Strength	= 9.37221[T] (400[MHz])
X_Acq_Duration	= 2.1889024[s]
X_Domain	= 1H
X_Freq	= 399.03472754[MHz]
X_Offset	= 5.0[ppm]
X_Points	= 16384
X_Prescans	= 1
X_Resolution	= 0.45684997[Hz]
X_Sweep	= 7.48502994[kHz]
X_Sweep_Clipped	= 5.98802395[kHz]
Irr_Domain	= Proton
Irr_Freq	= 399.03472754[MHz]
Irr_Offset	= 5.0[ppm]
Tri_Domain	= Proton
Tri_Freq	= 399.03472754[MHz]
Tri_Offset	= 5.0[ppm]
Clipped	= FALSE
Scans	= 8
Total_Scans	= 8
Relaxation_Delay	= 5[s]
Recvr_Gain	= 30
Temp_Get	= 17.4[dC]
X_90_Width	= 6.6[us]
X_Acq_Time	= 2.1889024[s]
X_Angle	= 45[deg]
X_Atn	= 1[dB]
X_Pulse	= 3.3[us]
Irr_Mode	= Off
Tri_Mode	= Off
Dante_Presat	= FALSE
Initial_Wait	= 1[s]
Repetition_Time	= 7.1889024[s]

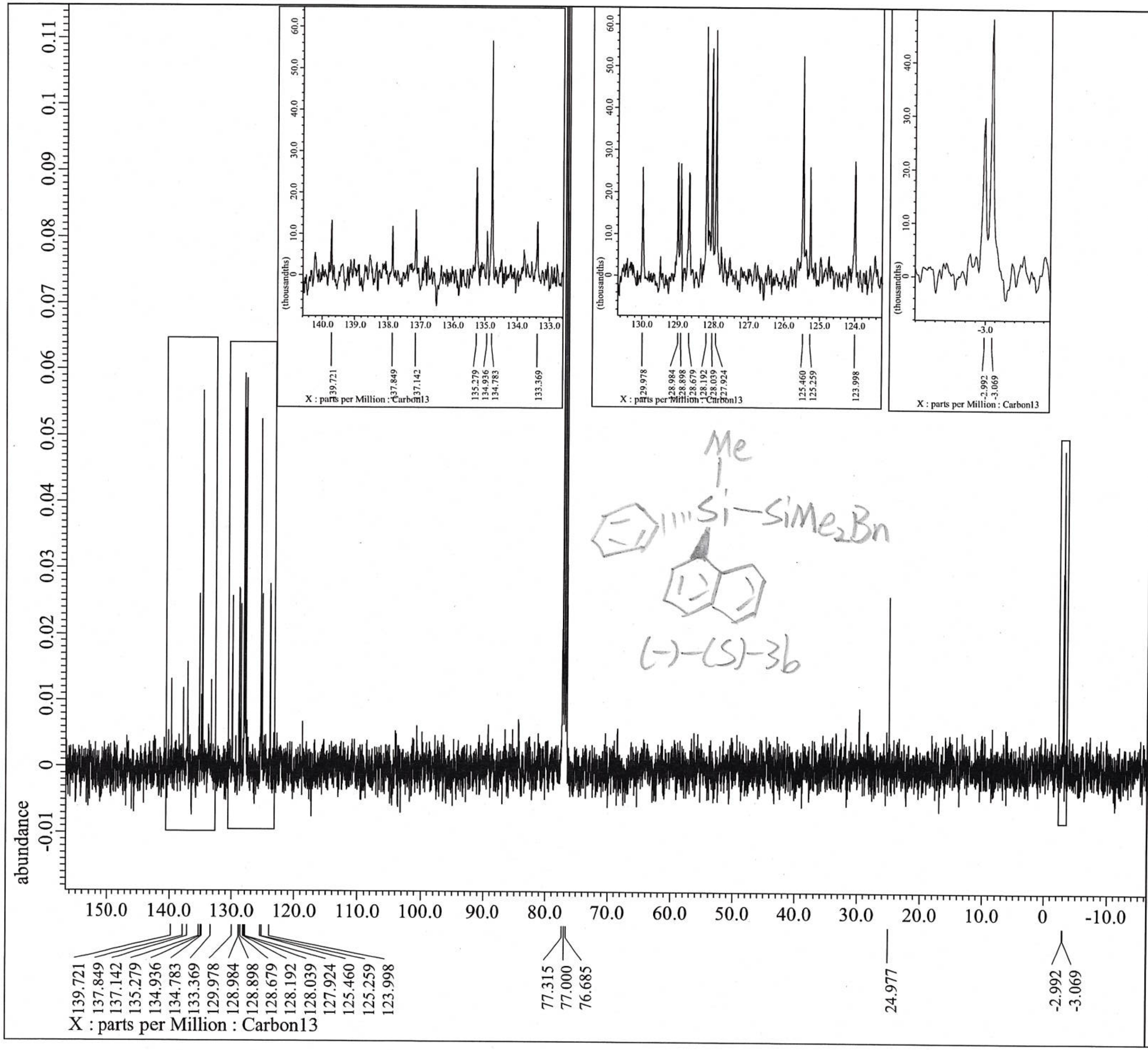

---- PROCESSING PARAMETERS ----
dc_balance(0, FALSE)
sexp(2.0[Hz], 0.0[s])
trapezoid(0[%], 0[%], 80[%], 100[%])
zerofill(1, TRUE)
fft(1, TRUE, TRUE)
machinephase
ppm

数据来源: wxh-228-4_Carbon-1-1.jdf

Filename	= wxh-228-4_Carbon-1-2.jdf
Author	= element
Experiment	= carbon.jxp
Sample_Id	= wxh-228-4
Solvent	= CHLOROFORM-D
Actual_Start_Time	= 10-MAY-2022 11:58:39
Revision_Time	= 10-MAY-2022 12:32:49
Comment	= single pulse decoupled ga
Data_Format	= 1D COMPLEX
Dim_Size	= 26214
X_Domain	= Carbon
Dim_Title	= Carbon13
Dim_Units	= [ppm]
Dimensions	= X
Site	= JNM-ECS400
Spectrometer	= DELTA2_NMR
Field_Strength	= 9.37221[T] (400[MHz])
X_Acq_Duration	= 1.04333312[s]
X_Domain	= 13C
X_Freq	= 100.33735165[MHz]
X_Offset	= 100.0[ppm]
X_Points	= 32768
X_Prescans	= 4
X_Resolution	= 0.95846665[Hz]
X_Sweep	= 31.40703518[kHz]
X_Sweep_Clipped	= 25.12562814[kHz]
Irr_Domain	= Proton
Irr_Freq	= 399.03472754[MHz]
Irr_Offset	= 5.0[ppm]
Clipped	= FALSE
Scans	= 128
Total_Scans	= 128
Relaxation_Delay	= 2[s]
Recvr_Gain	= 50
Temp_Get	= 17.6[dC]
X_90_Width	= 10.9[us]
X_Acq_Time	= 1.04333312[s]
X_Angle	= 30[deg]
X_Atn	= 5.4[dB]
X_Pulse	= 3.63333333[us]
Irr_Atn_Dec	= 25.823[dB]
Irr_Atn_Noe	= 25.823[dB]
Irr_Noise	= WALTZ
Irr_Pwidth	= 0.115[ms]
Decoupling	= TRUE
Initial_Wait	= 1[s]
Noe	= TRUE
Noe_Time	= 2[s]
Repetition_Time	= 3.04333312[s]

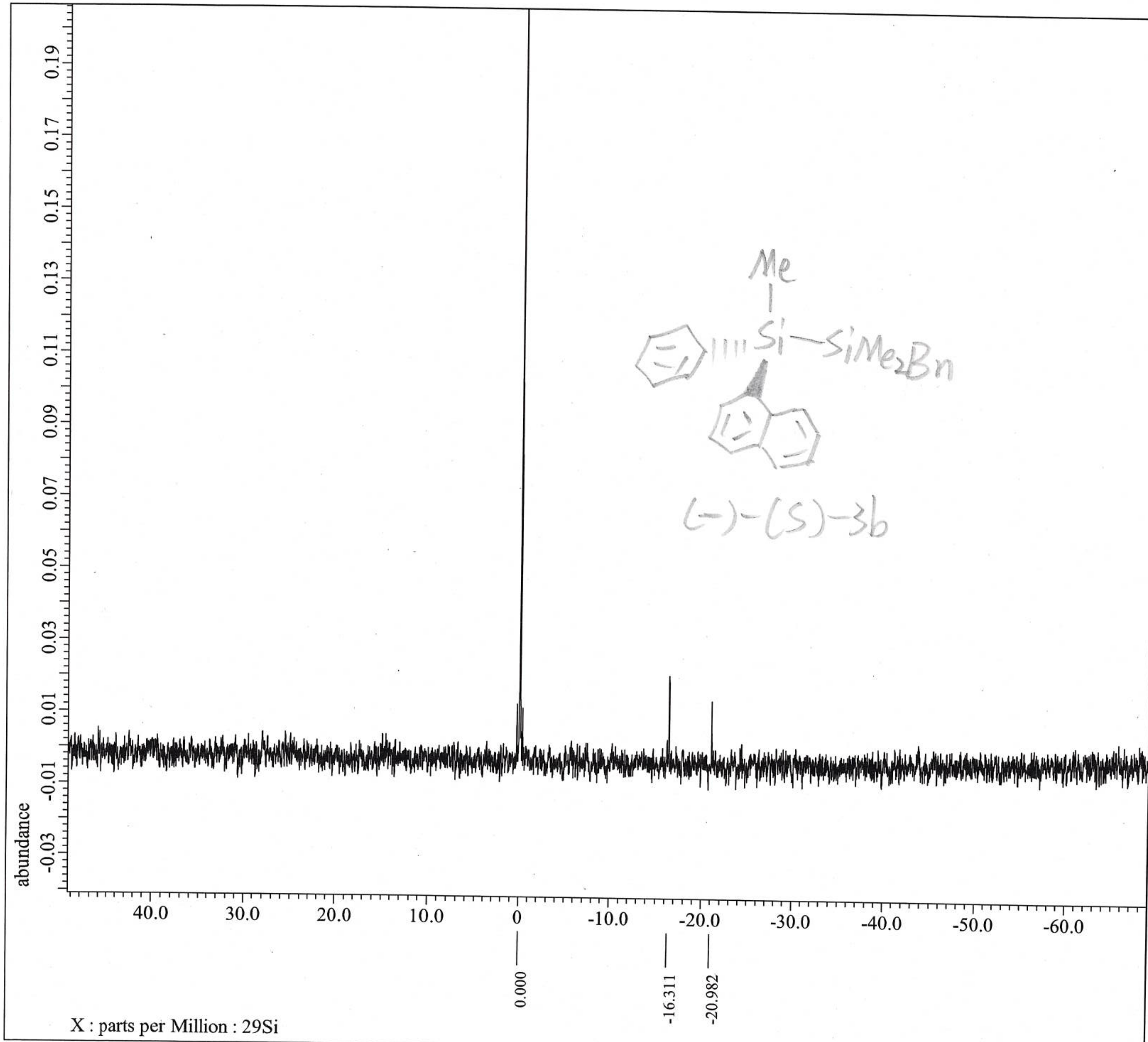


```
---- PROCESSING PARAMETERS ----
dc_balance( 0, FALSE )
sexp( 2.0[Hz], 0.0[s] )
trapezoid3( 0[%], 80[%], 100[%] )
zerofill( 1, TRUE )
fft( 1, TRUE, TRUE )
machinephase
ppm
phase( 70.17509, 0, 50[%] )
reference( 0.52646[ppm], 0[ppm] )
reference( 0[ppm], 0[ppm] )

数据来源: wxh-228-Si-1.jdf
```

```
Filename            = wxh-228-Si-2.jdf
Author              = element
Experiment          = single_pulse_dec
Sample_Id           = S#538483
Solvent             = CHLOROFORM-D
Actual_Start_Time   = 10-MAY-2022 21:47:19
Revision_Time       = 10-MAY-2022 16:59:00

Comment             = single pulse decoupled ga
Data_Format         = 1D COMPLEX
Dim_Size            = 26214
X_Domain            = 29Si
Dim_Title           = 29Si
Dim_Units           = [ppm]
Dimensions          = X
Site                = ECS 400
Spectrometer        = JNM-ECS400

Field_Strength      = 9.20197068[T] (390[MHz])
X_Acq_Duration      = 1.34217728[s]
X_Domain            = 29Si
X_Freq              = 77.83692472[MHz]
X_Offset            = 0[ppm]
X_Points            = 32768
X_Prescans          = 4
X_Resolution        = 0.74505806[Hz]
X_Sweep             = 24.4140625[kHz]
Irr_Domain          = 1H
Irr_Freq            = 391.78655441[MHz]
Irr_Offset          = 5[ppm]
Clipped             = FALSE
Scans               = 600
Total_Scans         = 600

Relaxation_Delay    = 9[s]
Recvr_Gain          = 60
Temp_Get            = 17.8[dC]
X_90_Width          = 10[us]
X_Acq_Time          = 1.34217728[s]
X_Angle             = 30[deg]
X_Atn               = 4.9[dB]
X_Pulse             = 3.33333333[us]
Irr_Atn_Dec         = 22.45[dB]
Irr_Noise           = WALTZ
Decoupling          = TRUE
Initial_Wait        = 1[s]
Noe                 = FALSE
Repetition_Time     = 10.34217728[s]
```

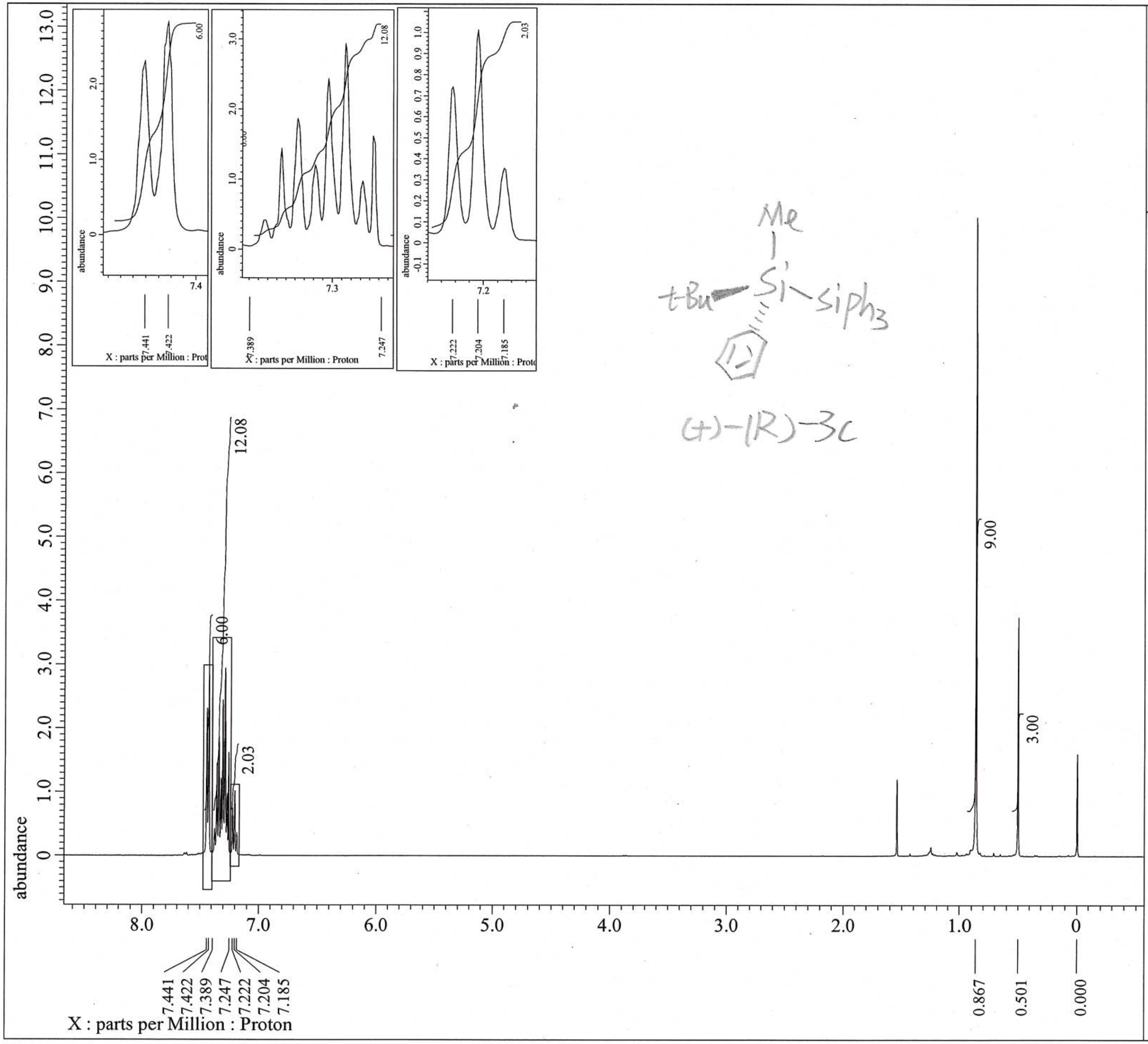

---- PROCESSING PARAMETERS ----
dc_balance(0, FALSE)
sexp(0.2[Hz], 0.0[s])
trapezoid(0[%], 0[%], 80[%], 100[%])
zerofill(1, TRUE)
fft(1, TRUE, TRUE)
machinephase
ppm

数据来源: wxh-189-3_Proton-1-1.jdf

Filename	= wxh-189-3_Proton-1-2.jdf
Author	= element
Experiment	= proton.jxp
Sample_Id	= wxh-189-4
Solvent	= CHLOROFORM-D
Actual_Start_Time	= 3-DEC-2021 16:32:10
Revision_Time	= 28-JUN-2023 14:56:29
Comment	= single_pulse
Data_Format	= 1D COMPLEX
Dim_Size	= 13107
X_Domain	= Proton
Dim_Title	= Proton
Dim_Units	= [ppm]
Dimensions	= X
Spectrometer	= DELTA2_NMR
Field_Strength	= 9.4073814[T] (400[MHz])
X_Acq_Duration	= 2.18103808[s]
X_Domain	= 1H
X_Freq	= 400.53219825[MHz]
X_Offset	= 5[ppm]
X_Points	= 16384
X_Prescans	= 1
X_Resolution	= 0.45849727[Hz]
X_Sweep	= 7.51201923[kHz]
X_Sweep_Clipped	= 6.00961538[kHz]
Irr_Domain	= Proton
Irr_Freq	= 400.53219825[MHz]
Irr_Offset	= 5[ppm]
Tri_Domain	= Proton
Tri_Freq	= 400.53219825[MHz]
Tri_Offset	= 5[ppm]
Clipped	= FALSE
Scans	= 8
Total_Scans	= 8
Relaxation_Delay	= 5[s]
Recvr_Gain	= 40
Temp_Get	= 18.6[dC]
X_90_Width	= 6[us]
X_Acq_Time	= 2.18103808[s]
X_Angle	= 45[deg]
X_Atn	= 0.8[dB]
X_Pulse	= 3[us]
Irr_Mode	= Off
Tri_Mode	= Off
Dante_Presat	= FALSE
Initial_Wait	= 1[s]
Repetition_Time	= 7.18103808[s]

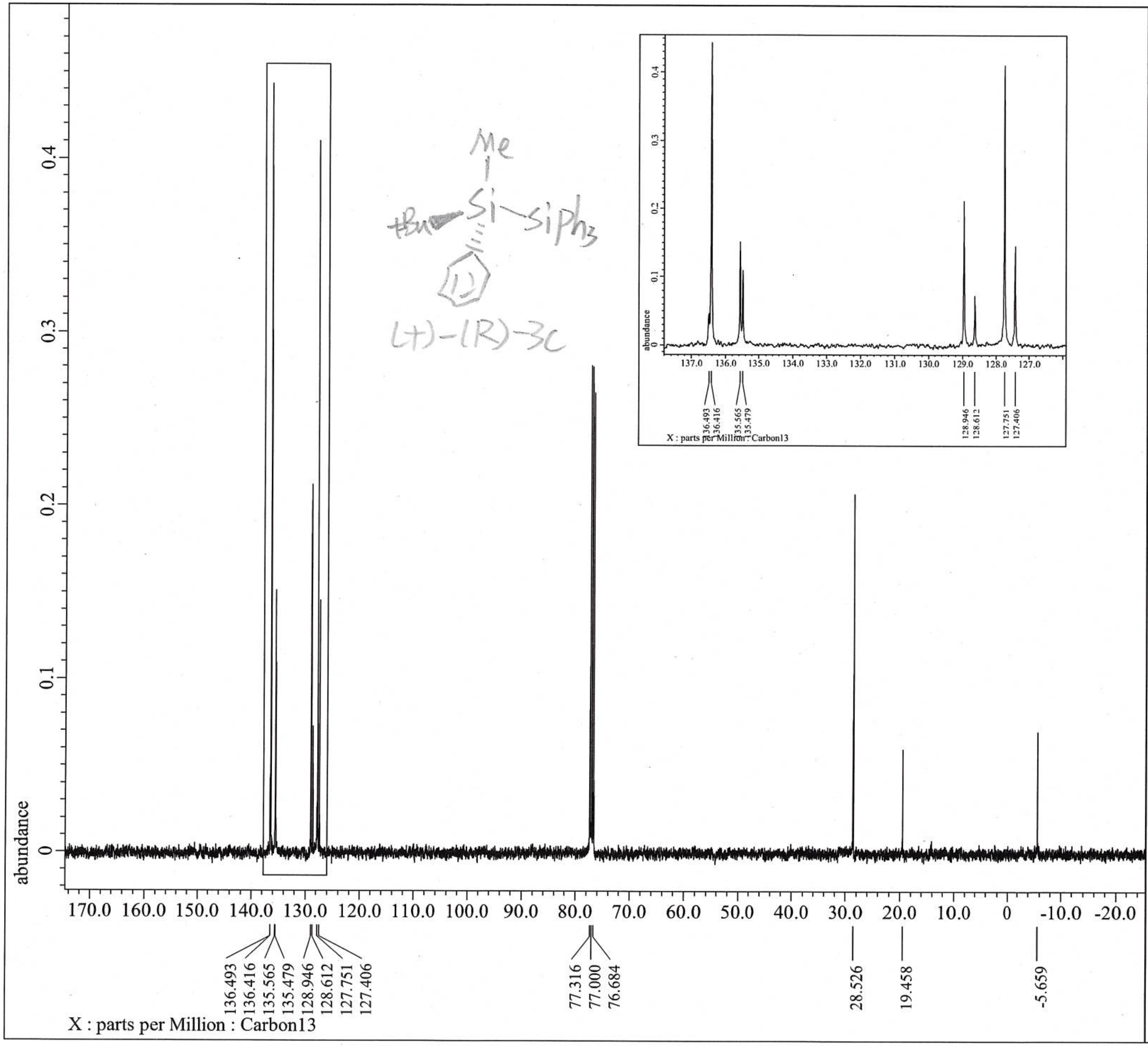


```
---- PROCESSING PARAMETERS ----
dc_balance( 0, FALSE )
sexp( 2.0[Hz], 0.0[s] )
trapezoid( 0[%], 0[%], 80[%], 100[%] )
zerofill( 1, TRUE )
fft( 1, TRUE, TRUE )
machinephase
ppm

数据来源: wxh-189-3_Carbon-1-1.jdf
```

```
Filename            = wxh-189-3_Carbon-1-2.jdf
Author              = element
Experiment          = carbon.jxp
Sample_Id           = wxh-189-3
Solvent             = CHLOROFORM-D
Actual_Start_Time   = 18-DEC-2021 14:45:15
Revision_Time       =  5-JAN-2022 17:27:24

Comment             = single pulse decoupled ga
Data_Format         = 1D COMPLEX
Dim_Size            = 26214
X_Domain            = Carbon
Dim_Title           = Carbon13
Dim_Units           = [ppm]
Dimensions          = X
Spectrometer        = DELTA2_NMR

Field_Strength      = 9.4073814[T] (400[MHz])
X_Acq_Duration      = 1.03809024[s]
X_Domain            = 13C
X_Freq              = 100.71389092[MHz]
X_Offset            = 100[ppm]
X_Points            = 32768
X_Prescans          = 4
X_Resolution        = 0.96330739[Hz]
X_Sweep             = 31.56565657[kHz]
X_Sweep_Clipped     = 25.25252525[kHz]
Irr_Domain          = Proton
Irr_Freq            = 400.53219825[MHz]
Irr_Offset          = 5[ppm]
Clipped             = FALSE
Scans               = 128
Total_Scans         = 128

Relaxation_Delay    = 2[s]
Recvr_Gain          = 50
Temp_Get            = 18[dC]
X_90_Width          = 10.9[us]
X_Acq_Time          = 1.03809024[s]
X_Angle             = 30[deg]
X_Atn               = 4[dB]
X_Pulse             = 3.63333333[us]
Irr_Atn_Dec         = 26.45[dB]
Irr_Atn_Noe         = 26.45[dB]
Irr_Noise           = WALTZ
Irr_Pwidth          = 0.115[ms]
Decoupling          = TRUE
Initial_Wait        = 1[s]
Noe                 = TRUE
Noe_Time            = 2[s]
Repetition_Time     = 3.03809024[s]
```

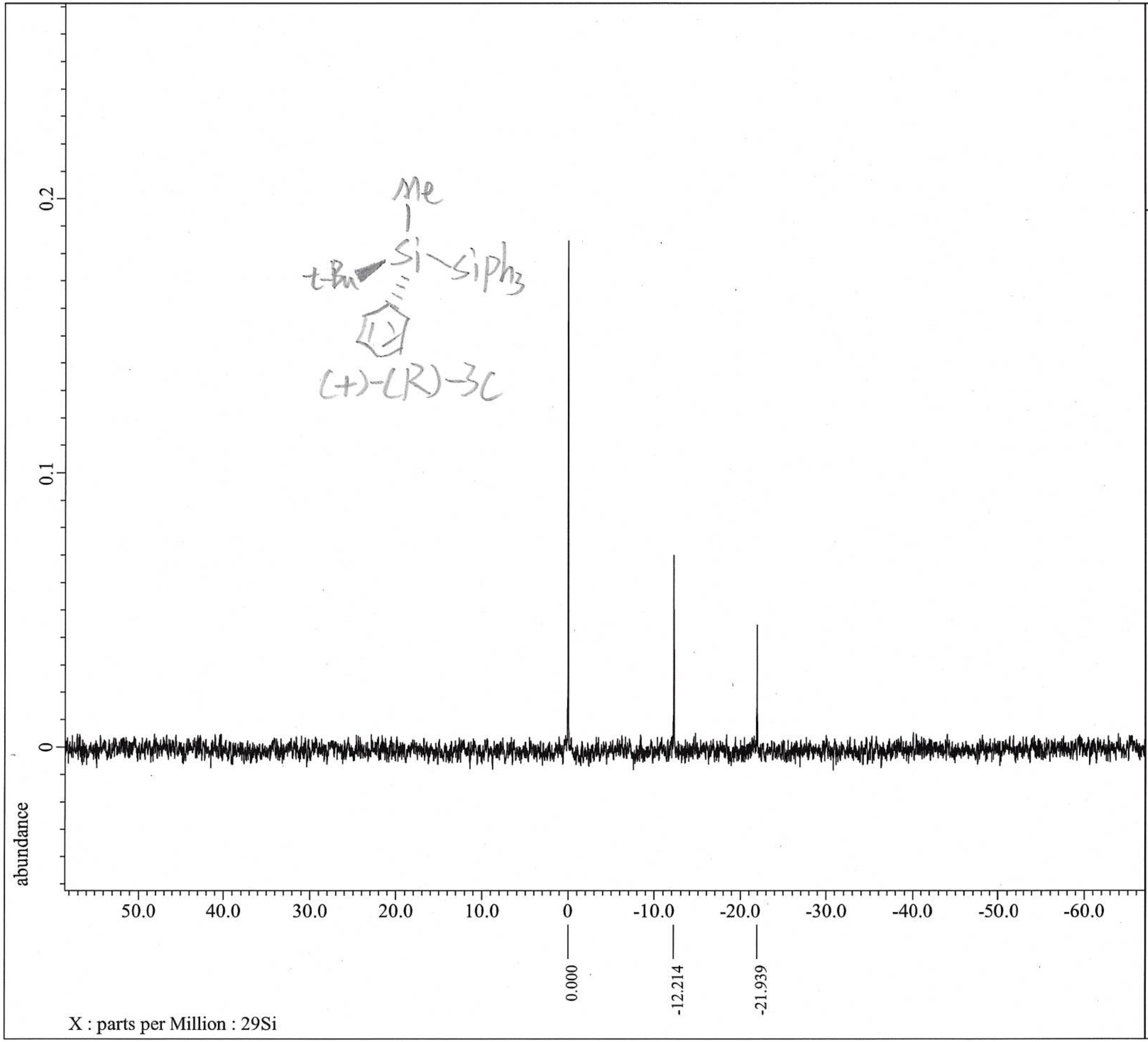


```
---- PROCESSING PARAMETERS ----
dc_balance( 0, FALSE )
sexp( 2.0[Hz], 0.0[s] )
trapezoid3( 0[%], 80[%], 100[%] )
zerofill( 1, TRUE )
fft( 1, TRUE, TRUE )
machinephase
ppm
phase( 32.45763, 0, 50[%] )

数据来源: wxh-189-Si-1.jdf
```

```
Filename            = wxh-189-Si-2.jdf
Author              = element
Experiment          = single_pulse_dec
Sample_Id           = S#754101
Solvent             = CHLOROFORM-D
Actual_Start_Time   = 10-MAY-2022 03:46:47
Revision_Time       = 10-MAY-2022 09:08:25

Comment             = single pulse decoupled ga
Data_Format         = 1D COMPLEX
Dim_Size            = 26214
X_Domain            = 29Si
Dim_Title           = 29Si
Dim_Units           = [ppm]
Dimensions          = X
Site                = ECS 400
Spectrometer        = JNM-ECS400

Field_Strength      = 9.20197068[T] (390[MHz])
X_Acq_Duration      = 1.34217728[s]
X_Domain            = 29Si
X_Freq              = 77.83692472[MHz]
X_Offset            = 0[ppm]
X_Points            = 32768
X_Prescans          = 4
X_Resolution        = 0.74505806[Hz]
X_Sweep             = 24.4140625[kHz]
Irr_Domain          = 1H
Irr_Freq            = 391.78655441[MHz]
Irr_Offset          = 5[ppm]
Clipped             = FALSE
Scans               = 600
Total_Scans         = 600

Relaxation_Delay    = 9[s]
Recvr_Gain          = 60
Temp_Get            = 17.9[dC]
X_90_Width          = 10[us]
X_Acq_Time          = 1.34217728[s]
X_Angle             = 30[deg]
X_Atn               = 4.9[dB]
X_Pulse             = 3.33333333[us]
Irr_Atn_Dec         = 22.45[dB]
Irr_Noise           = WALTZ
Decoupling          = TRUE
Initial_Wait        = 1[s]
Noe                 = FALSE
Repetition_Time     = 10.34217728[s]
```

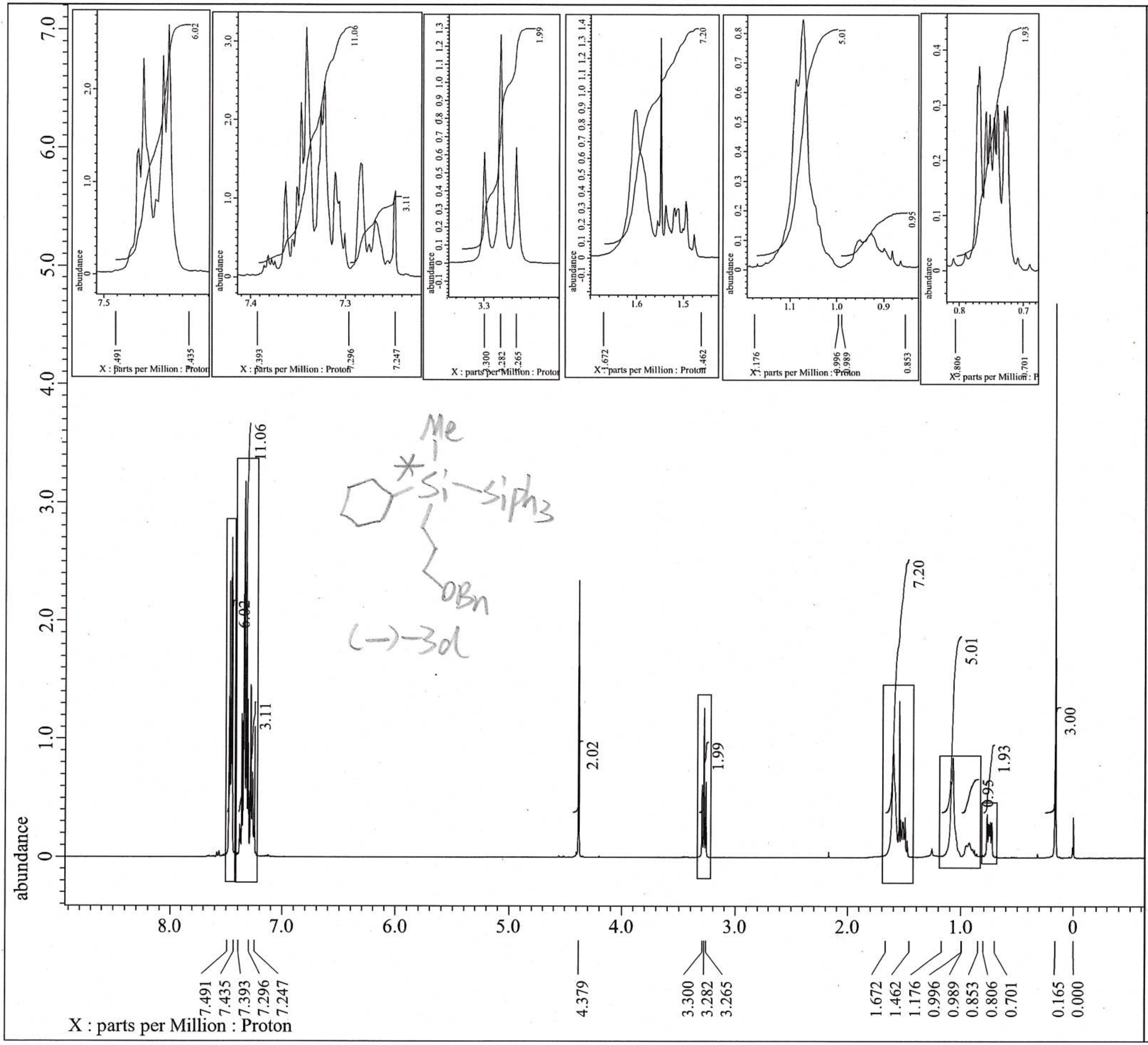

---- PROCESSING PARAMETERS ----
dc_balance(0, FALSE)
sexp(0.2[Hz], 0.0[s])
trapezoid(0[%], 0[%], 80[%], 100[%])
zerofill(1, TRUE)
fft(1, TRUE, TRUE)
machinephase
ppm

数据来源: wxh-253-2_Proton-1-1.jdf

Filename	=	wxh-253-2_Proton-1-2.jdf
Author	=	element
Experiment	=	proton.jxp
Sample_Id	=	wxh-253-2
Solvent	=	CHLOROFORM-D
Actual_Start_Time	=	25-APR-2022 14:10:38
Revision_Time	=	28-JUN-2023 15:13:49
Comment	=	single_pulse
Data_Format	=	1D COMPLEX
Dim_Size	=	13107
X_Domain	=	Proton
Dim_Title	=	Proton
Dim_Units	=	[ppm]
Dimensions	=	X
Site	=	JNM-ECS400
Spectrometer	=	DELTA2_NMR
Field_Strength	=	9.37221[T] (400[MHz])
X_Acq_Duration	=	2.1889024[s]
X_Domain	=	1H
X_Freq	=	399.03472754[MHz]
X_Offset	=	5.0[ppm]
X_Points	=	16384
X_Prescans	=	1
X_Resolution	=	0.45684997[Hz]
X_Sweep	=	7.48502994[kHz]
X_Sweep_Clipped	=	5.98802395[kHz]
Irr_Domain	=	Proton
Irr_Freq	=	399.03472754[MHz]
Irr_Offset	=	5.0[ppm]
Tri_Domain	=	Proton
Tri_Freq	=	399.03472754[MHz]
Tri_Offset	=	5.0[ppm]
Clipped	=	FALSE
Scans	=	8
Total_Scans	=	8
Relaxation_Delay	=	5[s]
Recvr_Gain	=	30
Temp_Get	=	19.3[dC]
X_90_Width	=	6.6[us]
X_Acq_Time	=	2.1889024[s]
X_Angle	=	45[deg]
X_Atn	=	1[dB]
X_Pulse	=	3.3[us]
Irr_Mode	=	Off
Tri_Mode	=	Off
Dante_Presat	=	FALSE
Initial_Wait	=	1[s]
Repetition_Time	=	7.1889024[s]

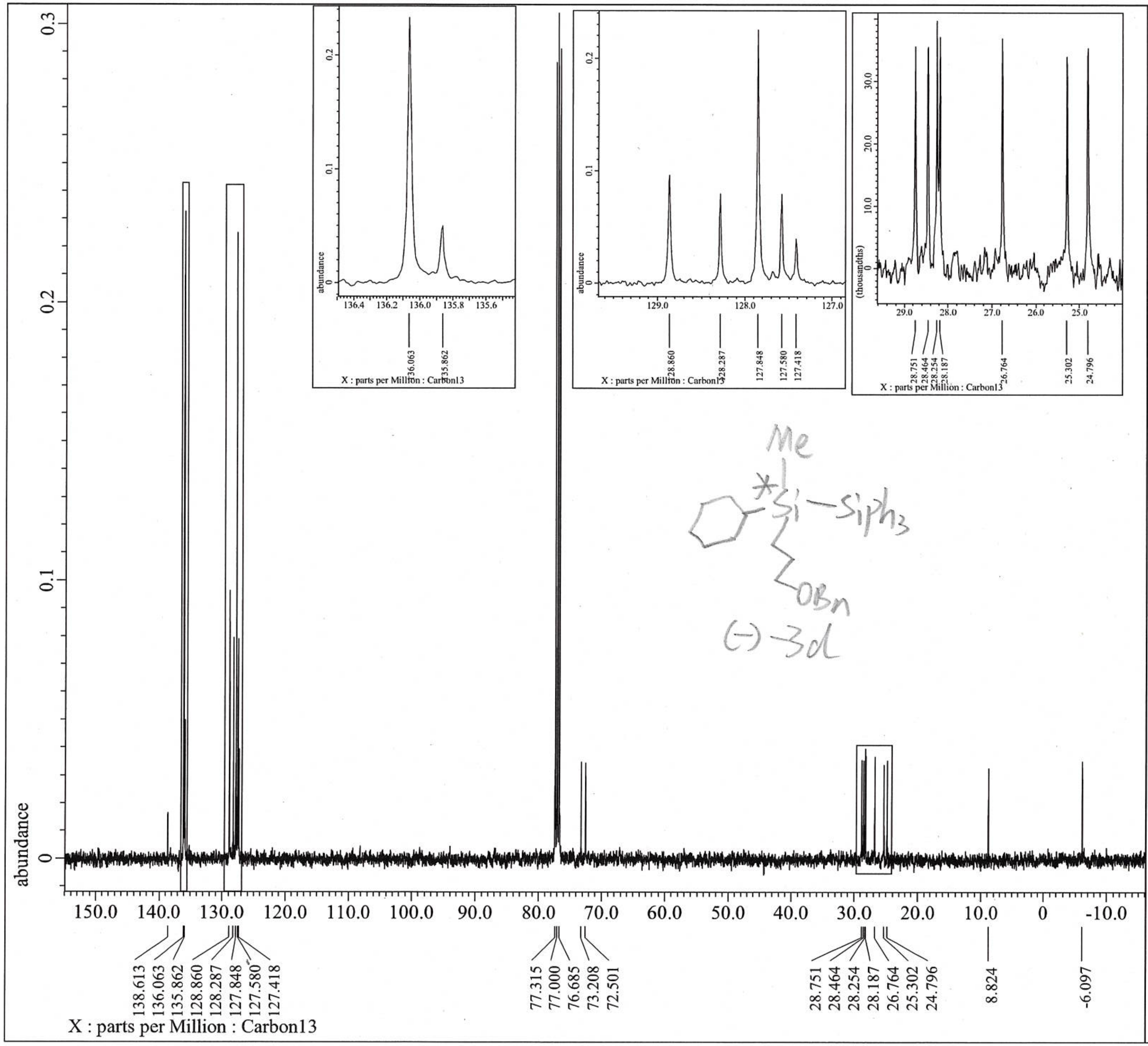


```
---- PROCESSING PARAMETERS ----
dc_balance( 0, FALSE )
sexp( 2.0[Hz], 0.0[s] )
trapezoid( 0[%], 0[%], 80[%], 100[%] )
zerofill( 1, TRUE )
fft( 1, TRUE, TRUE )
machinephase
ppm

数据来源: wxh-253-2_Carbon-1-1.jdf
```

```
Filename            = wxh-253-2_Carbon-1-2.jdf
Author              = element
Experiment          = carbon.jxp
Sample_Id           = wxh-253-2
Solvent             = CHLOROFORM-D
Actual_Start_Time   = 25-APR-2022 14:11:58
Revision_Time       =  6-MAY-2022 20:13:16

Comment             = single pulse decoupled ga
Data_Format         = 1D COMPLEX
Dim_Size            = 26214
X_Domain            = Carbon
Dim_Title           = Carbon13
Dim_Units           = [ppm]
Dimensions          = X
Site                = JNM-ECS400
Spectrometer        = DELTA2_NMR

Field_Strength      = 9.37221[T] (400[MHz])
X_Acq_Duration      = 1.04333312[s]
X_Domain            = 13C
X_Freq              = 100.33735165[MHz]
X_Offset            = 100.0[ppm]
X_Points            = 32768
X_Prescans          = 4
X_Resolution        = 0.95846665[Hz]
X_Sweep             = 31.40703518[kHz]
X_Sweep_Clipped     = 25.12562814[kHz]
Irr_Domain          = Proton
Irr_Freq            = 399.03472754[MHz]
Irr_Offset          = 5.0[ppm]
Clipped             = FALSE
Scans               = 256
Total_Scans         = 256

Relaxation_Delay    = 2[s]
Recvr_Gain          = 50
Temp_Get            = 19.6[dC]
X_90_Width          = 10.9[us]
X_Acq_Time          = 1.04333312[s]
X_Angle             = 30[deg]
X_Atn               = 5.4[dB]
X_Pulse             = 3.63333333[us]
Irr_Atn_Dec         = 25.823[dB]
Irr_Atn_Noe         = 25.823[dB]
Irr_Noise           = WALTZ
Irr_Pwidth          = 0.115[ms]
Decoupling          = TRUE
Initial_Wait        = 1[s]
Noe                 = TRUE
Noe_Time            = 2[s]
Repetition_Time     = 3.04333312[s]
```

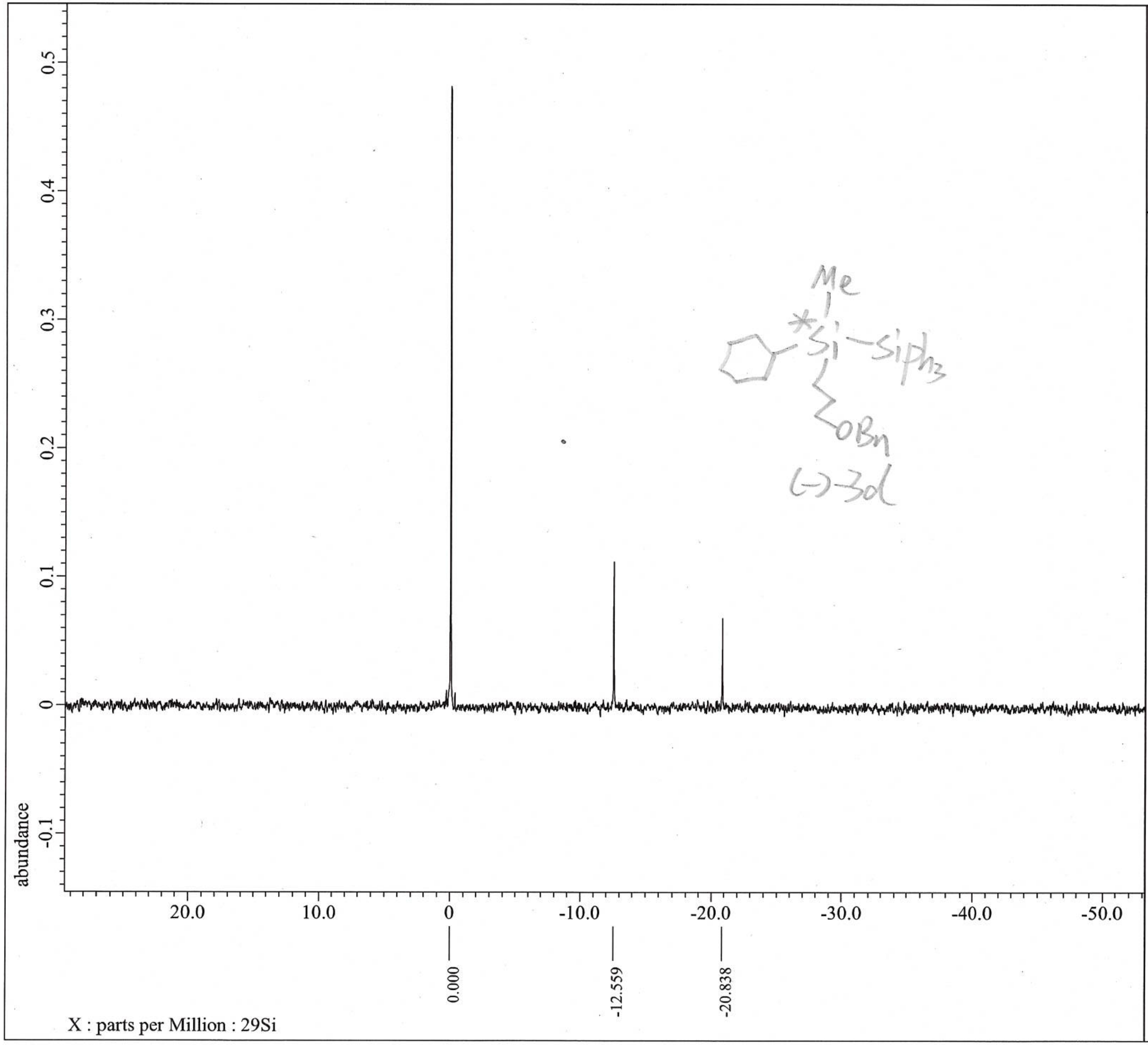


```
---- PROCESSING PARAMETERS ----
dc_balance( 0, FALSE )
sexp( 2.0[Hz], 0.0[s] )
trapezoid3( 0[%], 80[%], 100[%] )
zerofill( 1, TRUE )
fft( 1, TRUE, TRUE )
machinephase
ppm
phase( 60.45484, 0, 50[%] )

数据来源: wxh-253-Si-1.jdf

Filename            = wxh-253-Si-2.jdf
Author              = element
Experiment          = single_pulse_dec
Sample_Id           = S#501886
Solvent             = CHLOROFORM-D
Actual_Start_Time   = 25-APR-2022 20:42:38
Revision_Time       =  6-MAY-2022 20:34:58

Comment             = single pulse decoupled ga
Data_Format         = 1D COMPLEX
Dim_Size            = 26214
X_Domain            = 29Si
Dim_Title           = 29Si
Dim_Units           = [ppm]
Dimensions          = X
Site                = ECS 400
Spectrometer        = JNM-ECS400

Field_Strength      = 9.20197068[T] (390[MHz])
X_Acq_Duration      = 1.34217728[s]
X_Domain            = 29Si
X_Freq              = 77.83692472[MHz]
X_Offset            = 0[ppm]
X_Points            = 32768
X_Prescans          = 4
X_Resolution        = 0.74505806[Hz]
X_Sweep             = 24.4140625[kHz]
Irr_Domain          = 1H
Irr_Freq            = 391.78655441[MHz]
Irr_Offset          = 5[ppm]
Clipped             = FALSE
Scans               = 600
Total_Scans         = 600

Relaxation_Delay    = 9[s]
Recvr_Gain          = 60
Temp_Get            = 17.6[dC]
X_90_Width          = 10[us]
X_Acq_Time          = 1.34217728[s]
X_Angle             = 30[deg]
X_Atn               = 4.9[dB]
X_Pulse             = 3.33333333[us]
Irr_Atn_Dec         = 22.45[dB]
Irr_Noise           = WALTZ
Decoupling          = TRUE
Initial_Wait        = 1[s]
Noe                 = FALSE
Repetition_Time     = 10.34217728[s]
```

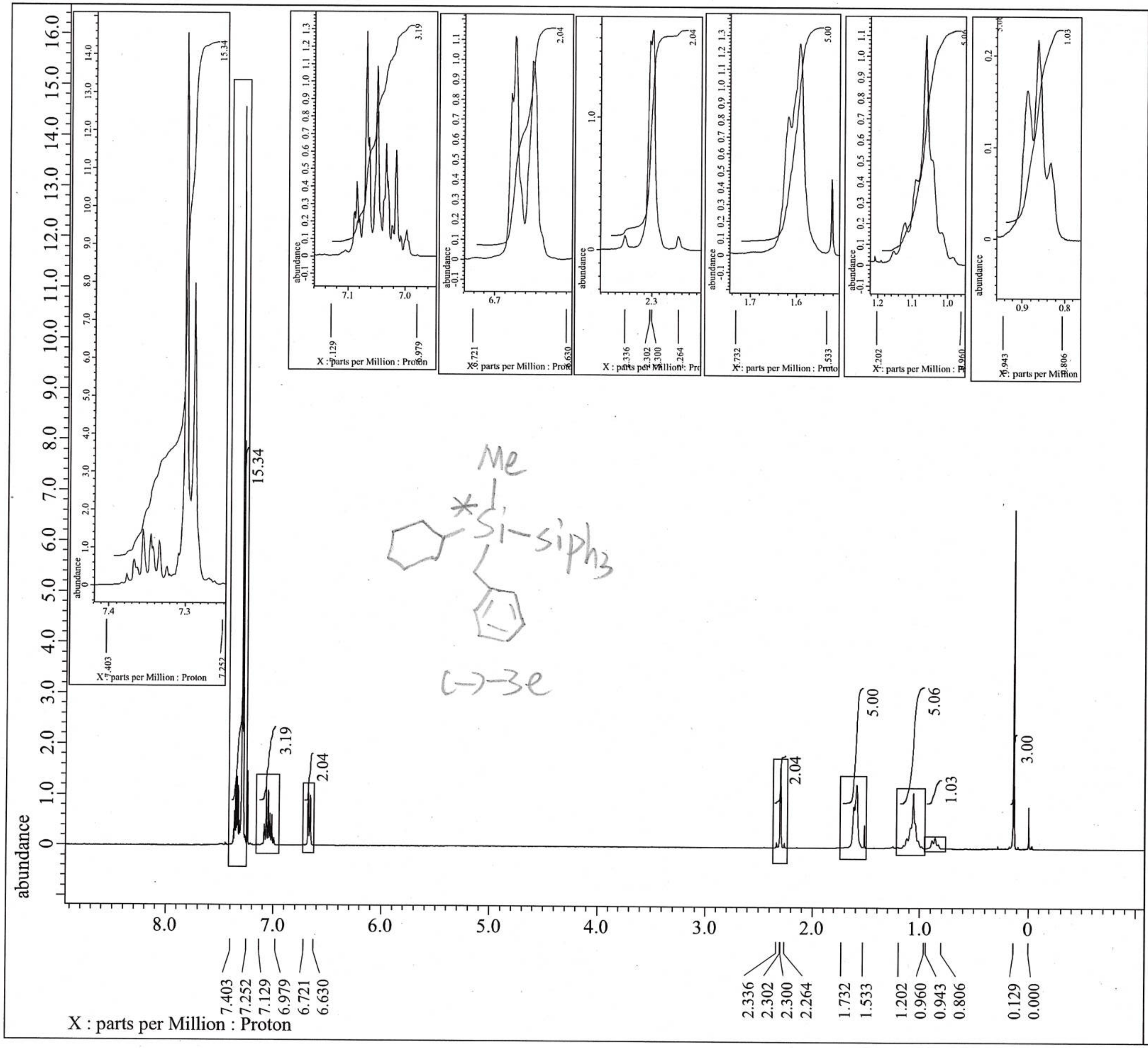

---- PROCESSING PARAMETERS ----
dc_balance(0, FALSE)
sexp(0.2[Hz], 0.0[s])
trapezoid(0[%], 0[%], 80[%], 100[%])
zerofill(1, TRUE)
fft(1, TRUE, TRUE)
machinephase
ppm

数据来源: wxh-108-6_Proton-1-1.jdf

Filename	= wxh-108-6_Proton-1-2.jdf
Author	= element
Experiment	= proton.jxp
Sample_Id	= wxh-108-6
Solvent	= CHLOROFORM-D
Actual_Start_Time	= 16-DEC-2021 11:02:58
Revision_Time	= 28-JUN-2023 15:30:31
Comment	= single_pulse
Data_Format	= 1D COMPLEX
Dim_Size	= 13107
X_Domain	= Proton
Dim_Title	= Proton
Dim_Units	= [ppm]
Dimensions	= X
Spectrometer	= DELTA2_NMR
Field_Strength	= 9.4073814[T] (400[MHz])
X_Acq_Duration	= 2.18103808[s]
X_Domain	= 1H
X_Freq	= 400.53219825[MHz]
X_Offset	= 5[ppm]
X_Points	= 16384
X_Prescans	= 1
X_Resolution	= 0.45849727[Hz]
X_Sweep	= 7.51201923[kHz]
X_Sweep_Clipped	= 6.00961538[kHz]
Irr_Domain	= Proton
Irr_Freq	= 400.53219825[MHz]
Irr_Offset	= 5[ppm]
Tri_Domain	= Proton
Tri_Freq	= 400.53219825[MHz]
Tri_Offset	= 5[ppm]
Clipped	= FALSE
Scans	= 8
Total_Scans	= 8
Relaxation_Delay	= 5[s]
Recvr_Gain	= 34
Temp_Get	= 18.5[dC]
X_90_Width	= 6[us]
X_Acq_Time	= 2.18103808[s]
X_Angle	= 45[deg]
X_Atn	= 0.8[dB]
X_Pulse	= 3[us]
Irr_Mode	= Off
Tri_Mode	= Off
Dante_Presat	= FALSE
Initial_Wait	= 1[s]
Repetition_Time	= 7.18103808[s]

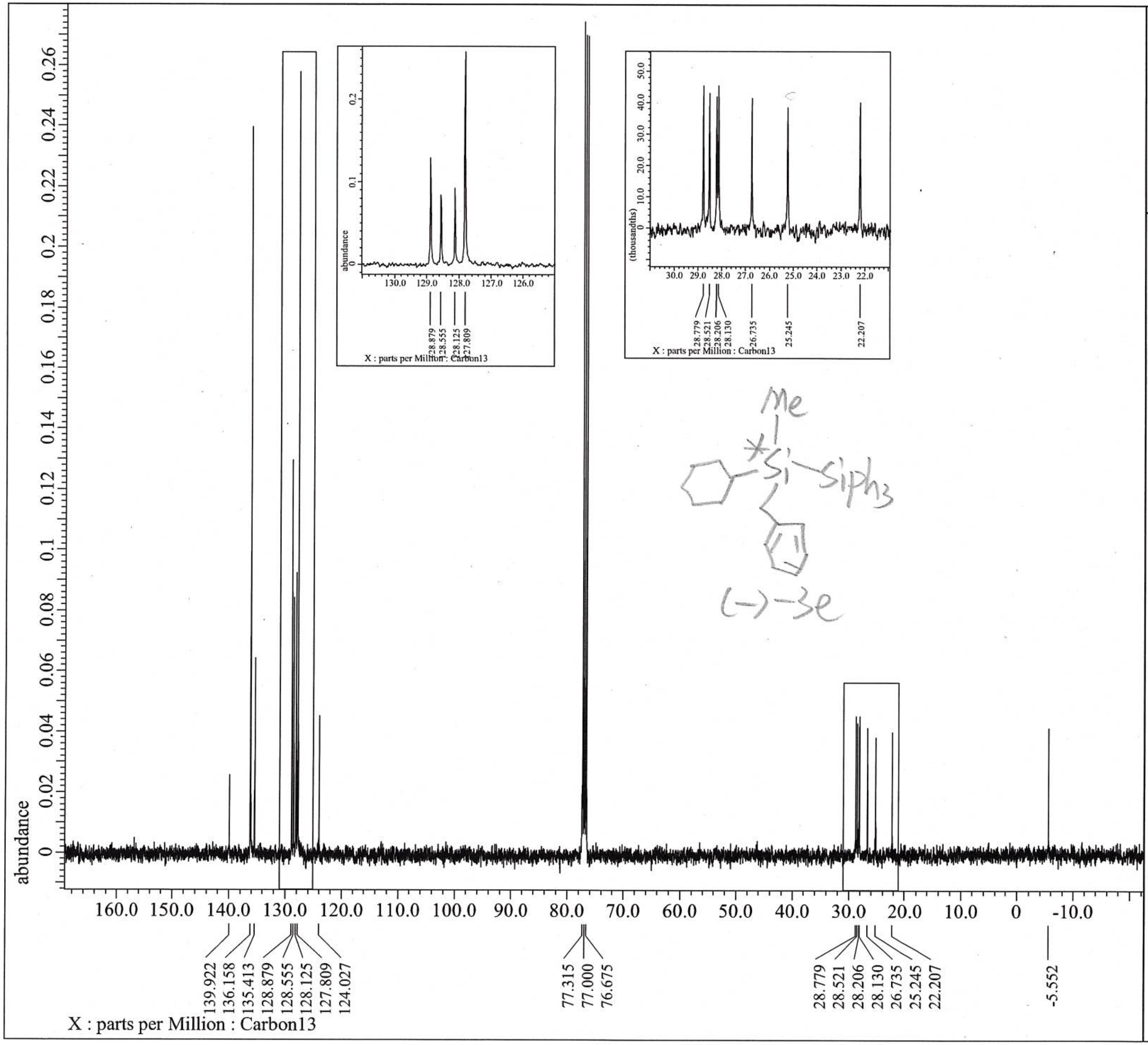

---- PROCESSING PARAMETERS ----
dc_balance(0, FALSE)
sexp(2.0[Hz], 0.0[s])
trapezoid(0[%], 0[%], 80[%], 100[%])
zerofill(1, TRUE)
fft(1, TRUE, TRUE)
machinephase
ppm

数据来源: wxh-108-6_Carbon-1-1.jdf

Filename	= wxh-108-6_Carbon-1-2.jdf
Author	= element
Experiment	= carbon.jxp
Sample_Id	= wxh-108-6
Solvent	= CHLOROFORM-D
Actual_Start_Time	= 16-DEC-2021 13:43:16
Revision_Time	= 5-JAN-2022 16:24:44
Comment	= single pulse decoupled ga
Data_Format	= 1D COMPLEX
Dim_Size	= 26214
X_Domain	= Carbon
Dim_Title	= Carbon13
Dim_Units	= [ppm]
Dimensions	= X
Site	= JNM-ECS400
Spectrometer	= DELTA2_NMR
Field_Strength	= 9.37221[T] (400[MHz])
X_Acq_Duration	= 1.04333312[s]
X_Domain	= 13C
X_Freq	= 100.33735165[MHz]
X_Offset	= 100.0[ppm]
X_Points	= 32768
X_Prescans	= 4
X_Resolution	= 0.95846665[Hz]
X_Sweep	= 31.40703518[kHz]
X_Sweep_Clipped	= 25.12562814[kHz]
Irr_Domain	= Proton
Irr_Freq	= 399.03472754[MHz]
Irr_Offset	= 5.0[ppm]
Clipped	= FALSE
Scans	= 256
Total_Scans	= 256
Relaxation_Delay	= 2[s]
Recvr_Gain	= 50
Temp_Get	= 20.5[dC]
X_90_Width	= 10.9[us]
X_Acq_Time	= 1.04333312[s]
X_Angle	= 30[deg]
X_Atn	= 5.4[dB]
X_Pulse	= 3.63333333[us]
Irr_Atn_Dec	= 25.823[dB]
Irr_Atn_Noe	= 25.823[dB]
Irr_Noise	= WALTZ
Irr_Pwidth	= 0.115[ms]
Decoupling	= TRUE
Initial_Wait	= 1[s]
Noe	= TRUE
Noe_Time	= 2[s]
Repetition_Time	= 3.04333312[s]

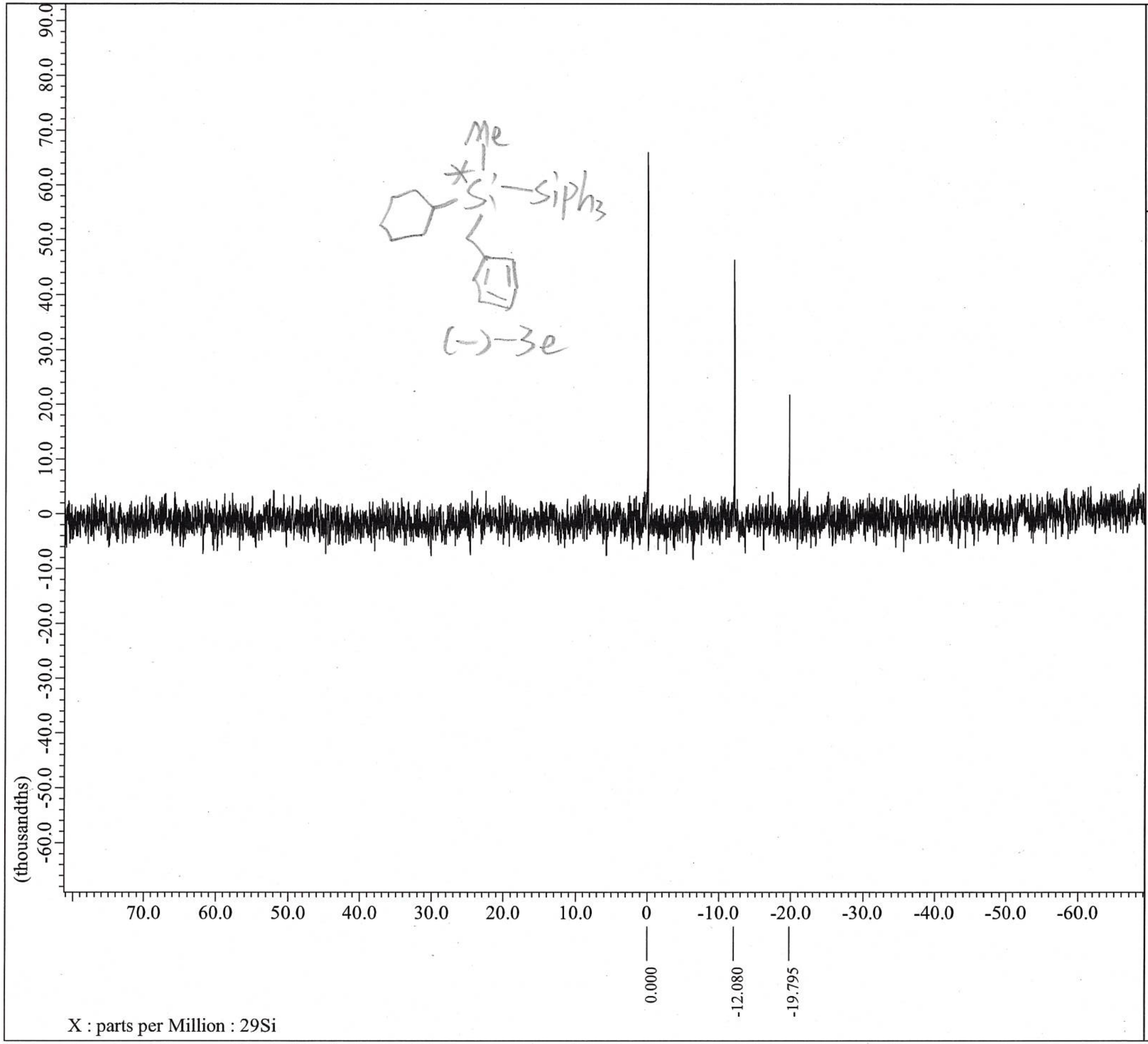


```
---- PROCESSING PARAMETERS ----
dc_balance( 0, FALSE )
sexp( 2.0[Hz], 0.0[s] )
trapezoid3( 0[%], 80[%], 100[%] )
zerofill( 1, TRUE )
fft( 1, TRUE, TRUE )
machinephase
ppm
phase( 58.31165, 0, 50[%] )

数据来源: wxh-108-6-Si-1.jdf
```

```
Filename           = wxh-108-6-Si-2.jdf
Author             = element
Experiment         = single_pulse_dec
Sample_Id          = S#667405
Solvent            = CHLOROFORM-D
Actual_Start_Time  = 17-DEC-2021 01:15:57
Revision_Time      =  5-JAN-2022 17:02:20

Comment            = single pulse decoupled ga
Data_Format        = 1D COMPLEX
Dim_Size           = 26214
X_Domain           = 29Si
Dim_Title          = 29Si
Dim_Units          = [ppm]
Dimensions         = X
Site               = ECS 400
Spectrometer       = JNM-ECS400

Field_Strength     = 9.20197068[T] (390[MHz])
X_Acq_Duration     = 1.34217728[s]
X_Domain           = 29Si
X_Freq             = 77.83692472[MHz]
X_Offset           = 0[ppm]
X_Points           = 32768
X_Prescans         = 4
X_Resolution       = 0.74505806[Hz]
X_Sweep            = 24.4140625[kHz]
Irr_Domain         = 1H
Irr_Freq           = 391.78655441[MHz]
Irr_Offset         = 5[ppm]
Clipped            = FALSE
Scans              = 700
Total_Scans        = 700

Relaxation_Delay   = 7[s]
Recvr_Gain         = 60
Temp_Get           = 20.3[dC]
X_90_Width         = 10[us]
X_Acq_Time         = 1.34217728[s]
X_Angle            = 30[deg]
X_Atn              = 4.9[dB]
X_Pulse            = 3.33333333[us]
Irr_Atn_Dec        = 22.45[dB]
Irr_Noise          = WALTZ
Decoupling         = TRUE
Initial_Wait       = 1[s]
Noe                = FALSE
Repetition_Time    = 8.34217728[s]
```

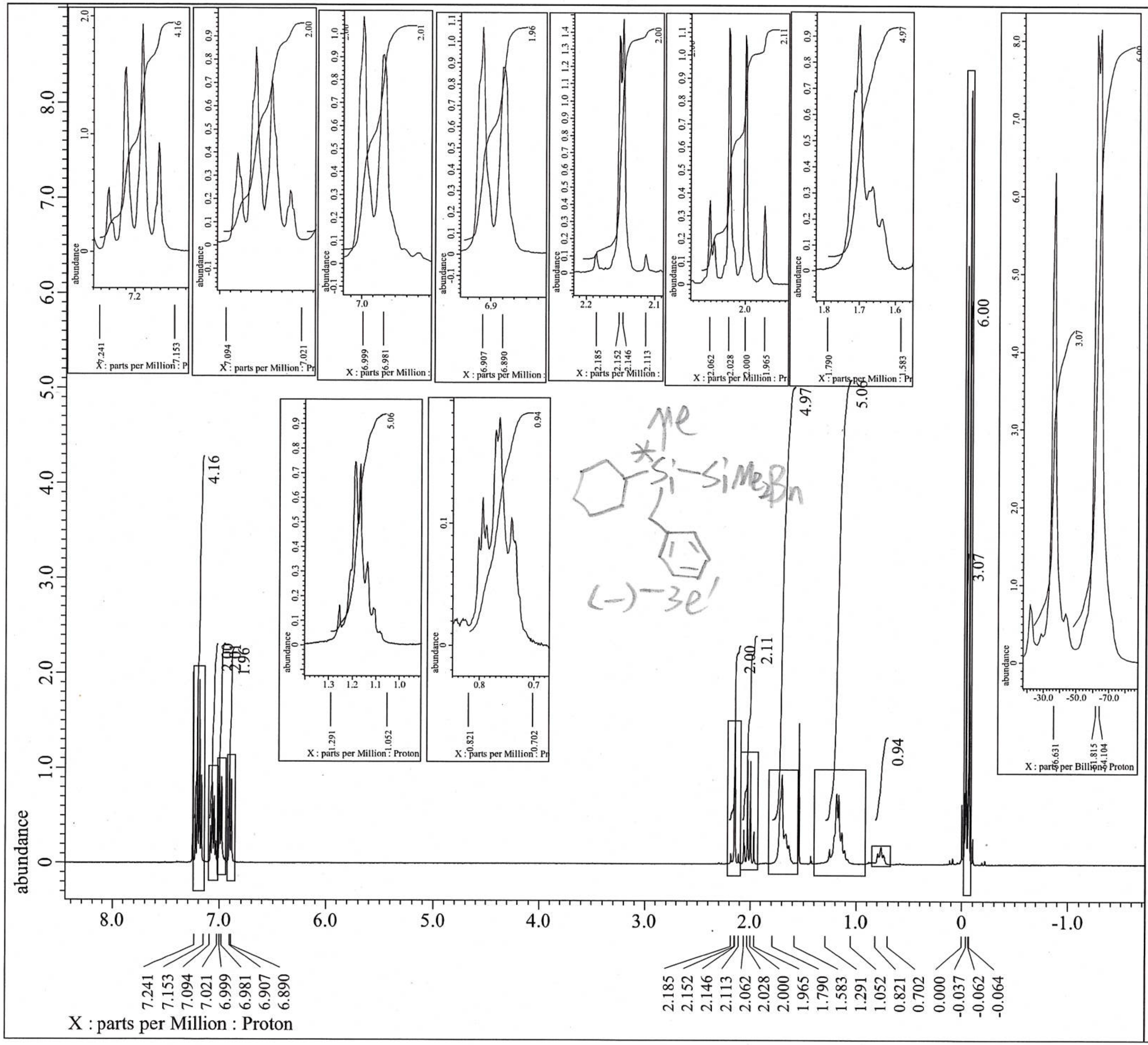


```
---- PROCESSING PARAMETERS ----
dc_balance( 0, FALSE )
sexp( 0.2[Hz], 0.0[s] )
trapezoid( 0[%], 0[%], 80[%], 100[%] )
zerofill( 1, TRUE )
fft( 1, TRUE, TRUE )
machinephase
ppm

数据来源: wxh-146-3_Proton-1-1.jdf

Filename            = wxh-146-3_Proton-1-2.jdf
Author              = element
Experiment          = proton.jxp
Sample_Id           = wxh-146-3
Solvent             = CHLOROFORM-D
Actual_Start_Time   = 28-APR-2022 10:51:31
Revision_Time       = 28-JUN-2023 15:51:34

Comment             = single_pulse
Data_Format         = 1D COMPLEX
Dim_Size            = 13107
X_Domain            = Proton
Dim_Title           = Proton
Dim_Units           = [ppm]
Dimensions          = X
Spectrometer        = DELTA2_NMR

Field_Strength      = 9.4073814[T] (400[MHz])
X_Acq_Duration      = 2.18103808[s]
X_Domain            = 1H
X_Freq              = 400.53219825[MHz]
X_Offset            = 5[ppm]
X_Points            = 16384
X_Prescans          = 1
X_Resolution        = 0.45849727[Hz]
X_Sweep             = 7.51201923[kHz]
X_Sweep_Clipped     = 6.00961538[kHz]
Irr_Domain          = Proton
Irr_Freq            = 400.53219825[MHz]
Irr_Offset          = 5[ppm]
Tri_Domain          = Proton
Tri_Freq            = 400.53219825[MHz]
Tri_Offset          = 5[ppm]
Clipped             = FALSE
Scans               = 8
Total_Scans         = 8

Relaxation_Delay    = 5[s]
Recvr_Gain          = 48
Temp_Get            = 18.5[dC]
X_90_Width          = 6[us]
X_Acq_Time          = 2.18103808[s]
X_Angle             = 45[deg]
X_Atn               = 0.8[dB]
X_Pulse             = 3[us]
Irr_Mode            = Off
Tri_Mode            = Off
Dante_Presat        = FALSE
Initial_Wait        = 1[s]
Repetition_Time     = 7.18103808[s]
```

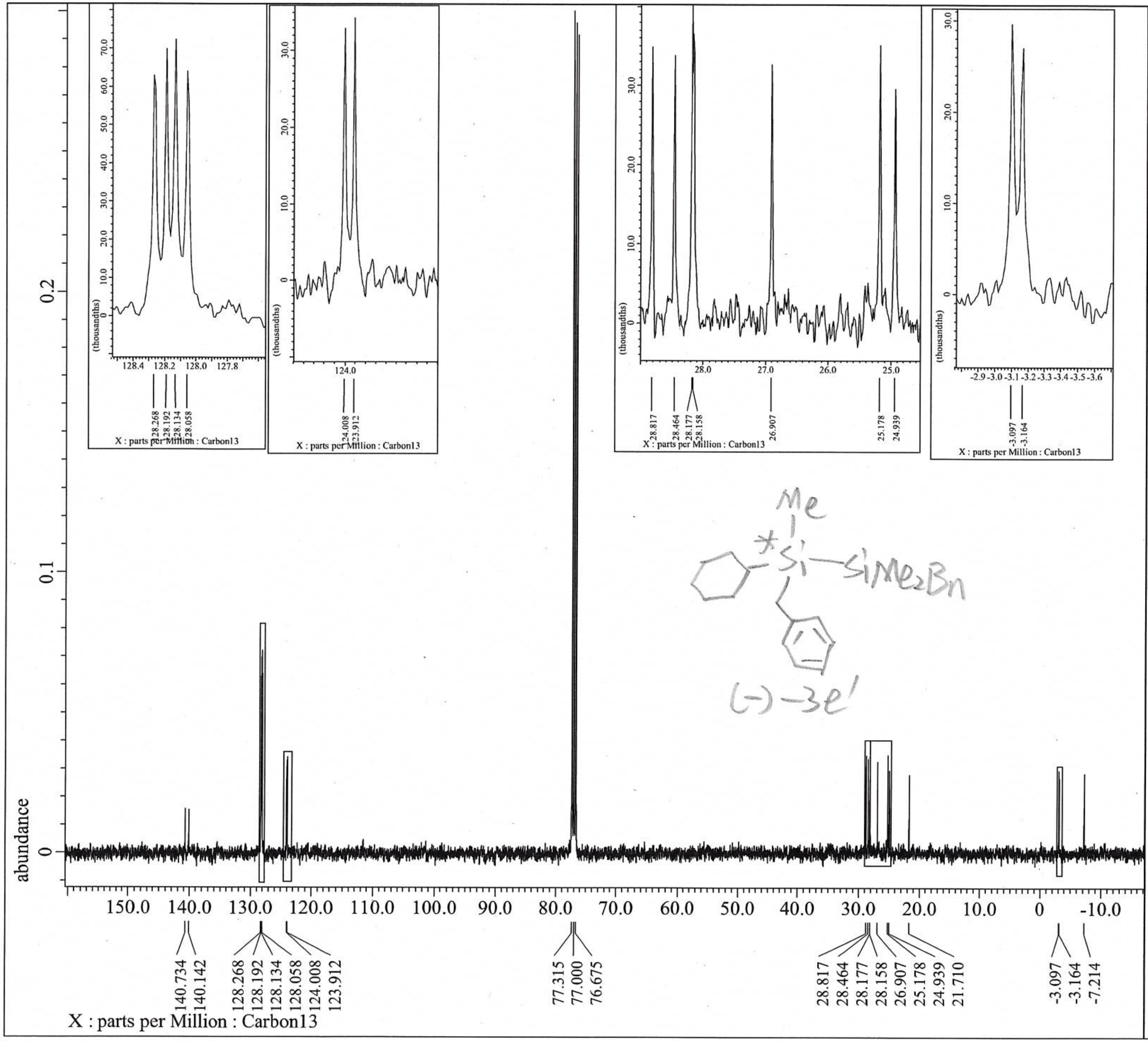


```
---- PROCESSING PARAMETERS ----
dc_balance( 0, FALSE )
sexp( 2.0[Hz], 0.0[s] )
trapezoid( 0[%], 0[%], 80[%], 100[%] )
zerofill( 1, TRUE )
fft( 1, TRUE, TRUE )
machinephase
ppm

数据来源: wxh-146-3_Carbon-1-1.jdf
```

```
Filename            = wxh-146-3_Carbon-1-2.jdf
Author              = element
Experiment          = carbon.jxp
Sample_Id           = wxh-146-3
Solvent             = CHLOROFORM-D
Actual_Start_Time   = 28-APR-2022 13:11:03
Revision_Time       =  7-MAY-2022 14:19:42

Comment             = single pulse decoupled ga
Data_Format         = 1D COMPLEX
Dim_Size            = 26214
X_Domain            = Carbon
Dim_Title           = Carbon13
Dim_Units           = [ppm]
Dimensions          = X
Site                = JNM-ECS400
Spectrometer        = DELTA2_NMR

Field_Strength      = 9.37221[T] (400[MHz])
X_Acq_Duration      = 1.04333312[s]
X_Domain            = 13C
X_Freq              = 100.33735165[MHz]
X_Offset            = 100.0[ppm]
X_Points            = 32768
X_Prescans          = 4
X_Resolution        = 0.95846665[Hz]
X_Sweep             = 31.40703518[kHz]
X_Sweep_Clipped     = 25.12562814[kHz]
Irr_Domain          = Proton
Irr_Freq            = 399.03472754[MHz]
Irr_Offset          = 5.0[ppm]
Clipped             = FALSE
Scans               = 256
Total_Scans         = 256

Relaxation_Delay    = 2[s]
Recvr_Gain          = 50
Temp_Get            = 17.5[dC]
X_90_Width          = 10.9[us]
X_Acq_Time          = 1.04333312[s]
X_Angle             = 30[deg]
X_Atn               = 5.4[dB]
X_Pulse             = 3.63333333[us]
Irr_Atn_Dec         = 25.823[dB]
Irr_Atn_Noe         = 25.823[dB]
Irr_Noise           = WALTZ
Irr_Pwidth          = 0.115[ms]
Decoupling          = TRUE
Initial_Wait        = 1[s]
Noe                 = TRUE
Noe_Time            = 2[s]
Repetition_Time     = 3.04333312[s]
```

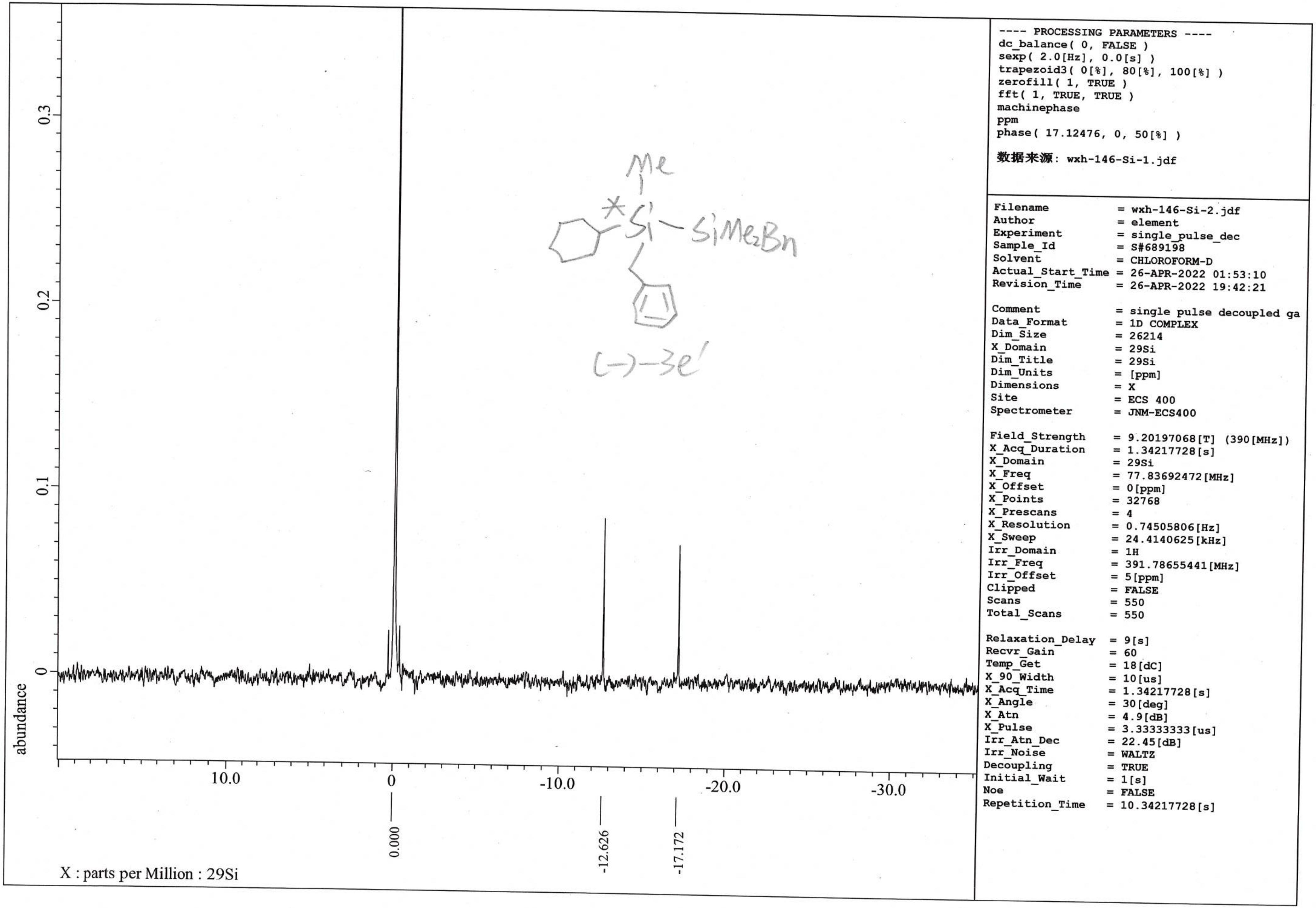

---- PROCESSING PARAMETERS ----
dc_balance(0, FALSE)
sexp(2.0[Hz], 0.0[s])
trapezoid3(0[%], 80[%], 100[%])
zerofill(1, TRUE)
fft(1, TRUE, TRUE)
machinephase
ppm
phase(17.12476, 0, 50[%])
数据来源: wxh-146-Si-1.jdf
Filename = wxh-146-Si-2.jdf
Author = element
Experiment = single_pulse_dec
Sample_Id = S#689198
Solvent = CHLOROFORM-D
Actual_Start_Time = 26-APR-2022 01:53:10
Revision_Time = 26-APR-2022 19:42:21
Comment = single pulse decoupled ga
Data_Format = 1D COMPLEX
Dim_Size = 26214
X_Domain = 29Si
Dim_Title = 29Si
Dim_Units = [ppm]
Dimensions = X
Site = ECS 400
Spectrometer = JNM-ECS400
Field_Strength = 9.20197068[T] (390[MHz])
X_Acq_Duration = 1.34217728[s]
X_Domain = 29Si
X_Freq = 77.83692472[MHz]
X_Offset = 0[ppm]
X_Points = 32768
X_Prescans = 4
X_Resolution = 0.74505806[Hz]
X_Sweep = 24.4140625[kHz]
Irr_Domain = 1H
Irr_Freq = 391.78655441[MHz]
Irr_Offset = 5[ppm]
Clipped = FALSE
Scans = 550
Total_Scans = 550
Relaxation_Delay = 9[s]
Recvr_Gain = 60
Temp_Get = 18[dC]
X_90_Width = 10[us]
X_Acq_Time = 1.34217728[s]
X_Angle = 30[deg]
X_Atn = 4.9[dB]
X_Pulse = 3.33333333[us]
Irr_Atn_Dec = 22.45[dB]
Irr_Noise = WALTZ
Decoupling = TRUE
Initial_Wait = 1[s]
Noe = FALSE
Repetition_Time = 10.34217728[s]
Me
Si
SiMe2Bn
(-)-3e'
0.3
0.2
0.1
0
abundance
10.0
0
-10.0
-20.0
-30.0
0.000
-12.626
-17.172
X : parts per Million : 29Si

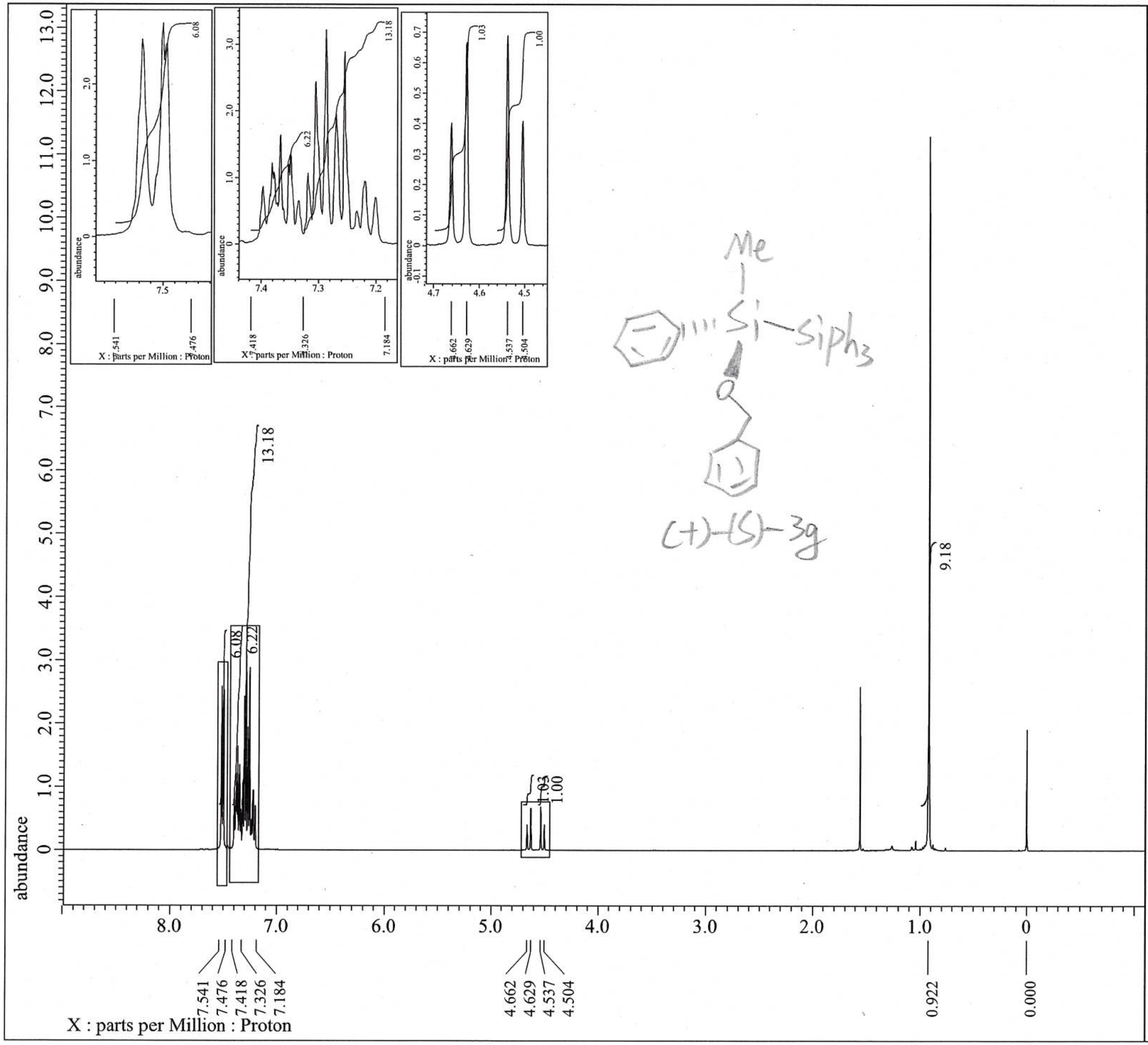

---- PROCESSING PARAMETERS ----
dc_balance(0, FALSE)
sexp(0.2[Hz], 0.0[s])
trapezoid(0[%], 0[%], 80[%], 100[%])
zerofill(1, TRUE)
fft(1, TRUE, TRUE)
machinephase
ppm

数据来源: wxh-335-1_Proton-1-1.jdf

Filename	= wxh-335-1_Proton-1-2.jdf
Author	= element
Experiment	= proton.jxp
Sample_Id	= wxh-335-1
Solvent	= CHLOROFORM-D
Actual_Start_Time	= 28-OCT-2022 17:32:34
Revision_Time	= 28-JUN-2023 16:05:08
Comment	= single_pulse
Data_Format	= 1D COMPLEX
Dim_Size	= 13107
X_Domain	= Proton
Dim_Title	= Proton
Dim_Units	= [ppm]
Dimensions	= X
Site	= JNM-ECS400
Spectrometer	= DELTA2_NMR
Field_Strength	= 9.37221[T] (400[MHz])
X_Acq_Duration	= 2.1889024[s]
X_Domain	= 1H
X_Freq	= 399.03472754[MHz]
X_Offset	= 5.0[ppm]
X_Points	= 16384
X_Prescans	= 1
X_Resolution	= 0.45684997[Hz]
X_Sweep	= 7.48502994[kHz]
X_Sweep_Clipped	= 5.98802395[kHz]
Irr_Domain	= Proton
Irr_Freq	= 399.03472754[MHz]
Irr_Offset	= 5.0[ppm]
Tri_Domain	= Proton
Tri_Freq	= 399.03472754[MHz]
Tri_Offset	= 5.0[ppm]
Clipped	= FALSE
Scans	= 8
Total_Scans	= 8
Relaxation_Delay	= 5[s]
Recvr_Gain	= 40
Temp_Get	= 16.8[dC]
X_90_Width	= 6.6[us]
X_Acq_Time	= 2.1889024[s]
X_Angle	= 45[deg]
X_Atn	= 1[dB]
X_Pulse	= 3.3[us]
Irr_Mode	= Off
Tri_Mode	= Off
Dante_Presat	= FALSE
Initial_Wait	= 1[s]
Repetition_Time	= 7.1889024[s]

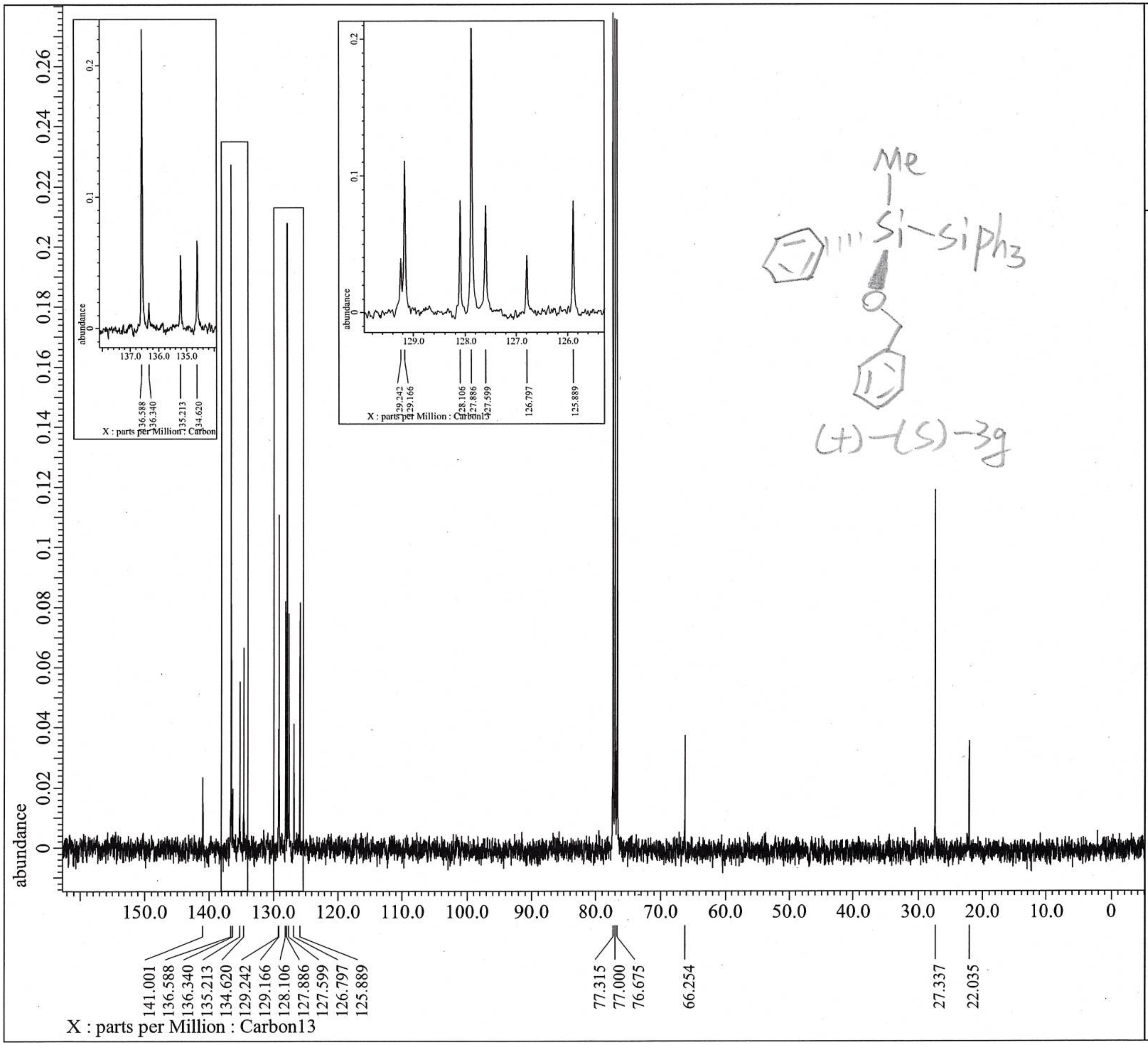


```
---- PROCESSING PARAMETERS ----
dc_balance( 0, FALSE )
sexp( 2.0[Hz], 0.0[s] )
trapezoid( 0[%], 0[%], 80[%], 100[%] )
zerofill( 1, TRUE )
fft( 1, TRUE, TRUE )
machinephase
ppm

数据来源: wxh-335-1_Carbon-1-1.jdf
```

```
Filename            = wxh-335-1_Carbon-1-2.jdf
Author              = element
Experiment          = carbon.jxp
Sample_Id           = wxh-335-1
Solvent             = CHLOROFORM-D
Actual_Start_Time   = 28-OCT-2022 17:59:16
Revision_Time       = 20-JAN-2023 20:41:44

Comment             = single pulse decoupled ga
Data_Format         = 1D COMPLEX
Dim_Size            = 26214
X_Domain            = Carbon
Dim_Title           = Carbon13
Dim_Units           = [ppm]
Dimensions          = X
Site                = JNM-ECS400
Spectrometer        = DELTA2_NMR

Field_Strength      = 9.37221[T] (400[MHz])
X_Acq_Duration      = 1.04333312[s]
X_Domain            = 13C
X_Freq              = 100.33735165[MHz]
X_Offset            = 100.0[ppm]
X_Points            = 32768
X_Prescans          = 4
X_Resolution        = 0.95846665[Hz]
X_Sweep             = 31.40703518[kHz]
X_Sweep_Clipped     = 25.12562814[kHz]
Irr_Domain          = Proton
Irr_Freq            = 399.03472754[MHz]
Irr_Offset          = 5.0[ppm]
Clipped             = FALSE
Scans               = 128
Total_Scans         = 128

Relaxation_Delay    = 2[s]
Recvr_Gain          = 50
Temp_Get            = 16.9[dC]
X_90_Width          = 10.9[us]
X_Acq_Time          = 1.04333312[s]
X_Angle             = 30[deg]
X_Atn               = 5.4[dB]
X_Pulse             = 3.63333333[us]
Irr_Atn_Dec         = 25.823[dB]
Irr_Atn_Noe         = 25.823[dB]
Irr_Noise           = WALTZ
Irr_Pwidth          = 0.115[ms]
Decoupling          = TRUE
Initial_Wait        = 1[s]
Noe                 = TRUE
Noe_Time            = 2[s]
Repetition_Time     = 3.04333312[s]
```

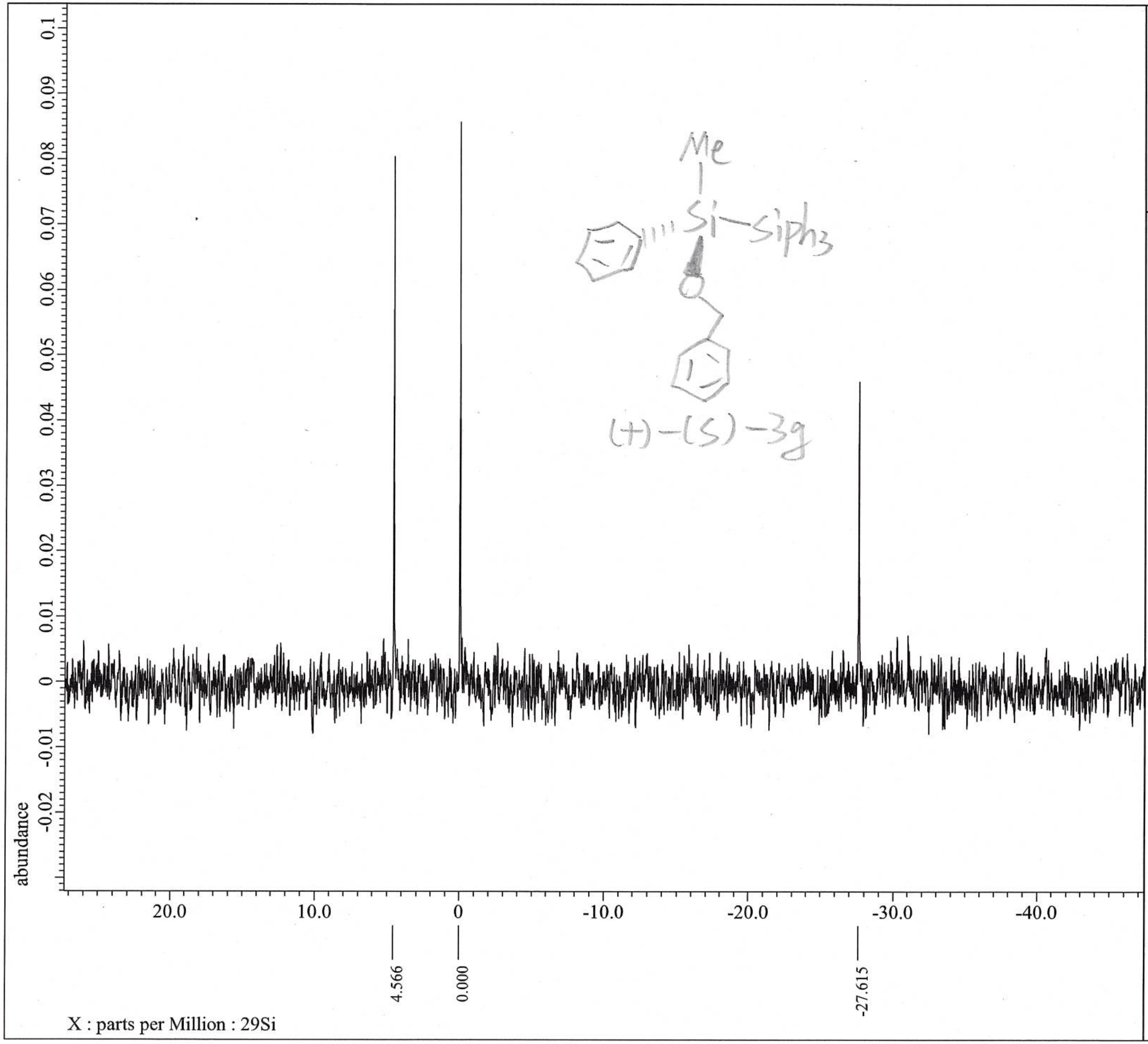


```
---- PROCESSING PARAMETERS ----
dc_balance( 0, FALSE )
sexp( 2.0[Hz], 0.0[s] )
trapezoid3( 0[%], 80[%], 100[%] )
zerofill( 1, TRUE )
fft( 1, TRUE, TRUE )
machinephase
ppm
phase( -66.02998, 0, 50[%] )

数据来源: wxh-335-1-Si-2.jdf

Filename           = wxh-335-1-Si-3.jdf
Author             = element
Experiment         = single_pulse_dec
Sample_Id          = S#654498
Solvent            = CHLOROFORM-D
Actual_Start_Time  = 29-OCT-2022 00:55:19
Revision_Time      = 20-JAN-2023 20:15:55

Comment            = single pulse decoupled ga
Data_Format        = 1D COMPLEX
Dim_Size           = 26214
X_Domain           = 29Si
Dim_Title          = 29Si
Dim_Units          = [ppm]
Dimensions         = X
Site               = ECS 400
Spectrometer       = JNM-ECS400

Field_Strength     = 9.20197068[T] (390[MHz])
X_Acq_Duration     = 1.34217728[s]
X_Domain           = 29Si
X_Freq             = 77.83692472[MHz]
X_Offset           = 0[ppm]
X_Points           = 32768
X_Prescans         = 4
X_Resolution       = 0.74505806[Hz]
X_Sweep            = 24.4140625[kHz]
Irr_Domain         = 1H
Irr_Freq           = 391.78655441[MHz]
Irr_Offset         = 5[ppm]
Clipped            = FALSE
Scans              = 450
Total_Scans        = 450

Relaxation_Delay   = 10[s]
Recvr_Gain         = 60
Temp_Get           = 17.7[dC]
X_90_Width         = 10[us]
X_Acq_Time         = 1.34217728[s]
X_Angle            = 30[deg]
X_Atn              = 4.9[dB]
X_Pulse            = 3.33333333[us]
Irr_Atn_Dec        = 22.45[dB]
Irr_Noise          = WALTZ
Decoupling         = TRUE
Initial_Wait       = 1[s]
Noe                = FALSE
Repetition_Time    = 11.34217728[s]
```

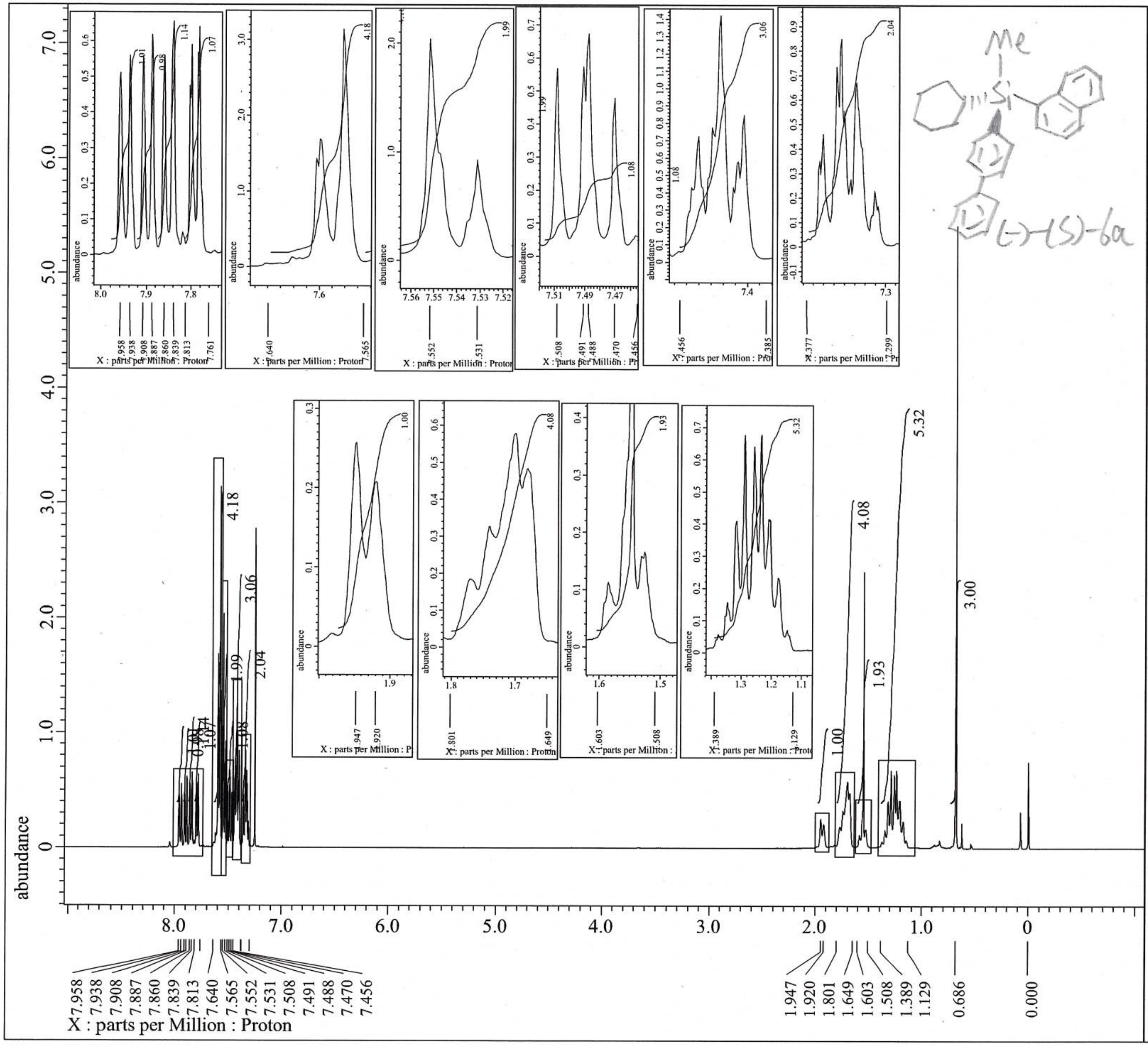

---- PROCESSING PARAMETERS ----
dc_balance(0, FALSE)
sexp(0.2[Hz], 0.0[s])
trapezoid(0[%], 0[%], 80[%], 100[%])
zerofill(1, TRUE)
fft(1, TRUE, TRUE)
machinephase
ppm

数据来源: wxh-248-2_Proton-1-1.jdf

Filename	=	wxh-248-2_Proton-1-2.jdf
Author	=	element
Experiment	=	proton.jxp
Sample_Id	=	248-2
Solvent	=	CHLOROFORM-D
Actual_Start_Time	=	27-APR-2022 10:11:22
Revision_Time	=	28-JUN-2023 16:28:11
Comment	=	single_pulse
Data_Format	=	1D COMPLEX
Dim_Size	=	13107
X_Domain	=	Proton
Dim_Title	=	Proton
Dim_Units	=	[ppm]
Dimensions	=	X
Site	=	JNM-ECS400
Spectrometer	=	DELTA2_NMR
Field_Strength	=	9.37221[T] (400[MHz])
X_Acq_Duration	=	2.1889024[s]
X_Domain	=	1H
X_Freq	=	399.03472754[MHz]
X_Offset	=	5.0[ppm]
X_Points	=	16384
X_Prescans	=	1
X_Resolution	=	0.45684997[Hz]
X_Sweep	=	7.48502994[kHz]
X_Sweep_Clipped	=	5.98802395[kHz]
Irr_Domain	=	Proton
Irr_Freq	=	399.03472754[MHz]
Irr_Offset	=	5.0[ppm]
Tri_Domain	=	Proton
Tri_Freq	=	399.03472754[MHz]
Tri_Offset	=	5.0[ppm]
Clipped	=	FALSE
Scans	=	8
Total_Scans	=	8
Relaxation_Delay	=	5[s]
Recvr_Gain	=	38
Temp_Get	=	17.5[dC]
X_90_Width	=	6.6[us]
X_Acq_Time	=	2.1889024[s]
X_Angle	=	45[deg]
X_Atn	=	1[dB]
X_Pulse	=	3.3[us]
Irr_Mode	=	Off
Tri_Mode	=	Off
Dante_Presat	=	FALSE
Initial_Wait	=	1[s]
Repetition_Time	=	7.1889024[s]

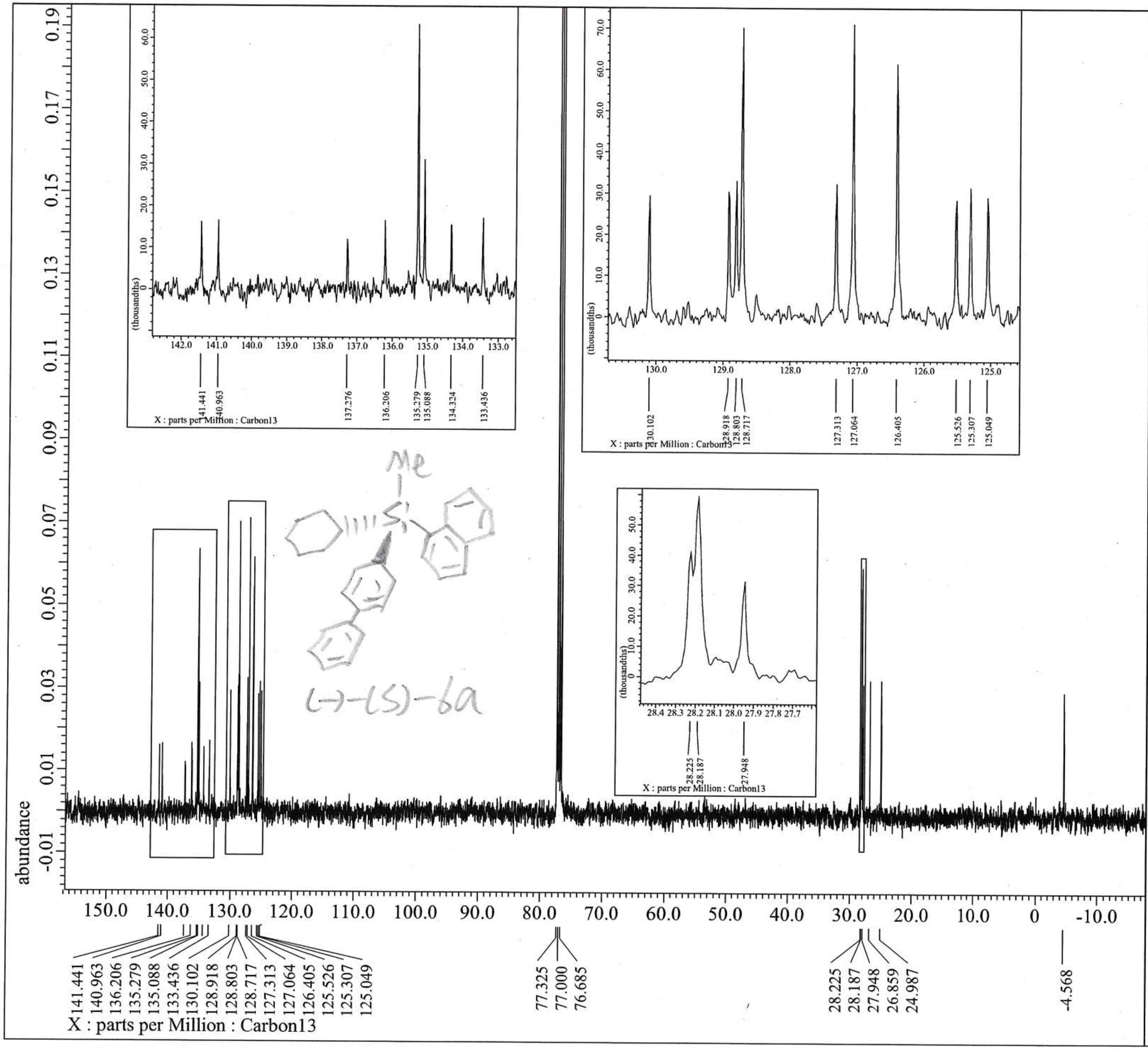


```
---- PROCESSING PARAMETERS ----
dc_balance( 0, FALSE )
sexp( 2.0[Hz], 0.0[s] )
trapezoid( 0[%], 0[%], 80[%], 100[%] )
zerofill( 1, TRUE )
fft( 1, TRUE, TRUE )
machinephase
ppm

数据来源: wxh-248-2_Carbon-1-1.jdf
```

```
Filename            = wxh-248-2_Carbon-1-2.jdf
Author              = element
Experiment          = carbon.jxp
Sample_Id           = wxh-248-2
Solvent             = CHLOROFORM-D
Actual_Start_Time   = 26-APR-2022 10:24:18
Revision_Time       = 26-APR-2022 15:22:51

Comment             = single pulse decoupled ga
Data_Format         = 1D COMPLEX
Dim_Size            = 26214
X_Domain            = Carbon
Dim_Title           = Carbon13
Dim_Units           = [ppm]
Dimensions          = X
Site                = JNM-ECS400
Spectrometer        = DELTA2_NMR

Field_Strength      = 9.37221[T] (400[MHz])
X_Acq_Duration      = 1.04333312[s]
X_Domain            = 13C
X_Freq              = 100.33735165[MHz]
X_Offset            = 100.0[ppm]
X_Points            = 32768
X_Prescans          = 4
X_Resolution        = 0.95846665[Hz]
X_Sweep             = 31.40703518[kHz]
X_Sweep_Clipped     = 25.12562814[kHz]
Irr_Domain          = Proton
Irr_Freq            = 399.03472754[MHz]
Irr_Offset          = 5.0[ppm]
Clipped             = FALSE
Scans               = 256
Total_Scans         = 256

Relaxation_Delay    = 2[s]
Recvr_Gain          = 50
Temp_Get            = 17.1[dC]
X_90_Width          = 10.9[us]
X_Acq_Time          = 1.04333312[s]
X_Angle             = 30[deg]
X_Atn               = 5.4[dB]
X_Pulse             = 3.63333333[us]
Irr_Atn_Dec         = 25.823[dB]
Irr_Atn_Noe         = 25.823[dB]
Irr_Noise           = WALTZ
Irr_Pwidth          = 0.115[ms]
Decoupling          = TRUE
Initial_Wait        = 1[s]
Noe                 = TRUE
Noe_Time            = 2[s]
Repetition_Time     = 3.04333312[s]
```

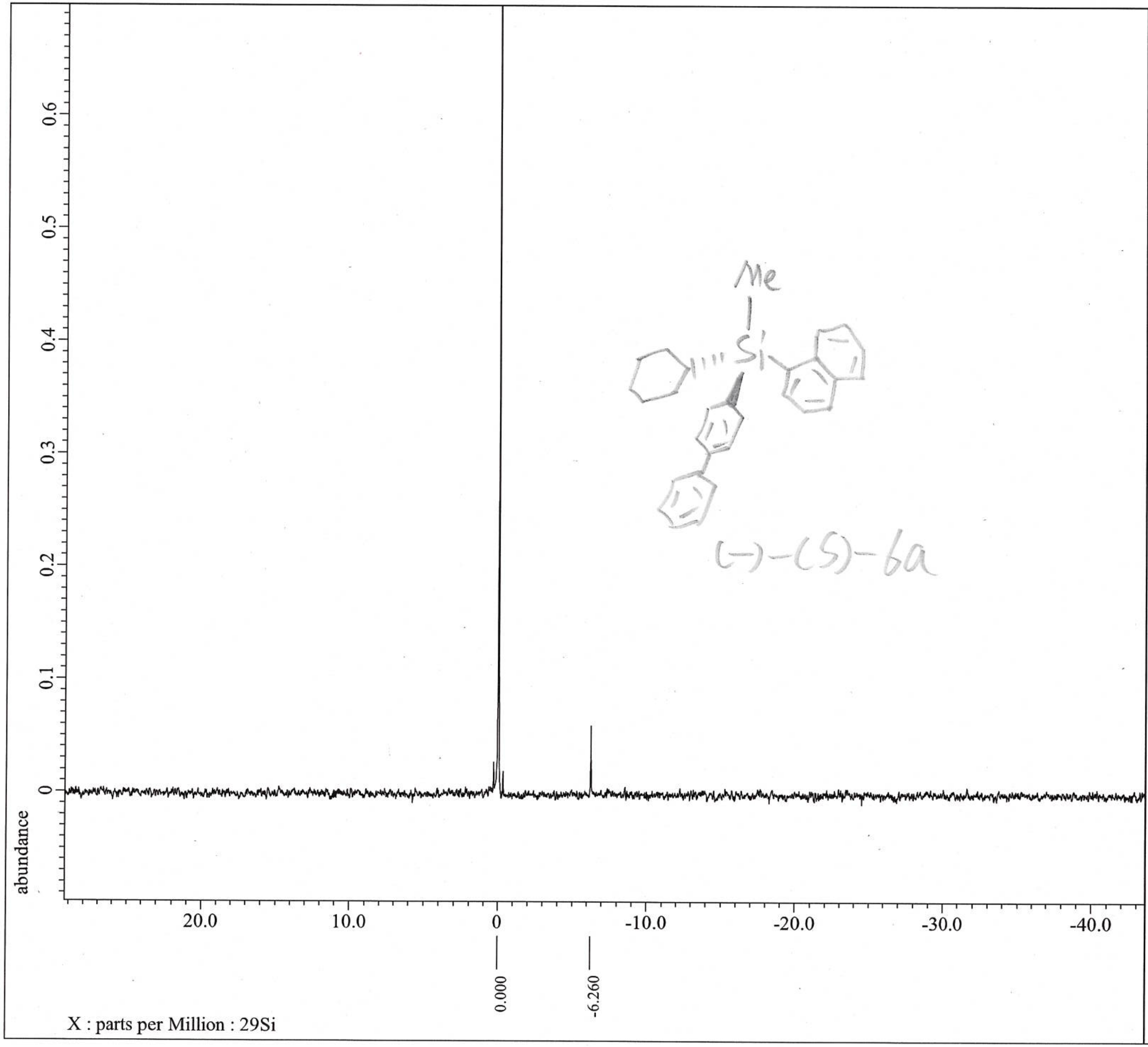


```
---- PROCESSING PARAMETERS ----
dc_balance( 0, FALSE )
sexp( 2.0[Hz], 0.0[s] )
trapezoid3( 0[%], 80[%], 100[%] )
zerofill( 1, TRUE )
fft( 1, TRUE, TRUE )
machinephase
ppm
phase( 27.22923, 0, 50[%] )

数据来源: wxh-248-Si-1.jdf
```

```
Filename             = wxh-248-Si-2.jdf
Author               = element
Experiment           = single_pulse_dec
Sample_Id            = S#363891
Solvent              = CHLOROFORM-D
Actual_Start_Time    = 26-APR-2022 16:51:14
Revision_Time        = 26-APR-2022 15:33:04

Comment              = single pulse decoupled ga
Data_Format          = 1D COMPLEX
Dim_Size             = 26214
X_Domain             = 29Si
Dim_Title            = 29Si
Dim_Units            = [ppm]
Dimensions           = X
Site                 = ECS 400
Spectrometer         = JNM-ECS400

Field_Strength       = 9.20197068[T] (390[MHz])
X_Acq_Duration       = 1.34217728[s]
X_Domain             = 29Si
X_Freq               = 77.83692472[MHz]
X_Offset             = 0[ppm]
X_Points             = 32768
X_Prescans           = 4
X_Resolution         = 0.74505806[Hz]
X_Sweep              = 24.4140625[kHz]
Irr_Domain           = 1H
Irr_Freq             = 391.78655441[MHz]
Irr_Offset           = 5[ppm]
Clipped              = FALSE
Scans                = 600
Total_Scans          = 600

Relaxation_Delay     = 9[s]
Recvr_Gain           = 60
Temp_Get             = 18.2[dC]
X_90_Width           = 10[us]
X_Acq_Time           = 1.34217728[s]
X_Angle              = 30[deg]
X_Atn                = 4.9[dB]
X_Pulse              = 3.33333333[us]
Irr_Atn_Dec          = 22.45[dB]
Irr_Noise            = WALTZ
Decoupling           = TRUE
Initial_Wait         = 1[s]
Noe                  = FALSE
Repetition_Time      = 10.34217728[s]
```

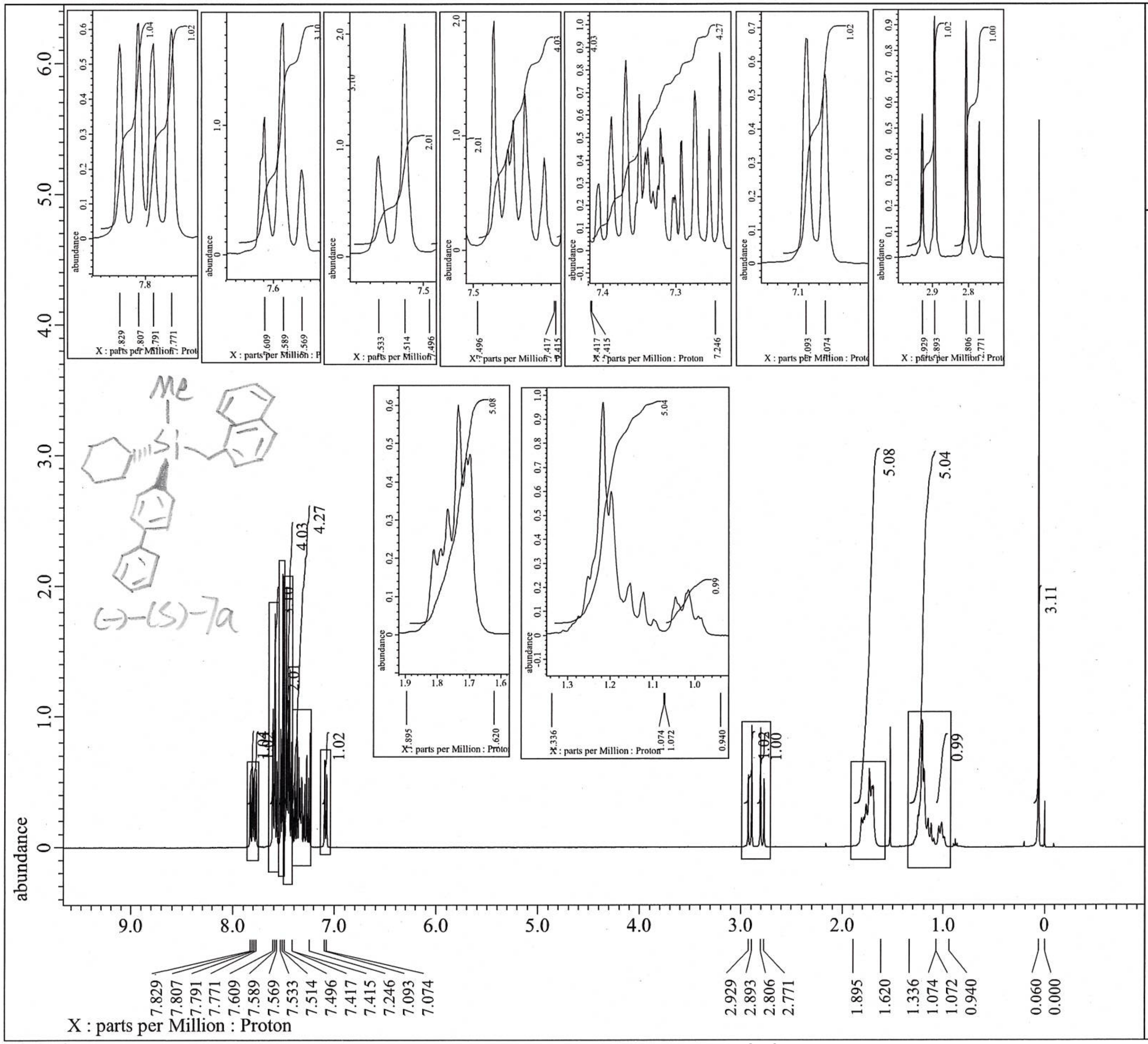

---- PROCESSING PARAMETERS ----
dc_balance(0, FALSE)
sexp(0.2[Hz], 0.0[s])
trapezoid(0[%], 0[%], 80[%], 100[%])
zerofill(1, TRUE)
fft(1, TRUE, TRUE)
machinephase
ppm

数据来源: wxh-227-1_Proton-1-1.jdf

Filename	= wxh-227-1_Proton-1-2.jdf
Author	= element
Experiment	= proton.jxp
Sample_Id	= wxh-237-1
Solvent	= CHLOROFORM-D
Actual_Start_Time	= 18-APR-2022 17:55:01
Revision_Time	= 28-JUN-2023 20:31:21
Comment	= single_pulse
Data_Format	= 1D COMPLEX
Dim_Size	= 13107
X_Domain	= Proton
Dim_Title	= Proton
Dim_Units	= [ppm]
Dimensions	= X
Site	= JNM-ECS400
Spectrometer	= DELTA2_NMR
Field_Strength	= 9.37221[T] (400[MHz])
X_Acq_Duration	= 2.1889024[s]
X_Domain	= 1H
X_Freq	= 399.03472754[MHz]
X_Offset	= 5.0[ppm]
X_Points	= 16384
X_Prescans	= 1
X_Resolution	= 0.45684997[Hz]
X_Sweep	= 7.48502994[kHz]
X_Sweep_Clipped	= 5.98802395[kHz]
Irr_Domain	= Proton
Irr_Freq	= 399.03472754[MHz]
Irr_Offset	= 5.0[ppm]
Tri_Domain	= Proton
Tri_Freq	= 399.03472754[MHz]
Tri_Offset	= 5.0[ppm]
Clipped	= FALSE
Scans	= 8
Total_Scans	= 8
Relaxation_Delay	= 5[s]
Recvr_Gain	= 32
Temp_Get	= 17.7[dC]
X_90_Width	= 6.6[us]
X_Acq_Time	= 2.1889024[s]
X_Angle	= 45[deg]
X_Atn	= 1[dB]
X_Pulse	= 3.3[us]
Irr_Mode	= Off
Tri_Mode	= Off
Dante_Presat	= FALSE
Initial_Wait	= 1[s]
Repetition_Time	= 7.1889024[s]

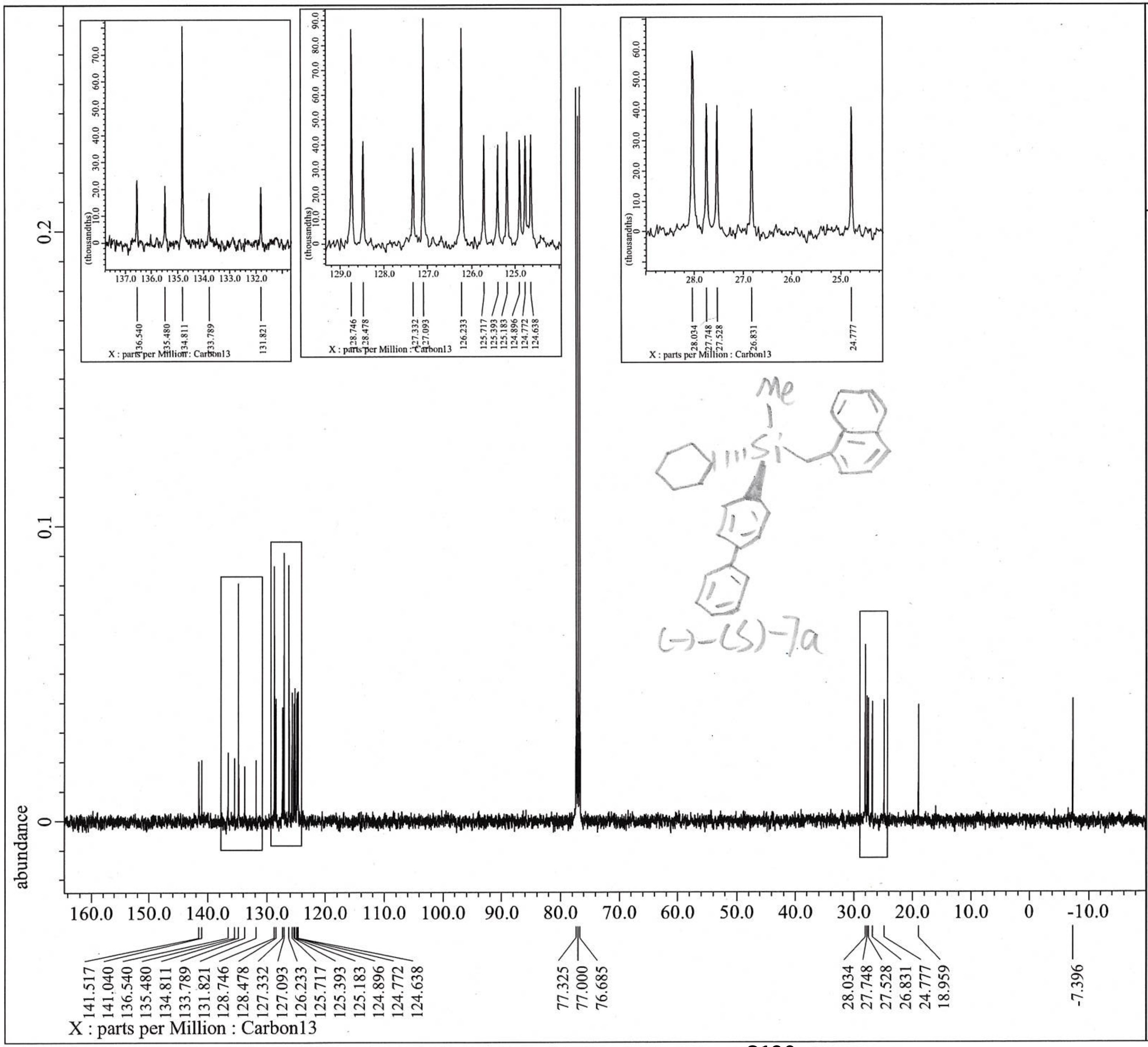

---- PROCESSING PARAMETERS ----
dc_balance(0, FALSE)
sexp(2.0[Hz], 0.0[s])
trapezoid(0[%], 0[%], 80[%], 100[%])
zerofill(1, TRUE)
fft(1, TRUE, TRUE)
machinephase
ppm

数据来源: wxh-227-1_Carbon-1-1.jdf

Filename	= wxh-227-1_Carbon-1-2.jdf
Author	= element
Experiment	= carbon.jxp
Sample_Id	= wxh-227-1
Solvent	= CHLOROFORM-D
Actual_Start_Time	= 18-APR-2022 18:02:21
Revision_Time	= 7-MAY-2022 17:28:58
Comment	= single pulse decoupled ga
Data_Format	= 1D COMPLEX
Dim_Size	= 26214
X_Domain	= Carbon
Dim_Title	= Carbon13
Dim_Units	= [ppm]
Dimensions	= X
Site	= JNM-ECS400
Spectrometer	= DELTA2_NMR
Field_Strength	= 9.37221[T] (400[MHz])
X_Acq_Duration	= 1.04333312[s]
X_Domain	= 13C
X_Freq	= 100.33735165[MHz]
X_Offset	= 100.0[ppm]
X_Points	= 32768
X_Prescans	= 4
X_Resolution	= 0.95846665[Hz]
X_Sweep	= 31.40703518[kHz]
X_Sweep_Clipped	= 25.12562814[kHz]
Irr_Domain	= Proton
Irr_Freq	= 399.03472754[MHz]
Irr_Offset	= 5.0[ppm]
Clipped	= FALSE
Scans	= 256
Total_Scans	= 256
Relaxation_Delay	= 2[s]
Recvr_Gain	= 50
Temp_Get	= 17.9[dC]
X_90_Width	= 10.9[us]
X_Acq_Time	= 1.04333312[s]
X_Angle	= 30[deg]
X_Atn	= 5.4[dB]
X_Pulse	= 3.63333333[us]
Irr_Atn_Dec	= 25.823[dB]
Irr_Atn_Noe	= 25.823[dB]
Irr_Noise	= WALTZ
Irr_Pwidth	= 0.115[ms]
Decoupling	= TRUE
Initial_Wait	= 1[s]
Noe	= TRUE
Noe_Time	= 2[s]
Repetition_Time	= 3.04333312[s]

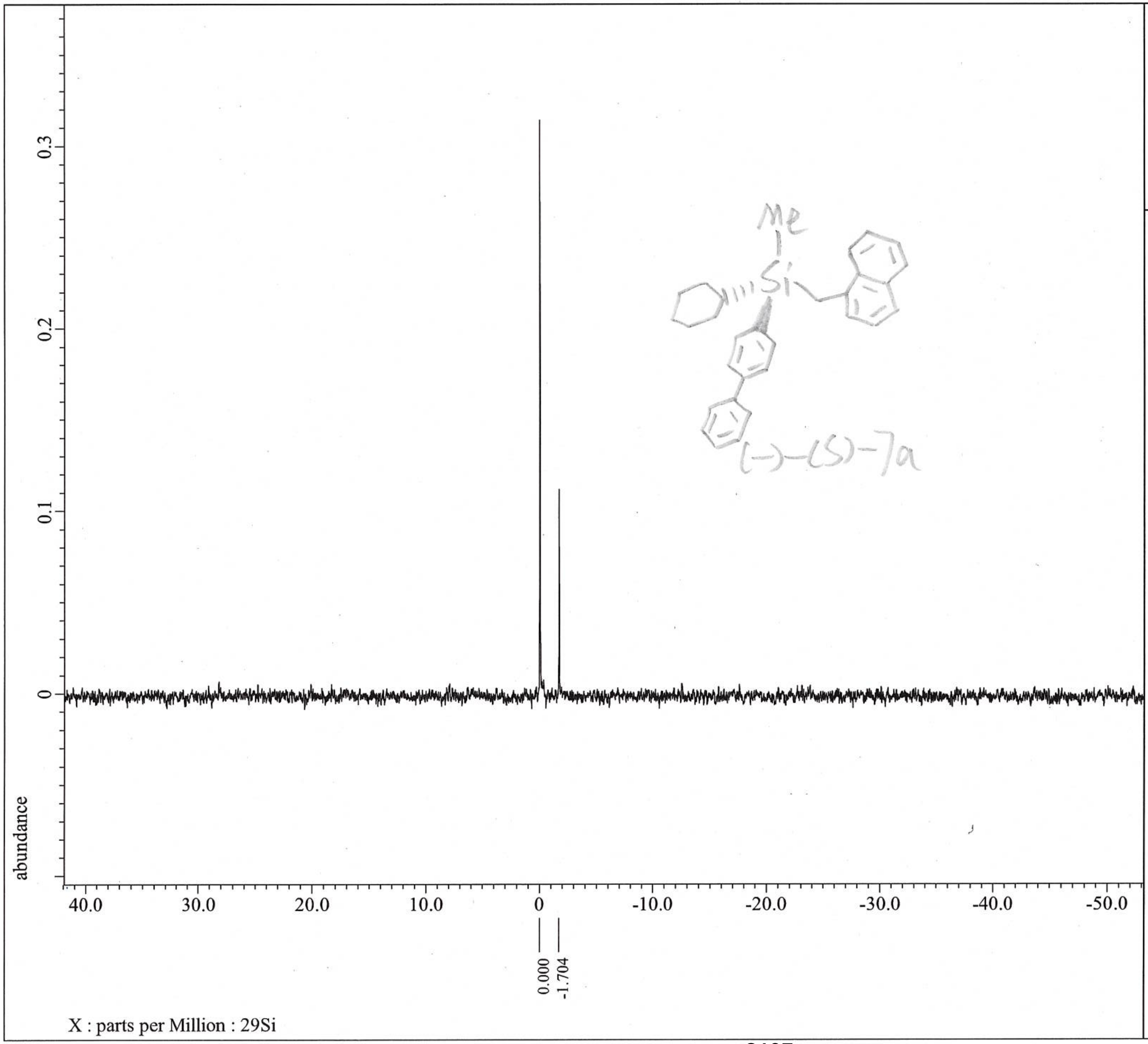


```
---- PROCESSING PARAMETERS ----
dc_balance( 0, FALSE )
sexp( 2.0[Hz], 0.0[s] )
trapezoid3( 0[%], 80[%], 100[%] )
zerofill( 1, TRUE )
fft( 1, TRUE, TRUE )
machinephase
ppm
phase( 64.29914, 0, 50[%] )

数据来源: wxh-227-Si-1.jdf
```

```
Filename           = wxh-227-Si-2.jdf
Author             = element
Experiment         = single_pulse_dec
Sample_Id          = S#640355
Solvent            = CHLOROFORM-D
Actual_Start_Time  = 19-APR-2022 00:34:03
Revision_Time      =  7-MAY-2022 17:54:05

Comment            = single pulse decoupled ga
Data_Format        = 1D COMPLEX
Dim_Size           = 26214
X_Domain           = 29Si
Dim_Title          = 29Si
Dim_Units          = [ppm]
Dimensions         = X
Site               = ECS 400
Spectrometer       = JNM-ECS400

Field_Strength     = 9.20197068[T] (390[MHz])
X_Acq_Duration     = 1.34217728[s]
X_Domain           = 29Si
X_Freq             = 77.83692472[MHz]
X_Offset           = 0[ppm]
X_Points           = 32768
X_Prescans         = 4
X_Resolution       = 0.74505806[Hz]
X_Sweep            = 24.4140625[kHz]
Irr_Domain         = 1H
Irr_Freq           = 391.78655441[MHz]
Irr_Offset         = 5[ppm]
Clipped            = FALSE
Scans              = 600
Total_Scans        = 600

Relaxation_Delay   = 9[s]
Recvr_Gain         = 60
Temp_Get           = 19.7[dC]
X_90_Width         = 10[us]
X_Acq_Time         = 1.34217728[s]
X_Angle            = 30[deg]
X_Atn              = 4.9[dB]
X_Pulse            = 3.33333333[us]
Irr_Atn_Dec        = 22.45[dB]
Irr_Noise          = WALTZ
Decoupling         = TRUE
Initial_Wait       = 1[s]
Noe                = FALSE
Repetition_Time    = 10.34217728[s]
```

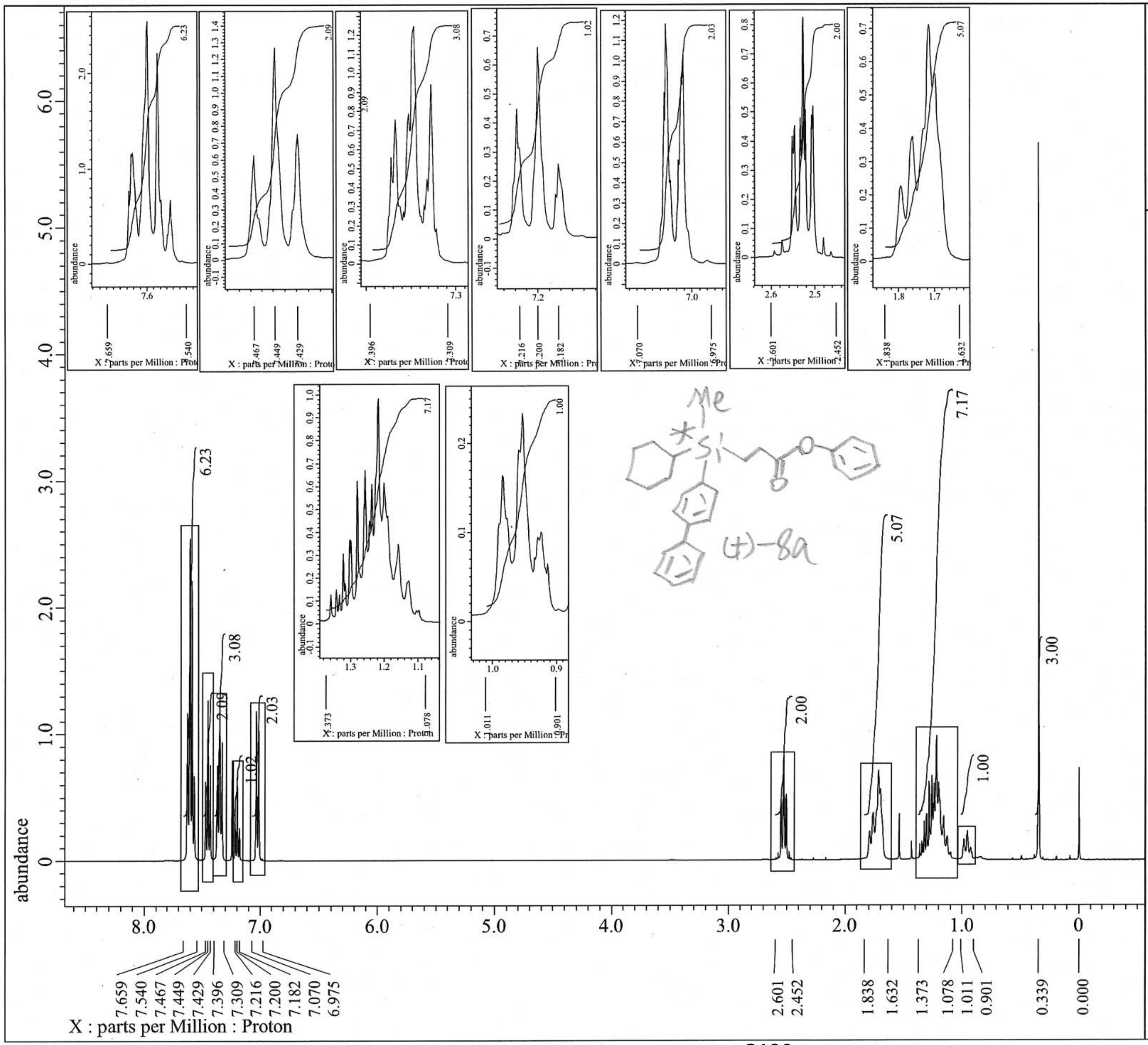


```
---- PROCESSING PARAMETERS ----
dc_balance( 0, FALSE )
sexp( 0.2[Hz], 0.0[s] )
trapezoid( 0[%], 0[%], 80[%], 100[%] )
zerofill( 1, TRUE )
fft( 1, TRUE, TRUE )
machinephase
ppm

数据来源: wxh-181-3_Proton-1-1.jdf

Filename            = wxh-181-3_Proton-1-2.jdf
Author              = element
Experiment          = proton.jxp
Sample_Id           = wxh-181-3
Solvent             = CHLOROFORM-D
Actual_Start_Time   = 14-DEC-2021 17:04:07
Revision_Time       = 28-JUN-2023 20:49:15

Comment             = single_pulse
Data_Format         = 1D COMPLEX
Dim_Size            = 13107
X_Domain            = Proton
Dim_Title           = Proton
Dim_Units           = [ppm]
Dimensions          = X
Spectrometer        = DELTA2_NMR

Field_Strength      = 9.4073814[T] (400[MHz])
X_Acq_Duration      = 2.18103808[s]
X_Domain            = 1H
X_Freq              = 400.53219825[MHz]
X_Offset            = 5[ppm]
X_Points            = 16384
X_Prescans          = 1
X_Resolution        = 0.45849727[Hz]
X_Sweep             = 7.51201923[kHz]
X_Sweep_Clipped     = 6.00961538[kHz]
Irr_Domain          = Proton
Irr_Freq            = 400.53219825[MHz]
Irr_Offset          = 5[ppm]
Tri_Domain          = Proton
Tri_Freq            = 400.53219825[MHz]
Tri_Offset          = 5[ppm]
Clipped             = FALSE
Scans               = 8
Total_Scans         = 8

Relaxation_Delay    = 5[s]
Recvr_Gain          = 32
Temp_Get            = 18.6[dC]
X_90_Width          = 6[us]
X_Acq_Time          = 2.18103808[s]
X_Angle             = 45[deg]
X_Atn               = 0.8[dB]
X_Pulse             = 3[us]
Irr_Mode            = Off
Tri_Mode            = Off
Dante_Presat        = FALSE
Initial_Wait        = 1[s]
Repetition_Time     = 7.18103808[s]
```

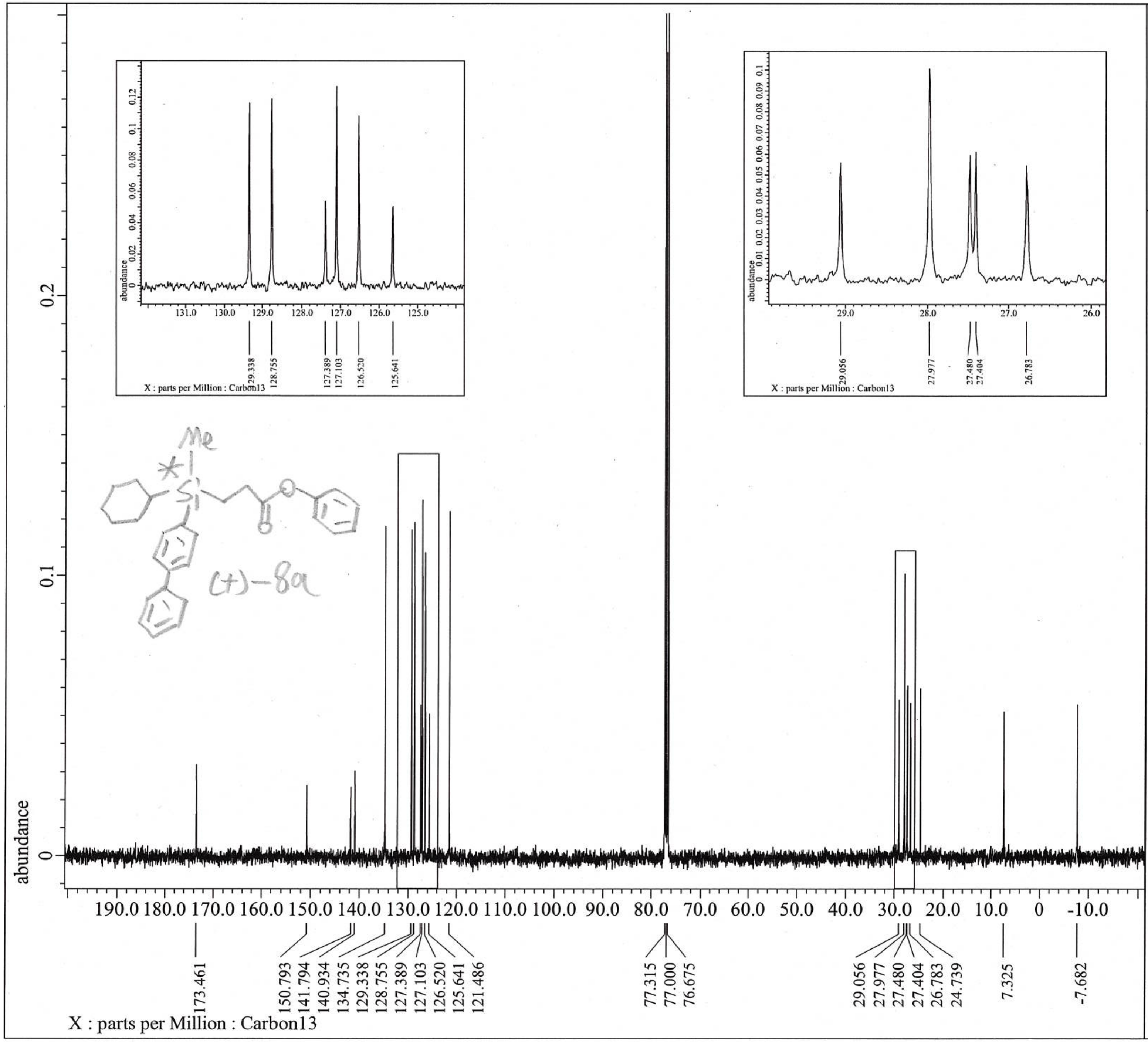


```
---- PROCESSING PARAMETERS ----
dc_balance( 0, FALSE )
sexp( 2.0[Hz], 0.0[s] )
trapezoid( 0[%], 0[%], 80[%], 100[%] )
zerofill( 1, TRUE )
fft( 1, TRUE, TRUE )
machinephase
ppm

数据来源: wxh-181-3_Carbon-1-1.jdf

Filename            = wxh-181-3_Carbon-1-2.jdf
Author              = element
Experiment          = carbon.jxp
Sample_Id           = wxh-181-3
Solvent             = CHLOROFORM-D
Actual_Start_Time   = 14-DEC-2021 19:51:02
Revision_Time       =  6-JAN-2022 16:09:37

Comment             = single pulse decoupled ga
Data_Format         = 1D COMPLEX
Dim_Size            = 26214
X_Domain            = Carbon
Dim_Title           = Carbon13
Dim_Units           = [ppm]
Dimensions          = X
Site                = JNM-ECS400
Spectrometer        = DELTA2_NMR

Field_Strength      = 9.37221[T] (400[MHz])
X_Acq_Duration      = 1.04333312[s]
X_Domain            = 13C
X_Freq              = 100.33735165[MHz]
X_Offset            = 100.0[ppm]
X_Points            = 32768
X_Prescans          = 4
X_Resolution        = 0.95846665[Hz]
X_Sweep             = 31.40703518[kHz]
X_Sweep_Clipped     = 25.12562814[kHz]
Irr_Domain          = Proton
Irr_Freq            = 399.03472754[MHz]
Irr_Offset          = 5.0[ppm]
Clipped             = FALSE
Scans               = 256
Total_Scans         = 256

Relaxation_Delay    = 2[s]
Recvr_Gain          = 50
Temp_Get            = 20.6[dC]
X_90_Width          = 10.9[us]
X_Acq_Time          = 1.04333312[s]
X_Angle             = 30[deg]
X_Atn               = 5.4[dB]
X_Pulse             = 3.63333333[us]
Irr_Atn_Dec         = 25.823[dB]
Irr_Atn_Noe         = 25.823[dB]
Irr_Noise           = WALTZ
Irr_Pwidth          = 0.115[ms]
Decoupling          = TRUE
Initial_Wait        = 1[s]
Noe                 = TRUE
Noe_Time            = 2[s]
Repetition_Time     = 3.04333312[s]
```

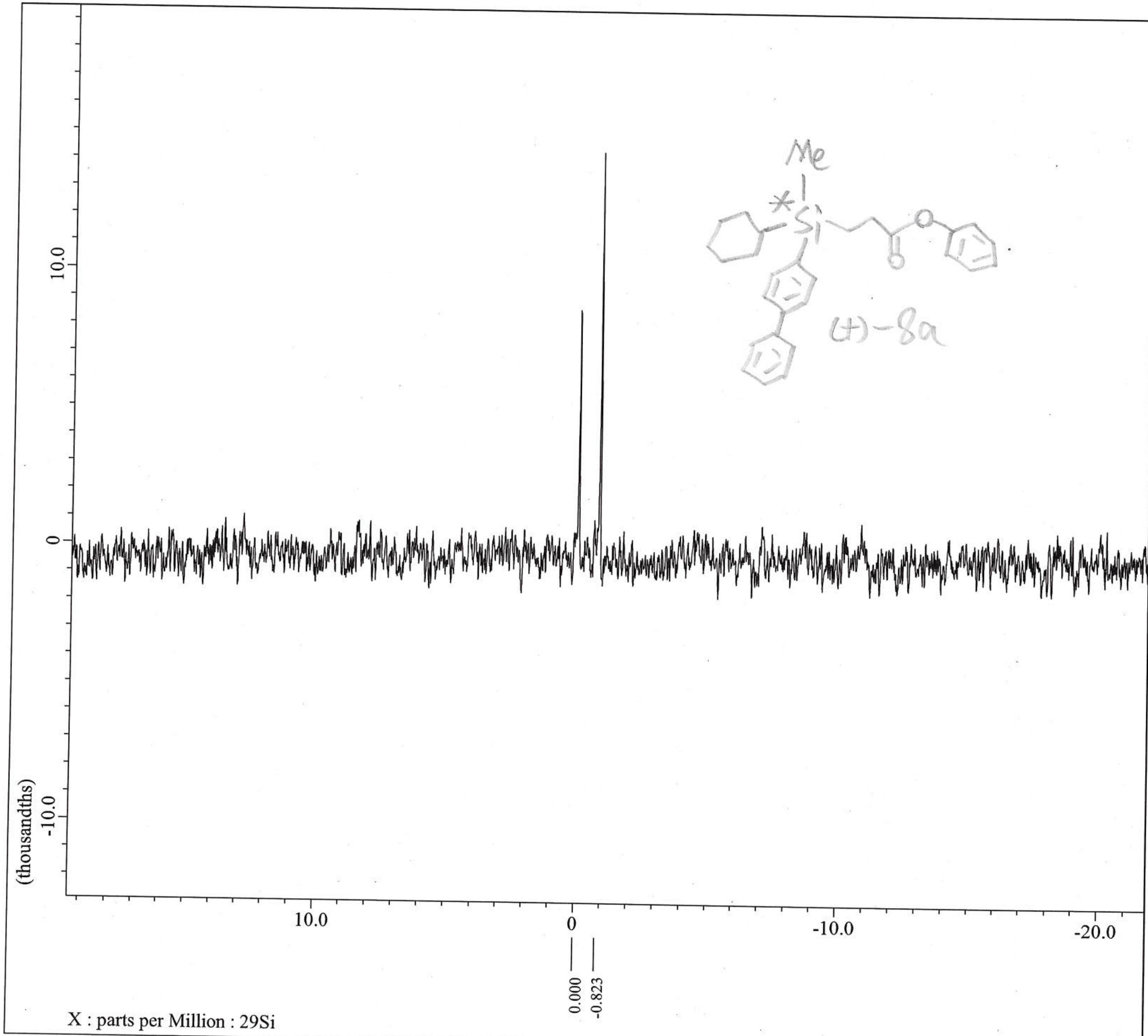

---- PROCESSING PARAMETERS ----
dc_balance(0, FALSE)
sexp(2.0[Hz], 0.0[s])
trapezoid3(0[%], 80[%], 100[%])
zerofill(1, TRUE)
fft(1, TRUE, TRUE)
machinephase
ppm
phase(40.54198, 0, 50[%])

数据来源: wxh-181-3-Si-1.jdf

Filename	= wxh-181-3-Si-2.jdf
Author	= element
Experiment	= single_pulse_dec
Sample_Id	= S#755661
Solvent	= CHLOROFORM-D
Actual_Start_Time	= 15-DEC-2021 03:44:58
Revision_Time	= 6-JAN-2022 16:59:28
Comment	= single pulse decoupled ga
Data_Format	= 1D COMPLEX
Dim_Size	= 26214
X_Domain	= 29Si
Dim_Title	= 29Si
Dim_Units	= [ppm]
Dimensions	= X
Site	= ECS 400
Spectrometer	= JNM-ECS400
Field_Strength	= 9.20197068[T] (390[MHz])
X_Acq_Duration	= 1.34217728[s]
X_Domain	= 29Si
X_Freq	= 77.83692472[MHz]
X_Offset	= 0[ppm]
X_Points	= 32768
X_Prescans	= 4
X_Resolution	= 0.74505806[Hz]
X_Sweep	= 24.4140625[kHz]
Irr_Domain	= 1H
Irr_Freq	= 391.78655441[MHz]
Irr_Offset	= 5[ppm]
Clipped	= TRUE
Scans	= 700
Total_Scans	= 700
Relaxation_Delay	= 8[s]
Recvr_Gain	= 48
Temp_Get	= 20.5[dC]
X_90_Width	= 10[us]
X_Acq_Time	= 1.34217728[s]
X_Angle	= 30[deg]
X_Atn	= 4.9[dB]
X_Pulse	= 3.33333333[us]
Irr_Atn_Dec	= 22.45[dB]
Irr_Noise	= WALTZ
Decoupling	= TRUE
Initial_Wait	= 1[s]
Noe	= FALSE
Repetition_Time	= 9.34217728[s]

D-2000 Elite HPLC System Manager Report

Analyzed Date and Time: 2021/08/05 17:49

Reported Date and Time: 2021/08/05 19:45

Processed Date and Time: 2021/08/05 19:45

Data Path: C:\WIN32APP\D2000HSM\Isocratic\DATA\3542\

Processing Method: 0.0/100.0 iPrOH/Hexane

System (acquisition): Sys 1

Application(data): Isocratic HPLC

Sample Name: WXH-132-1-OJ-0%

Injection from this vial: 1 of 1

Sample Description:

Series: 3542

Vial Number: 181

Vial Type: UNK

Volume: 10.0 ul

Chrom Type: HPLC Channel : 1

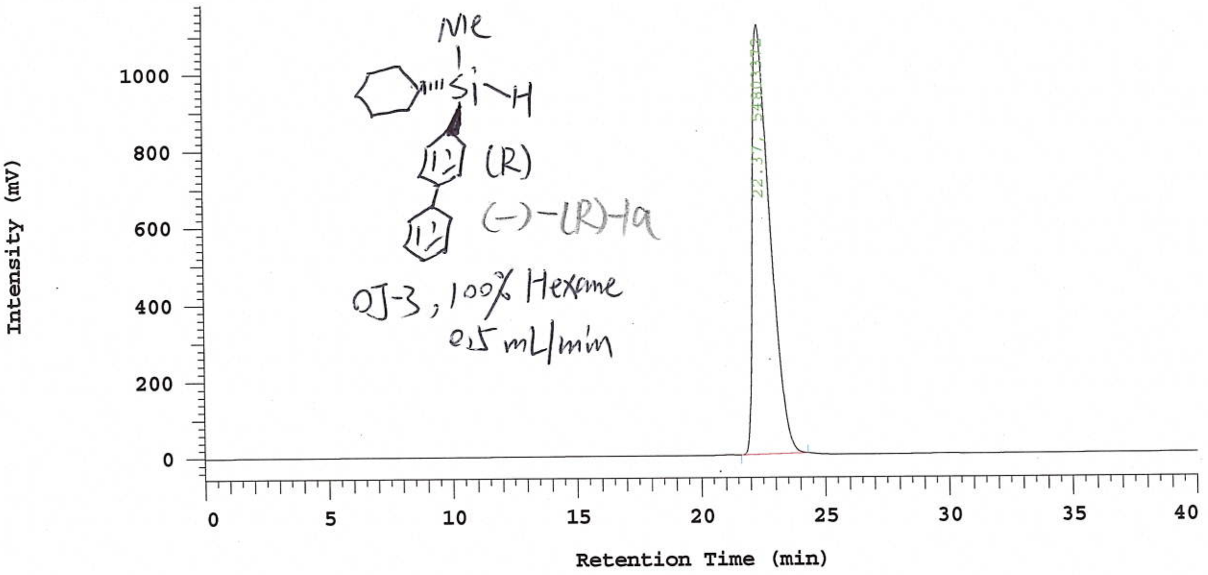

Processing Method: 0.0/100.0 iPrOH/Hexane

Column Type: OD-H 2

Method Developer: Administrator

Pump A: L-2130

Pump A Solvent A: Hexane

Pump A Solvent B: 10/90 iPrOH/Hexane

Pump A Solvent C: iPrOH

Pump A Solvent D: EtOH

Method Description:

Chrom Type: HPLC Channel : 1

Peak Quantitation: AREA

Calculation Method: AREA%

No.	RT	Area	Area %
1	22.37	54803372	100.000
		54803372	100.000

Peak rejection level: 0

D-2000: Isocratic HPLC Series: 3544 Report Name: modified System: Sys 1

D-2000 Elite HPLC System Manager Report

Analyzed Date and Time: 2021/08/05 19:32

Reported Date and Time: 2021/08/05 20:36

Processed Date and Time: 2021/08/05 20:36

Data Path: C:\WIN32APP\D2000HSM\Isocratic\DATA\3544\

Processing Method: 0.0/100.0 iPrOH/Hexane

System (acquisition): Sys 1

Application(data): Isocratic HPLC

Sample Name: WXH-132-race-OJ-0%

Injection from this vial: 1 of 1

Sample Description:

Series: 3544

Vial Number: 183

Vial Type: UNK

Volume: 10.0 ul

Chrom Type: HPLC Channel : 1

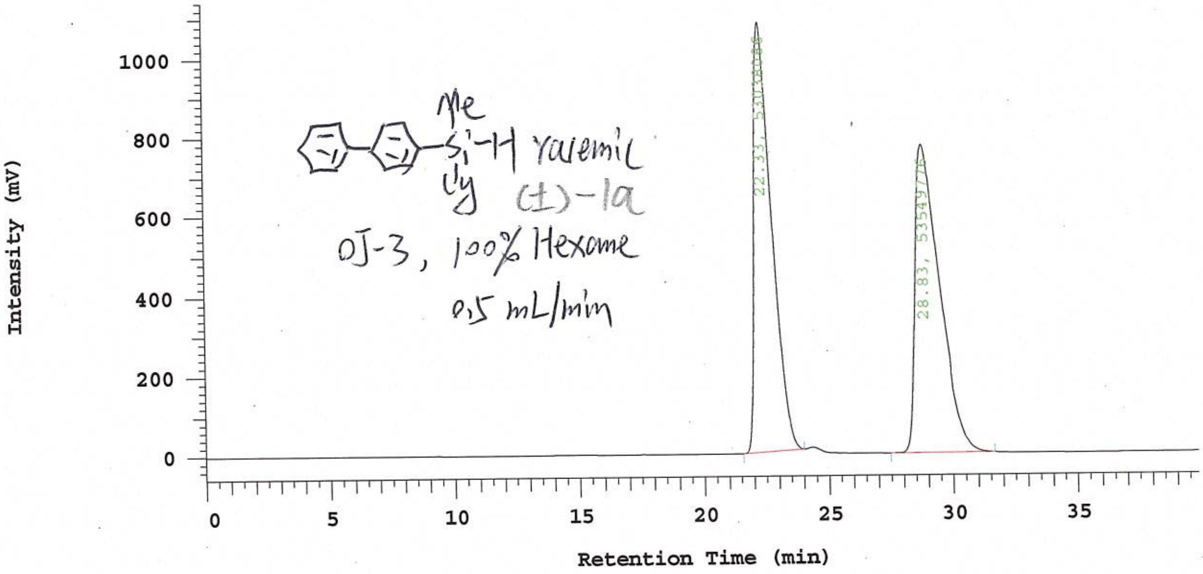

Processing Method: 0.0/100.0 iPrOH/Hexane

Column Type: OD-H 2

Method Developer: Administrator

Pump A: L-2130

Pump A Solvent A: Hexane

Pump A Solvent B: 10/90 iPrOH/Hexane

Pump A Solvent C: iPrOH

Pump A Solvent D: EtOH

Method Description:

Chrom Type: HPLC Channel : 1

Peak Quantitation: AREA

Calculation Method: AREA%

No.	RT	Area	Area %
1	22.33	53038086	49.760
2	28.83	53549776	50.240
		1.065E+08	100.000

Peak rejection level: 0

D-2000: Isocratic HPLC Series: 3900 Report Name: modified System: Sys 1

D-2000 Elite HPLC System Manager Report

Analyzed Date and Time: 2022/04/04 13:46 Reported Date and Time: 2022/04/06 13:20

Processed Date and Time: 2022/04/04 14:49

Data Path: C:\WIN32APP\D2000HSM\Isocratic\DATA\3900\
Processing Method: 05/95 iPrOH/Hexane

System (acquisition): Sys 1
Application(data): Isocratic HPLC
Sample Name: WXH-145-1-5% IPA
Injection from this vial: 1 of 1
Sample Description:

Series: 3900
Vial Number: 182
Vial Type: UNK
Volume: 10.0 ul

Chrom Type: HPLC Channel : 1

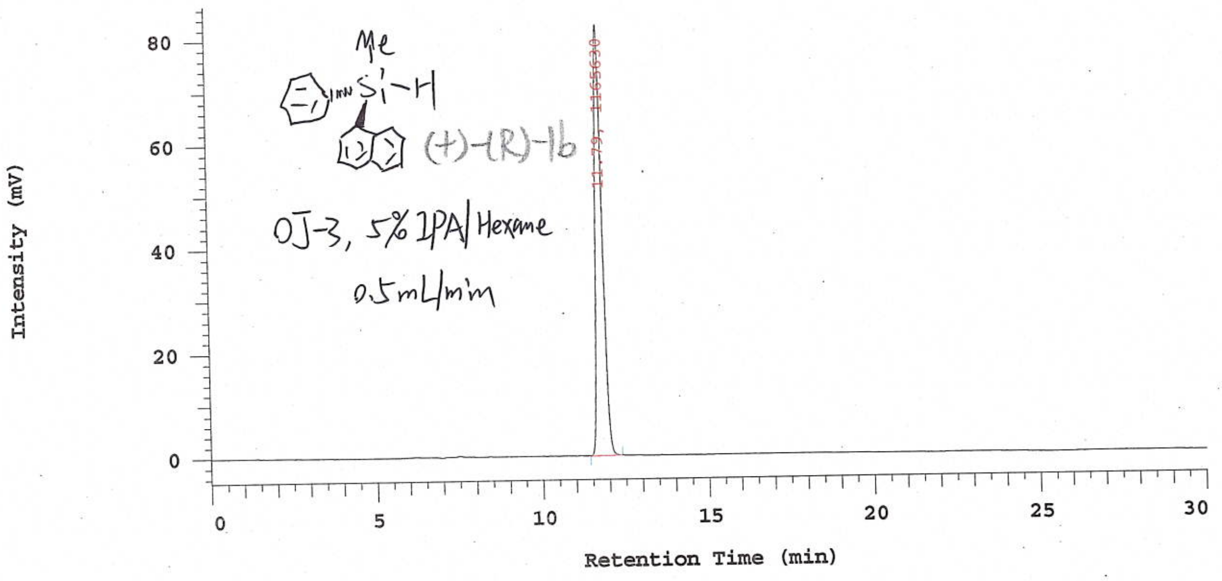

Processing Method: 05/95 iPrOH/Hexane
Column Type: OD-H 2
Pump A: L-2130
Pump A Solvent A: Hexane
Pump A Solvent C: iPrOH
Method Description:

Method Developer: Administrator
Pump A Solvent B: 10/90 iPrOH/Hexane
Pump A Solvent D: iPrOH

Chrom Type: HPLC Channel : 1

Peak Quantitation: AREA
Calculation Method: AREA%

No.	RT	Area	Area %
1	11.79	1165630	100.000
		1165630	100.000

Peak rejection level: 0

D-2000: Isocratic HPLC Series: 3899 Report Name: modified System: Sys 1

D-2000 Elite HPLC System Manager Report

Analyzed Date and Time: 2022/04/04 12:44
Reported Date and Time: 2022/04/06 13:21
Processed Date and Time: 2022/04/06 13:21
Data Path: C:\WIN32APP\D2000HSM\Isocratic\DATA\3899\
Processing Method: 05/95 iPrOH/Hexane
System (acquisition): Sys 1
Application(data): Isocratic HPLC
Sample Name: WXH-145-5% IPA
Injection from this vial: 1 of 1
Sample Description:
Series: 3899
Vial Number: 181
Vial Type: UNK
Volume: 10.0 ul

Chrom Type: HPLC Channel : 1

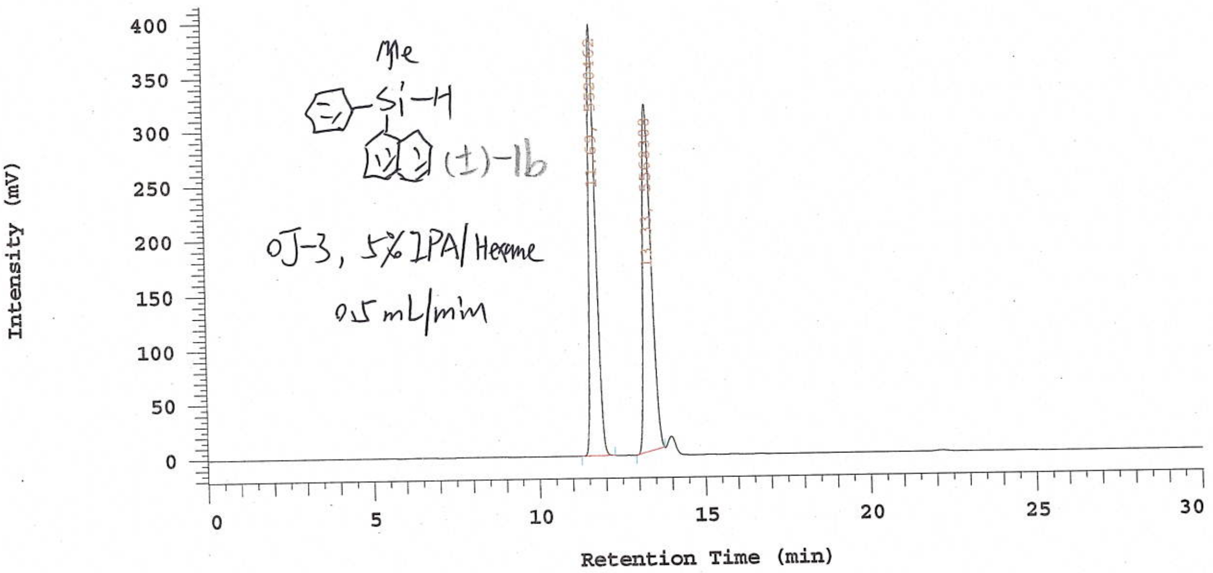

Processing Method: 05/95 iPrOH/Hexane
Column Type: OD-H 2
Method Developer: Administrator
Pump A: L-2130
Pump A Solvent A: Hexane
Pump A Solvent B: 10/90 iPrOH/Hexane
Pump A Solvent C: iPrOH
Pump A Solvent D: iPrOH
Method Description:

Chrom Type: HPLC Channel : 1
Peak Quantitation: AREA
Calculation Method: AREA%

No.	RT	Area	Area %
1	11.69	5520462	49.784
2	13.33	5568308	50.216
		11088770	100.000

Peak rejection level: 0

D-2000 Elite HPLC System Manager Report

Analyzed Date and Time: 2021/11/27 19:38

Reported Date and Time: 2021/11/29 09:13

Processed Date and Time: 2021/11/29 09:13

Data Path: C:\WIN32APP\D2000HSM\Isocratic\DATA\3768\

Processing Method: 100 MeOH 1.0mL/min

System (acquisition): Sys 1

Application(data): Isocratic HPLC

Sample Name: wxh-174-OJ-100%MeOH

Injection from this vial: 1 of 1

Sample Description:

Series: 3768

Vial Number: 183

Vial Type: UNK

Volume: 10.0 ul

Chrom Type: HPLC Channel : 1

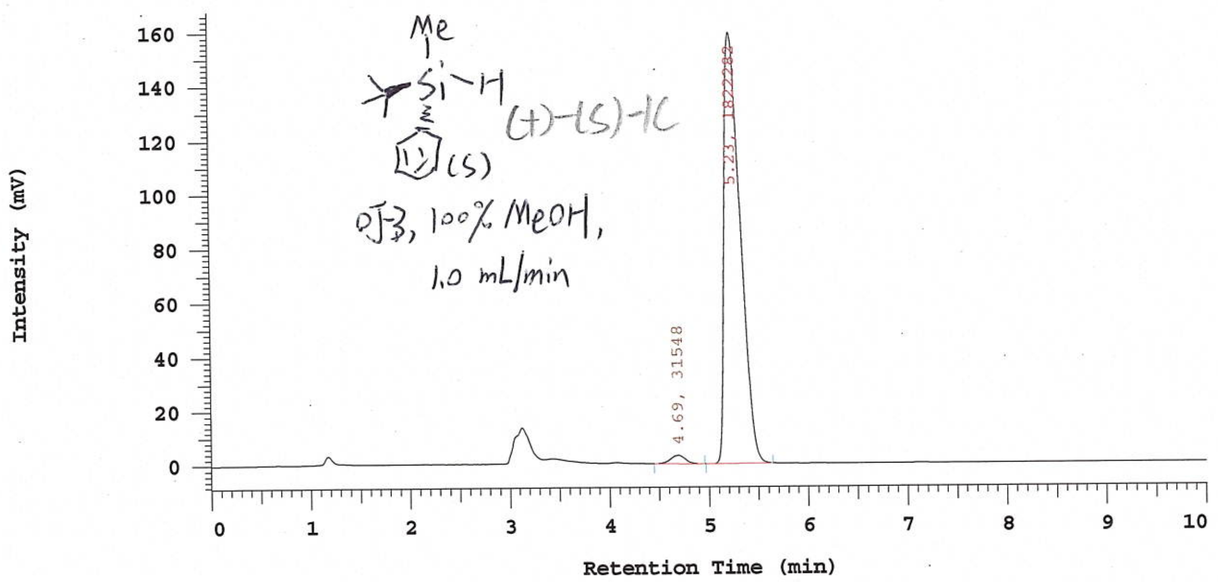

Processing Method: 100 MeOH 1.0mL/min

Column Type: OD-H 2

Method Developer: Administrator

Pump A: L-2130

Pump A Solvent A: Hexane

Pump A Solvent B: 10/90 iPrOH/Hexane

Pump A Solvent C: iPrOH

Pump A Solvent D: MeOH

Method Description:

Chrom Type: HPLC Channel : 1

Peak Quantitation: AREA

Calculation Method: AREA%

No.	RT	Area	Area %
1	4.69	31548	1.702
2	5.23	1822282	98.298
		1853830	100.000

Peak rejection level: 0

D-2000: Isocrati c HPLC Series: 3767 Report Name: modified System: Sys 1

D-2000 Elite HPLC System Manager Report

Analyzed Date and Time: 2021/11/27 18:56
Reported Date and Time: 2021/11/30 13:50

Processed Date and Time: 2021/11/30 13:50

Data Path: C:\WIN32APP\D2000HSM\Isocratic\DATA\3767\

Processing Method: 0.0/100.0 iPrOH/Hexane

System (acquisition): Sys 1
Series: 3767

Application(data): Isocratic HPLC
Vial Number: 182

Sample Name: wxh-174-OJ-100%MeOH
Vial Type: UNK

Injection from this vial: 1 of 1
Volume: 10.0 ul

Sample Description:

Chrom Type: HPLC Channel : 1

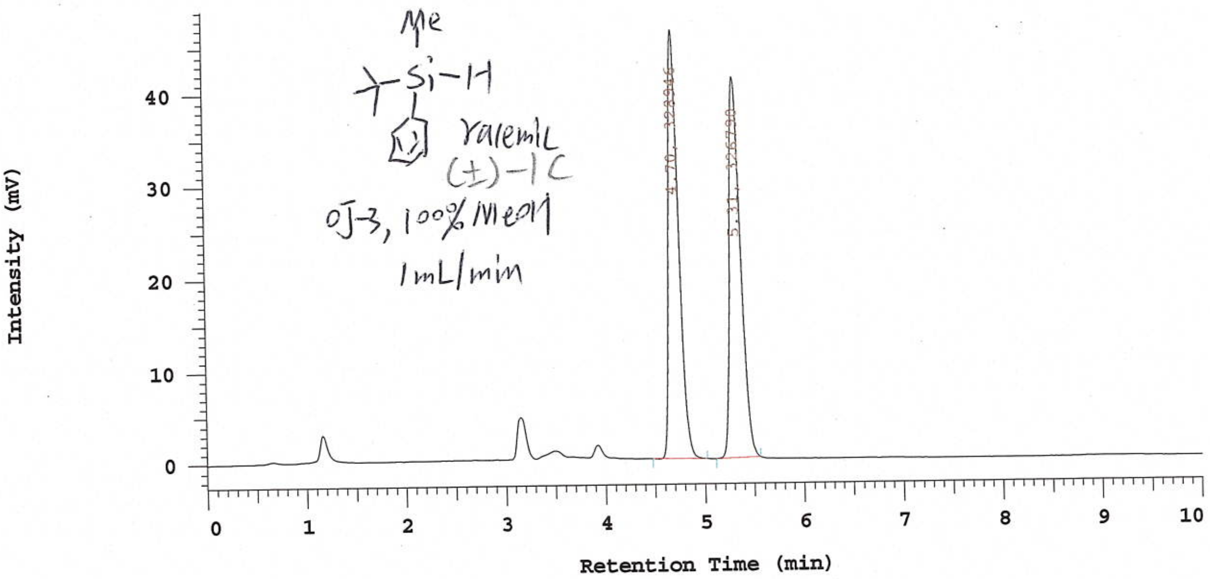

Processing Method: 0.0/100.0 iPrOH/Hexane

Column Type: OD-H 2
Method Developer: Administrator

Pump A: L-2130

Pump A Solvent A: Hexane
Pump A Solvent B: 10/90 iPrOH/Hexane

Pump A Solvent C: iPrOH
Pump A Solvent D: MeOH

Method Description:

Chrom Type: HPLC Channel : 1

Peak Quantitation: AREA

Calculation Method: AREA%

No.	RT	Area	Area %
1	4.70	328946	50.164
2	5.31	326790	49.836
		655736	100.000

Peak rejection level: 0

Chromaster System Manager Report

Analyzed Date and Time: 2022/04/04 16:13

Reported Date and Time: 2022/04/04 19:07:49

Processed Date and Time: 2022/04/04 19:07

Data Path: C:\WIN32APP\CHROMASTER\WXH\DATA\0415\

Processing Method: ID_UV

System (acquisition): Sys 1

Application(data): WXH

Sample Name: WXH-237-1-ID-IPA 0%

Injection from this vial: 1 of 1

Sample Description:

Series: 0415

Vial Number: 12

Vial Type: UNK

Volume: 10.0 ul

Chrom Type: Fixed WL Chromatogram, 250 nm

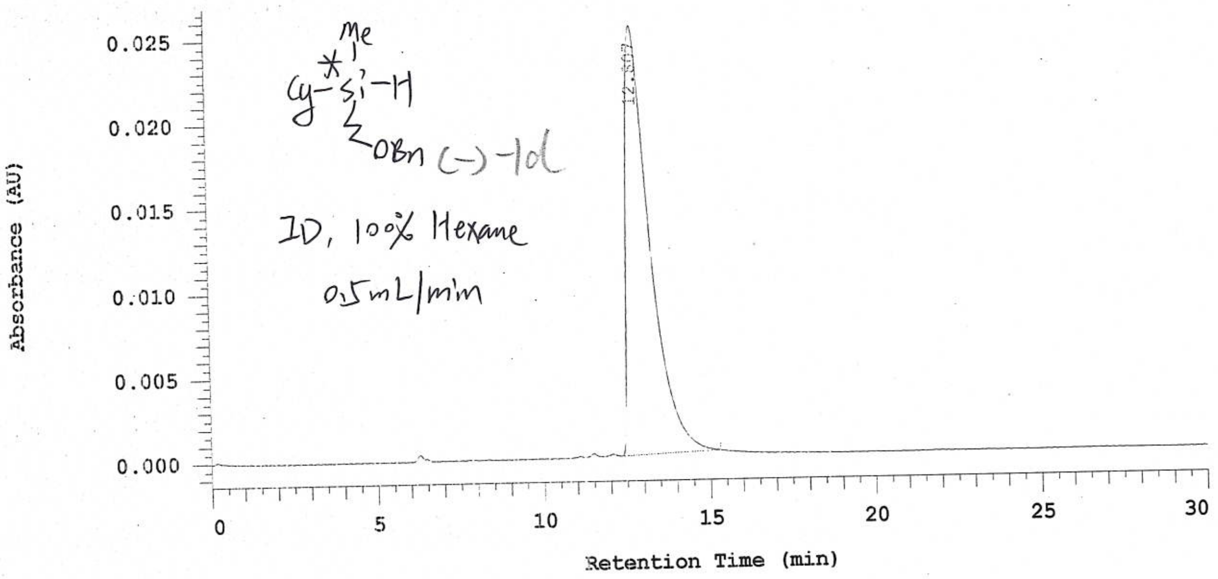

Processing Method: ID_UV

Method Developer:

Pump 1: 5110

Pump 1 Solvent A: hexane

Pump 1 Solvent B: 2-propanol

Pump 1 Solvent C:

Pump 1 Solvent D:

Method Description:

Chrom Type: Fixed WL Chromatogram, 250 nm

Peak Quantitation: AREA

Calculation Method: AREA%

No.	RT	Area	Conc 1	BC
1	12.807	626753	100.000	BB
		626753	100.000	

Peak rejection level: 0

Chromaster System Manager Report

Analyzed Date and Time: 2022/04/04 15:12
Reported Date and Time: 2022/04/04 19:05:58

Processed Date and Time: 2022/04/04 19:05

Data Path: C:\WIN32APP\CHROMASTER\WXH\DATA\0414\
Processing Method: ID_UV

System (acquisition): Sys 1
Application(data): WXH
Sample Name: WXH-237-ID-IPA 0%
Injection from this vial: 1 of 1
Sample Description:

Series: 0414
Vial Number: 11
Vial Type: UNK
Volume: 10.0 ul

Chrom Type: Fixed WL Chromatogram, 250 nm

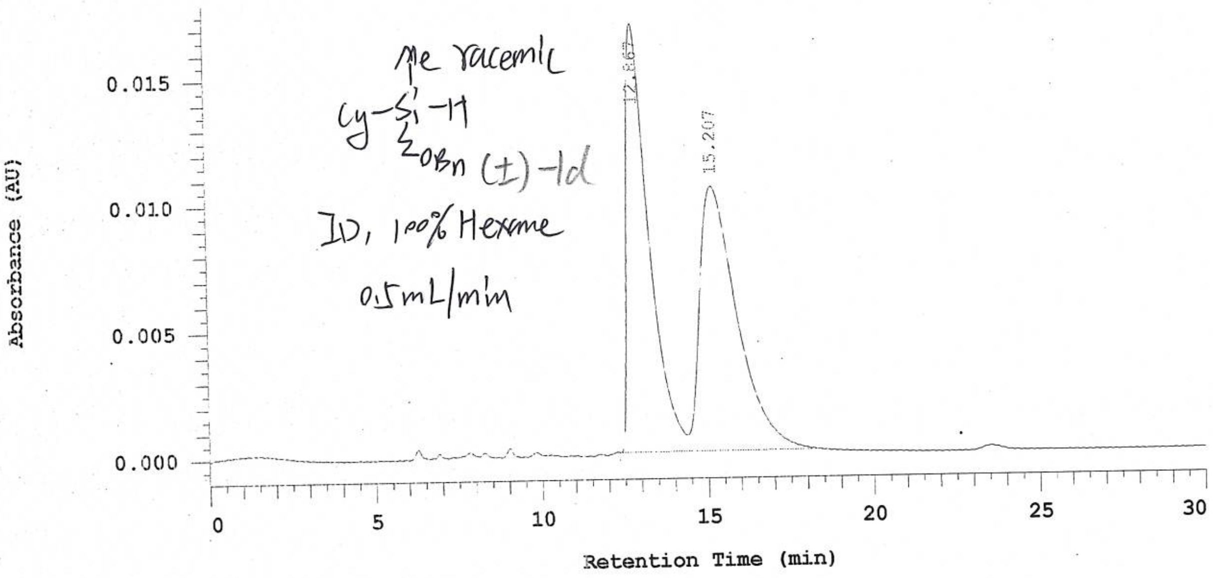

Processing Method: ID_UV
Method Developer:
Pump 1: 5110
Pump 1 Solvent A: hexane
Pump 1 Solvent B: 2-propanol
Pump 1 Solvent C:
Pump 1 Solvent D:
Method Description:

Chrom Type: Fixed WL Chromatogram, 250 nm

Peak Quantitation: AREA
Calculation Method: AREA%

No.	RT	Area	Conc 1	BC
1	12.867	394556	49.187	BV
2	15.207	407596	50.813	VB
		802152	100.000	

Peak rejection level: 0

D-2000 Elite HPLC System Manager Report

Analyzed Date and Time: 2021/09/01 15:59
Reported Date and Time: 2021/09/02 20:55
Processed Date and Time: 2021/09/02 20:54
Data Path: C:\WIN32APP\D2000HSM\Isocratic\DATA\3569\
Processing Method: 10/90 iPrOH/Hexane
System (acquisition): Sys 1
Series: 3569
Application(data): Isocratic HPLC
Vial Number: 182
Sample Name: WXH-106-1-OJ-0%
Vial Type: UNK
Injection from this vial: 1 of 1
Volume: 10.0 ul
Sample Description:

Chrom Type: HPLC Channel : 1

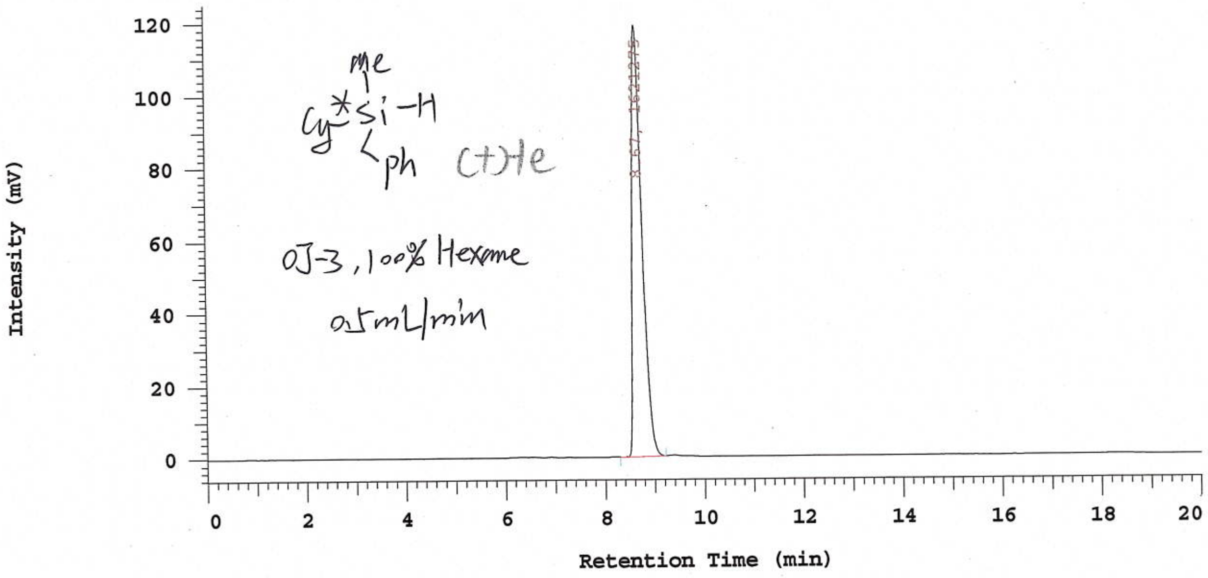

Processing Method: 10/90 iPrOH/Hexane
Column Type: OD-H 2
Method Developer: Administrator
Pump A: L-2130
Pump A Solvent A: Hexane
Pump A Solvent B: 10/90 iPrOH/Hexane
Pump A Solvent C: iPrOH
Pump A Solvent D: EtOH
Method Description:

Chrom Type: HPLC Channel : 1
Peak Quantitation: AREA
Calculation Method: AREA%

No.	RT	Area	Area %
1	8.67	1621275	100.000
		1621275	100.000

Peak rejection level: 0

D-2000: Isocratic HPLC Series: 3568 Report Name: modified System: Sys 1

D-2000 Elite HPLC System Manager Report

Analyzed Date and Time: 2021/09/01 15:18

Reported Date and Time: 2021/09/02 21:01

Processed Date and Time: 2021/09/02 21:01

Data Path: C:\WIN32APP\D2000HSM\Isocratic\DATA\3568\

Processing Method: 10/90 iPrOH/Hexane

System (acquisition): Sys 1

Application(data): Isocratic HPLC

Sample Name: WXH-106-OJ-0%

Injection from this vial: 1 of 1

Sample Description:

Series: 3568

Vial Number: 181

Vial Type: UNK

Volume: 10.0 ul

Chrom Type: HPLC Channel : 1

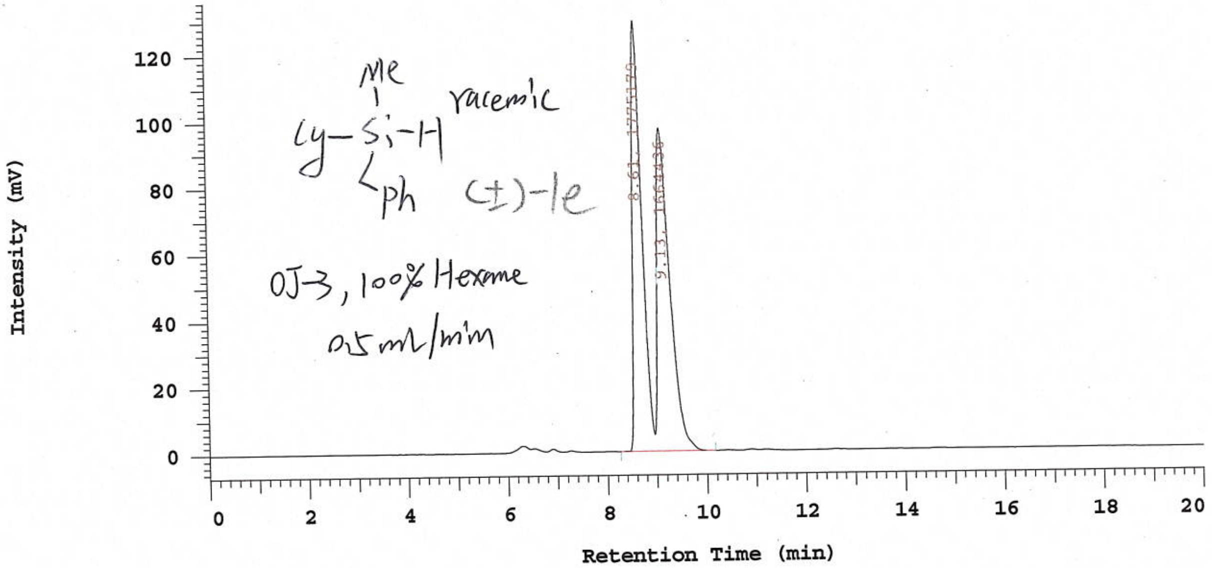

Processing Method: 10/90 iPrOH/Hexane

Column Type: OD-H 2

Method Developer: Administrator

Pump A: L-2130

Pump A Solvent A: Hexane

Pump A Solvent B: 10/90 iPrOH/Hexane

Pump A Solvent C: iPrOH

Pump A Solvent D: EtOH

Method Description:

Chrom Type: HPLC Channel : 1

Peak Quantitation: AREA

Calculation Method: AREA%

No.	RT	Area	Area %
1	8.61	1775179	51.610
2	9.13	1664436	48.390
		3439615	100.000

Peak rejection level: 0

D-2000 Elite HPLC System Manager Report

Analyzed Date and Time: 2021/08/04 20:01 Reported Date and Time: 2021/08/04 21:01
Processed Date and Time: 2021/08/04 21:00
Data Path: C:\WIN32APP\D2000HSM\Isocratic\DATA\3541\
Processing Method: 0.0/100.0 iPrOH/Hexane
System (acquisition): Sys 1 Series: 3541
Application(data): Isocratic HPLC Vial Number: 182
Sample Name: WXH-128-1-OJ-0% Vial Type: UNK
Injection from this vial: 1 of 1 Volume: 10.0 ul
Sample Description:

Chrom Type: HPLC Channel : 1

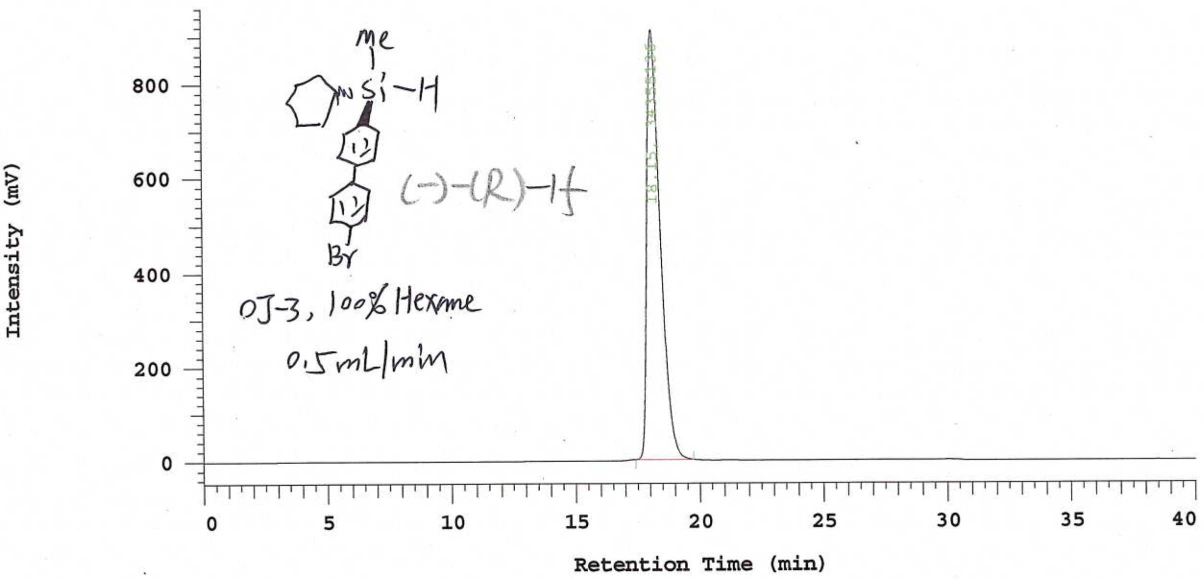

Processing Method: 0.0/100.0 iPrOH/Hexane
Column Type: OD-H 2 Method Developer: Administrator
Pump A: L-2130
Pump A Solvent A: Hexane Pump A Solvent B: 10/90 iPrOH/Hexane
Pump A Solvent C: iPrOH Pump A Solvent D: EtOH
Method Description:

Chrom Type: HPLC Channel : 1
Peak Quantitation: AREA
Calculation Method: AREA%

No.	RT	Area	Area %
1	18.15	34355136	100.000
		34355136	100.000

Peak rejection level: 0

D-2000 Elite HPLC System Manager Report

Analyzed Date and Time: 2021/08/04 19:10
Reported Date and Time: 2021/08/04 21:00
Processed Date and Time: 2021/08/04 21:00
Data Path: C:\WIN32APP\D2000HSM\Isocratic\DATA\3540\
Processing Method: 0.0/100.0 iPrOH/Hexane

System (acquisition): Sys 1
Application(data): Isocratic HPLC
Sample Name: WXH-128-race-OJ-0%
Injection from this vial: 1 of 1
Sample Description:

Series: 3540
Vial Number: 181
Vial Type: UNK
Volume: 10.0 ul

Chrom Type: HPLC Channel : 1

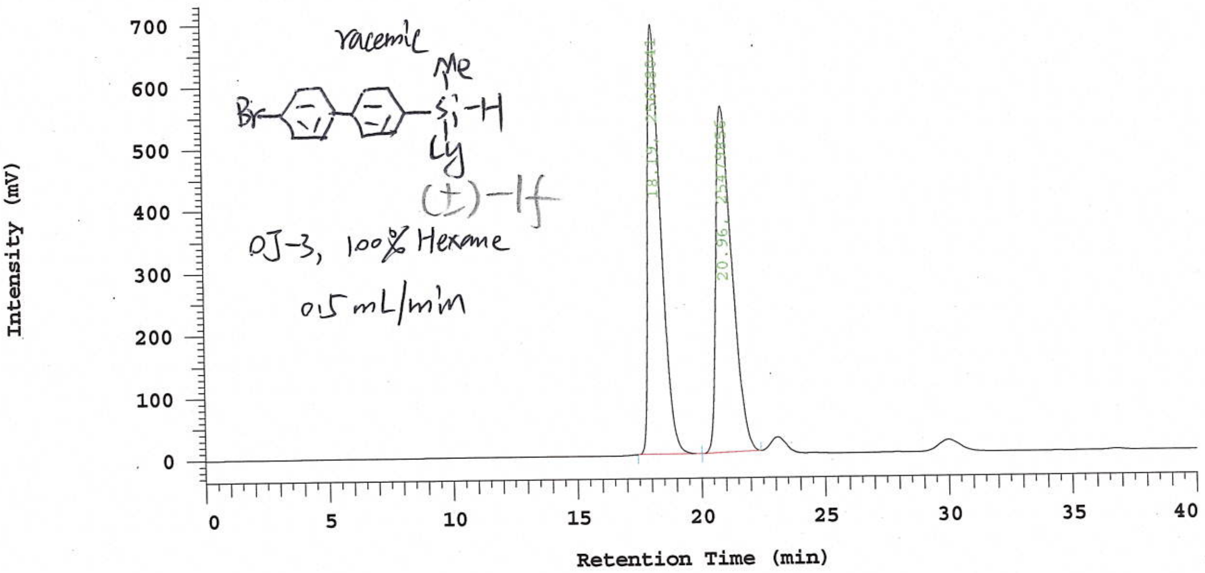

Processing Method: 0.0/100.0 iPrOH/Hexane
Column Type: OD-H 2
Method Developer: Administrator
Pump A: L-2130
Pump A Solvent A: Hexane
Pump A Solvent B: 10/90 iPrOH/Hexane
Pump A Solvent C: iPrOH
Pump A Solvent D: EtOH
Method Description:

Chrom Type: HPLC Channel : 1
Peak Quantitation: AREA
Calculation Method: AREA%

No.	RT	Area	Area %
1	18.19	25668041	50.184
2	20.96	25479856	49.816
		51147897	100.000

Peak rejection level: 0

D-2000 Elite HPLC System Manager Report

Analyzed Date and Time: 2022/10/11 19:58
Reported Date and Time: 2023/03/08 13:56
Processed Date and Time: 2023/03/08 13:56
Data Path: C:\WIN32APP\D2000HSM\Isocratic\DATA\4047\
Processing Method: 0.0/100.0 iPrOH/Hexane

System (acquisition): Sys 1
Series: 4047
Application(data): Isocratic HPLC
Vial Number: 182
Sample Name: wxh-284-0% IPA-ODOD
Vial Type: UNK
Injection from this vial: 1 of 1
Volume: 10.0 ul
Sample Description:

Chrom Type: HPLC Channel : 1

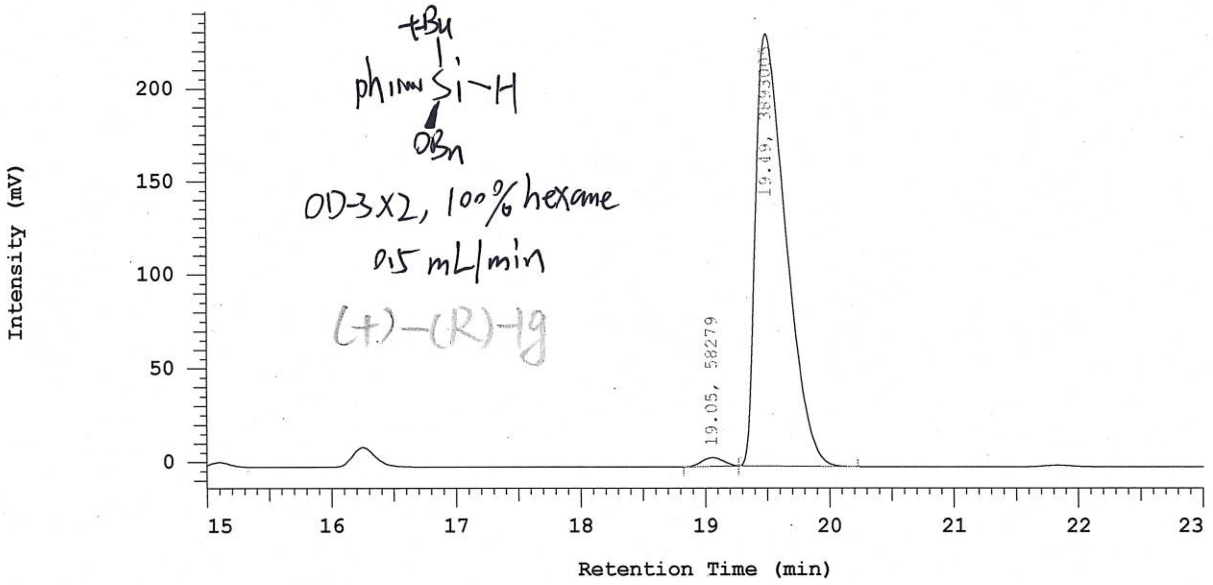

Processing Method: 0.0/100.0 iPrOH/Hexane
Column Type: OD-H 2
Method Developer: Administrator
Pump A: L-2130
Pump A Solvent A: Hexane
Pump A Solvent B: 10/90 iPrOH/Hexane
Pump A Solvent C: iPrOH
Pump A Solvent D: EtOH
Method Description:

Chrom Type: HPLC Channel : 1
Peak Quantitation: AREA
Calculation Method: AREA%

No.	RT	Area	Area %
1	19.05	58279	1.475
2	19.49	3893005	98.525
		3951284	100.000

Peak rejection level: 0

D-2000 Elite HPLC System Manager Report

Analyzed Date and Time: 2022/10/11 18:50
Reported Date and Time: 2023/03/08 13:55
Processed Date and Time: 2023/03/08 13:55
Data Path: C:\WIN32APP\D2000HSM\Isocratic\DATA\4046\
Processing Method: 0.0/100.0 iPrOH/Hexane

System (acquisition): Sys 1
Series: 4046
Application(data): Isocratic HPLC
Vial Number: 181
Sample Name: wxh-287-race-0% IPA-ODOD
Vial Type: UNK
Injection from this vial: 1 of 1
Volume: 10.0 ul
Sample Description:

Chrom Type: HPLC Channel : 1

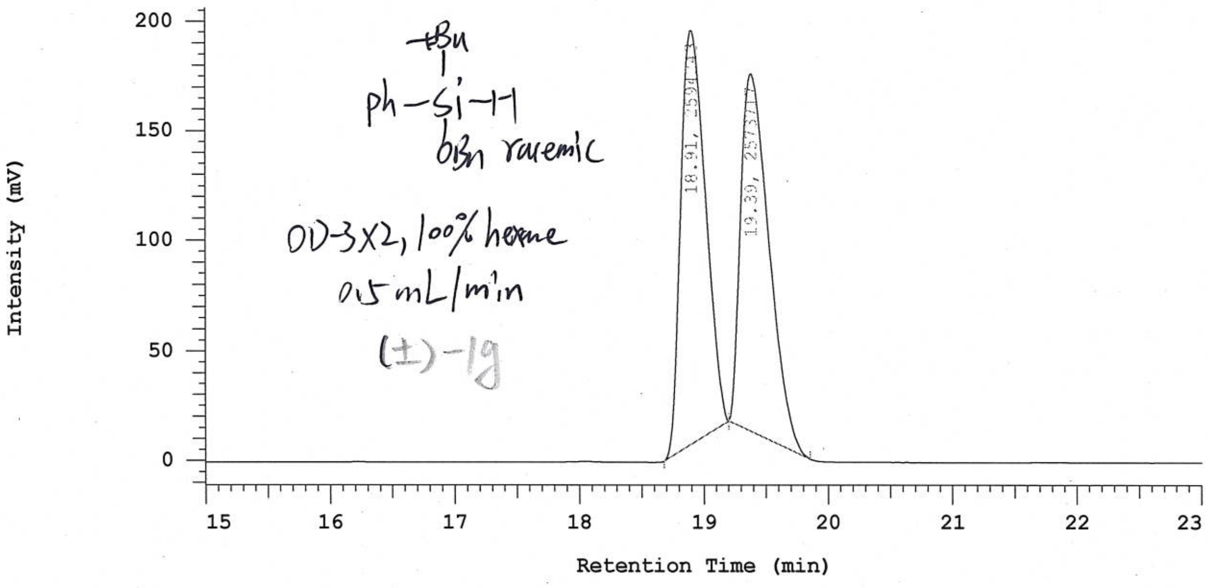

Processing Method: 0.0/100.0 iPrOH/Hexane
Column Type: OD-H 2
Method Developer: Administrator
Pump A: L-2130
Pump A Solvent A: Hexane
Pump A Solvent B: 10/90 iPrOH/Hexane
Pump A Solvent C: iPrOH
Pump A Solvent D: EtOH
Method Description:

Chrom Type: HPLC Channel : 1
Peak Quantitation: AREA
Calculation Method: AREA%

No.	RT	Area	Area %
1	18.91	2594243	50.199
2	19.39	2573717	49.801
		5167960	100.000

Peak rejection level: 0

D-2000 Elite HPLC System Manager Report

Analyzed Date and Time: 2023/02/08 10:07   Reported Date and Time: 2023/02/08 13:04

Processed Date and Time: 2023/02/08 12:56

Data Path: C:\WIN32APP\D2000HSM\Isocratic\DATA\4158\

Processing Method: 0.0/100.0 iPrOH/Hexane

System (acquisition): Sys 1   Series: 4158

Application(data): Isocratic HPLC   Vial Number: 181

Sample Name: wxh-406-OJOJ-0%IPA   Vial Type: UNK

Injection from this vial: 1 of 1   Volume: 10.0 ul

Sample Description:

Chrom Type: HPLC Channel : 1   20°C

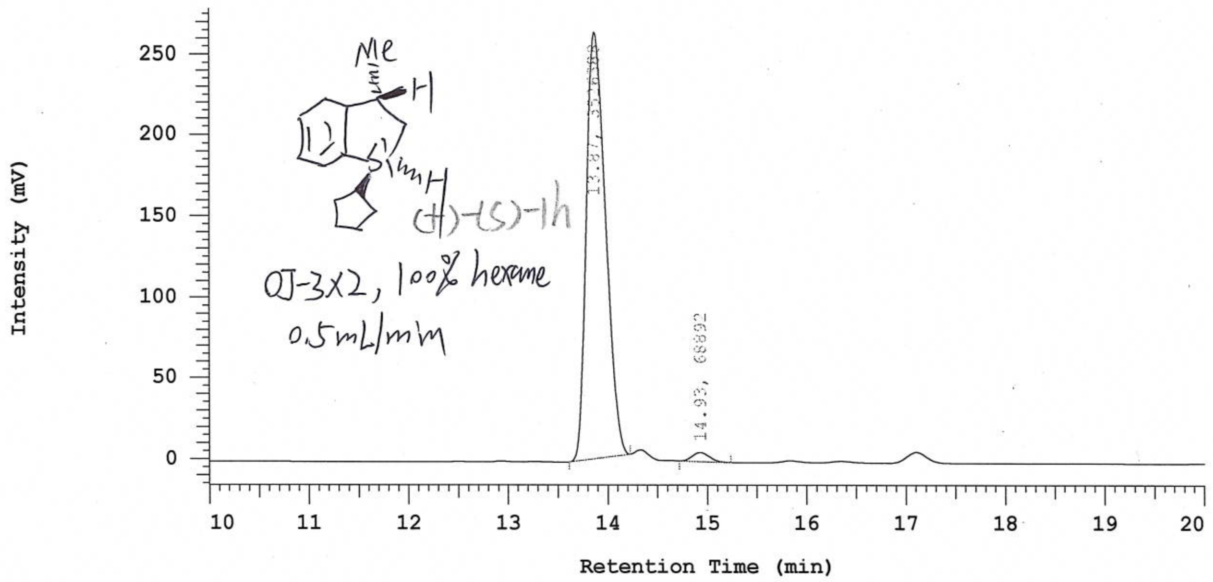

Processing Method: 0.0/100.0 iPrOH/Hexane

Column Type: OD-H 2   Method Developer: Administrator

Pump A: L-2130

Pump A Solvent A: Hexane   Pump A Solvent B: 10/90 iPrOH/Hexane

Pump A Solvent C: iPrOH   Pump A Solvent D: EtOH

Method Description:

Chrom Type: HPLC Channel : 1

Peak Quantitation: AREA

Calculation Method: AREA%

No.	RT	Area	Area %
1	13.87	3526388	98.084
2	14.93	68892	1.916
		3595280	100.000

Peak rejection level: 0

D-2000 Elite HPLC System Manager Report

Analyzed Date and Time: 2023/02/08 11:08
Reported Date and Time: 2023/02/08 13:04
Processed Date and Time: 2023/02/08 12:59
Data Path: C:\WIN32APP\D2000HSM\Isocratic\DATA\4159\
Processing Method: 0.0/100.0 iPrOH/Hexane

System (acquisition): Sys 1
Series: 4159
Application(data): Isocratic HPLC
Vial Number: 182
Sample Name: wxh-314-OJOJ-0%IPA
Vial Type: UNK
Injection from this vial: 1 of 1
Volume: 10.0 ul
20°C
Sample Description:

Chrom Type: HPLC Channel : 1

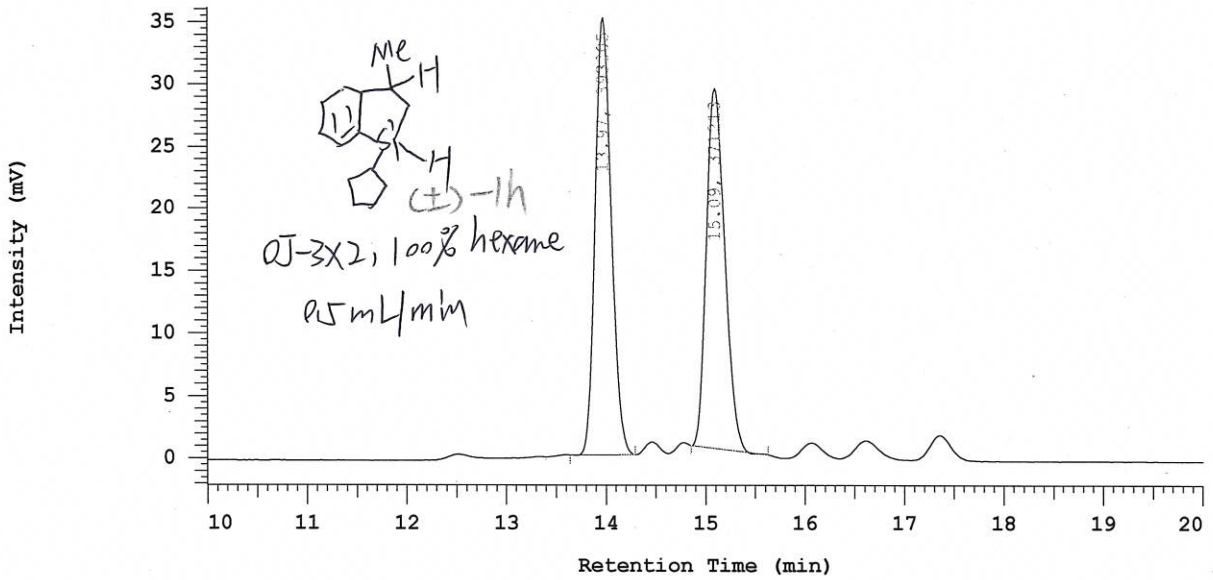

Processing Method: 0.0/100.0 iPrOH/Hexane
Column Type: OD-H 2
Method Developer: Administrator
Pump A: L-2130
Pump A Solvent A: Hexane
Pump A Solvent B: 10/90 iPrOH/Hexane
Pump A Solvent C: iPrOH
Pump A Solvent D: EtOH
Method Description:

Chrom Type: HPLC Channel : 1
Peak Quantitation: AREA
Calculation Method: AREA%

No.	RT	Area	Area %
1	13.97	393205	51.438
2	15.09	371213	48.562
		764418	100.000

Peak rejection level: 0

Chromaster System Manager Report

Analyzed Date and Time: 2021/08/19 14:06

Reported Date and Time: 2021/08/19 15:45:58

Processed Date and Time: 2021/08/19 15:45

Data Path: C:\WIN32APP\CHROMASTER\WXH\DATA\0306\

Processing Method: IB_UV

System (acquisition): Sys 1

Series: 0306

Application(data): WXH

Vial Number: 11

Sample Name: WXH-138-IB-IPA 0%

Vial Type: UNK

Injection from this vial: 1 of 1

Volume: 10.0 ul

Sample Description:

Chrom Type: Fixed WL Chromatogram, 250 nm

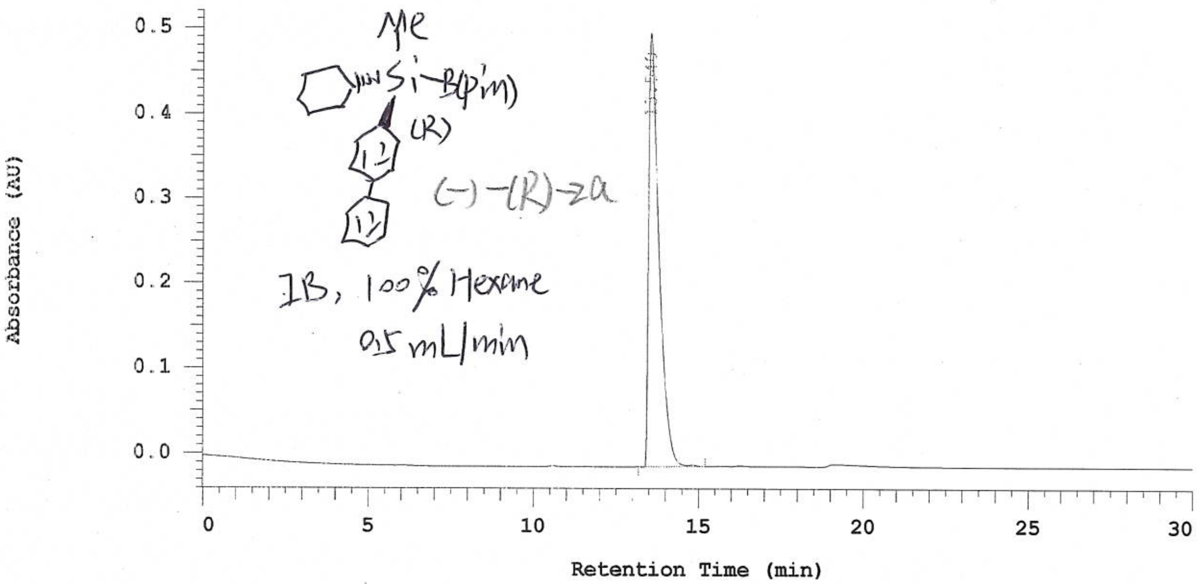

Processing Method: IB_UV

Method Developer:

Pump 1: 5110

Pump 1 Solvent A: hexane

Pump 1 Solvent B: 2-propanol

Pump 1 Solvent C:

Pump 1 Solvent D:

Method Description:

Chrom Type: Fixed WL Chromatogram, 250 nm

Peak Quantitation: AREA

Calculation Method: AREA%

No.	RT	Area	Conc 1	BC
1	13.540	5464428	100.000	MC
		5464428	100.000	

Peak rejection level: 0

CSM: WXH Series: 0307 Report Name: modified System: Sys 1

Chromaster System Manager Report

Analyzed Date and Time: 2021/08/19 14:38

Reported Date and Time: 2021/08/19 15:44:31

Processed Date and Time: 2021/08/19 15:44

Data Path: C:\WIN32APP\CHROMASTER\WXH\DATA\0307\

Processing Method: IB_UV

System (acquisition): Sys 1
Application(data): WXH
Sample Name: WXH-133-IB-IPA 0%
Injection from this vial: 1 of 1
Sample Description:

Series: 0307
Vial Number: 12
Vial Type: UNK
Volume: 10.0 ul

Chrom Type: Fixed WL Chromatogram, 250 nm

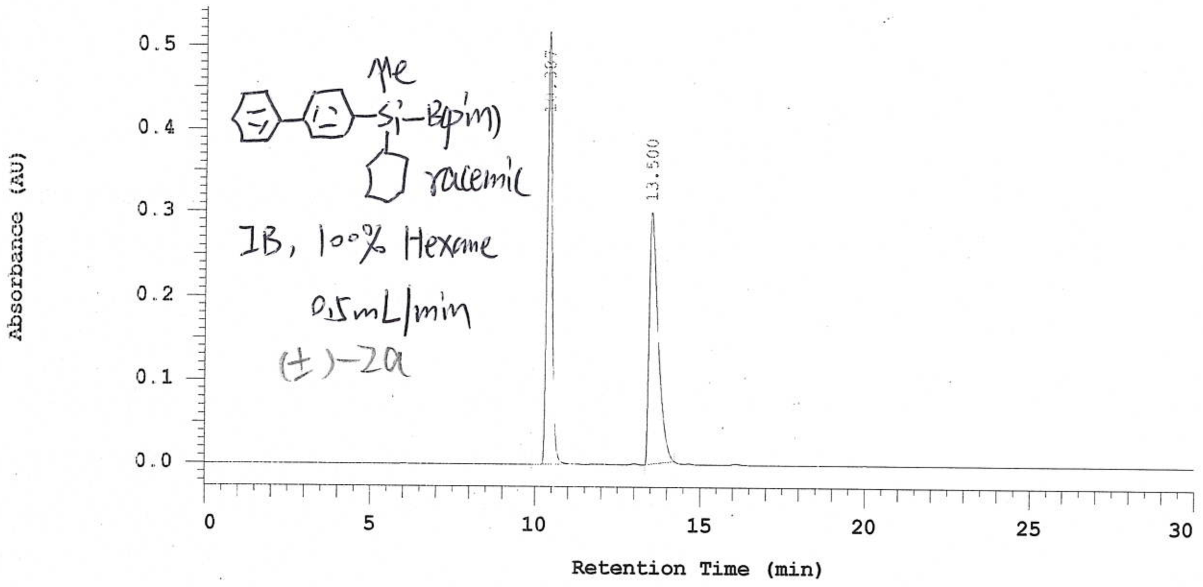

Processing Method: IB_UV
Method Developer:
Pump 1: 5110
Pump 1 Solvent A: hexane
Pump 1 Solvent B: 2-propanol
Pump 1 Solvent C:
Pump 1 Solvent D:
Method Description:

Chrom Type: Fixed WL Chromatogram, 250 nm
Peak Quantitation: AREA
Calculation Method: AREA%

No.	RT	Area	Conc 1	BC
1	10.387	2711520	49.157	MC
2	13.500	2804551	50.843	MC
		5516071	100.000	

Peak rejection level: 0

D-2000 Elite HPLC System Manager Report

Analyzed Date and Time: 2021/12/02 15:52
Reported Date and Time: 2021/12/02 16:30
Processed Date and Time: 2021/12/02 16:30
Data Path: C:\WIN32APP\D2000HSM\Isocratic\DATA\3789\
Processing Method: 0.0/100.0 iPrOH/Hexane
System (acquisition): Sys 1
Series: 3789
Application(data): Isocratic HPLC
Vial Number: 182
Sample Name: WXH-184-ODOD-0%
Vial Type: UNK
Injection from this vial: 1 of 1
Volume: 10.0 ul
Sample Description:

Chrom Type: HPLC Channel : 1

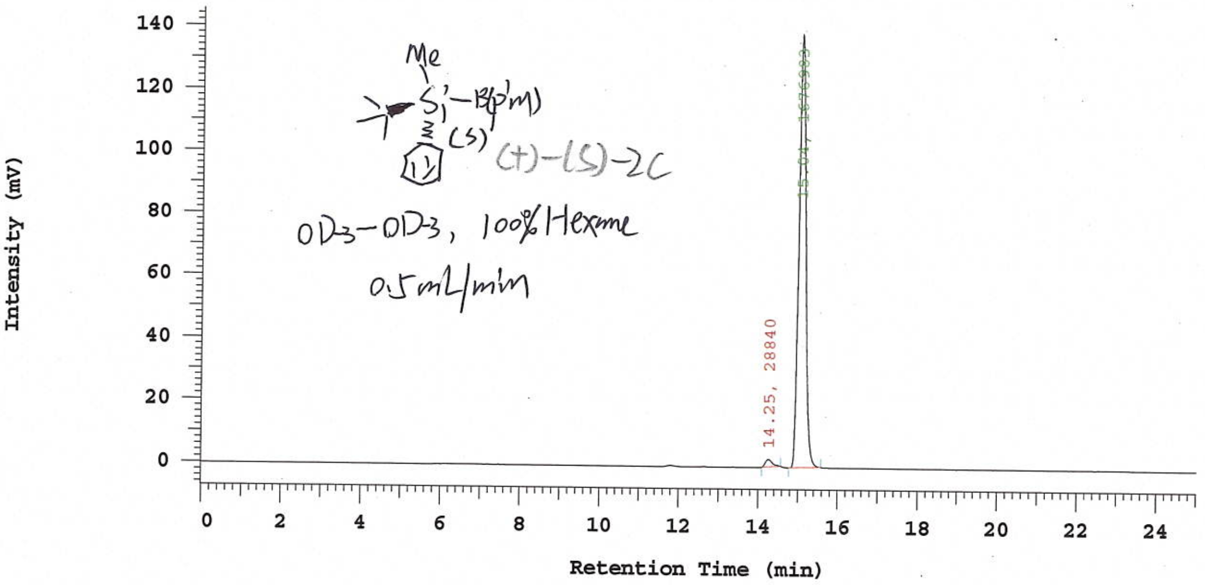

Processing Method: 0.0/100.0 iPrOH/Hexane
Column Type: OD-H 2
Method Developer: Administrator
Pump A: L-2130
Pump A Solvent A: Hexane
Pump A Solvent B: 10/90 iPrOH/Hexane
Pump A Solvent C: iPrOH
Pump A Solvent D: EtOH
Method Description:

Chrom Type: HPLC Channel : 1
Peak Quantitation: AREA
Calculation Method: AREA%

No.	RT	Area	Area %
1	14.25	28840	1.691
2	15.04	1676983	98.309
		1705823	100.000

Peak rejection level: 0

D-2000 Elite HPLC System Manager Report

Analyzed Date and Time: 2021/12/02 15:10

Reported Date and Time: 2021/12/02 16:15

Processed Date and Time: 2021/12/02 16:15

Data Path: C:\WIN32APP\D2000HSM\Isocratic\DATA\3788\

Processing Method: 0.0/100.0 iPrOH/Hexane

System (acquisition): Sys 1

Series: 3788

Application(data): Isocratic HPLC

Vial Number: 181

Sample Name: WXH-187-ODOD-0%

Vial Type: UNK

Injection from this vial: 1 of 1

Volume: 10.0 ul

Sample Description:

Chrom Type: HPLC Channel : 1

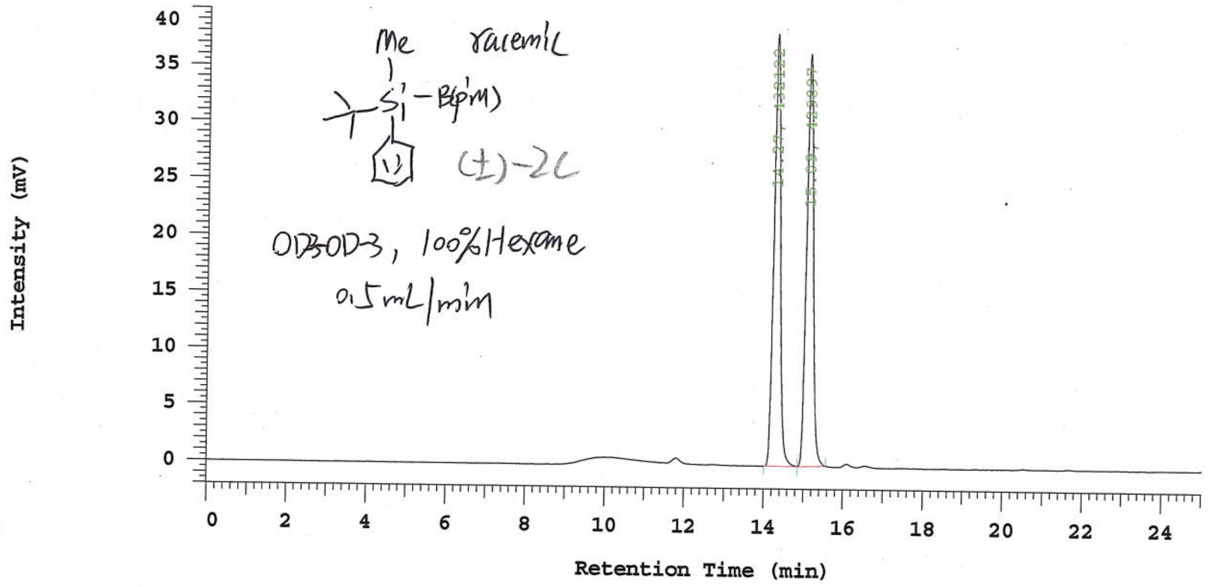

Processing Method: 0.0/100.0 iPrOH/Hexane

Column Type: OD-H 2

Method Developer: Administrator

Pump A: L-2130

Pump A Solvent A: Hexane

Pump A Solvent B: 10/90 iPrOH/Hexane

Pump A Solvent C: iPrOH

Pump A Solvent D: EtOH

Method Description:

Chrom Type: HPLC Channel : 1

Peak Quantitation: AREA

Calculation Method: AREA%

No.	RT	Area	Area %
1	14.27	432122	50.129
2	15.09	429897	49.871
		862019	100.000

Peak rejection level: 0

D-2000: Isocratic HPLC Series: 3944 Report Name: modified System: Sys 1

D-2000 Elite HPLC System Manager Report

Analyzed Date and Time: 2022/07/02 17:53
Reported Date and Time: 2022/07/04 08:50
Processed Date and Time: 2022/07/04 08:50
Data Path: C:\WIN32APP\D2000HSM\Isocratic\DATA\3944\
Processing Method: 0.0/100.0 iPrOH/Hexane

System (acquisition): Sys 1
Series: 3944
Application(data): Isocratic HPLC
Vial Number: 181
Sample Name: wxh-252-OD-0%
Vial Type: UNK
Injection from this vial: 1 of 1
Volume: 10.0 ul
Sample Description:

Chrom Type: HPLC Channel : 1

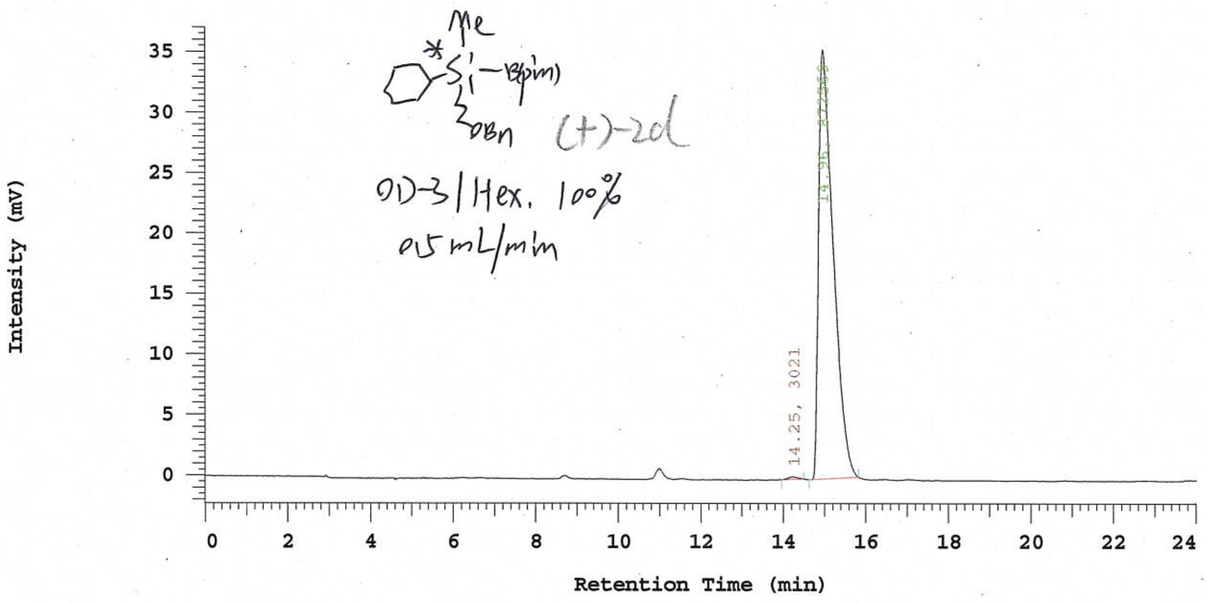

Processing Method: 0.0/100.0 iPrOH/Hexane
Column Type: OD-H 2
Method Developer: Administrator
Pump A: L-2130
Pump A Solvent A: Hexane
Pump A Solvent B: 10/90 iPrOH/Hexane
Pump A Solvent C: iPrOH
Pump A Solvent D: EtOH
Method Description:

Chrom Type: HPLC Channel : 1
Peak Quantitation: AREA
Calculation Method: AREA%

No.	RT	Area	Area %
1	14.25	3021	0.345
2	14.96	872569	99.655
		875590	100.000

Peak rejection level: 0

D-2000 Elite HPLC System Manager Report

Analyzed Date and Time: 2022/07/02 14:08
Reported Date and Time: 2022/07/04 08:49
Processed Date and Time: 2022/07/04 08:49
Data Path: C:\WIN32APP\D2000HSM\Isocratic\DATA\3942\
Processing Method: 0.0/100.0 iPrOH/Hexane

System (acquisition): Sys 1
Series: 3942
Application(data): Isocratic HPLC
Vial Number: 181
Sample Name: wxh-251-rac-OD-0%
Vial Type: UNK
Injection from this vial: 1 of 1
Volume: 10.0 ul
Sample Description:

Chrom Type: HPLC Channel : 1

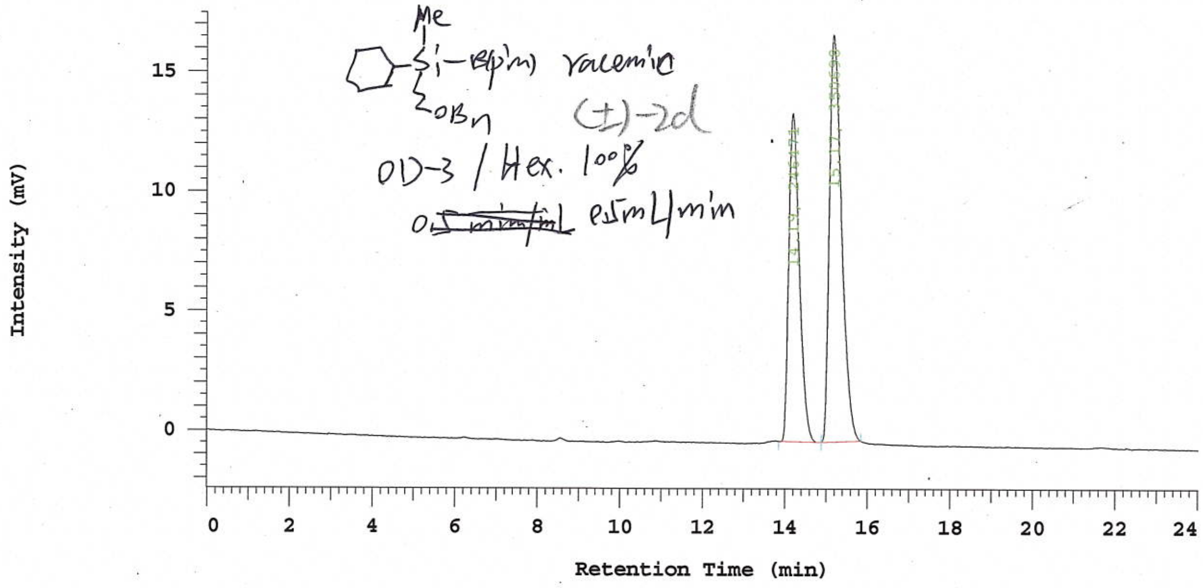

Processing Method: 0.0/100.0 iPrOH/Hexane
Column Type: OD-H 2
Method Developer: Administrator
Pump A: L-2130
Pump A Solvent A: Hexane
Pump A Solvent B: 10/90 iPrOH/Hexane
Pump A Solvent C: iPrOH
Pump A Solvent D: EtOH
Method Description:

Chrom Type: HPLC Channel : 1
Peak Quantitation: AREA
Calculation Method: AREA%

No.	RT	Area	Area %
1	14.19	246471	41.273
2	15.17	350698	58.727
		597169	100.000

Peak rejection level: 0

Chromaster System Manager Report

Analyzed Date and Time: 2021/09/01 18:54

Reported Date and Time: 2021/09/01 19:30:08

Processed Date and Time: 2021/09/01 19:29

Data Path: C:\WIN32APP\CHROMASTER\WXH\DATA\0326\

Processing Method: IB_UV

System (acquisition): Sys 1

Series: 0326

Application(data): WXH

Vial Number: 11

Sample Name: WXH-142-IB-IPA 0%

Vial Type: UNK

Injection from this vial: 1 of 1

Volume: 10.0 ul

Sample Description:

Chrom Type: Fixed WL Chromatogram, 250 nm

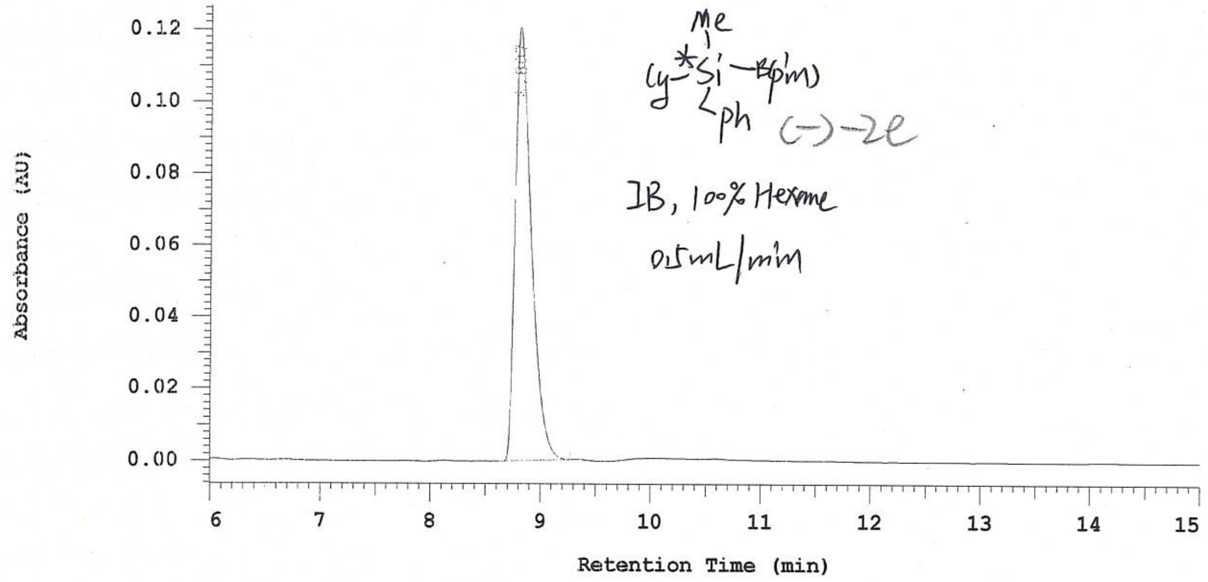

Processing Method: IB_UV

Method Developer:

Pump 1: 5110

Pump 1 Solvent A: hexane

Pump 1 Solvent B: 2-propanol

Pump 1 Solvent C:

Pump 1 Solvent D:

Method Description:

Chrom Type: Fixed WL Chromatogram, 250 nm

Peak Quantitation: AREA

Calculation Method: AREA%

No.	RT	Area	Conc 1	BC
1	8.807	623300	100.000	MC
		623300	100.000	

Peak rejection level: 0

Chromaster System Manager Report

Analyzed Date and Time: 2021/09/01 17:44

Reported Date and Time: 2021/09/01 19:36:01

Processed Date and Time: 2021/09/01 19:29

Data Path: C:\WIN32APP\CHROMASTER\WXH\DATA\0325\

Processing Method: IB_UV

System (acquisition): Sys 1
Application(data): WXH
Sample Name: WXH-104-IB-IPA 0%
Injection from this vial: 1 of 1
Sample Description:

Series: 0325
Vial Number: 11
Vial Type: UNK
Volume: 10.0 ul

Chrom Type: Fixed WL Chromatogram, 250 nm

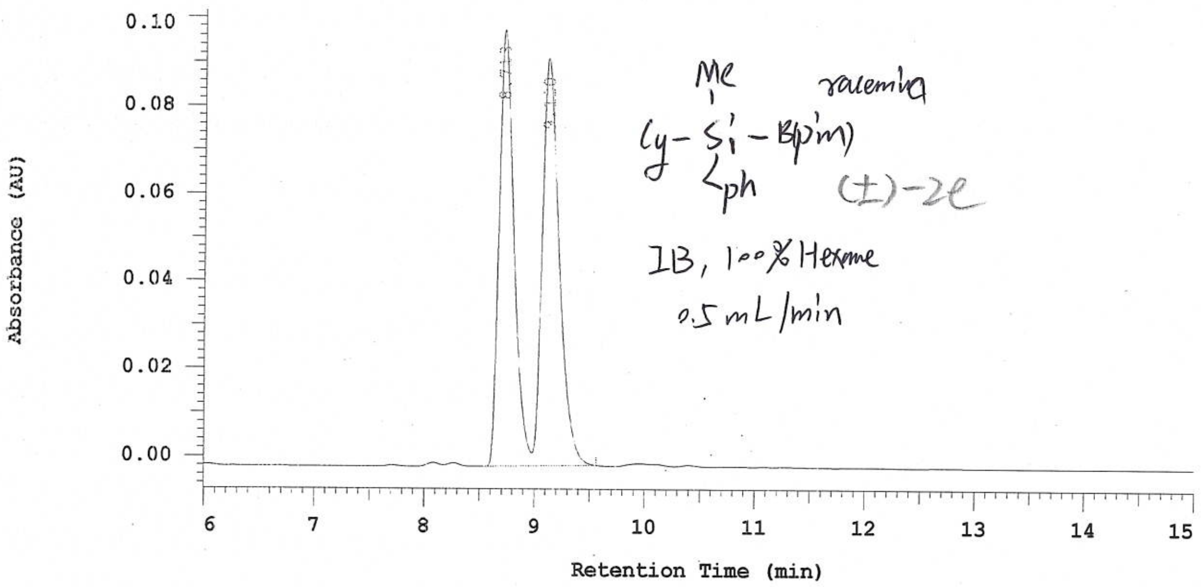

Processing Method: IB_UV
Method Developer:
Pump 1: 5110
Pump 1 Solvent A: hexane
Pump 1 Solvent B: 2-propanol
Pump 1 Solvent C:
Pump 1 Solvent D:
Method Description:

Chrom Type: Fixed WL Chromatogram, 250 nm
Peak Quantitation: AREA
Calculation Method: AREA%

No.	RT	Area	Conc 1	BC
1	8.713	473772	48.874	MC
2	9.120	495608	51.126	MC
		969380	100.000	

Peak rejection level: 0

D-2000 Elite HPLC System Manager Report

Analyzed Date and Time: 2021/08/19 19:13 Reported Date and Time: 2021/08/19 19:48

Processed Date and Time: 2021/08/19 19:48

Data Path: C:\WIN32APP\D2000HSM\Isocratic\DATA\3554\

Processing Method: 0.0/100.0 iPrOH/Hexane

System (acquisition): Sys 1 Series: 3554

Application(data): Isocratic HPLC Vial Number: 181

Sample Name: WXH-137-OD-0% Vial Type: UNK

Injection from this vial: 1 of 1 Volume: 10.0 ul

Sample Description:

Chrom Type: HPLC Channel : 1

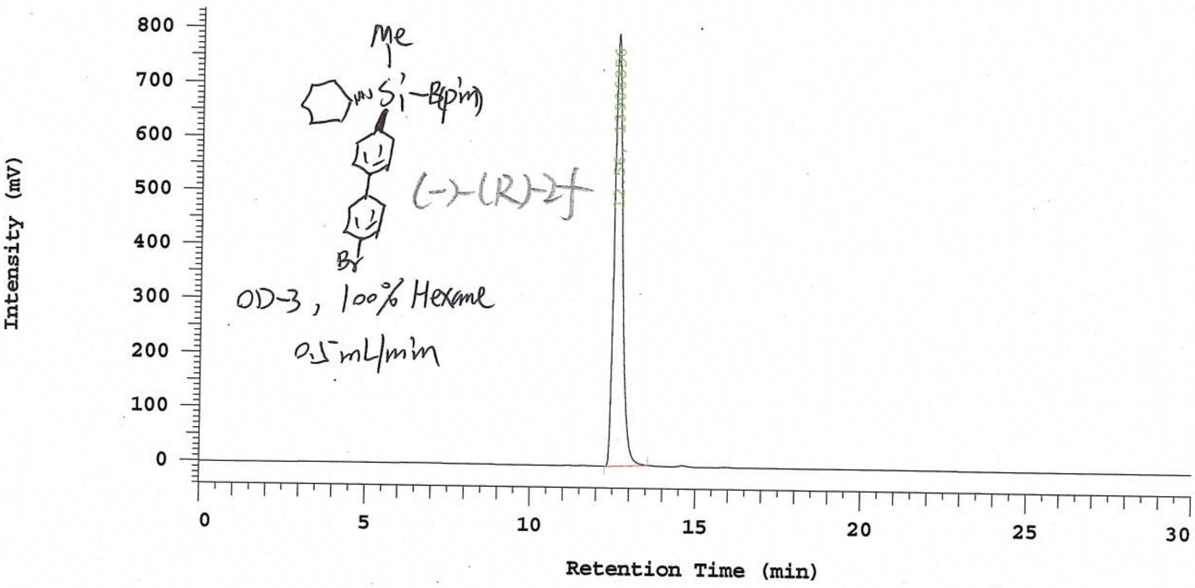

Processing Method: 0.0/100.0 iPrOH/Hexane

Column Type: OD-H 2 Method Developer: Administrator

Pump A: L-2130

Pump A Solvent A: Hexane

Pump A Solvent B: 10/90 iPrOH/Hexane

Pump A Solvent C: iPrOH

Pump A Solvent D: EtOH

Method Description:

Chrom Type: HPLC Channel : 1

Peak Quantitation: AREA

Calculation Method: AREA%

No.	RT	Area	Area %
1	12.56	13906856	100.000
		13906856	100.000

Peak rejection level: 0

D-2000: Isocratic HPLC Series: 3553 Report Name: modified System: Sys 1

D-2000 Elite HPLC System Manager Report

Analyzed Date and Time: 2021/08/19 17:36
Reported Date and Time: 2021/08/19 19:03
Processed Date and Time: 2021/08/19 19:02
Data Path: C:\WIN32APP\D2000HSM\Isocratic\DATA\3553\
Processing Method: 0.0/100.0 iPrOH/Hexane

System (acquisition): Sys 1
Series: 3553
Application(data): Isocratic HPLC
Vial Number: 181
Sample Name: WXH-130-OD-0%
Vial Type: UNK
Injection from this vial: 1 of 1
Volume: 10.0 ul
Sample Description:

Chrom Type: HPLC Channel : 1

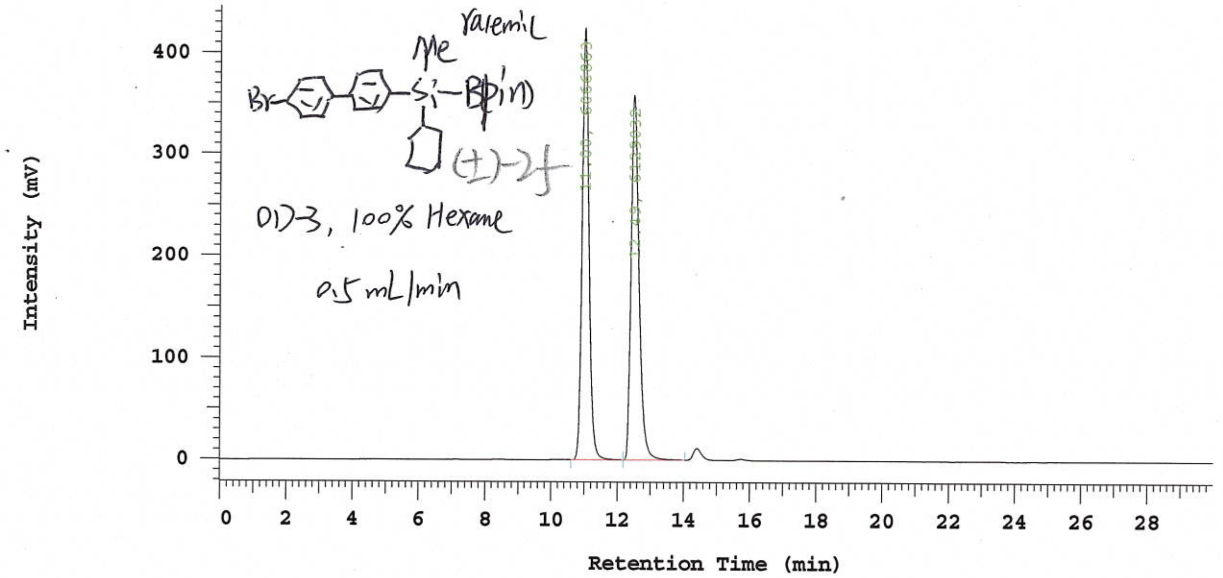

Processing Method: 0.0/100.0 iPrOH/Hexane
Column Type: OD-H 2
Method Developer: Administrator
Pump A: L-2130
Pump A Solvent A: Hexane
Pump A Solvent B: 10/90 iPrOH/Hexane
Pump A Solvent C: iPrOH
Pump A Solvent D: EtOH
Method Description:

Chrom Type: HPLC Channel : 1
Peak Quantitation: AREA
Calculation Method: AREA%

No.	RT	Area	Area %
1	11.00	6066863	49.745
2	12.49	6129032	50.255
		12195895	100.000

Peak rejection level: 0

D-2000 Elite HPLC System Manager Report

Analyzed Date and Time: 2022/10/26 18:21

Reported Date and Time: 2022/10/28 17:33

Processed Date and Time: 2022/10/28 17:33

Data Path: C:\WIN32APP\D2000HSM\Isocratic\DATA\4078\

Processing Method: 0.0/100.0 iPrOH/Hexane

System (acquisition): Sys 1

Series: 4078

Application(data): Isocratic HPLC

Vial Number: 181

Sample Name: wxh-332-0% IPA-OZ

Vial Type: UNK

Injection from this vial: 1 of 1

Volume: 10.0 ul

Sample Description:

Chrom Type: HPLC Channel : 1

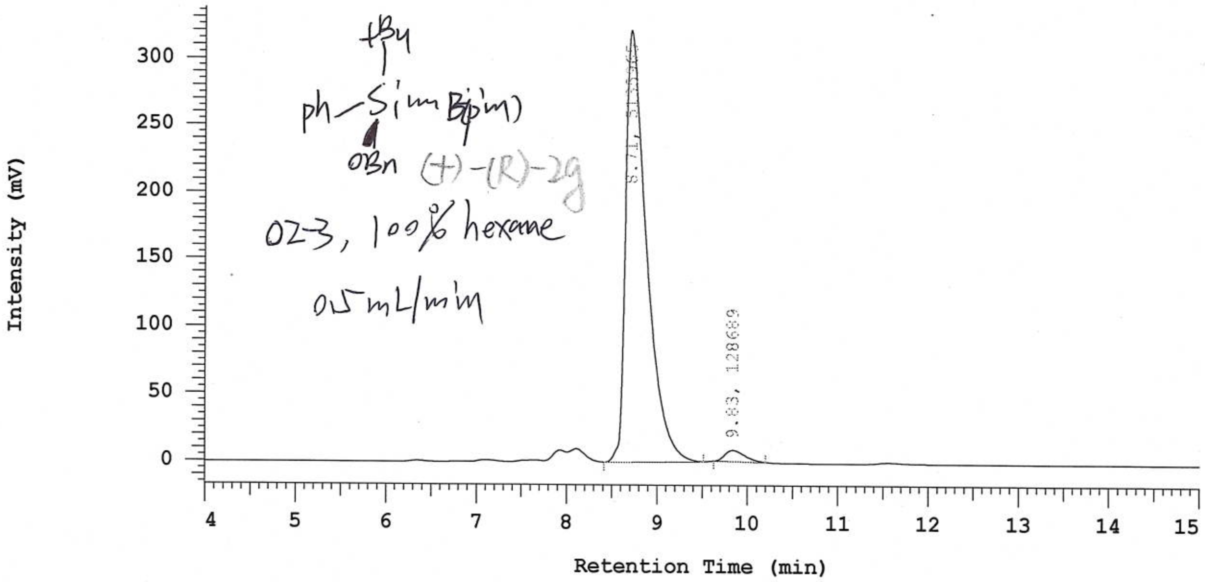

Processing Method: 0.0/100.0 iPrOH/Hexane

Column Type: OD-H 2

Method Developer: Administrator

Pump A: L-2130

Pump A Solvent A: Hexane

Pump A Solvent B: 10/90 iPrOH/Hexane

Pump A Solvent C: iPrOH

Pump A Solvent D: EtOH

Method Description:

Chrom Type: HPLC Channel : 1

Peak Quantitation: AREA

Calculation Method: AREA%

No.	RT	Area	Area %
1	8.71	5135965	97.556
2	9.83	128689	2.444
		5264654	100.000

Peak rejection level: 0

D-2000 Elite HPLC System Manager Report

Analyzed Date and Time: 2022/10/26 16:00
Reported Date and Time: 2022/10/28 17:32
Processed Date and Time: 2022/10/28 17:32
Data Path: C:\WIN32APP\D2000HSM\Isocratic\DATA\4077\
Processing Method: 0.0/100.0 iPrOH/Hexane

System (acquisition): Sys 1
Series: 4077
Application(data): Isocratic HPLC
Vial Number: 181
Sample Name: wxh-302-race-0% IPA-OZ
Vial Type: UNK
Injection from this vial: 1 of 1
Volume: 10.0 ul
Sample Description:

Chrom Type: HPLC Channel : 1

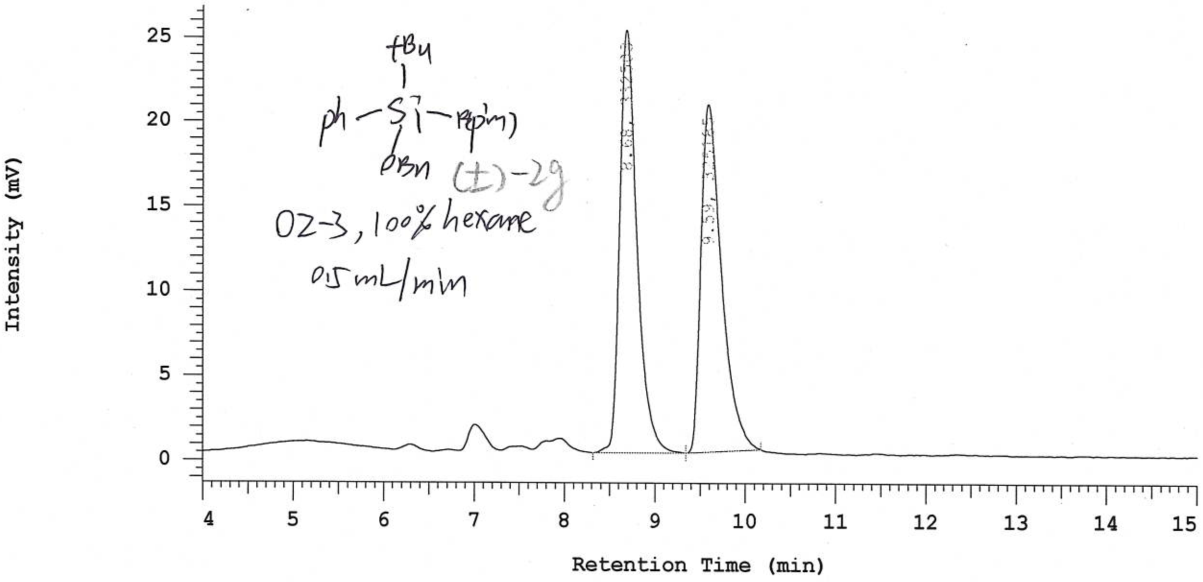

Processing Method: 0.0/100.0 iPrOH/Hexane
Column Type: OD-H 2
Method Developer: Administrator
Pump A: L-2130
Pump A Solvent A: Hexane
Pump A Solvent B: 10/90 iPrOH/Hexane
Pump A Solvent C: iPrOH
Pump A Solvent D: EtOH
Method Description:

Chrom Type: HPLC Channel : 1
Peak Quantitation: AREA
Calculation Method: AREA%

No.	RT	Area	Area %
1	8.68	332503	50.556
2	9.59	325185	49.444
		657688	100.000

Peak rejection level: 0

CSM: WXH Series: 0502 Report Name: modified System: Sys 1

Chromaster System Manager Report

Analyzed Date and Time: 2023/02/16 23:05

Reported Date and Time: 2023/02/17 09:44:30

Processed Date and Time: 2023/02/17 09:44

Data Path: C:\WIN32APP\CHROMASTER\WXH\DATA\0502\

Processing Method: IB_UV

System (acquisition): Sys 1

Application(data): WXH

Sample Name: wxh-407-IB-0%IPA

Injection from this vial: 1 of 1

Sample Description:

Series: 0502

Vial Number: 12

Vial Type: UNK

Volume: 10.0 ul

Chrom Type: Fixed WL Chromatogram, 268 nm

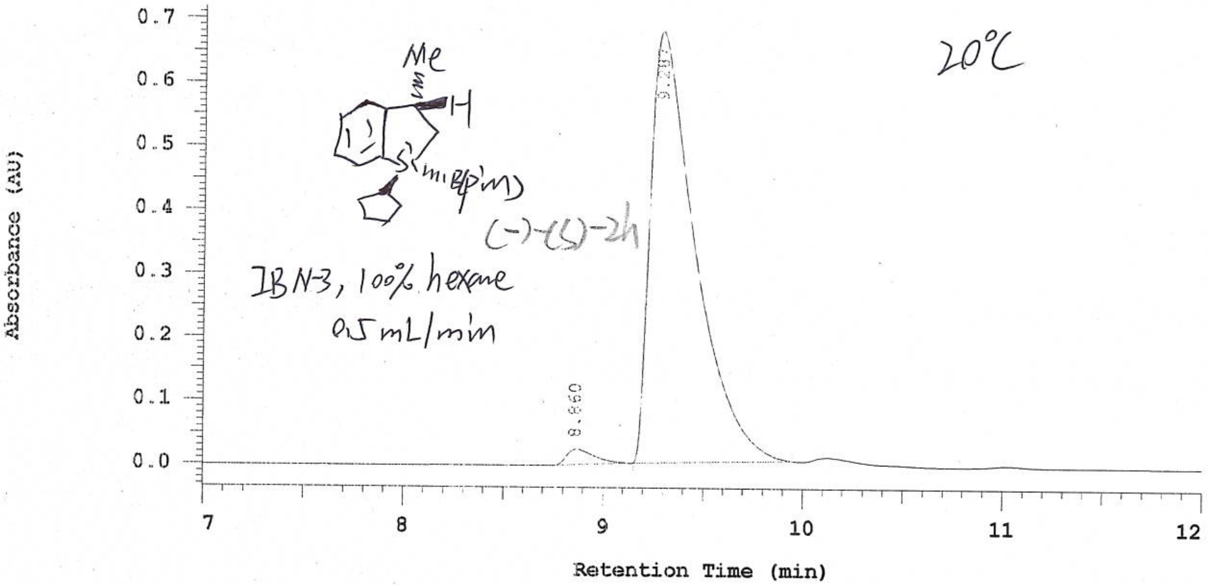

Processing Method: IB_UV

Method Developer:

Pump 1: 5110

Pump 1 Solvent A: hexane

Pump 1 Solvent B: 2-propanol

Pump 1 Solvent C:

Pump 1 Solvent D:

Method Description:

Chrom Type: Fixed WL Chromatogram, 268 nm

Peak Quantitation: AREA

Calculation Method: AREA%

No.	RT	Area	Conc 1	BC
1	8.860	112749	2.081	MC
2	9.287	5305772	97.919	MC
		5418521	100.000	

Peak rejection level: 0

CSM: WXH Series: 0501 Report Name: modified System: Sys 1

Chromaster System Manager Report

Analyzed Date and Time: 2023/02/16 22:03 Reported Date and Time: 2023/06/26 14:55:34

Processed Date and Time: 2023/06/26 14:55

Data Path: C:\WIN32APP\CHROMASTER\WXH\DATA\0501\

Processing Method: column1(IA-3)_0.3%

System (acquisition): Sys 1 Series: 0501
Application(data): WXH Vial Number: 11
Sample Name: wxh-411-IB-0%IPA Vial Type: UNK
Injection from this vial: 1 of 1 Volume: 10.0 ul
Sample Description:

Chrom Type: Fixed WL Chromatogram, 268 nm

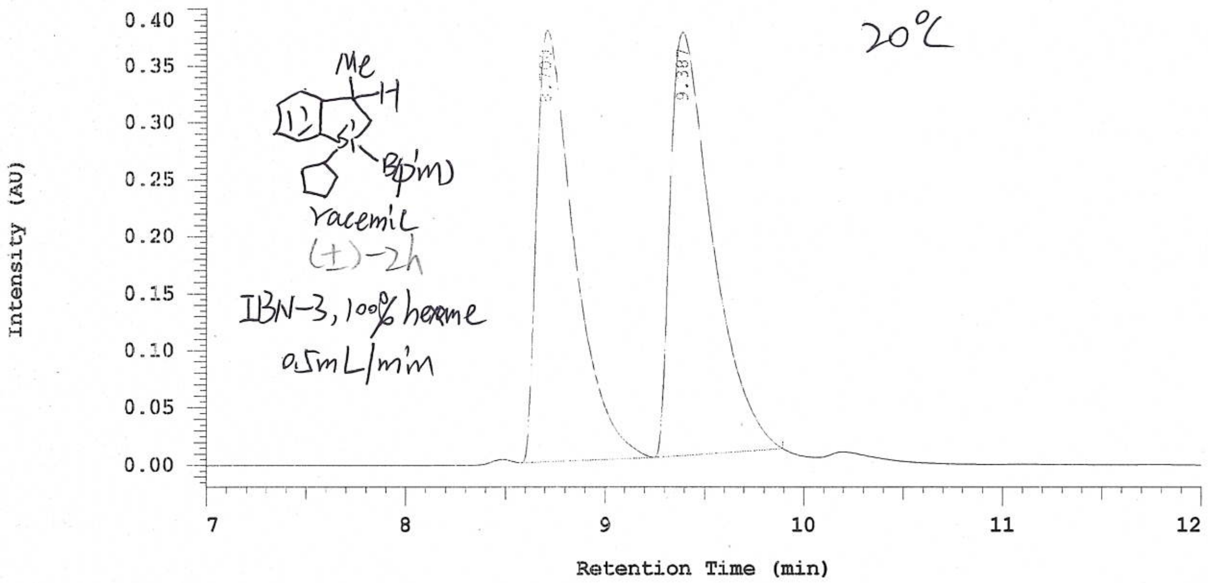

Processing Method: column1(IA-3)_0.3%
Method Developer:
Pump 1: 5110
Pump 1 Solvent A: hexane Pump 1 Solvent B: 2-propanol
Pump 1 Solvent C: Pump 1 Solvent D:
Method Description:

Chrom Type: Fixed WL Chromatogram, 268 nm
Peak Quantitation: AREA
Calculation Method: AREA%

No.	RT	Area	Conc 1	BC
1	8.700	2476646	47.601	MC
2	9.387	2726303	52.399	MC
		5202949	100.000	

Peak rejection level: 0

D-2000 Elite HPLC System Manager Report

Analyzed Date and Time: 2021/08/19 16:42
Reported Date and Time: 2021/08/19 17:26
Processed Date and Time: 2021/08/19 17:26
Data Path: C:\WIN32APP\D2000HSM\Isocratic\DATA\3552\
Processing Method: 0.0/100.0 iPrOH/Hexane
System (acquisition): Sys 1
Series: 3552
Application(data): Isocratic HPLC
Vial Number: 182
Sample Name: WXH-139-OD-0%
Vial Type: UNK
Injection from this vial: 1 of 1
Volume: 10.0 ul
Sample Description:

Chrom Type: HPLC Channel : 1

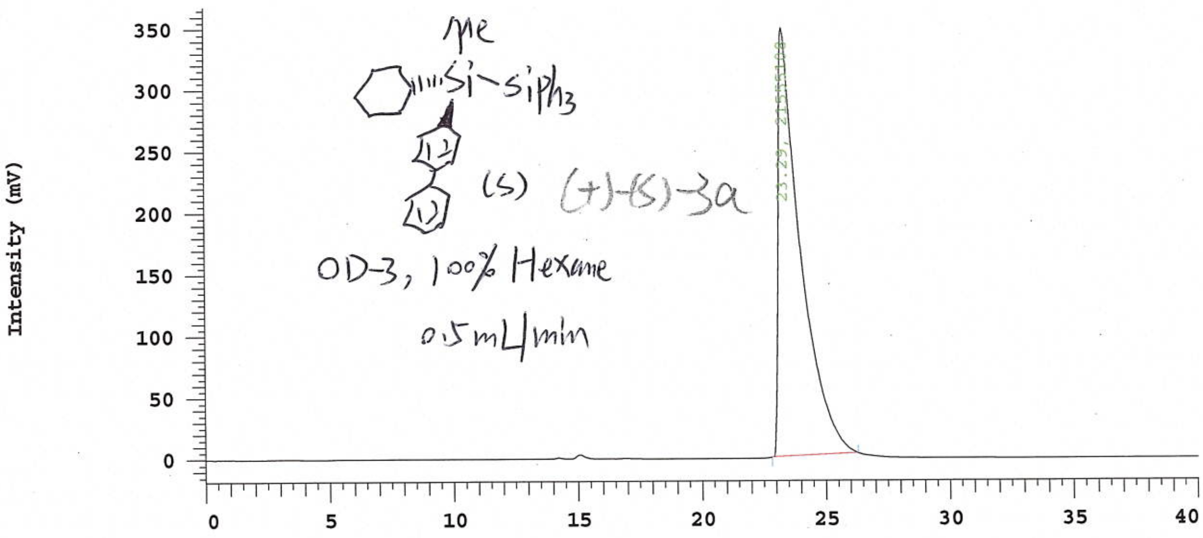

Processing Method: 0.0/100.0 iPrOH/Hexane
Column Type: OD-H 2
Method Developer: Administrator
Pump A: L-2130
Pump A Solvent A: Hexane
Pump A Solvent B: 10/90 iPrOH/Hexane
Pump A Solvent C: iPrOH
Pump A Solvent D: EtOH
Method Description:

Chrom Type: HPLC Channel : 1
Peak Quantitation: AREA
Calculation Method: AREA%

No.	RT	Area	Area %
1	23.29	21555108	100.000
		21555108	100.000

Peak rejection level: 0

D-2000 Elite HPLC System Manager Report

Analyzed Date and Time: 2021/08/19 16:01
Reported Date and Time: 2021/08/19 16:47
Processed Date and Time: 2021/08/19 16:47
Data Path: C:\WIN32APP\D2000HSM\Isocratic\DATA\3551\
Processing Method: 0.0/100.0 iPrOH/Hexane

System (acquisition): Sys 1
Series: 3551
Application(data): Isocratic HPLC
Vial Number: 181
Sample Name: WXH-136-OD-0%
Vial Type: UNK
Injection from this vial: 1 of 1
Volume: 10.0 ul
Sample Description:

Chrom Type: HPLC Channel : 1

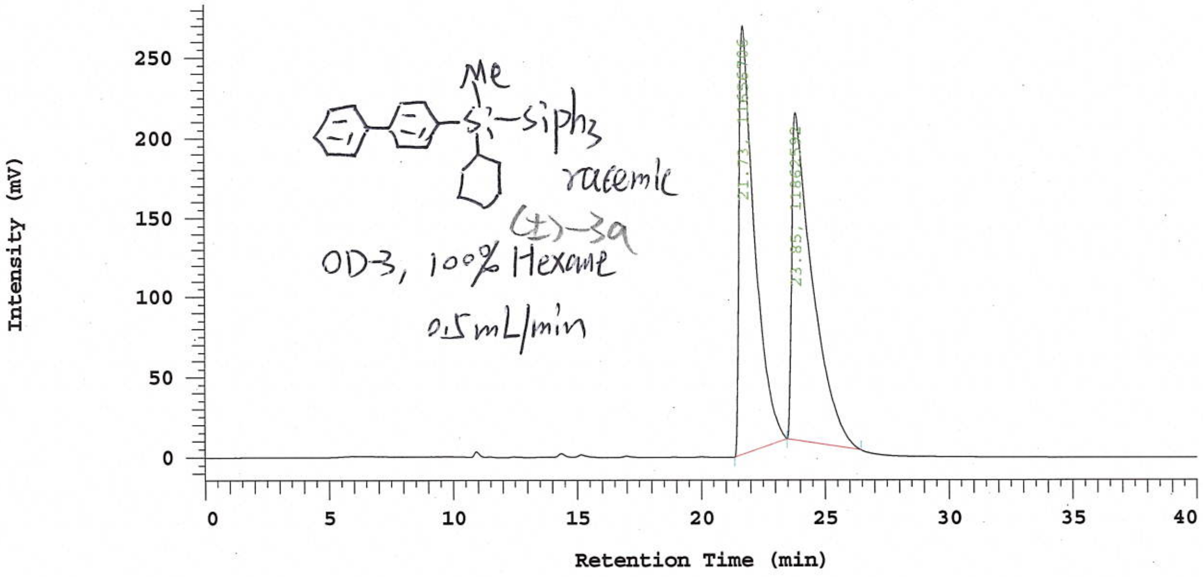

Processing Method: 0.0/100.0 iPrOH/Hexane
Column Type: OD-H 2
Method Developer: Administrator
Pump A: L-2130
Pump A Solvent A: Hexane
Pump A Solvent B: 10/90 iPrOH/Hexane
Pump A Solvent C: iPrOH
Pump A Solvent D: EtOH
Method Description:

Chrom Type: HPLC Channel : 1
Peak Quantitation: AREA
Calculation Method: AREA%

No.	RT	Area	Area %
1	21.73	11656706	49.562
2	23.85	11862592	50.438
		23519298	100.000

Peak rejection level: 0

D-2000: Isocratic HPLC Series: 3882 Report Name: modified System: Sys 1

D-2000 Elite HPLC System Manager Report

Analyzed Date and Time: 2022/02/22 11:12
Reported Date and Time: 2022/02/22 16:51
Processed Date and Time: 2022/02/22 16:51
Data Path: C:\WIN32APP\D2000HSM\Isocratic\DATA\3882\
Processing Method: 0.0/100.0 iPrOH/Hexane

System (acquisition): Sys 1
Application(data): Isocratic HPLC
Sample Name: WXH-228-OD-0%
Injection from this vial: 1 of 1
Sample Description:

Series: 3882
Vial Number: 182
Vial Type: UNK
Volume: 10.0 ul

Chrom Type: HPLC Channel : 1

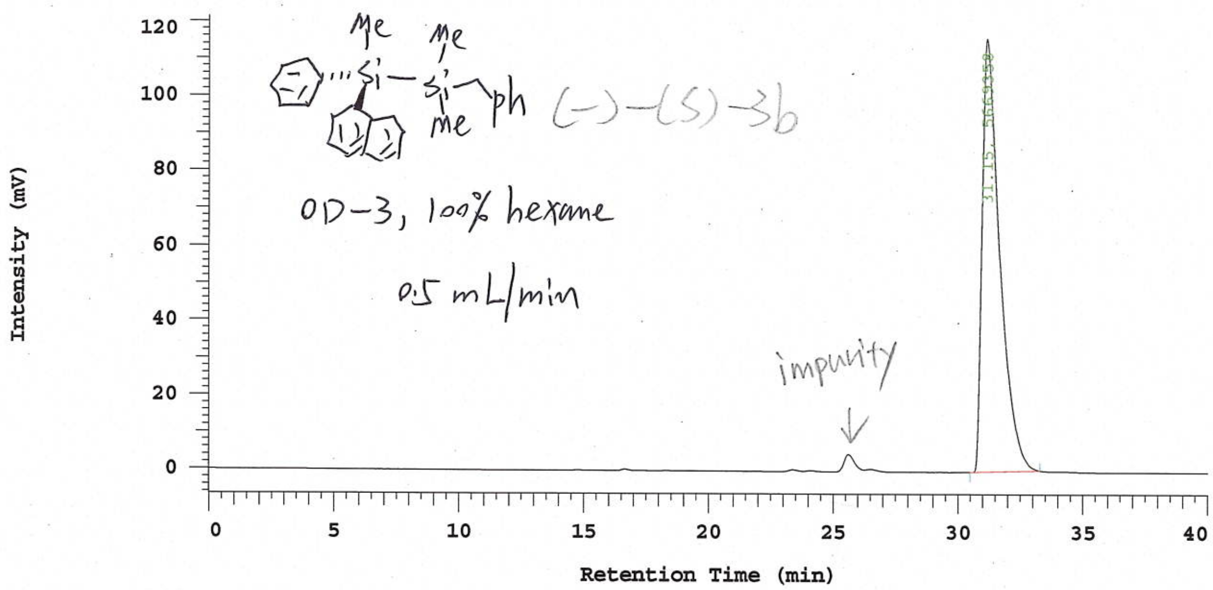

Processing Method: 0.0/100.0 iPrOH/Hexane
Column Type: OD-H 2
Method Developer: Administrator
Pump A: L-2130
Pump A Solvent A: Hexane
Pump A Solvent B: 10/90 iPrOH/Hexane
Pump A Solvent C: iPrOH
Pump A Solvent D: EtOH
Method Description:

Chrom Type: HPLC Channel : 1
Peak Quantitation: AREA
Calculation Method: AREA%

No.	RT	Area	Area %
1	31.15	5669358	100.000
		5669358	100.000

Peak rejection level: 0

D-2000 Elite HPLC System Manager Report

Analyzed Date and Time: 2022/02/22 15:47
Reported Date and Time: 2022/02/22 16:48
Processed Date and Time: 2022/02/22 16:48
Data Path: C:\WIN32APP\D2000HSM\Isocratic\DATA\3884\
Processing Method: 0.0/100.0 iPrOH/Hexane

System (acquisition): Sys 1
Series: 3884
Application(data): Isocratic HPLC
Vial Number: 181
Sample Name: WXH-167-race-OD-0%
Vial Type: UNK
Injection from this vial: 1 of 1
Volume: 10.0 ul
Sample Description:

Chrom Type: HPLC Channel : 1

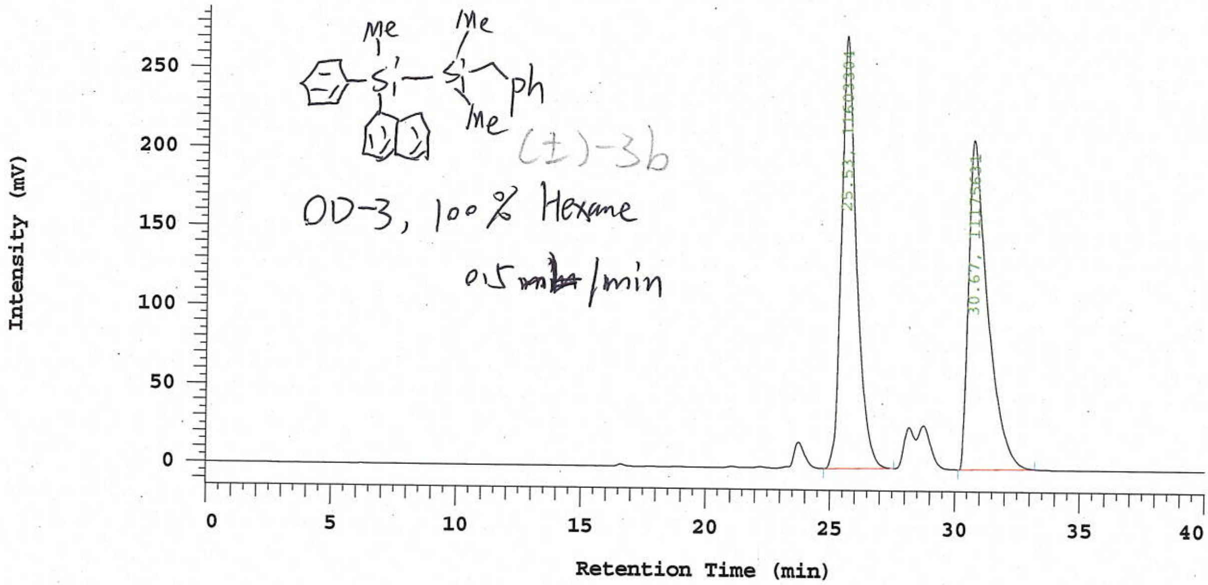

Processing Method: 0.0/100.0 iPrOH/Hexane
Column Type: OD-H 2
Method Developer: Administrator
Pump A: L-2130
Pump A Solvent A: Hexane
Pump A Solvent B: 10/90 iPrOH/Hexane
Pump A Solvent C: iPrOH
Pump A Solvent D: EtOH
Method Description:

Chrom Type: HPLC Channel : 1
Peak Quantitation: AREA
Calculation Method: AREA%

No.	RT	Area	Area %
1	25.53	11603304	50.939
2	30.67	11175631	49.061
		22778935	100.000

Peak rejection level: 0

D-2000 Elite HPLC System Manager Report

Analyzed Date and Time: 2021/12/03 17:32
Reported Date and Time: 2021/12/03 18:22
Processed Date and Time: 2021/12/03 18:22
Data Path: C:\WIN32APP\D2000HSM\Isocratic\DATA\3793\
Processing Method: 0.0/100.0 iPrOH/Hexane

System (acquisition): Sys 1
Series: 3793
Application(data): Isocratic HPLC
Vial Number: 182
Sample Name: WXH-189-OD3-0%
Vial Type: UNK
Injection from this vial: 1 of 1
Volume: 10.0 ul
Sample Description:

Chrom Type: HPLC Channel : 1

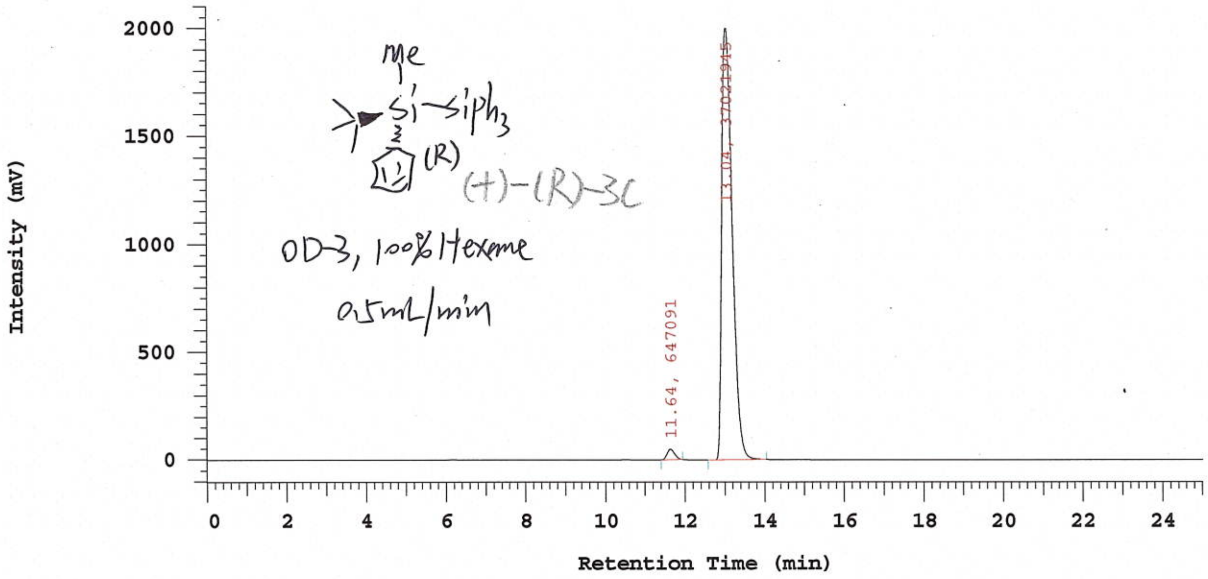

Processing Method: 0.0/100.0 iPrOH/Hexane
Column Type: OD-H 2
Method Developer: Administrator
Pump A: L-2130
Pump A Solvent A: Hexane
Pump A Solvent B: 10/90 iPrOH/Hexane
Pump A Solvent C: iPrOH
Pump A Solvent D: EtOH
Method Description:

Chrom Type: HPLC Channel : 1
Peak Quantitation: AREA
Calculation Method: AREA%

No.	RT	Area	Area %
1	11.64	647091	1.718
2	13.04	37021945	98.282
		37669036	100.000

Peak rejection level: 0

D-2000 Elite HPLC System Manager Report

Analyzed Date and Time: 2021/12/03 16:50
Reported Date and Time: 2021/12/03 18:24
Processed Date and Time: 2021/12/03 18:24
Data Path: C:\WIN32APP\D2000HSM\Isocratic\DATA\3792\
Processing Method: 0.0/100.0 iPrOH/Hexane

System (acquisition): Sys 1
Series: 3792
Application(data): Isocratic HPLC
Vial Number: 181
Sample Name: WXH-169-OD3-0%
Vial Type: UNK
Injection from this vial: 1 of 1
Volume: 10.0 ul
Sample Description:

Chrom Type: HPLC Channel : 1

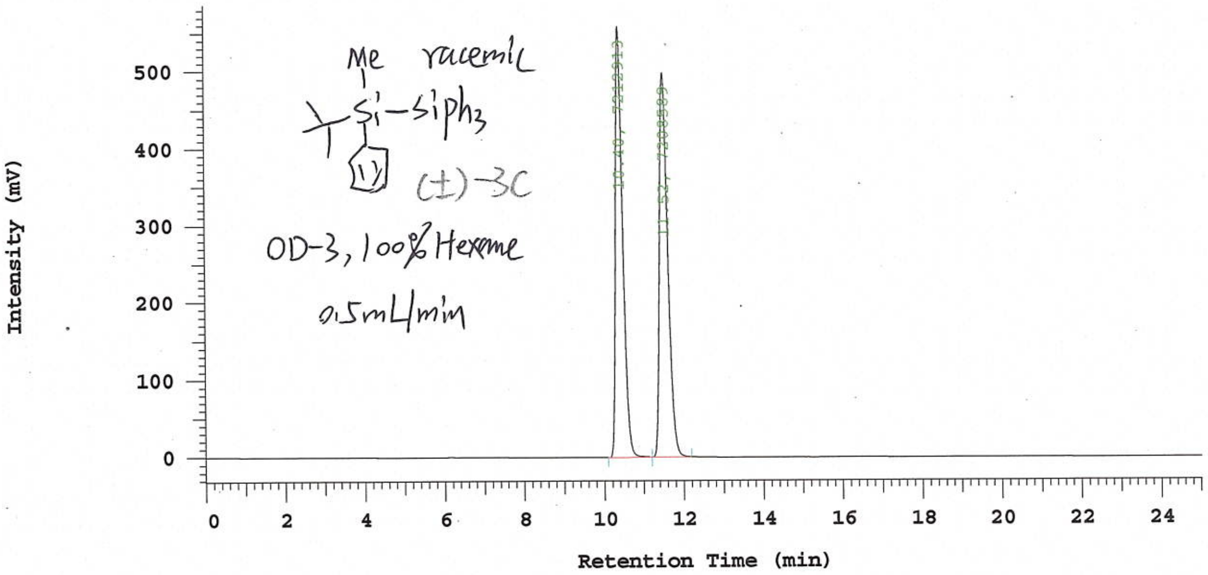

Processing Method: 0.0/100.0 iPrOH/Hexane
Column Type: OD-H 2
Method Developer: Administrator
Pump A: L-2130
Pump A Solvent A: Hexane
Pump A Solvent B: 10/90 iPrOH/Hexane
Pump A Solvent C: iPrOH
Pump A Solvent D: EtOH
Method Description:

Chrom Type: HPLC Channel : 1
Peak Quantitation: AREA
Calculation Method: AREA%

No.	RT	Area	Area %
1	10.40	7212913	50.015
2	11.52	7208589	49.985
		14421502	100.000

Peak rejection level: 0

Chromaster System Manager Report

Analyzed Date and Time: 2022/04/04 11:45
Reported Date and Time: 2022/04/04 14:04:47
Processed Date and Time: 2022/04/04 14:04
Data Path: C:\WIN32APP\CHROMASTER\WXH\DATA\0413\
Processing Method: IB_UV
System (acquisition): Sys 1
Series: 0413
Application(data): WXH
Vial Number: 12
Sample Name: WXH-253-IB-IPA 0%
Vial Type: UNK
Injection from this vial: 1 of 1
Volume: 10.0 ul
Sample Description:

Chrom Type: Fixed WL Chromatogram, 250 nm

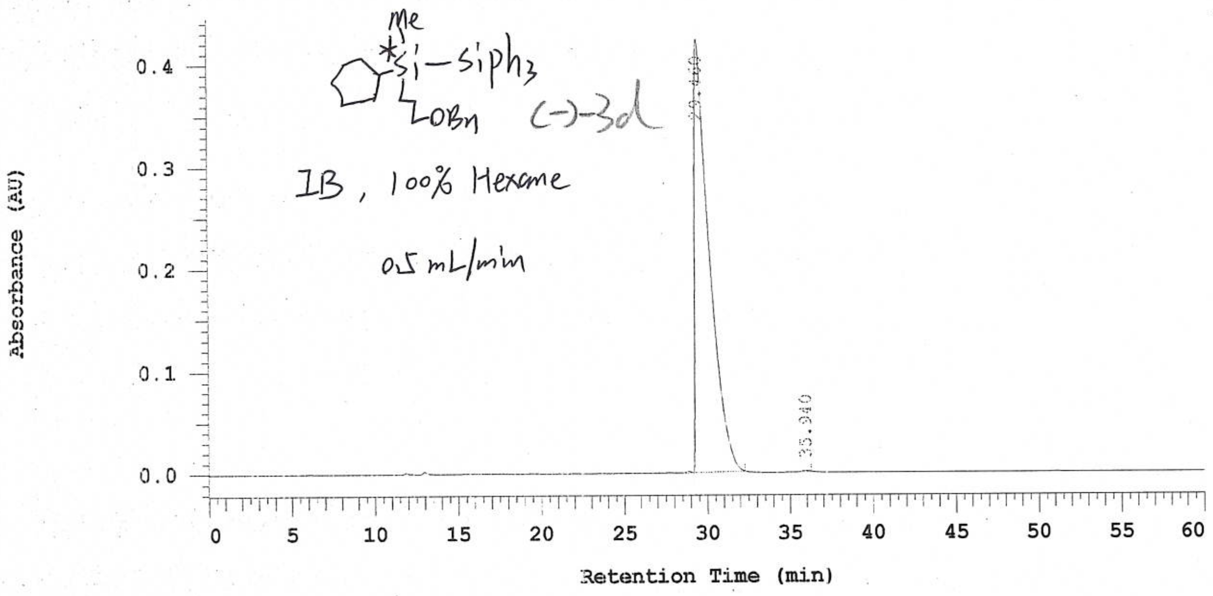

Processing Method: IB_UV
Method Developer:
Pump 1: 5110
Pump 1 Solvent A: hexane
Pump 1 Solvent B: 2-propanol
Pump 1 Solvent C:
Pump 1 Solvent D:
Method Description:

Chrom Type: Fixed WL Chromatogram, 250 nm
Peak Quantitation: AREA
Calculation Method: AREA%

No.	RT	Area	Conc 1	BC
1	29.460	12738625	99.933	BB
2	35.940	8604	0.067	BB
		12747229	100.000	

Peak rejection level: 0

Chromaster System Manager Report

Analyzed Date and Time: 2022/04/04 10:14
Reported Date and Time: 2022/04/04 14:03:14

Processed Date and Time: 2022/04/04 14:03

Data Path: C:\WIN32APP\CHROMASTER\WXH\DATA\0412\

Processing Method: IB_UV

System (acquisition): Sys 1
Application(data): WXH
Sample Name: WXH-062-IB-IPA 0%
Injection from this vial: 1 of 1
Sample Description:

Series: 0412
Vial Number: 11
Vial Type: UNK
Volume: 10.0 ul

Chrom Type: Fixed WL Chromatogram, 250 nm

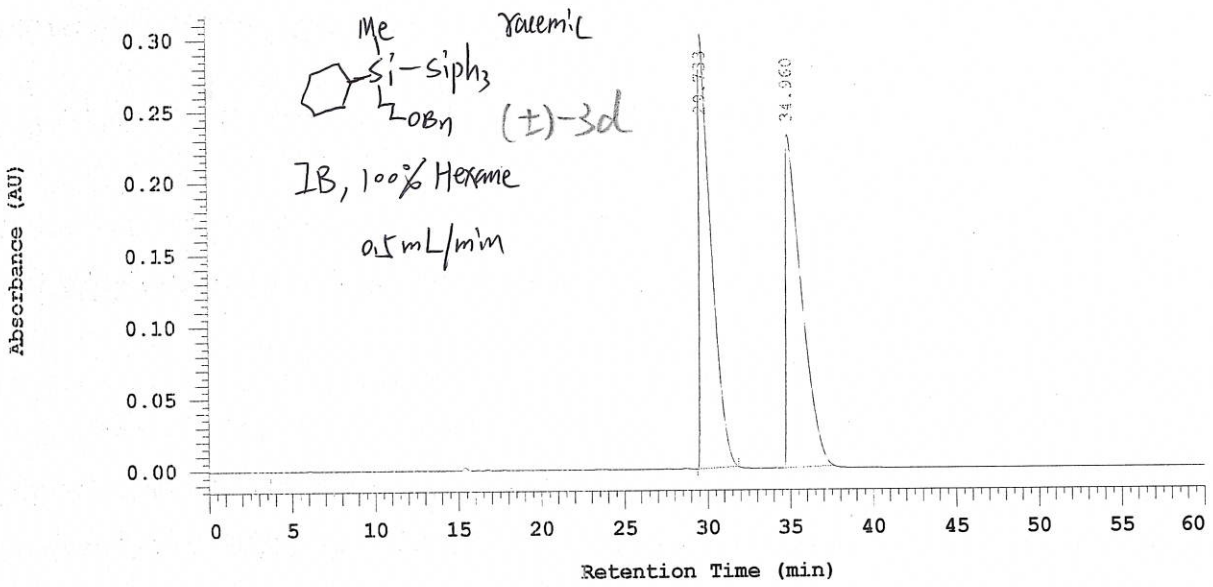

Processing Method: IB_UV
Method Developer:
Pump 1: 5110
Pump 1 Solvent A: hexane
Pump 1 Solvent B: 2-propanol
Pump 1 Solvent C:
Pump 1 Solvent D:
Method Description:

Chrom Type: Fixed WL Chromatogram, 250 nm

Peak Quantitation: AREA
Calculation Method: AREA%

No.	RT	Area	Conc 1	BC
1	29.733	7506940	50.247	BB
2	34.960	7433171	49.753	BB
		14940111	100.000	

Peak rejection level: 0

D-2000 Elite HPLC System Manager Report

Analyzed Date and Time: 2021/06/17 11:39

Reported Date and Time: 2022/04/06 15:34

Processed Date and Time: 2022/04/06 15:34

Data Path: C:\WIN32APP\D2000HSM\Isocratic\DATA\3398\

Processing Method: 05/95 iPrOH/Hexane

System (acquisition): Sys 1

Application(data): Isocratic HPLC

Sample Name: WXH-108-ODOD-0%

Injection from this vial: 1 of 1

Sample Description:

Series: 3398

Vial Number: 181

Vial Type: UNK

Volume: 1.0 ul

Chrom Type: HPLC Channel : 1

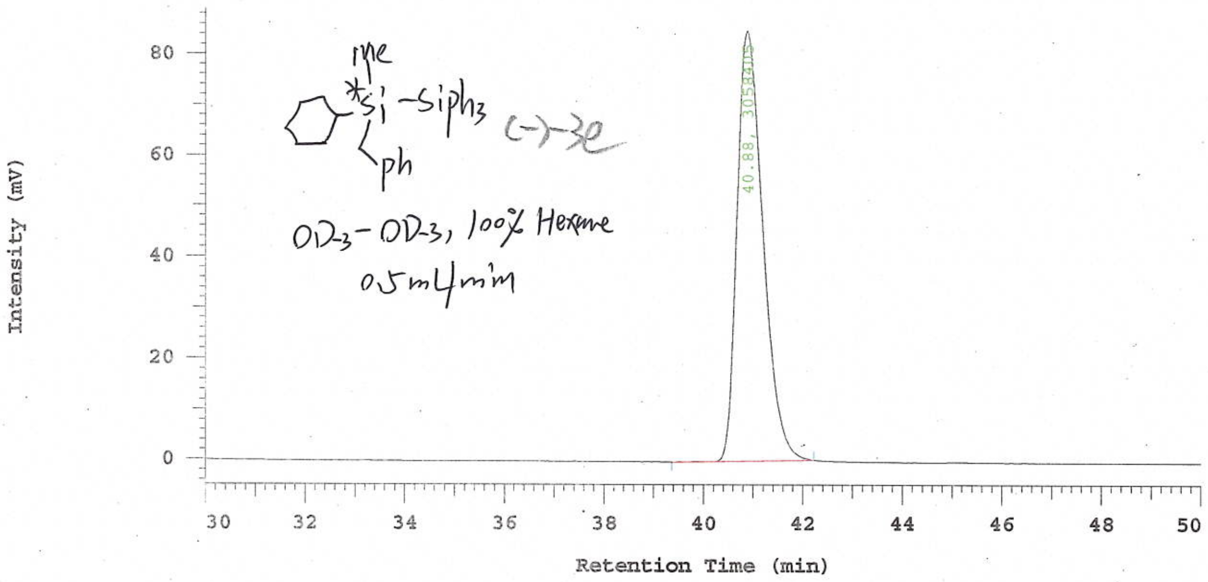

Processing Method: 05/95 iPrOH/Hexane

Column Type: OD-H 2

Method Developer: Administrator

Pump A: L-2130

Pump A Solvent A: Hexane

Pump A Solvent B: 10/90 iPrOH/Hexane

Pump A Solvent C: iPrOH

Pump A Solvent D: EtOH

Method Description:

Chrom Type: HPLC Channel : 1

Peak Quantitation: AREA

Calculation Method: AREA%

No.	RT	Area	Area %
1	40.88	3058405	100.000
		3058405	100.000

Peak rejection level: 0

D-2000 Elite HPLC System Manager Report

Analyzed Date and Time: 2021/06/17 12:41
Reported Date and Time: 2022/04/06 15:33
Processed Date and Time: 2022/04/06 15:33
Data Path: C:\WIN32APP\D2000HSM\Isocratic\DATA\3399\
Processing Method: 05/95 iPrOH/Hexane

System (acquisition): Sys 1
Series: 3399
Application(data): Isocratic HPLC
Vial Number: 182
Sample Name: WXH-085-ODOD-0%
Vial Type: UNK
Injection from this vial: 1 of 1
Volume: 1.0 ul
Sample Description:

Chrom Type: HPLC Channel : 1

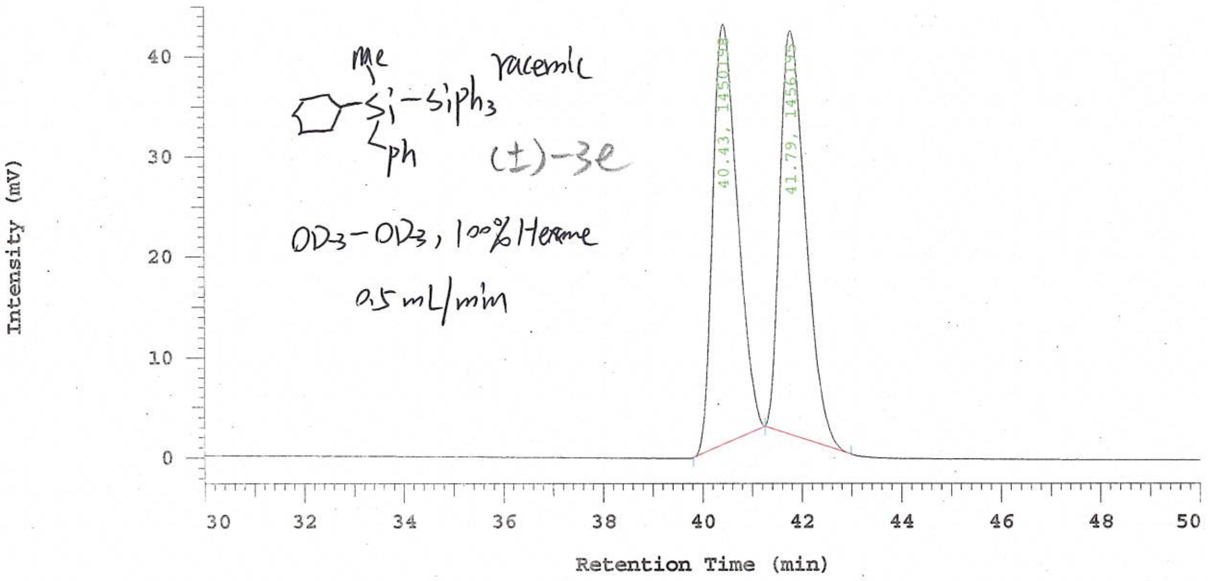

Processing Method: 05/95 iPrOH/Hexane
Column Type: OD-H 2
Method Developer: Administrator
Pump A: L-2130
Pump A Solvent A: Hexane
Pump A Solvent B: 10/90 iPrOH/Hexane
Pump A Solvent C: iPrOH
Pump A Solvent D: EtOH
Method Description:

Chrom Type: HPLC Channel : 1
Peak Quantitation: AREA
Calculation Method: AREA%

No.	RT	Area	Area %
1	40.43	1450198	49.897
2	41.79	1456195	50.103
		2906393	100.000

Peak rejection level: 0

Chromaster System Manager Report

Analyzed Date and Time: 2021/09/01 16:05

Reported Date and Time: 2022/04/06 16:05:24

Processed Date and Time: 2022/04/06 16:05

Data Path: C:\WIN32APP\CHROMASTER\WXH\DATA\0324\

Processing Method: ID_UV

System (acquisition): Sys 1

Application(data): WXH

Sample Name: WXH-146-IB-IPA 0%

Injection from this vial: 1 of 1

Sample Description:

Series: 0324

Vial Number: 12

Vial Type: UNK

Volume: 10.0 ul

Chrom Type: Fixed WL Chromatogram, 250 nm

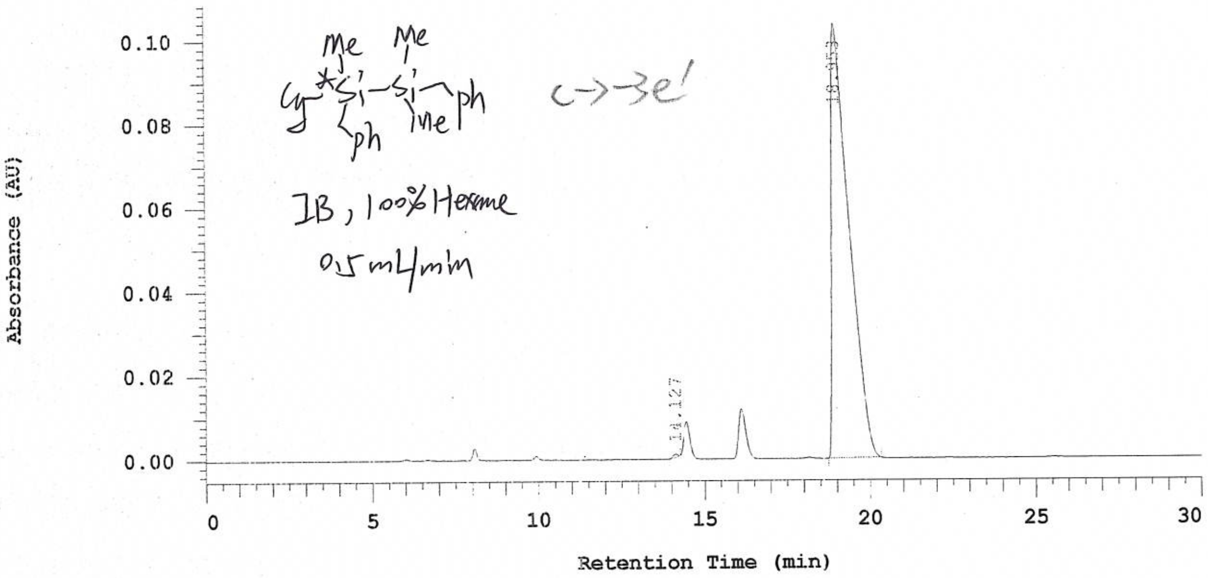

Processing Method: ID_UV

Method Developer:

Pump 1: 5110

Pump 1 Solvent A: hexane

Pump 1 Solvent B: 2-propanol

Pump 1 Solvent C:

Pump 1 Solvent D:

Method Description:

Chrom Type: Fixed WL Chromatogram, 250 nm

Peak Quantitation: AREA

Calculation Method: AREA%

No.	RT	Area	Conc 1	BC
1	14.127	3199	0.168	MC
2	18.953	1898966	99.832	BB
		1902165	100.000	

Peak rejection level: 0

Chromaster System Manager Report

Analyzed Date and Time: 2021/09/01 15:04

Reported Date and Time: 2021/09/01 19:36:22

Processed Date and Time: 2021/09/01 17:07

Data Path: C:\WIN32APP\CHROMASTER\WXH\DATA\0323\

Processing Method: IB_UV

System (acquisition): Sys 1

Application(data): WXH

Sample Name: WXH-105-IB-IPA 0%

Injection from this vial: 1 of 1

Sample Description:

Series: 0323

Vial Number: 11

Vial Type: UNK

Volume: 10.0 ul

Chrom Type: Fixed WL Chromatogram, 250 nm

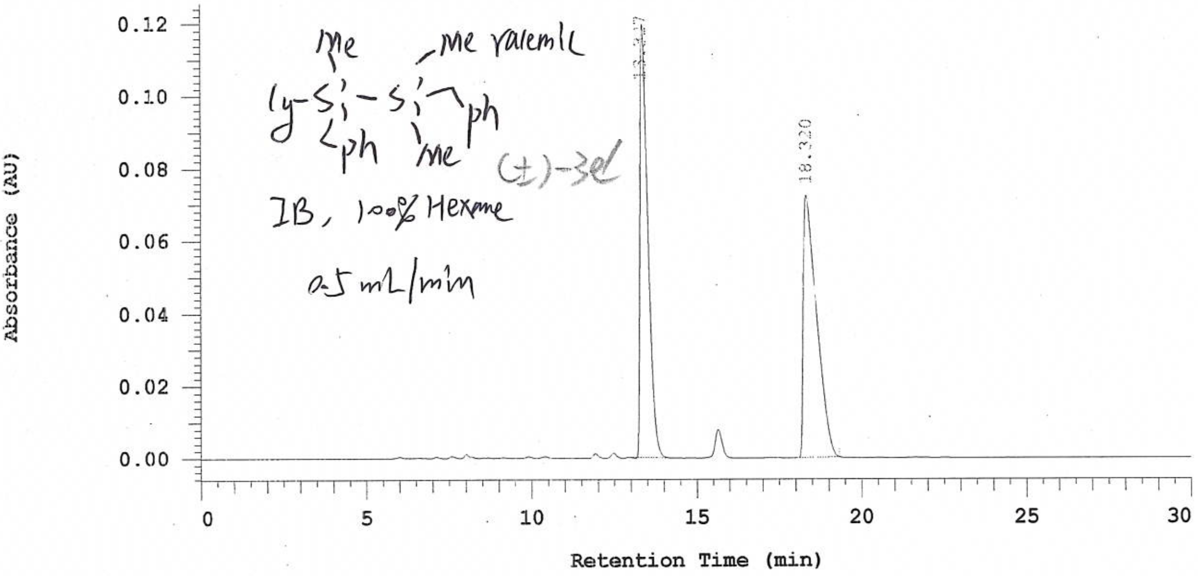

Processing Method: IB_UV

Method Developer:

Pump 1: 5110

Pump 1 Solvent A: hexane

Pump 1 Solvent B: 2-propanol

Pump 1 Solvent C:

Pump 1 Solvent D:

Method Description:

Chrom Type: Fixed WL Chromatogram, 250 nm

Peak Quantitation: AREA

Calculation Method: AREA%

No.	RT	Area	Conc 1	BC
1	13.327	970420	48.982	MC
2	18.320	1010773	51.018	BB
		1981193	100.000	

Peak rejection level: 0

D-2000 Elite HPLC System Manager Report

Analyzed Date and Time: 2022/10/28 16:36
Reported Date and Time: 2022/10/28 17:20
Processed Date and Time: 2022/10/28 17:19
Data Path: C:\WIN32APP\D2000HSM\Isocratic\DATA\4082\
Processing Method: 0.0/100.0 iPrOH/Hexane

System (acquisition): Sys 1
Series: 4082
Application(data): Isocratic HPLC
Vial Number: 181
Sample Name: wxh-335-0% IPA-ODOD
Vial Type: UNK
Injection from this vial: 1 of 1
Volume: 10.0 ul
Sample Description:

Chrom Type: HPLC Channel : 1

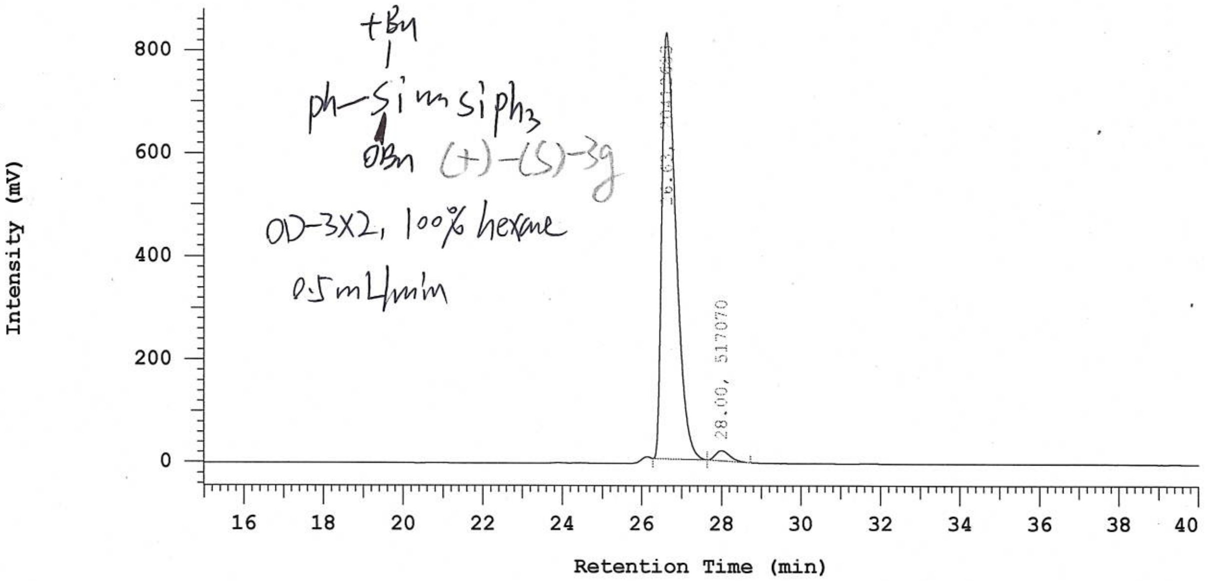

Processing Method: 0.0/100.0 iPrOH/Hexane
Column Type: OD-H 2
Method Developer: Administrator
Pump A: L-2130
Pump A Solvent A: Hexane
Pump A Solvent B: 10/90 iPrOH/Hexane
Pump A Solvent C: iPrOH
Pump A Solvent D: EtOH
Method Description:

Chrom Type: HPLC Channel : 1
Peak Quantitation: AREA
Calculation Method: AREA%

No.	RT	Area	Area %
1	26.63	20412683	97.529
2	28.00	517070	2.471
		20929753	100.000

Peak rejection level: 0

D-2000 Elite HPLC System Manager Report

Analyzed Date and Time: 2022/10/28 11:35
Reported Date and Time: 2022/10/28 17:21
Processed Date and Time: 2022/10/28 17:21
Data Path: C:\WIN32APP\D2000HSM\Isocratic\DATA\4081\
Processing Method: 0.0/100.0 iPrOH/Hexane

System (acquisition): Sys 1
Series: 4081
Application(data): Isocratic HPLC
Vial Number: 181
Sample Name: wxh-323-race-0% IPA-ODOD
Vial Type: UNK
Injection from this vial: 1 of 1
Volume: 10.0 ul
Sample Description:

Chrom Type: HPLC Channel : 1

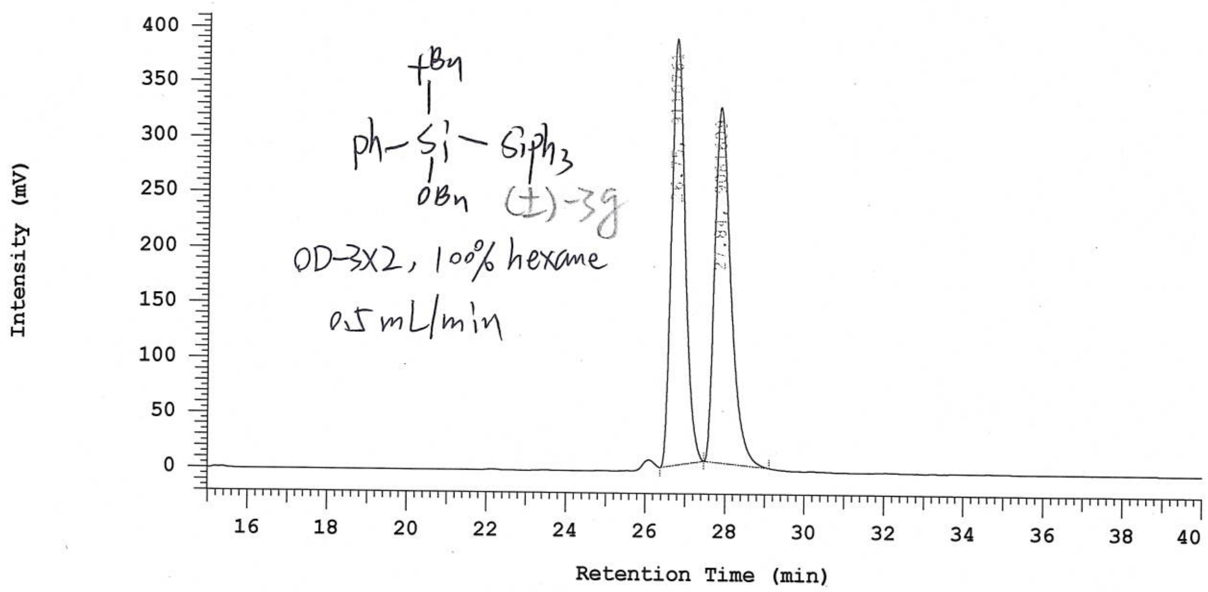

Processing Method: 0.0/100.0 iPrOH/Hexane
Column Type: OD-H 2
Method Developer: Administrator
Pump A: L-2130
Pump A Solvent A: Hexane
Pump A Solvent B: 10/90 iPrOH/Hexane
Pump A Solvent C: iPrOH
Pump A Solvent D: EtOH
Method Description:

Chrom Type: HPLC Channel : 1
Peak Quantitation: AREA
Calculation Method: AREA%

No.	RT	Area	Area %
1	26.75	9110761	50.080
2	27.84	9081609	49.920
		18192370	100.000

Peak rejection level: 0

D-2000 Elite HPLC System Manager Report

Analyzed Date and Time: 2022/03/17 21:22
Reported Date and Time: 2022/03/18 09:57
Processed Date and Time: 2022/03/18 09:57
Data Path: C:\WIN32APP\D2000HSM\Isocratic\DATA\3897\
Processing Method: 0.0/100.0 iPrOH/Hexane

System (acquisition): Sys 1
Series: 3897
Application(data): Isocratic HPLC
Vial Number: 182
Sample Name: WXH-248-OD-0%
Vial Type: UNK
Injection from this vial: 1 of 1
Volume: 10.0 ul
Sample Description:

Chrom Type: HPLC Channel : 1

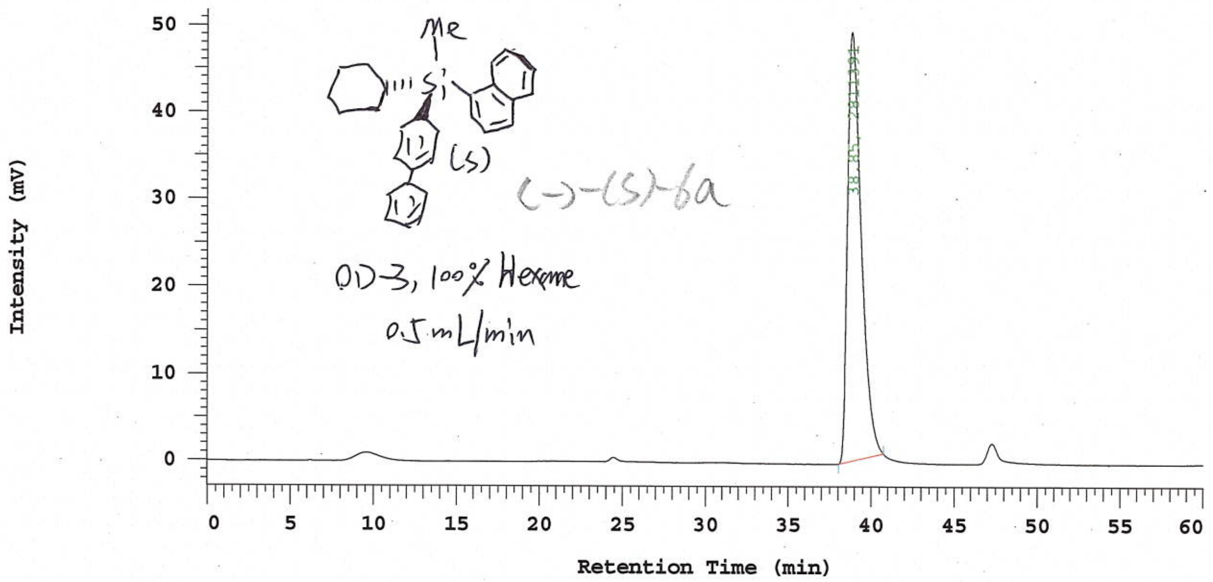

Processing Method: 0.0/100.0 iPrOH/Hexane
Column Type: OD-H 2
Method Developer: Administrator
Pump A: L-2130
Pump A Solvent A: Hexane
Pump A Solvent B: 10/90 iPrOH/Hexane
Pump A Solvent C: iPrOH
Pump A Solvent D: EtOH
Method Description:

Chrom Type: HPLC Channel : 1
Peak Quantitation: AREA
Calculation Method: AREA%

No.	RT	Area	Area %
1	38.85	2811391	100.000
		2811391	100.000

Peak rejection level: 0

D-2000 Elite HPLC System Manager Report

Analyzed Date and Time: 2022/03/17 20:01
Reported Date and Time: 2022/03/18 09:57
Processed Date and Time: 2022/03/18 09:56
Data Path: C:\WIN32APP\D2000HSM\Isocratic\DATA\3896\
Processing Method: 0.0/100.0 iPrOH/Hexane
System (acquisition): Sys 1
Application(data): Isocratic HPLC
Sample Name: WXH-238-OD-0%
Injection from this vial: 1 of 1
Sample Description:
Series: 3896
Vial Number: 181
Vial Type: UNK
Volume: 10.0 ul

Chrom Type: HPLC Channel : 1

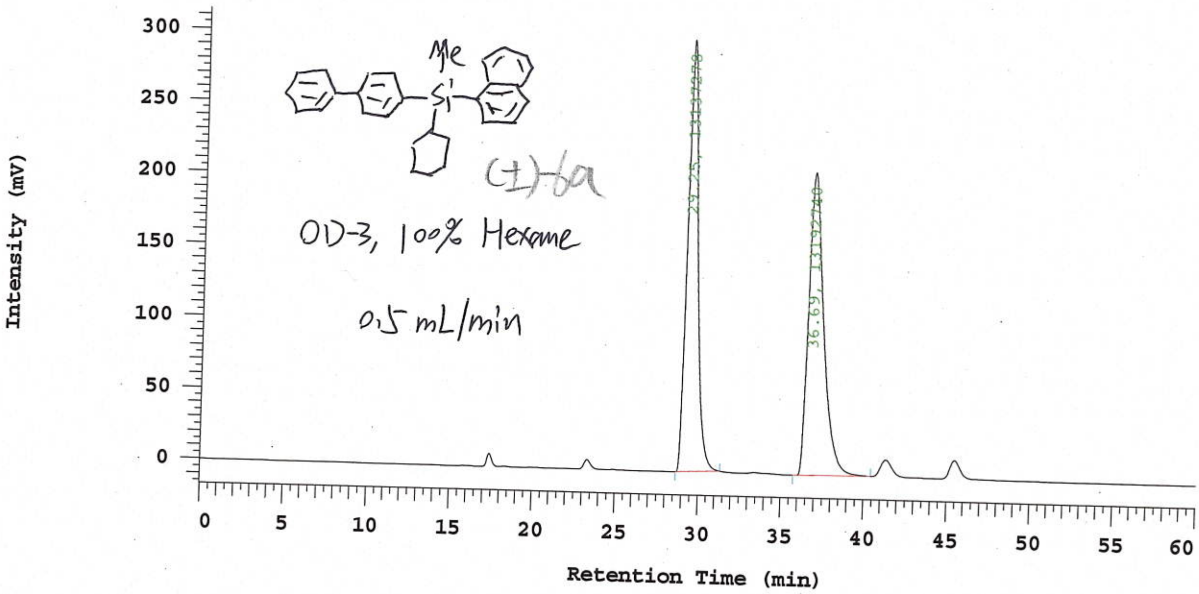

Processing Method: 0.0/100.0 iPrOH/Hexane
Column Type: OD-H 2
Method Developer: Administrator
Pump A: L-2130
Pump A Solvent A: Hexane
Pump A Solvent B: 10/90 iPrOH/Hexane
Pump A Solvent C: iPrOH
Pump A Solvent D: EtOH
Method Description:

Chrom Type: HPLC Channel : 1
Peak Quantitation: AREA
Calculation Method: AREA%

No.	RT	Area	Area %
1	29.25	13137278	49.895
2	36.69	13192740	50.105
		26330018	100.000

Peak rejection level: 0

D-2000 Elite HPLC System Manager Report

Analyzed Date and Time: 2022/02/22 18:17
Reported Date and Time: 2022/02/22 20:14
Processed Date and Time: 2022/02/22 20:14
Data Path: C:\WIN32APP\D2000HSM\Isocratic\DATA\3886\
Processing Method: 05/95 iPrOH/Hexane

System (acquisition): Sys 1
Series: 3886
Application(data): Isocratic HPLC
Vial Number: 182
Sample Name: WXH-227-OD-5%
Vial Type: UNK
Injection from this vial: 1 of 1
Volume: 10.0 ul
Sample Description:

Chrom Type: HPLC Channel : 1

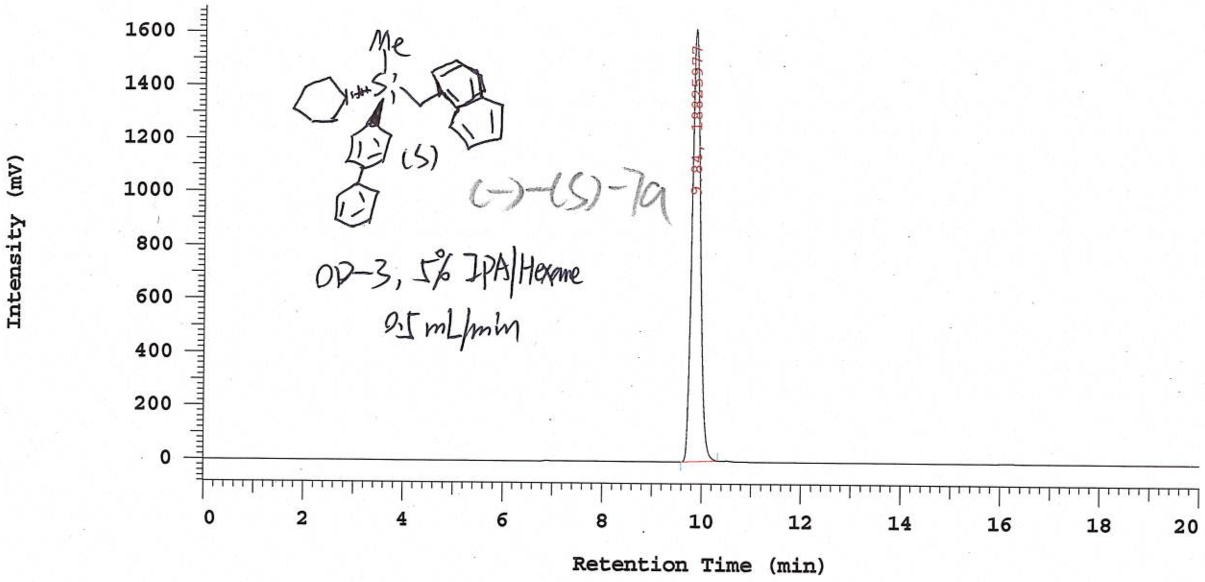

Processing Method: 05/95 iPrOH/Hexane
Column Type: OD-H 2
Method Developer: Administrator
Pump A: L-2130
Pump A Solvent A: Hexane
Pump A Solvent B: 10/90 iPrOH/Hexane
Pump A Solvent C: iPrOH
Pump A Solvent D: iPrOH
Method Description:

Chrom Type: HPLC Channel : 1
Peak Quantitation: AREA
Calculation Method: AREA%

No.	RT	Area	Area %
1	9.84	18825977	100.000
		18825977	100.000

Peak rejection level: 0

D-2000 Elite HPLC System Manager Report

Analyzed Date and Time: 2022/02/22 17:36

Reported Date and Time: 2022/02/22 20:13

Processed Date and Time: 2022/02/22 20:13

Data Path: C:\WIN32APP\D2000HSM\Isocratic\DATA\3885\

Processing Method: 05/95 iPrOH/Hexane

System (acquisition): Sys 1

Application(data): Isocratic HPLC

Sample Name: WXH-223-race-OD-5%

Injection from this vial: 1 of 1

Sample Description:

Series: 3885

Vial Number: 181

Vial Type: UNK

Volume: 10.0 ul

Chrom Type: HPLC Channel : 1

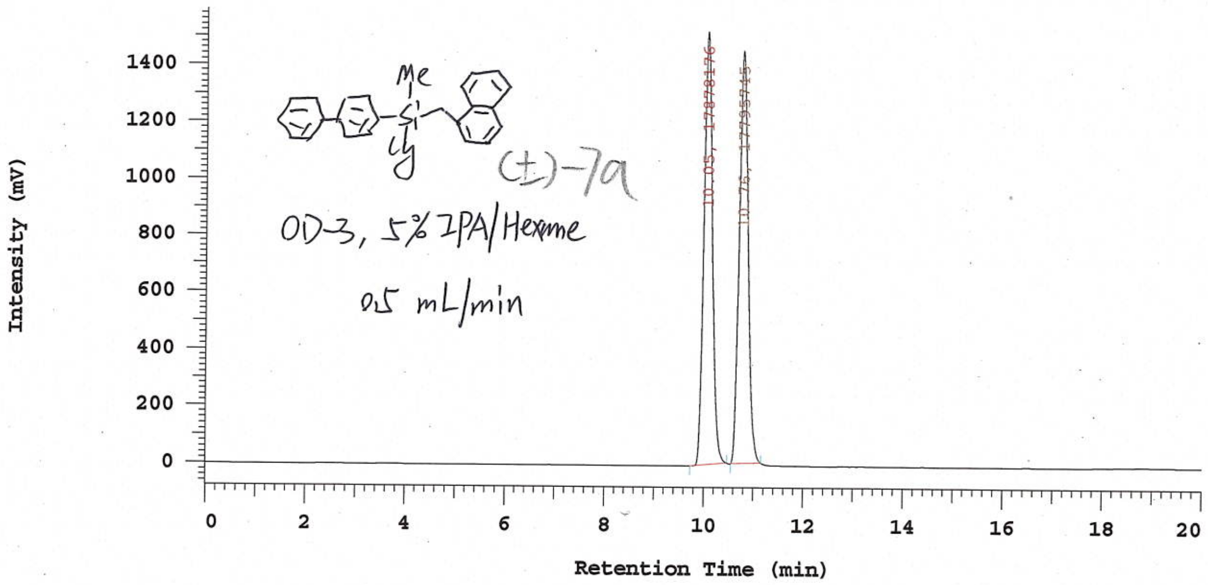

Processing Method: 05/95 iPrOH/Hexane

Column Type: OD-H 2

Method Developer: Administrator

Pump A: L-2130

Pump A Solvent A: Hexane

Pump A Solvent B: 10/90 iPrOH/Hexane

Pump A Solvent C: iPrOH

Pump A Solvent D: iPrOH

Method Description:

Chrom Type: HPLC Channel : 1

Peak Quantitation: AREA

Calculation Method: AREA%

No.	RT	Area	Area %
1	10.05	17878176	50.116
2	10.76	17795745	49.884
		35673921	100.000

Peak rejection level: 0

D-2000: Isocratic HPLC Series: 3803 Report Name: modified System: Sys 1

D-2000 Elite HPLC System Manager Report

Analyzed Date and Time: 2021/12/14 15:13 Reported Date and Time: 2021/12/14 17:06

Processed Date and Time: 2021/12/14 17:06

Data Path: C:\WIN32APP\D2000HSM\Isocratic\DATA\3803\

Processing Method: 01/99 iPrOH/Hexane

System (acquisition): Sys 1 Series: 3803

Application(data): Isocratic HPLC Vial Number: 182

Sample Name: WXH-181-OD-1% Vial Type: UNK

Injection from this vial: 1 of 1 Volume: 10.0 ul

Sample Description:

Chrom Type: HPLC Channel : 1

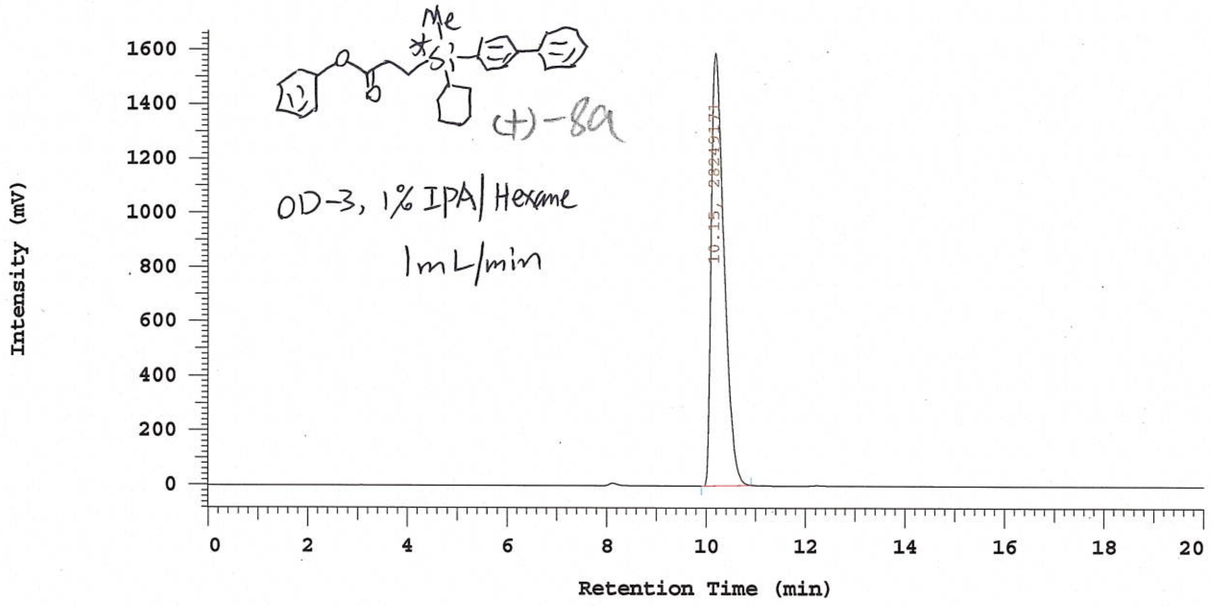

Processing Method: 01/99 iPrOH/Hexane

Column Type: OD-H 2 Method Developer: Administrator

Pump A: L-2130

Pump A Solvent A: Hexane Pump A Solvent B: 10/90 iPrOH/Hexane

Pump A Solvent C: iPrOH Pump A Solvent D: EtOH

Method Description:

Chrom Type: HPLC Channel : 1

Peak Quantitation: AREA

Calculation Method: AREA%

No.	RT	Area	Area %
1	10.15	28249171	100.000
		28249171	100.000

Peak rejection level: 0

D-2000 Elite HPLC System Manager Report

Analyzed Date and Time: 2021/12/14 16:46
Reported Date and Time: 2021/12/14 17:20
Processed Date and Time: 2021/12/14 17:20
Data Path: C:\WIN32APP\D2000HSM\Isocratic\DATA\3805\
Processing Method: 01/99 iPrOH/Hexane
System (acquisition): Sys 1
Series: 3805
Application(data): Isocratic HPLC
Vial Number: 182
Sample Name: WXH-178-OD-1%
Vial Type: UNK
Injection from this vial: 1 of 1
Volume: 10.0 ul
Sample Description:

Chrom Type: HPLC Channel : 1

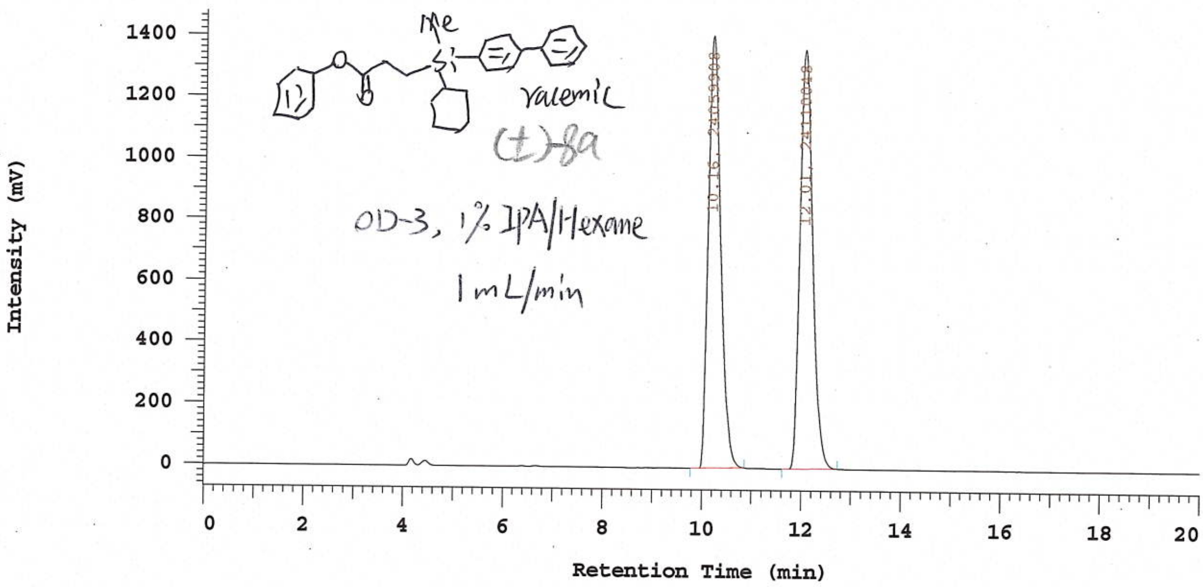

Processing Method: 01/99 iPrOH/Hexane
Column Type: OD-H 2
Method Developer: Administrator
Pump A: L-2130
Pump A Solvent A: Hexane
Pump A Solvent B: 10/90 iPrOH/Hexane
Pump A Solvent C: iPrOH
Pump A Solvent D: EtOH
Method Description:

Chrom Type: HPLC Channel : 1
Peak Quantitation: AREA
Calculation Method: AREA%

No.	RT	Area	Area %
1	10.16	24059908	49.948
2	12.01	24110048	50.052
		48169956	100.000

Peak rejection level: 0

18. References.

1. S. H. Kim, D. Ahn, Y. Y. Kang, M. Kim, K. S. Lee, J. Lee, M. H. Park, Y. Kim. *Eur. J. Inorg. Chem.* **2014**, 5107–5112.
2. Y. Tokoro, K. Sugita, S. Fukuzawa. *Chem. Eur. J.* **2015**, *21*, 13229–13232.
3. V. T. Trepohl, R. Fröhlich, M. Oestreich. *Tetrahedron*, **2009**, *65*, 6510–6518.
4. R. Shishido, M. Uesugi, R. Takahashi, T. Mita, T. Ishiyama, K. Kubota, H. Ito. *J. Am. Chem. Soc.* **2020**, *142*, 14125−14133.
5. H. G. Gudmundsson, C. J. Kuper, D. Cornut, F. Urbitsch, B. L. Elbert, E. A. Anderson. *J. Org. Chem.* **2019**, *84*, 14868−14882.
6. J. Zhu, S. Chen, C. He, *J. Am. Chem. Soc.* **2021**, *143*, 5301−5307.
7. X. Chang, P.-L. Ma, H.-C. Chen, C.-Y. Li, P. Wang. *Angew. Chem. Int. Ed.* **2020**, *59*, 8937–8940.
8. Y.-H. Huang, Y. Wu, Z. Zhu, S. Zheng, Z. Ye, Q. Peng, P. Wang, *Angew. Chem. Int. Ed.* **2022**, *61*, e202113052.
9. Z.-D. Huang, R. Ding, P. Wang, Y.-H. Xu, T.-P. Loh. *Chem. Commun.*, **2016**, *52*, 5609–612.
10. H. Guo, X. Chen, C. Zhao, W. He. *Chem. Commun.*, **2015**, *51*, 17410–17412.
11. K.-s. Lee, A. H. Hoveyda. *J. Am. Chem. Soc.* **2010**, *132*, 2898–2900.
12. (a) A. D. Becke, *Phys. Rev. A* **1988**, 38, 3098-3100. (b) A. D. Becke, *J. Chem. Phys.* **1993**, *98*, 1372–1377. (c) C. Lee, W. Yang, R. G. Parr, *Phys. Rev. B* **1988**, *37*, 785-789.
13. Gaussian 16, Revision C.01, M. J. Frisch, G. W. Trucks, H. B. Schlegel, G. E. Scuseria, M. A. Robb, J. R. Cheeseman, G. Scalmani, V. Barone, G. A. Petersson, H. Nakatsuji, X. Li, M. Caricato, A. V. Marenich, J. Bloino, B. G. Janesko, R. Gomperts, B. Mennucci, H. P. Hratchian, J. V. Ortiz, A. F. Izmaylov, J. L. Sonnenberg, D. Williams-Young, F. Ding, F. Lipparini, F. Egidi, J. Goings, B. Peng, A. Petrone, T. Henderson, D. Ranasinghe, V. G. Zakrzewski, J. Gao, N. Rega, G. Zheng, W. Liang, M. Hada, M. Ehara, K. Toyota, R. Fukuda, J. Hasegawa, M. Ishida, T. Nakajima, Y. Honda, O. Kitao, H. Nakai, T. Vreven, K. Throssell, J. A. Montgomery, Jr., J. E. Peralta, F. Ogliaro, M. J. Bearpark, J. J. Heyd, E. N. Brothers, K. N. Kudin, V. N. Staroverov, T. A. Keith, R. Kobayashi, J. Normand, K. Raghavachari, A. P. Rendell, J. C. Burant, S. S. Iyengar, J. Tomasi, M. Cossi, J. M. Millam, M. Klene, C. Adamo, R. Cammi, J. W. Ochterski, R. L. Martin, K. Morokuma, O. Farkas, J. B. Foresman, and D. J. Fox, Gaussian, Inc., Wallingford CT, 2016.
14. (a) S. Grimme, J. Antony, S. Ehrlich, H. Krieg, *J. Chem. Phys.* **2010**, *132*, 154014. (b) S. Grimme, S. Ehrlich, L. Goerigk, *J. Comput. Chem.* **2011**, *32*, 1456-1465.
15. (a) F. Weigend, R. Ahlrichs, *Phys. Chem. Chem. Phys.* **2005**, *7*, 3297-3305. (b) F. Weigend, *Phys. Chem. Chem. Phys.* **2006**, *8*, 1057-1065.G. Scalmani, M. J. Frisch, *J. Chem. Phys.* **2010**, *132,* 114110.
16. G. Scalmani, M. J. Frisch, *J. Chem. Phys.* **2010**, *132*, 114110.
